# Synthesis of Secondary Boronates via Deaminative Cross-Coupling of Alkyl Nitroso Carbamates and Boronic Acids

**DOI:** 10.1002/anie.202408432

**Published:** 2024-08-02

**Authors:** Shashwati Paul, M. Kevin Brown

**Affiliations:** Department of Chemistry, Indiana University, 800 E. Kirkwood Ave., Bloomington, IN 47405, USA

## Abstract

A strategy for transition metal-free cross-coupling of alkyl nitroso-carbamates and boronic acids is reported. The *N*-nitroso carbamates are easily prepared from the corresponding amine in two simple steps. This method allows for the synthesis of a wide variety of secondary boronates, benzylic boronates and formal Csp^3^–Csp^2^ cross-coupling products under operationally simple conditions. Functional group tolerance is also demonstrated and applied in the modification of lysine to make non-canonical amino acids.

## Graphical Abstract

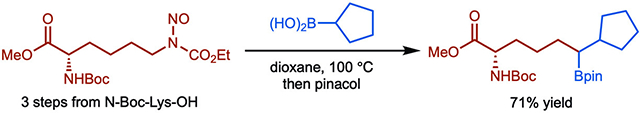

Cross-coupling reactions are one of the most important classes of transformations in chemical synthesis as they allow for the modular construction of molecules.^[1]^ Despite the wide acceptance of this methodology, the coupling of C–N bonds remains an emerging method and is challenging. Engaging C–N bonds in cross-coupling reactions is significant due to the abundance of alkyl amines among commodity chemicals, natural products, and pharmaceutical agents.^[2]^ Early work for C–N coupling focused on strained or electronically activated bonds.^[3]^ More recently, Watson and co-workers introduced several methods for Ni-catalyzed cross-coupling of Katritzky pyridinium salts, which are derived from unactivated alkyl amines, with a variety of reagents to generate Csp^3^–Csp^2^ and Csp^3^–Csp^3^ bonds (Scheme 1A).^[4]^ In these reactions, an alkyl radical is generated as a key intermediate. This area of research has been rapidly expanded upon by others.^[5]^ The Rovis group recently reported the coupling of the C–N bond of an imine, which has shown promise for constructing hindered C-C bonds.^[6,7]^ Due to the value of deaminative cross-coupling reactions, we envisioned an alternative mode of activation that would involve the generation of a diazo compound and subsequent reaction with a boronic acid under a transition metal-free process (Scheme 1A). This type of approach would not only cleave the C–N bond but generate a product that contains a valuable C–B bond.^[8–10]^

Prior work for the synthesis of diazos from amines and subsequent capture with a boronic ester/acid is illustrated in Scheme 1B. In 2014, Wang published the cross-coupling of α-aminoesters with boronic acids to deliver α-aryl esters.^[11]^ The reaction operated by diazotization of amines in situ and subsequent trapping with alkyl/aryl boronic acids. A recent report from the Morandi group showcased the coupling reaction between benzylic amines and aryl boronic acids to deliver diarylmethanes.^[12]^ For these two cases, protodeborylation was observed due to the instability of the benzylic boronic acids under the reaction conditions. In 2013, Molander reported the synthesis of α-trifluoromethylated alkyl boron compounds by treating a solution of CF_3_CHN_2_ with different alkyl and aryl boronic acids.^[13a]^ Later, Szabo reported an enantioselective variant of this method.^[13b]^ Diazos derived from hydrazones have also been shown to undergo coupling with boronic acids to deliver alkyl boronate derivatives.^[14]^ However, diazos generated from unactivated alkyl amines and coupling with organoboranes has not been fully realized. In this manuscript, we report a method to achieve the coupling of *N*-nitroso-carbamates derived from unactivated alkyl amines and aryl/alkyl boronic acids to synthesize secondary boronates. *N*-nitroso carbamates are easily prepared from alkyl amines and used after a simple filtration through silica gel.^[15]^ The method is significant because a broad range of amines can be utilized, including lysine, which allows for the synthesis of non-canonical amino acids (Scheme 1C).

Inspiration for developing the deaminative cross-coupling came from the known base-mediated decomposition of *N*-nitroso-tosylamides to diazos. This has been most notably accomplished using *N*-nitroso-tosylmethylamide (Diazald) to generate diazomethane.^[16]^ When we treated *N*-nitroso-tosylamide **1** with Cs_2_CO_3_ and *n*-BuB(OH)_2_ (**5)** in dioxane at 100 °C, the desired product **4** formed in 20% yield (Scheme 2A). Inspired by literature reports, additional optimization were conducted by variation of the electron-withdrawing group on the nitrogen, which ultimately led to the use of carbamate **3** to generate **4** in 95% yield (Scheme 2A).^[17]^ Typically, the starting nitroso-carbamates were prepared in two steps from the amine by first synthesis of the carbamate followed by treatment of the crude reaction mixture with sodium nitrite under acidic conditions. The products were easily purified by passage through a short plug of silica gel. For amines containing acid labile groups, the nitrosation reaction was performed with nitrosonium tetrafluoroborate (NO^+^BF_4_^−^).^[18,19]^

With a set of optimized conditions in hand, the reaction was probed further (Scheme 2B, entry 1). The use of K_2_CO_3_ decreased the yield slightly (Scheme 2B, entry 2). Base-free conditions could also be used, albeit with a decrease in yield (Scheme 2B, entry 3). While 3 equiv. of *n*-BuB(OH)_2_ resulted in near quantitative yield, use of 2 equiv. also provided the product in good yield (Scheme 2B, entry 4). Finally, the reaction temperature could also be reduced, albeit with a slight drop in yield (Scheme 2B, entry 5). It is worth mentioning that the reaction works under aerobic conditions using benchtop solvent without any decrease in the yield, which further underscores the robustness of the process.^[20]^

Based on literature reports for base-mediated decomposition of *N*-nitroso carbamates, a plausible mechanism is proposed in Scheme 1C.^[22]^ Treatment of **I** with base at elevated temperatures leads to the formation of diazoate **II**, which undergoes conversion to diazoalkane **III**. The addition of the boronic acid forms Lewis acid/base adduct **IV**, which is primed for migration to generate **V**. With alkyl boronic acids, the addition of pinacol results in the formation of the Bpin-containing products. In the case of aryl boronic acids, protodeborylation occurs under the reaction conditions to generate the alkane (see below). (Scheme 2C).

Under the optimized set of conditions, a range of boronic acids and *N*-nitroso carbamates were explored (Scheme 3). Methyl, primary and secondary boronic acids worked well in the reaction (e.g., products **8**–**10**). Tertiary boronic acids, however, failed to react. Primary and β-branched primary amines worked well in the reaction; however, α-branched amines (e.g., **23**) did not undergo reaction (<10% yield). The method was tolerant of pendant tertiary amines (products **12**–**13**), carbamates (products **8**–**10**), esters (product **19**), pyridines (products **14**–**15**), thiophenes (products **16**–**17**), and ethers (product **21**). In addition, benzyl-protected 1,2-amino alcohol could be used (product **22**). Moreover, primary alcohol worked well in the reaction (product **20**). Aryl boronic acids were also employed in the optimized reaction conditions. Due to the instability of the benzyl boronates under the basic reaction conditions, protodeborylation was observed (products **24**–**28**). In these cases, tolerance to an acid (product **24**), ester (product **25**), alkene (product **26**), pyridine (product **27**) and tertiary amine (product **28**) was demonstrated (Scheme 3).

As shown in Scheme 4A, we have extended the reaction to the use of alkylBF_3_K salts. These salts are useful because of their high stability and crystallinity. Under the standard conditions, ~20% yield was observed due to the slow hydrolysis of the BF_3_K to the boronic acids. To circumvent this issue, we performed the reaction in a toluene/H_2_O mixture in the presence of Et_3_N, which resulted in the formation of product **4** in 95% yield. Under these conditions, facile in situ hydrolysis of the BF_3_K occurred, as has been demonstrated previously (Scheme 4A).^[21]^

As noted previously, coupling with arylboronic acids resulted in products from protodeborylation (Scheme 3). As the coupling works in the absence of base (see Scheme 2B, entry 3), we suspected that this might be a tactic to mitigate protodeborylation. However, under base free conditions, mixtures of the protodeborylated and borylated products were still generated (see the Supporting Information). Based on literature reports, decomposition of alkyl nitrosoureas can occur at room temperature in the presence of a strong base.^[22]^ Ultimately, it was identified that treating *N*-nitroso urea **29** with LiOEt at room temperature afforded the desired borylated product **31**. In these cases, alcohols **32**–**35** was isolated to facilitate purification (Scheme 4B).

One of the most significant aspects of deaminative coupling is the conversion of naturally occurring amines to value-added products. Non-canonical amino acids play an important role in a variety of settings from protein labelling to bioconjugation handles, to amino acid-based drugs.^[23]^ The classical strategy for non-canonical amino acid synthesis typically involves de novo synthesis.^[24]^ Another less explored strategy involves the reaction of canonical amino acids. Among many notable methods, Watson, Kalyani and Dion reported a nickel-catalyzed deaminative coupling of lysine with aryl bromides to generate a wide range of non-canonical amino acids.^[4e]^

*N*-nitroso carbamate **36** was easily prepared from *N*-Boc-Lys-OH in three steps. Treatment of **36** under the standard conditions allowed for the formation of **38** in good yield, however, partial epimerization occurred due to the presence of a base in the reaction mixture (Scheme 4C). Optimization was then conducted to identify base-free conditions. Ultimately, it was found that heating **36** with boronic acid **37** led to the formation of **38** in good yield without racemization (>99:1 er). While the product of a coupling of an aryl boronic acid **38** can also be accessed with the method developed by Watson, Kalyani and Dion,^[4e]^ a key feature of this method is that coupling of alkyl boronic acids generates new non-canonical amino acids (products **39**–**42**), which contain a versatile C-Bpin motif. This modified Bpin-lysine **42** is stable and, once deprotected with TFA, can be coupled to Boc-L–Leu-OH to generate borylated dipeptide **44** in 67% yield over two steps.^[25]^ In principle, the C–B bond can be further modified to create a diverse range of structures.

Finally, a one-pot reaction sequence was developed for the deaminative cross-coupling (Scheme 4D). The starting carbamate **45** was treated with nitrosonium tetrafluoroborate NO^+^BF_4_^−^ in dioxane at room temperature. Subsequent addition of *n*-BuB(OH)_2_ and Cs_2_CO_3_ followed by heating and final oxidation with hydrogen peroxide afforded the secondary alcohol **46** in 46% yield (3 steps) on 4 mmol scale.

In conclusion, we have developed a metal-free cross-coupling of nitroso-carbamates prepared from commercially available primary amines and alkyl boronic acids to construct complex secondary alkyl boronates. Reaction conditions were also developed for the synthesis of benzylic boronates. Finally, non-canonical amino acids could be accessed from L-lysine with conservation of enantiopurity. Further exploration of the reactivity of nitroso carbamates is under exploration.

## Supplementary Material

Supporting Info

## Figures and Tables

**Scheme 1.**
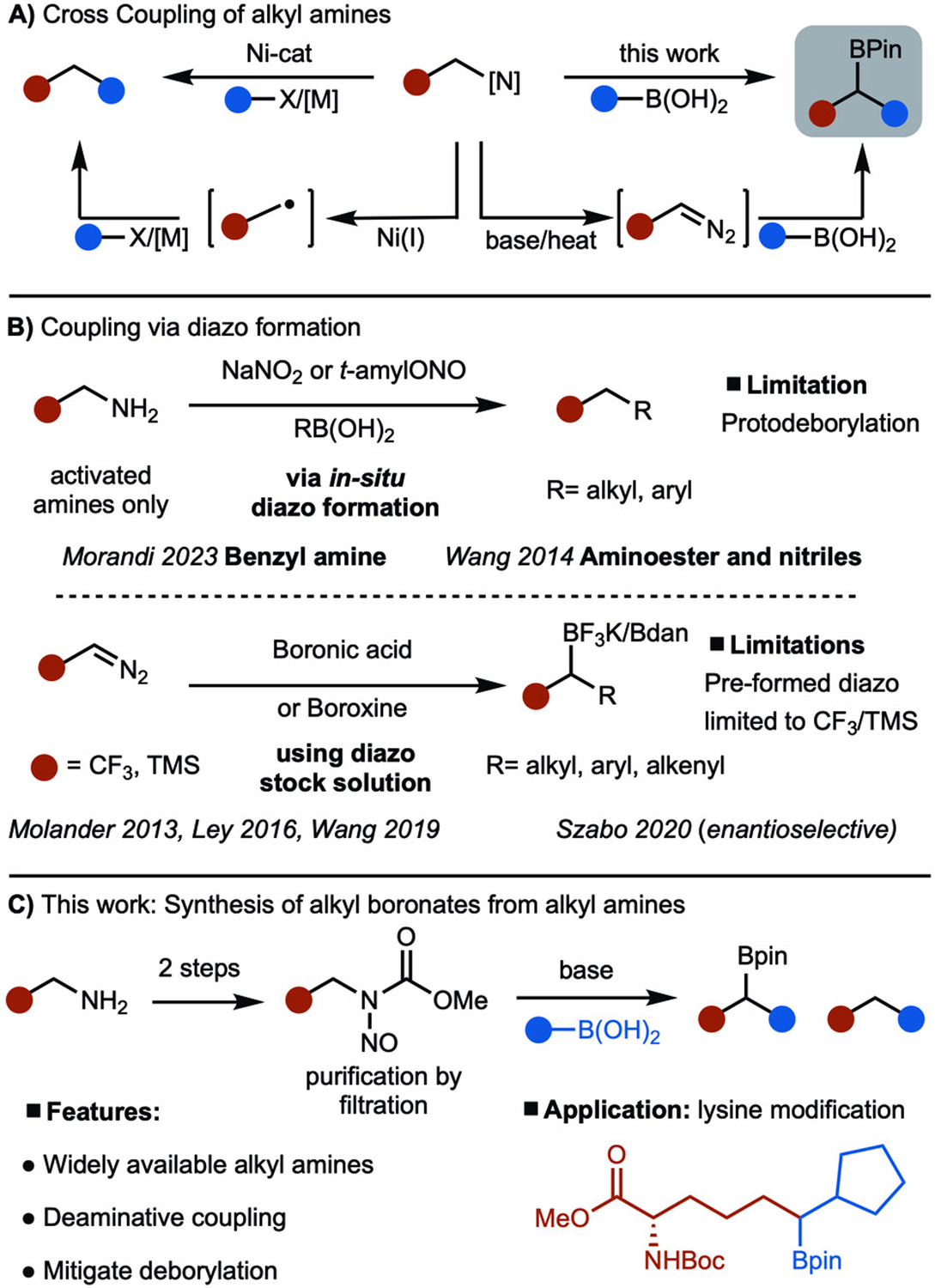
Coupling of organoboronic acids and amine derivatives.

**Scheme 2.**
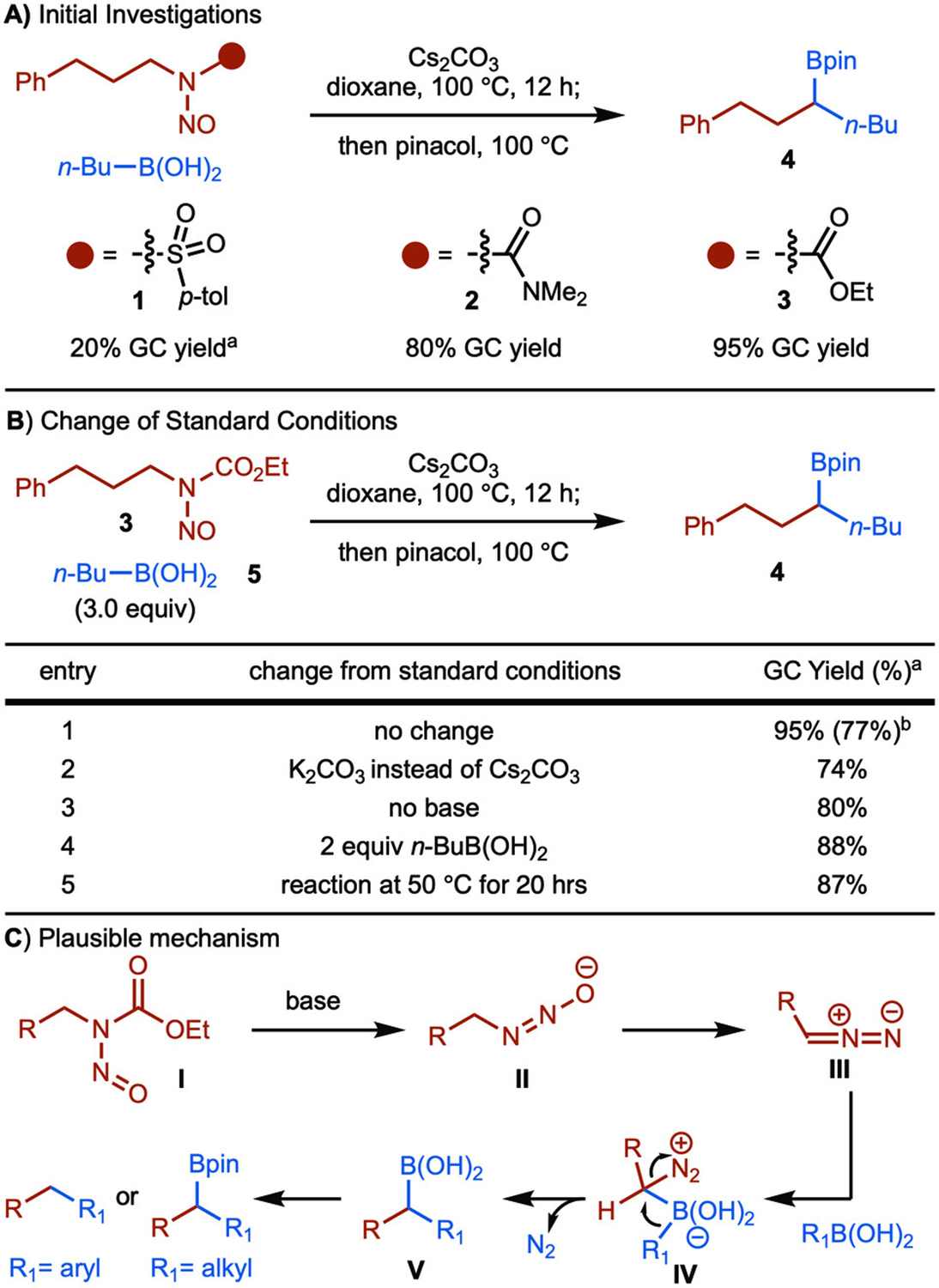
Initial studies. ^a^ Yields were determined by GC analysis of the unpurified reaction mixture with dodecane as internal standard. ^b^ Yield of isolated product after oxidation with H_2_O_2_.

**Scheme 3.**
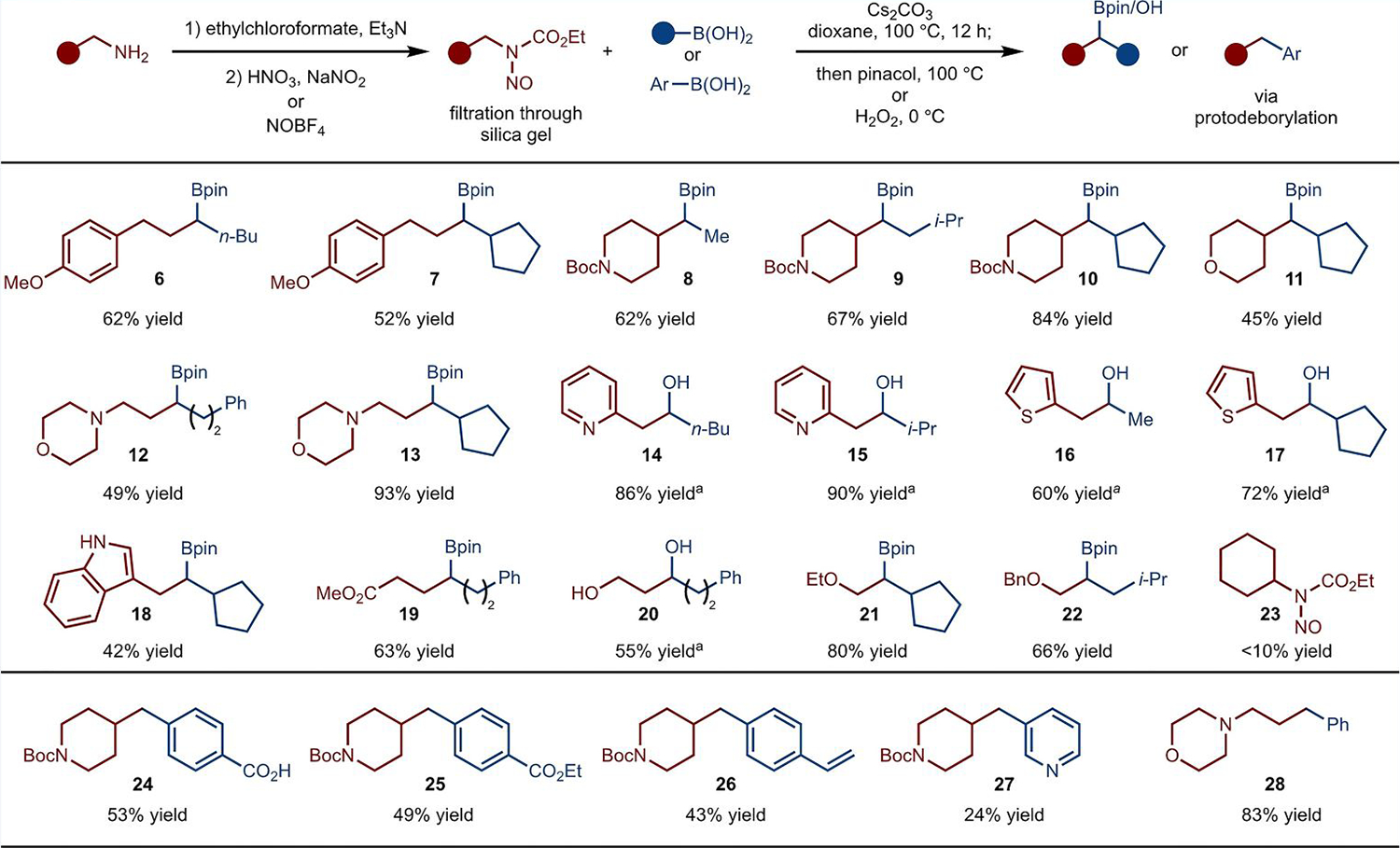
Substrate scope. ^a^ Yield of isolated product after oxidation with H_2_O_2_.

**Scheme 4.**
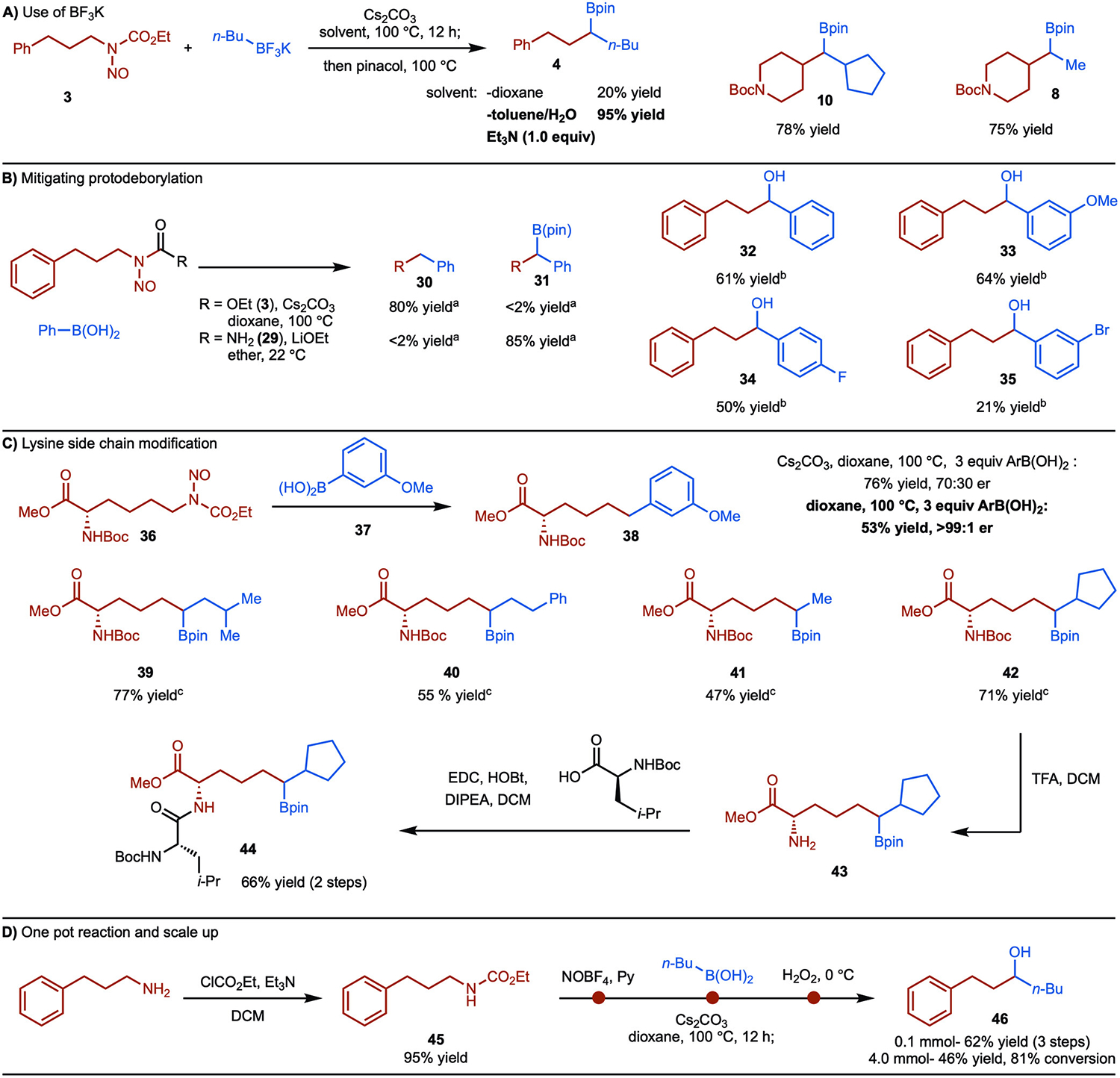
Further applications. ^a^ Yields are determined by GC analysis of the unpurified reaction mixture with dodecane as internal standard. ^b^ Yield of isolated product after oxidation with H_2_O_2_^c^ Pinacol was added at the completion of the coupling reaction.

## Data Availability

The data that support the findings of this study are available in the supplementary material of this article.
