# Supplementary material for: Synthesis of Secondary Boronates via Deaminative Cross-Coupling of Alkyl Nitroso Carbamates and Boronic Acids
Source: Angew Chem Int Ed Engl. Author manuscript; Available in PMC 2025 Sep 9. (PMC11733801; doi:10.1002/anie.202408432)
Supplement: Supporting Info [file NIHMS2046758-supplement-Supporting_Info.pdf]

Angewandte Chemie
Eine Zeitschrift der Gesellschaft Deutscher Chemiker

Supporting Information

# Synthesis of Secondary Boronates via Deaminative Cross-Coupling of Alkyl Nitroso Carbamates and Boronic Acids

*S. Paul, M. K. Brown**

# Synthesis of Secondary Boronates via Deaminative Cross-Coupling of Alkyl Nitroso Carbamates and Boronic Acids

Shashwati Paul and M. Kevin Brown*,

Department of Chemistry, Indiana University, 800 E. Kirkwood Ave, Bloomington, IN 47405

**Supplementary information- Table of contents**

I. General Information……………………………………………………………………2

II. Reagents…………………………………………………………………………..3-6

III. Reaction optimization……………………………………………………………7-9

IV. General procedure 1 …………………………..…………………………………...8-20

V. General procedure 2………………………………………….…………………..20-36

VI. General procedure 3…………………………………………………………...36-40

VII. General procedure 4…………………………………………………………...40-41

VIII. General procedure 5…………………………………………………………...42-45

IX. General procedure 6…………………………………………………………...45-51

X. Spectra……………………………………………………………………………...52-101

XI. HPLC……………………………………………………………………………102-104

XII. References…………………………………………………………………………..105

## I. General information:

**NMR:** NMR ($^{1}H$, $^{13}C$) were recorded at room temperature on a Varian I500, Varian I600 or a Bruker 500 spectrometer. $^{13}C$ were recorded on a Bruker 500 (126 MHz) or Varian I500 (126 MHz) spectrometer with complete proton decoupling. $^{1}H$ Chemical shifts are recorded in ppm with the residual solvent resonance as the internal standard ($CDCl_3$ 7.26 ppm). $^{13}C$ Chemical shifts are recorded in ppm with the residual solvent resonance as the internal standard ($CDCl_3$ 77.16 ppm). Data are reported as follows: chemical shifts (ppm), multiplicity (s = singlet, d = doublet, t = triplet, q = quartet, br = broad, m = multiplate etc.), coupling constants (Hz), integration.

**IR:** Infrared spectra were recorded on a Bruker Tensor ii FT-IR spectrometer, $v_{max}$ in $cm^{-1}$. Bands are characterised as s = strong, m = medium, br = broad, w = weak etc.

**HRMS:** High-Resolution Mass Spectrometry analysis was obtained using Electrospray Ionization (ESI) technique using a Wayters/Micromass LCT classic (ESI-TOF) and reported as *m/z* (relative intensity), electron Impact Ionization (EI) methods.

**HPLC:** Chiral HPLC analysis was performed on an Agilent 1220 Infinity LC system using a chiral column eluted with a mixture of hexane and isopropanol.

**Solvent:** Dichloromethane ($CH_2Cl_2$), diethyl ether ($Et_2O$), dimethyl formamide (DMF), tetrahydrofuran (THF), acetonitrile (MeCN) were purified under a positive pressure of Argon by passing through two columns of activated alumina. Toluene (PhMe) was purified under a positive pressure of dry argon by passing through activated alumina and $Q_5$ (Grubbs apparatus).

**Reaction:** All reactions were carried out in degassed and distilled solvents under an atmosphere of nitrogen in oven-dried glassware with the Schlenk line technique. All workup and purification were carried out using reagent-grade solvent under air.

**Purification:** Medium-pressure liquid chromatography (MLPC) technique was used for purification using a Teledyne ISCO CombiFlash Rf 150 instrument. Standard flash column chromatography (FCC) techniques ZEOprep 60/40-63 µm silica gel was used for purification.

## II. Reagents:

**1-Hydroxybenzotriazole - hydrate (HOBT)** was purchased from Oakwood and used as received.

**1-(3-Dimethylaminopropyl)-3-ethylcarbodiimideHydrochloride (EDCI)** was purchased from Oakwood and used as received.

**2-(1H-indol-3-yl)ethan-1-amine** was purchased from Sigma Aldrich and used as received.

**(2-ethoxyethyl)amine oxalate** was purchased from Combi Blocks and used as received.

**(3-bromoxyphenyl)boronic acid** was purchased from Combi blocks and used as received.

**(3-methoxyphenyl)boronic acid** was purchased from Combi blocks and used as received.

**(4-(ethoxycarbonyl)phenyl)boronic acid** was purchased from Matrix Scientific and used as received.

**(4-vinylphenyl)boronic acid** was purchased from Sigma Aldrich and used as received.

**2-(1H-indol-3-yl)ethan-1-amine** was purchased from Sigma Aldrich and used as received.

**2-(Phenylmethoxy)ethylamine** was purchased from Ambeed and used as received.

**2-(Pyridin-2-yl)ethylamine** was purchased from Ambeed and used as received.

**2-Thiopheneethanamine** was purchased from Ambeed and used as received**.**

**3-Amino-propionic acid** was purchased from Oakwood and used as received**.**

**3-(Morpholin-4-yl)propan-1-amine** was purchased from Ambeed and used as received.

**3-phenylpropan-1-amine** was purchased from Alfa Aesar and used as received**.**

**4-(Aminomethyl)piperidine ,N1-BOC** was purchased from Ambeed and used as received.

**4-carboxyphenylboronic acid** was purchased from A K Scientific and used as received.

**benzenepropanamine, 4-methoxy-** was purchased from Combi Blocks and used as received.

**BOC-L-Leucine-OH** was purchased from Sigma Aldrich and used as received.

**Butylboronic acid** was purchased from Combi Blocks and used as received.

**cesium carbonate** was purchased from Strem Chemicals, Inc. and used as received.

**cyclopentylboronic acid** was purchased from Ambeed and used as received.

**Dihexyl** was purchased from VWR International and used as received.

**Diisopropylethylamine** was purchased from Sigma Aldrich and distilled over $CaH_2$ before using.

**Dioxane** was purchased from Sigma Aldrich and used as received.

**Ethyl chloroformate** was purchased from Sigma Aldrich and used as received.

**Hydrogen peroxide** was purchased from Macron and used as received.

**isobutylboronic acid** was purchased from ambeed and used as received.

**isopropylboronic acid** was purchased ambeed from and used as received.

**LiOEt** was purchased from Sigma Aldrich and used as received.

**methylboronic acid** was purchased from Ambeed and used as received.

**nitrosyl tetrafluoroborate** was purchased from Oakwood and used as received.

**phenethyl boronic Acid** was purchased from Ambeed and used as received.

**Phenyl boronic acid** was purchased from Matrix Scientific and used as received.

**Pinacol** was purchased from Oakwood and used as received.

**Potassium bifluoride** was purchased from Oakwood and used as received.

**potassium trifluoro(methyl)borate** was purchased from Ambeed and used as received.

**pyridin-3-ylboronic acid** was purchased from Matrix Scientific and used as received.

**Pyridine** was purchased from Macron and distilled over $CaH_2$ before using.

**Sodium nitrite** was purchased from Mallinckrodt and used as received.

(**tert-butoxycarbonyl)-L-lysine** was purchased from Sigma Aldrich and used as received.

**tetrahydro-2H-pyran-4-ylmethylamine** was purchased from Ambeed and used as received.

**Triethylamine** was purchased from Sigma Aldrich and distilled over $CaH_2$ before using.

## III. Reaction optimization-

**Table A) Coupling with alkyl boronic acid**

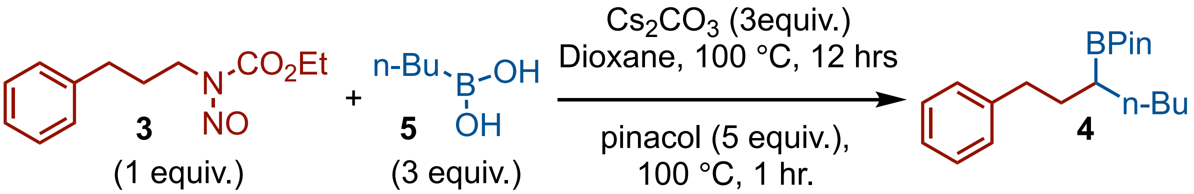


| Entry | Change from the above condition | yield[a] |
|---|---|---|
| 1 | none | 95%(77%)[b] |
| 2 | $K_2CO_3$, Dioxane | 74% |
| 3 | no base, Dioxane | 80% |
| 4 | $Cs_2CO_3$ as base, DCE as solvent | quant |
| 5 | $Cs_2CO_3$ as base, PhCl as solvent | 88% |
| 6 | $^nBuB(OH)_2$ 2 equiv. | 88% |
| 7[c] | reaction ran at 50 °C | 88% |
| 8 | reaction ran at 120 °C | 98% |
| 9[c] | reaction ran at rt | 40%[c] |
| 10 | **SI-3** instead of **3** | 20% |
| 11 | **SI-4** instead of **3** | 80% |
| 12 | Insitu generation of **3** with tert-butylnitrite | trace |
| 13 | Insitu generation of **3** with $NO^+BF_4^-$ | trace |
| 14 | coupling with **SI-5** | 20% |
| 15 | coupling with in-situ generated $nBu(OH)_2$ from **SI-5** | 99% |

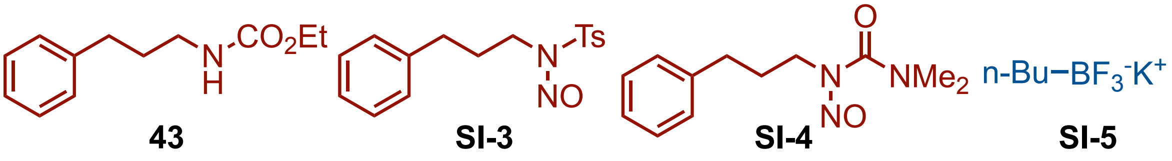


[a]Yield was determined by GC analysis using dodecane as internal standard.
[b]Isolated yield after oxidation with $H_2O_2$. [c]Reaction ran for 48 hrs.

**Optimization procedure:** A reaction tube equipped with a stir bar was charged with ethyl nitroso(3-phenylpropyl)carbamate (24 mg, 1 Eq, 0.10 mmol), caesium carbonate (99 mg, 3 Eq, 0.30 mmol), and butyl boronic acid (31 mg, 3 Eq, 0.30 mmol). The tube was closed

with a septum and evacuated/backfilled with nitrogen. Next dioxane (0.1 M, 1 ml) was added to the tube and the septa were replaced with a screw cap. Next, the reaction mixture was placed in a metal block heated to 100 °C. The reaction mixture was stirred at that temperature for 16 hrs. Next, the reaction was cooled to room temperature, and pinacol (60 mg, 5 Eq, 0.51 mmol) was added and the reaction mixture was again placed in the metal block for 1 hr. After that, the reaction was cooled to room temperature, diluted with ethyl acetate, and passed through a pad of celite. Dodecane (17 mg, 23 µL, 1 Eq, 0.10 mmol) was added as an internal standard to the filtrate and the mixture was subjected to gas chromatography. [1]

**Table B) Coupling with aryl boronic acid**

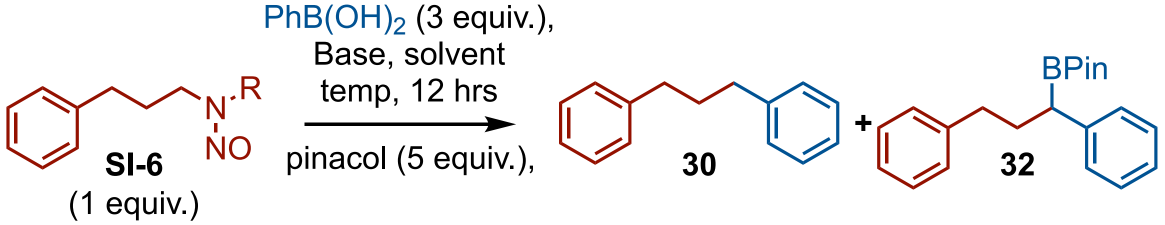


| Entry | R | Base | Solvent | Temp | yield (30:32) |
|---|---|---|---|---|---|
| 1 | $CO_2Et$ | $Cs_2CO_3$ | Dioxane | 100 °C | 80:<2 |
| 2 | $CO_2Et$ | no base | Dioxane | 100 °C | 27:20 |
| 3 | $CO_2NH_2$ | LiOEt (3 equiv.) | ether | RT | <2:56 |
| 4 | $CO_2NH_2$ | KOH (3 equiv.) | ether | RT | <2:40 |
| 5 | $CO_2NH_2$ | LiOEt (6 equiv.) | ether | RT | <2: 85 (61%)[b] |

[a]Yield was determined by GC analysis using dodecane as internal standard.
[b]Isolated yield after oxidation with $H_2O_2$.

**Optimization procedure:** A reaction tube equipped with a stir bar was charged with starting material (1 Eq, 0.10 mmol), base (as mentioned in the **Table B**), and phenyl boronic acid (36.58 mg, 3 Eq., 300.0 µmol). The tube was closed with a septum and evacuated/backfilled with nitrogen. Next dioxane (0.1 M, 1 ml) was added to the tube and

the septa were replaced with a screw cap. Next, the reaction mixture was placed in a metal block heated to 100 °C. The reaction mixture was stirred at that temperature for 16 hrs. Next, the reaction was cooled to room temperature, and pinacol (60 mg, 5 Eq, 0.51 mmol) was added and the reaction mixture was again placed in the metal block for 1 hr. After that, the reaction was cooled to room temperature, diluted with ethyl acetate, and passed through a pad of celite. Dodecane (17 mg, 23 µL, 1 Eq, 0.10 mmol) was added as an internal standard to the filtrate and the mixture was subjected to gas chromatography.

## IV. General procedure 1- Starting material synthesis:

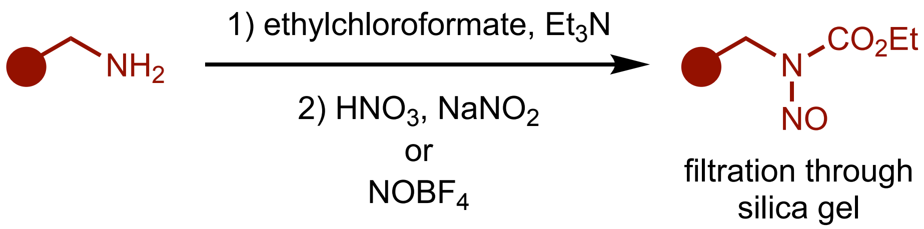


An oven-dried 25 ml round bottom flask equipped with a stir bar was evacuated/back-filled three times with nitrogen. The flask was closed with a septa and a nitrogen line was placed into it. Next amine (1 Eq.) was added followed by $CH_2Cl_2$ (0.33 M). Next triethylamine (1.1 Eq.) was added and the reaction mixture was cooled to 0 °C for 5 mins. After that ethyl chloroformate (1.05 Eq.) was added dropwise to the cold solution and the reaction mixture was stirred at that temperature for 5 more mins. Next, the flask was moved to room temperature and stirred for 18 hours. Upon completion, the reaction mixture was quenched with sat $NH_4Cl$ solution and the aqueous layer was extracted with $CH_2Cl_2$ (3 times, 20 ml each). The combined organic layers were washed with sat. $NaHCO_3$ solution and followed by brine. The organic layer was dried over $Na_2SO_4$ and

filtered. The filtrate was evaporated to dryness and the crude mixture was proceeded to the next step without further purification.

**A) Nitrosation with $NaNO_2$:** The crude reaction mixture from the previous step was dissolved in diethyl ether (1 M) and water (1 M) and the reaction mixture was placed in an ice-water bath to keep the temperature below 15 °C. To this mixture sodium nitrite (20 Eq.) was added followed by dropwise addition of 10 ml 1:1 $HNO_3$/water mixture to maintain a green colour of the solution. The mixture was stirred at this temperature for 2-3 hrs and disappearance of the starting material was monitored via thin layer chromatography. Next, the reaction mixture was extracted with diethyl ether (2 times, 10 ml), and the combined organic layer was dried over $Na_2SO_4$, filtered, and evaporated till dryness. Next, the crude mixture was dissolved in 10% ethyl acetate/hexane and passed through a pad of silica to afford the nitroso-carbamate.

**B) Nitrosation with $NO^+BF_4^-$** : The crude carbamate was dissolved in dry acetonitrile (0.4 M) and placed under nitrogen. To this pyridine (2 Eq.) was added, and following the reaction mixture was cooled to -30 °C (acetonitrile/dry ice bath). To this cold solution was added nitrosyl tetrafluoroborate (1.3 Eq.) in a few portions. The reaction was stirred at -30°C for 30 mins and then at 0 °C for two hours. The completion of the reaction was ensured by thin-layer chromatography. Next ice water and ethyl acetate were added to the reaction mixture. The organic phase was separated and the organic layer was washed with sat $NH_4Cl$ solution to remove pyridine. The organic phase was washed with brine, dried over $Na_2SO_4$, filtered, and evaporated. Next, the crude mixture was dissolved in 10% ethyl acetate/hexane and passed through a pad of silica to effort the nitroso-

carbamate. *Note: This nitrosation procedure was performed on amines containing acid-labile functional groups.*

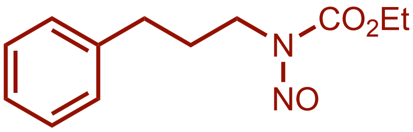


**3**

**ethyl nitroso(3-phenylpropyl)carbamate:** This compound was synthesized according to general procedure **1A** using 3-phenylpropan-1-amine (1.4 g, 1 Eq, 10 mmol) to afford **SI-1** as a yellow oil (600 mg, 2.54 mmol, 53 % over two steps). **$R_f$**: 0.5 in 10% EA/Hex.

**$^1$H NMR (500 MHz, $CDCl_3$)** δ 7.26 – 7.17 (m, 2H), 7.16 – 7.04 (m, 3H), 4.46 (q, *J* = 7.1 Hz, 2H), 3.75 – 3.66 (m, 2H), 2.49 (t, *J* = 7.8 Hz, 2H), 1.66 (p, *J* = 7.7 Hz, 2H), 1.38 (t, *J* = 7.1 Hz, 3H).

**$^{13}$C NMR (126 MHz, $CDCl_3$)** δ 154.1, 140.7, 128.6, 128.4, 126.3, 64.6, 40.8, 33.2, 28.4, 14.4.

**IR (neat):** 2985 (w), 1745 (s), 1497 (m), 1401 (m), 1379 (m), 1136 (s).

**HRMS (ESI):** Calculated for $C_{12}$ $H_{16}$ $O_3$ $N_2$ Na $[M+Na]^+$ 259.1053; found 259.1054.

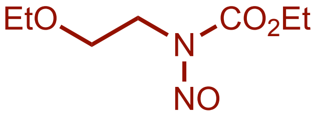


**SI-7**

**ethyl (2-ethoxyethyl)(nitroso)carbamate:** This compound was synthesized according to general procedure **1A** using (2-ethoxyethyl)amine oxalate (446 mg, 523 µL, 1 Eq, 5.00 mmol) to afford **SI-7** as a yellow oil (730 mg, 3.84 mmol, 76.8 % over two steps). **$R_f$**: 0.5 in 10% EA/Hex.

$^{1}$**H NMR (500 MHz, CDCl$_3$)** δ 4.55 (q, *J* = 7.2 Hz, 2H), 3.98 (t, *J* = 6.0 Hz, 2H), 3.46 – 3.37 (m, 4H), 1.46 (t, *J* = 7.1 Hz, 3H), 1.12 (t, *J* = 7.0 Hz, 3H).

$^{13}$**C NMR (126 MHz, CDCl$_3$)** δ 154.1, 66.4, 66.1, 64.6, 40.2, 15.1, 14.4.

**IR (neat):** 2979 (w), 2872 (w), 1749 (s), 1515 (m), 1379 (m), 1132 (s).

**HRMS (ESI):** TBD

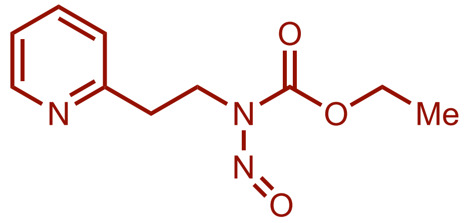


**SI-8**

**ethyl nitroso(2-(pyridin-2-yl)ethyl)carbamate:** This compound was synthesized according to general procedure **1A** using 2-(Pyridin-2-yl)ethylamine (611 mg, 0.51 mL, 1 Eq, 5.00 mmol) to afford **SI-8** as a yellow oil (900 mg, 4.03 mmol, 80.6 % over two steps) **R$_f$**: 0.5 in 50% EA/Hex.

$^{1}$**H NMR (500 MHz, CDCl$_3$)** δ 8.53 (d, *J* = 4.8 Hz, 1H), 7.58 (td, *J* = 7.7, 1.8 Hz, 1H), 7.13 (dd, *J* = 7.5, 4.9 Hz, 1H), 7.08 (d, *J* = 7.8 Hz, 1H), 4.48 (q, *J* = 7.1 Hz, 2H), 4.15 (dd, *J* = 8.1, 6.7 Hz, 2H), 2.86 (t, *J* = 7.4 Hz, 2H), 1.40 (t, *J* = 7.1 Hz, 3H).

$^{13}$**C NMR (126 MHz, CDCl$_3$)** δ 157.8, 153.9, 149.7, 136.6, 123.3, 121.9, 64.5, 40.6, 35.3, 14.4.

**IR (neat):** 2984 (w), 1745 (s), 1591 (w), 1506 (m), 1474 (m), 1378 (m), 1136 (s).

**HRMS (ESI):** Calculated for $C_{10}\,H_{14}\,O_3\,N_3$ $[M+H]^+$ 224.1030; found 224.1030.

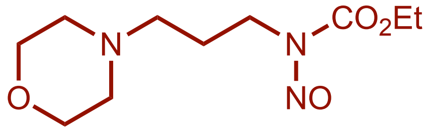


**SI-9**

**ethyl (3-morpholinopropyl)(nitroso)carbamate:** This compound was synthesized according to general procedure **1A** using 3-(Morpholin-4-yl)propan-1-amine (721 mg, 731 µL, 1 Eq, 5.00 mmol) to afford **SI-9** as a yellow oil (700 mg, 2.85 mmol, 57.1 % over two steps) **R$_f$**: 0.5 in 50% EA/Hex.

**$^1$H NMR (500 MHz, CDCl$_3$)** δ 4.54 (q, *J* = 7.1 Hz, 2H), 3.87 – 3.78 (m, 2H), 3.68 (t, *J* = 4.7 Hz, 4H), 2.37 (t, *J* = 4.6 Hz, 4H), 2.29 (t, *J* = 7.0 Hz, 2H), 1.58 (p, *J* = 7.0 Hz, 2H), 1.46 (t, *J* = 7.1 Hz, 3H).

**$^{13}$C NMR (126 MHz, CDCl$_3$)** δ 154.1, 67.0, 64.6, 56.1, 53.6, 39.5, 23.8, 14.4.

**IR (neat):** 2962 (w), 1747 (s), 1506 (m), 1402 (w), 1139 (s).

**HRMS (ESI):** Calculated for $C_{10}H_{20}O_4N_3$ $[M+H]^+$ 246.1448; found 246.1448.

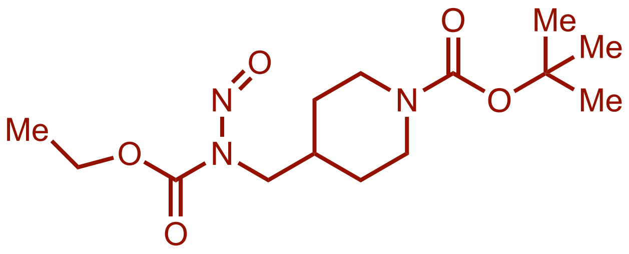


**SI-10**

***tert*-butyl 4-(((ethoxycarbonyl)(nitroso)amino)methyl)piperidine-1-carboxylate:** This compound was synthesized according to general procedure **1B** using 4-(Aminomethyl)piperidine, N1-BOC protected (1.07 g, 1.06 mL, 1 Eq, 5.00 mmol) to afford **SI-10** as a yellow solid (820 mg, 2.60 mmol, 52.0 % over two steps ) **R$_f$**: 0.5 in 25% EA/Hex.

**$^1$H NMR (500 MHz, CDCl$_3$)** δ 4.55 (q, *J* = 7.2 Hz, 2H), 4.06 (s, 2H), 3.66 (d, *J* = 7.2 Hz, 2H), 2.60 (t, *J* = 12.7 Hz, 2H), 1.67 (dtq, *J* = 15.1, 7.4, 3.5 Hz, 1H), 1.45 (d, *J* = 12.7 Hz, 14H), 1.10 (qd, *J* = 12.2, 4.3 Hz, 2H).

**$^{13}$C NMR (126 MHz, CDCl$_3$)** δ 154.8, 154.4, 79.6, 64.7, 45.8, 43.4, 34.9, 29.8, 28.5, 14.4.

**IR (neat):** 2978 (w), 2932 (w), 1746 (m), 1690 (s), 1511 (m), 1421 (m), 1134 (s).

**HRMS (ESI):** Calculated for $C_{14}$ $H_{25}$ $O_5$ $N_3$ Na; $[M+Na]^+$ 338.1686; found 338.1689.

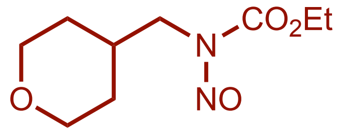

**SI-11**

**ethyl nitroso((tetrahydro-2*H*-pyran-4-yl)methyl)carbamate:** This compound was synthesized according to general procedure **1A** using tetrahydro-2H-pyran-4-ylmethylamine (576 mg, 596 µL, 1 Eq, 5.00 mmol) to afford **SI-11** as a yellow oil (450 mg, 2.08 mmol, 41.6 % over two steps) **$R_f$**: 0.5 in 10% EA/Hex.

**$^1$H NMR (500 MHz, $CDCl_3$)** δ 4.55 (d, *J* = 7.1 Hz, 2H), 4.01 – 3.93 (m, 2H), 3.67 (d, *J* = 7.2 Hz, 2H), 3.39 – 3.27 (m, 2H), 1.78 (ttt, *J* = 11.3, 7.4, 3.9 Hz, 1H), 1.49 – 1.45 (m, 3H), 1.43 – 1.41 (m, 1H), 1.33 (td, *J* = 12.5, 4.5 Hz, 3H).

**$^{13}$C NMR (126 MHz, $CDCl_3$)** δ 154.4, 67.6, 64.8, 45.9, 33.7, 30.4, 14.4.

**IR (neat):** 2844 (w), 1747 (s), 1509 (m), 1338 (w).

**HRMS (ESI):** Calculated for $C_9$ $H_{16}$ $O_4$ $N_2$ Na; $[M+Na]^+$ 239.1002; found 239.1002.

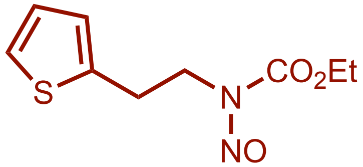

**SI-12**

**ethyl nitroso(2-(thiophen-2-yl)ethyl)carbamate:** This compound was synthesized according to general procedure **1A** using 2-Thiopheneethanamine (636 mg, 585 µL, 1 Eq, 5.00 mmol) to afford **SI-12** as a yellow oil (937 mg, 4.10 mmol, 82.1 % over two steps) **$R_f$**: 0.5 in 10% EA/Hex.

**$^1$H NMR (500 MHz, CDCl$_3$)** δ 7.15 (d, *J* = 5.1 Hz, 1H), 6.92 (dd, *J* = 5.1, 3.5 Hz, 1H), 6.79 (d, *J* = 3.4 Hz, 1H), 4.51 (q, *J* = 7.1 Hz, 2H), 4.06 – 3.95 (m, 2H), 2.91 (t, *J* = 7.5 Hz, 2H), 1.43 (t, *J* = 7.1 Hz, 3H).

**$^{13}$C NMR (126 MHz, CDCl$_3$)** δ 153.8, 139.5, 127.2, 125.9, 124.4, 64.7, 42.1, 27.2, 14.4.

**IR (neat):** 2984 (w), 1744 (s), 1507 (m), 1378 (m), 1134 (s).

**HRMS (ESI):** Calculated for $C_9 H_{12} O_3 N_2 Na S$ $[M+Na]^+$ 251.0461; found 251.0462.

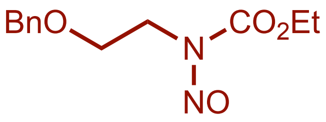

**SI-13**

**ethyl (2-(benzyloxy)ethyl)(nitroso)carbamate:** This compound was synthesized according to general procedure **1B** using 2-(Phenylmethoxy)ethylamine (756 mg, 744 µL, 1 Eq, 5.00 mmol) to afford **SI-13** as a yellow oil (1.096 g, 4.345 mmol, 86.9 % over two steps) **R$_f$**: 0.5 in 10% EA/Hex.

**$^1$H NMR (500 MHz, CDCl$_3$)** δ 7.32 (t, *J* = 7.1 Hz, 2H), 7.30 – 7.21 (m, 3H), 4.52 (q, *J* = 7.2 Hz, 2H), 4.45 (s, 2H), 4.03 (t, *J* = 5.9 Hz, 2H), 3.46 (t, *J* = 5.9 Hz, 2H), 1.43 (t, *J* = 7.1 Hz, 3H).

**$^{13}$C NMR (126 MHz, CDCl$_3$)** δ 154.1, 137.8, 128.5, 127.9, 127.7, 72.9, 65.8, 64.6, 40.1, 14.4.

**IR (neat):** 2900 (w), 2869 (w), 1747 (s), 1511 (s), 1378 (s), 1128 (s).

**HRMS (ESI):** Calculated for $C_{12} H_{16} O_4 N_2 Na$ $[M+Na]^+$ 275.1002; found 275.1002.

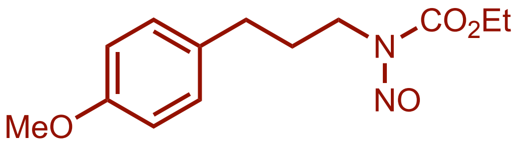

**SI-14**

**ethyl (3-(4-methoxyphenyl)propyl)(nitroso)carbamate:** This compound was synthesized according to general procedure **1B** 4-methoxy- benzenepropanamine, (330 mg, 1 Eq, 2.00 mmol) to afford **SI-14** a yellow ethyl (3-(4-methoxyphenyl)propyl)(nitroso)carbamate (450 mg, 1.69 mmol, 84.5 %)**$R_f$**: 0.5 in 10% EA/Hex.

**$^1$H NMR (500 MHz, $CDCl_3$)** δ 6.99 (d, *J* = 8.2 Hz, 2H), 6.75 (d, *J* = 8.4 Hz, 2H), 4.46 (q, *J* = 7.1 Hz, 2H), 3.70 (m, range, 5H), 2.43 (t, *J* = 7.8 Hz, 2H), 1.62 (p, *J* = 7.6 Hz, 2H), 1.38 (t, *J* = 7.1 Hz, 3H).

**$^{13}$C NMR (126 MHz, $CDCl_3$)** δ 158.1, 154.1, 132.8, 129.3, 114.0, 64.6, 55.4, 40.7, 32.3, 28.6, 14.4.

**IR (neat):** 2936 (w), 2836 (w), 1745 (s), 1511 (s), 1612 (m), 1464 (m).

**HRMS (ESI):** Calculated for $C_{13}H_{18}O_4N_2Na$; $[M+Na]^+$ 289.1159; found 289.1160.

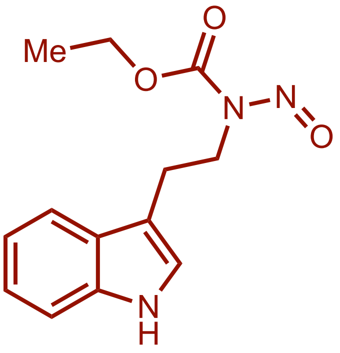


**SI-15**

**ethyl (2-(1*H*-indol-3-yl)ethyl)(nitroso)carbamate:** This compound was synthesized according to general procedure **1B** using 2-(1H-indol-3-yl)ethan-1-amine (100 mg, 1 Eq., 0.624 mmol) to afford ethyl (2-(1H-indol-3-yl)ethyl)(nitroso)carbamate **SI-15** (70 mg, 0.27 mmol, 43 %) as a yellow semi-solid. **$R_f$**: 0.5 in 30 % EA/Hex. *The compound is unstable.*

$^{1}$**H NMR (600 MHz, $CDCl_3$)** δ 7.41 (d, *J* = 8.0 Hz, 1H), 6.88 – 6.76 (m, 1H), 6.72 (t, *J* = 7.7 Hz, 1H), 6.65 (dd, *J* = 8.6, 6.1 Hz, 2H), 4.06 (s, 1H), 3.37 (dt, *J* = 14.3, 7.2 Hz, 2H), 2.87 – 2.71 (m, 2H), 2.26 – 2.06 (m, 2H), 0.49 (q, *J* = 7.0 Hz, 3H).

$^{13}$**C NMR (126 MHz, $CDCl_3$)** δ 156.7, 136.1, 129.6, 126.8, 125.9, 120.1, 116, 112.6, 111.7, 61.1, 40.0, 26.0, 14.7.

**IR (neat):** 3333 (br), 2981 (w), 1694 (s), 1525 (m), 1443 (m), 1249 (s), 1116 (s).

**HRMS (ESI):** Calculated for $C_{13} H_{15} O_2 N_2$ $[M\text{-}NO]^+$ 231.1128; found 231.1125.

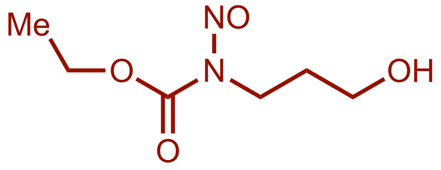


**SI-16**

**ethyl (3-hydroxypropyl)(nitroso)carbamate:** This compound was synthesized according to general procedure **1B** using 2-(1H-indol-3-yl)ethan-1-amine (100 mg, 1 Eq., 0.624 mmol) to afford ethyl (2-(1H-indol-3-yl)ethyl)(nitroso)carbamate **SI-16** (480 mg, 2.72 mmol, 68.1 %)as a yellow semi-solid. The carbamate was prepared in water using 1.5 Eq. of ethyl chloroformate and 1.6 Eq. of Potassium carbonate as the base.[2]

**$R_f$**: 0.5 in 50 % EA/Hex.

$^{1}$**H NMR (500 MHz, $CDCl_3$)** δ 4.55 (d, *J* = 7.1 Hz, 2H), 3.88 (t, *J* = 6.7 Hz, 2H), 3.50 (t, *J* = 5.9 Hz, 2H), 1.64 (p, *J* = 6.3 Hz, 2H), 1.46 (t, *J* = 7.1 Hz, 3H).

$^{13}$**C NMR (126 MHz, $CDCl_3$)** δ 154.5, 64.9, 59.5, 38.0, 30.1, 14.4.

**IR (neat):** 3395 (br), 2960 (w), 1745 (s), 1507 (m), 1379 (s), 1139 (s).

**HRMS (ESI):** Calculated for $C_6 H_{12} O_4 N_2 Na$ $[M\text{-}NO]^+$ 199.0689; found 199.0689.

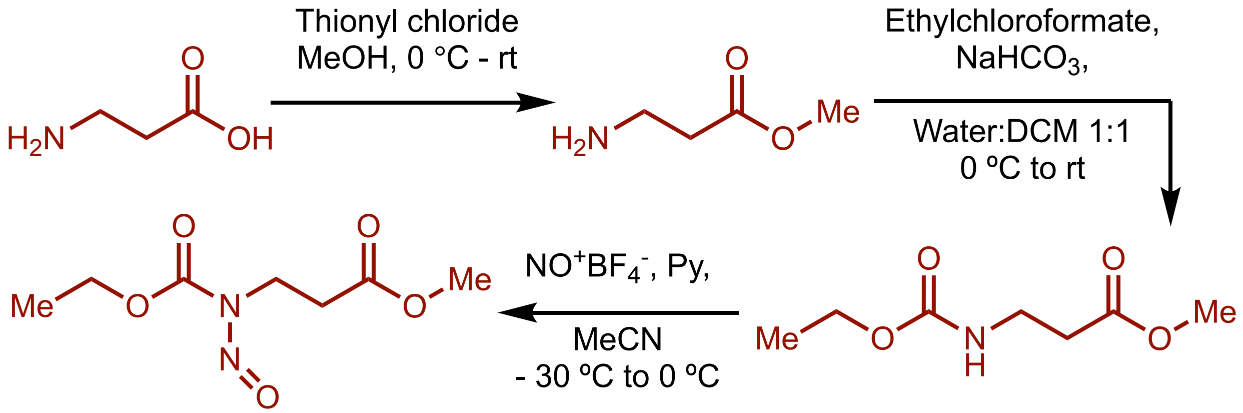


**SI-17**

**methyl 3-((ethoxycarbonyl)(nitroso)amino)propanoate:** To an ovendried 100 ml round bottom flask was taken 3-Amino-propionic acid (500 mg, 348 µL, 1 Eq, 5.61 mmol) and dissolved in 30 ml methanol. The mixture was cooled to 0 °C and Thionyl chloride (1.67 g, 1.03 mL, 2.5 Eq, 14.0 mmol) was added dropwise. The reaction mixture was warmed to room temperature and stirred overnight. After that methanol was evaporated and the crude was directly used in the next reaction.

The crude mixture was dissolved in 1:1 water/ $CH_2Cl_2$ (0.3 molar, 17 ml) and sodium bicarbonate (1.882 g, 4 Eq, 22.40 mmol) was added to it. The reaction mixture was cooled to 0 °C and ethyl chloroformate (729.3 mg, 641.4 µL, 1.2 Eq, 6.720 mmol) was added dropwise and the reaction was stirred overnight.

Next, the nitroso was synthesized according to general procedure 1B to afford **SI-17** as a yellow liquid (425 mg, 2.08 mmol, 37.2 % over three steps). **$R_f$**: 0.5 in 10% EA/Hex.

**$^1$H NMR (500 MHz, $CDCl_3$)** δ 4.55 (q, $J$ = 7.1 Hz, 2H), 4.03 (t, $J$ = 7.4 Hz, 2H), 3.67 (s, 3H), 2.42 (t, $J$ = 7.4 Hz, 2H), 1.46 (t, $J$ = 7.1 Hz, 3H).

**$^{13}$C NMR (126 MHz, $CDCl_3$)** δ 170.9, 153.7, 64.8, 52.1, 36.5, 31.4, 14.4.

**IR (neat):** 2956 (w), 1736 (s), 1510 (m), 1376 (m), 1141 (s).

**HRMS (ESI):** Calculated for $C_7 H_{12} O_5 N_2 Na$ $[M+Na]^+$ 227.0638; found 227.0637.

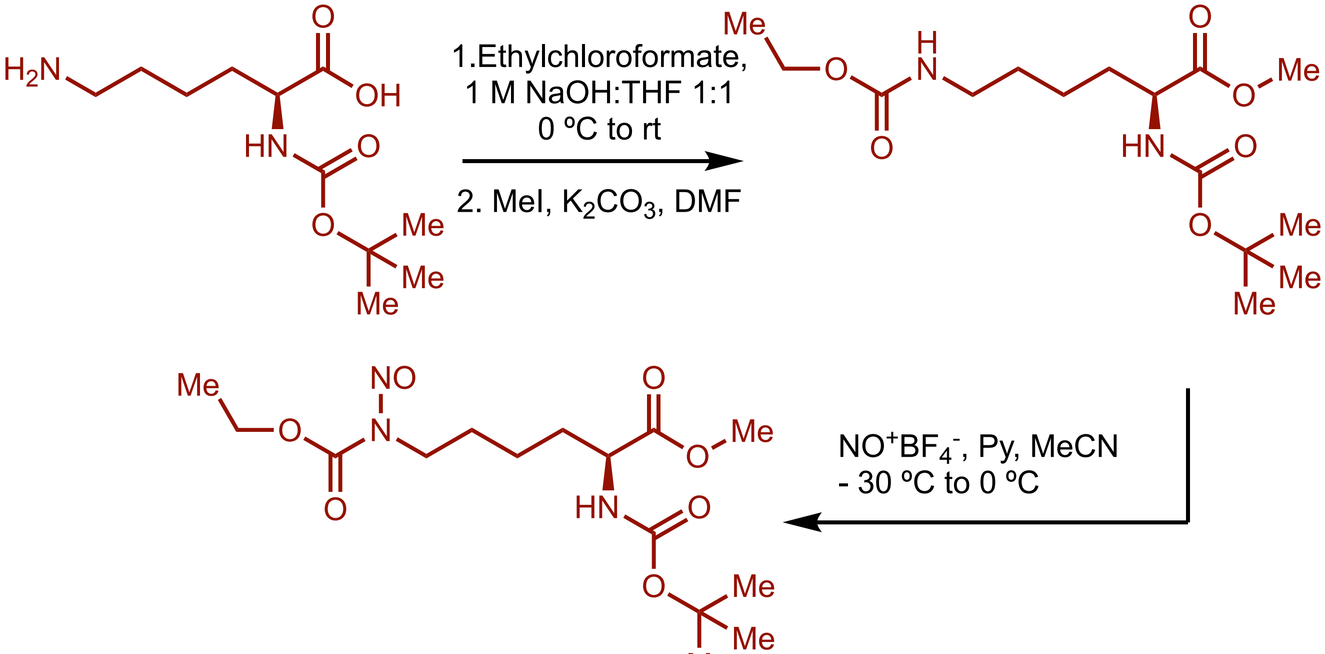


**SI-18**

**methyl** ***N*²-(*tert*-butoxycarbonyl)-*N*⁶-(ethoxycarbonyl)-*N*⁶-nitroso-*L*-lysinate:** In a reaction tube (tert-butoxycarbonyl)-L-lysine (1400 mg, 18.95 mL, 0.3 molar, 1 Eq, 5.684 mmol) was taken and dissolved in 1:1 THF/1 M NaOH and cooled down to 0 °C. To this ice-cold mixture ethyl chloroformate was added (678.5 mg, 596.7 µL, 1.1 Eq, 6.252 mmol) dropwise over 10 mins and the solution was stirred overnight at room temperature. Next, ethyl acetate was added and the aqueous phase was acidified with Conc. HCl to pH 1.5. The aqueous phase was extracted with ethyl acetate, dried over $Na_2SO_4$ and directly used in the next step.

Next, the crude material was dissolved in DMF (0.3 M, 19 ml) and potassium hydrogen carbonate (1.42 g, 2.5 Eq, 14.2 mmol) was added followed by methyl iodide (1.21 g, 551 µL, 1.5 Eq, 8.52 mmol) and the mixture was stirred for overnight. Following the reaction

was quenched with water and the aqueous layer was extracted in diethylether (3 times), dried over $Na_2SO_4$, filtered and evaporated under vacuum. The crude was purified in column chromatography to afford methyl N2-(tert-butoxycarbonyl)-N6-(ethoxycarbonyl)lysinate (1.32 g, 3.97 mmol, 69.9 %).

Finally the nitroso-carbamate was synthesized according to general procedure **1B** using methyl N2-(tert-butoxycarbonyl)-N6-(ethoxycarbonyl)-L-lysinate (150 mg, 1 Eq., 451 μmol) to afford **SI-18** a yellow liquid (88 mg, 0.24 mmol, 54 %). **$R_f$**: 0.5 in 20% EA/Hex.

**$^1H$ NMR (500 MHz, $CDCl_3$)** δ 5.00 (d, *J* = 8.4 Hz, 1H), 4.54 (q, *J* = 7.1 Hz, 2H), 4.31 – 4.21 (m, 1H), 3.73 (d, *J* = 3.9 Hz, 4H), 1.77 (dq, *J* = 16.2, 5.5 Hz, 1H), 1.59 (ddd, *J* = 13.8, 6.7, 3.9 Hz, 1H), 1.45 (d, *J* = 10.3 Hz, 14H), 1.31 – 1.22 (m, 3 H).

**$^{13}C$ NMR (126 MHz, $CDCl_3$)** δ 173.2, 155.5, 154.1, 80.1, 64.6, 53.3, 52.4, 40.5, 32.3, 28.4, 28.1, 28.1, 26.7, 22.7, 14.4.

**IR (neat):** 3669 (br), 2953 (w), 1744 (s), 1513 (s), 1368 (m), 1142 (s).

**HRMS (ESI):** Calculated for $C_{15} H_{27} O_7 N_3 Na$ $[M+Na]^+$ 384.1741; found 384.1744.

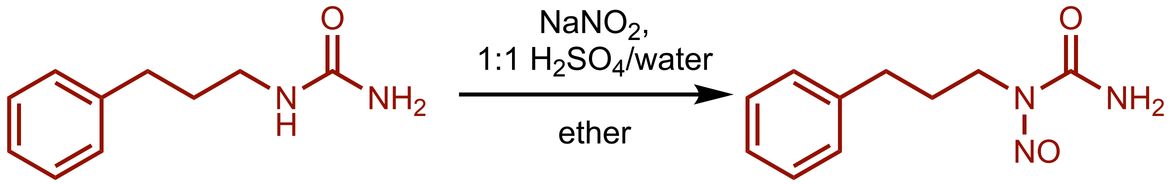


**SI-19**

**1-nitroso-1-(3-phenylpropyl) urea:** In a 20 dram vial 1-(3-phenylpropyl)urea[3] (400 mg, 1.00 Eq., 2.24 mmol) was dissolved in diethyl ether (1 M) and water (1 M) and the mixture was placed in an ice-water bath to keep the temperature below 15 °C. To this mixture

sodium nitrite (309 mg, 2 Eq., 4.48 mmol) was added followed by a dropwise addition of 10 ml 1:1 $H_2SO_4$/water mixture to maintain a green colour of the solution. The mixture was stirred at this temperature for 2-3 hrs and disappearance of the starting material was monitored via thin layer chromatography. Next, the reaction mixture was extracted with diethyl ether (2 times, 10 ml), and the combined organic layer was dried over $Na_2SO_4$, filtered, and evaporated till dryness. Next, the crude mixture was dissolved in 10% ethyl acetate/hexane and passed through a pad of silica to afford **SI-19** (150 mg, 724 µmol, 32.3 %)

**$^1$H NMR (500 MHz, $CDCl_3$)** δ 7.28 – 7.16 (m, 2H), 7.10 (dd, *J* = 17.4, 7.5 Hz, 3H), 6.76 (s, 1H), 5.44 (s, 1H), 3.79 (t, *J* = 7.5 Hz, 2H), 2.50 (t, *J* = 7.9 Hz, 2H), 1.69 (p, *J* = 7.7 Hz, 2H).

**$^{13}$C NMR (126 MHz, $CDCl_3$)** δ 154.5, 140.8, 128.6, 128.4, 126.2, 39.3, 33.3, 28.4.

**IR (neat):** 3384 (w), 3342 (w), 2948 (w), 1732 (s), 1602 (m), 1480 (m), 1334 (m), 1192 (m).

## V. General procedure 2- Cross-coupling reaction:

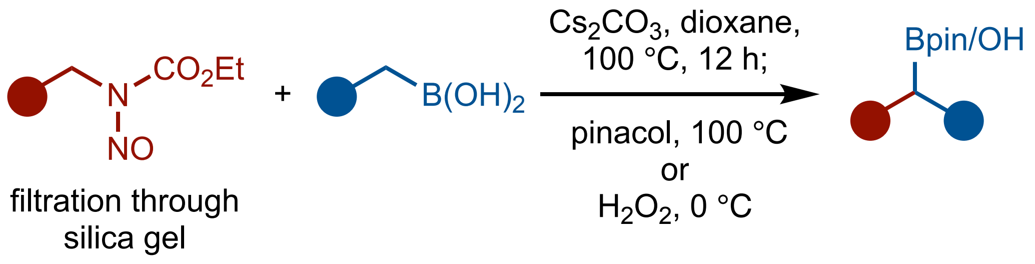


A reaction tube equipped with a stir bar was charged with nitrosocarbamate (1 Eq.), caesium carbonate (3 Eq.), and boronic acid (3 Eq.). The tube was closed with a septum and evacuated/backfilled with nitrogen. Next dioxane (0.1 M) was added to the tube and

the septum was replaced with a screw cap and placed in a metal block heated to 100 °C. The reaction mixture was stirred at that temperature for 12 hrs.

**A) Work up:** The crude reaction was cooled to room temperature, and pinacol (5 Eq.) was added and placed at 100 °C for 1 hr. After that, the reaction was cooled to room temperature, diluted with ethyl acetate, and passed through a pad of celite. The filtrate was evaporated to dryness and purified via flash column chromatography.

**B) Work up:** The crude reaction was cooled to 0 °C, and $H_2O_2$ (0.5 ml) was added and stirred at the same temperature. After 30 mins, the reaction was warm to room temperature, and the aqueous layer was extracted with ethyl acetate (3 times). The combined organic layers were dried over $Na_2SO_4$ and filtered. The filtrate was evaporated to dryness and purified via flash column chromatography.

**Substrate Scope:**

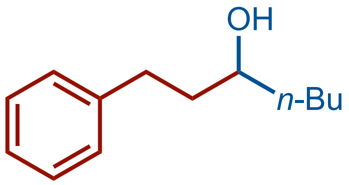


**44**

**1-phenylheptan-3-ol:** This compound was synthesized according to general procedure **2B** using ethyl nitroso(3-phenylpropyl)carbamate (24 mg, 1 Eq, 0.10 mmol) and butyl boronic acid (30.5 mg, 3 Eq, 0.30 mmol) to afford **44** as a clear liquid (15 mg, 78 µmol, 77 %) **$R_f$**: 0.5 in 20% EA/Hex. Spectral data matched with the literature report.[4]

**$^{1}$H NMR (500 MHz, $CDCl_3$)** δ 7.20 (q, *J* = 7.1 Hz, 2H), 7.12 (dd, *J* = 14.8, 7.5 Hz, 3H), 3.55 (tt, *J* = 8.2, 4.5 Hz, 1H), 2.78 – 2.70 (m, 1H), 2.60 (ddd, *J* = 13.8, 9.8, 6.6 Hz, 1H), 1.79 – 1.59 (m, 2H), 1.48 – 1.16 (m, 6H), 0.83 (t, *J* = 6.8 Hz, 3H).

**$^{13}$C NMR (126 MHz, $CDCl_3$)** δ 142.4, 128.6, 128.5, 125.9, 71.6, 39.2, 37.4, 32.2, 27.9, 22.9, 14.2.

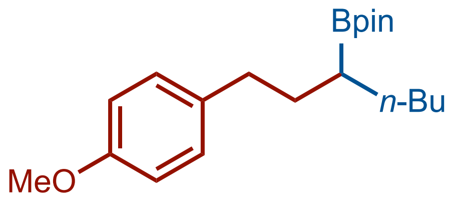


**6**

**2-(1-(4-methoxyphenyl)heptan-3-yl)-4,4,5,5-tetramethyl-1,3,2-dioxaborolane**: This compound was synthesized according to general procedure **2A** using ethyl (3-(4-methoxyphenyl)propyl)(nitroso)carbamate (53.3 mg, 1 Eq, 0.200 mmol) and butylboronic acid (61 mg, 3 Eq, 0.60 mmol) to afford **6** as a clear liquid (41 mg, 0.12 mmol, 62 %) **$R_f$**: 0.5 in 15 % EA/Hex. Spectral data matched with the literature report.[5]

**$^{1}$H NMR (500 MHz, $CDCl_3$)** δ 7.10 (d, *J* = 8.1 Hz, 2H), 6.82 (d, *J* = 8.7 Hz, 2H), 3.78 (s, 3H), 2.55 (tt, *J* = 18.0, 6.5 Hz, 2H), 1.71 (tq, *J* = 9.5, 4.6 Hz, 1H), 1.67 – 1.56 (m, 1H), 1.51 – 1.36 (m, 2H), 1.33 – 1.27 (m, 16H), 1.03 (td, *J* = 8.8, 4.4 Hz, 1H), 0.88 (t, *J* = 6.9 Hz, 3H).

**$^{13}$C NMR (126 MHz, $CDCl_3$)** δ 157.7, 135.4, 129.4, 113.8, 83.0, 55.4, 34.9, 33.9, 31.6, 31.1, 25.0, 25.0, 23.1, 14.2. (Signal of carbon directly bonded to boron was not detected because of quadrupolar relaxation)

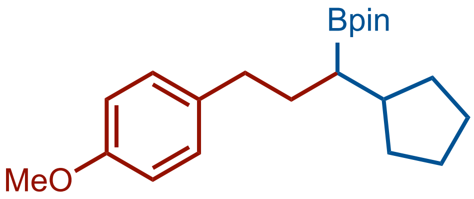

**7**

**2-(1-cyclopentyl-3-(4-methoxyphenyl)propyl)-4,4,5,5-tetramethyl-1,3,2-dioxaborolane:** This compound was synthesized according to general procedure **2A** using ethyl (3-(4-methoxyphenyl)propyl)(nitroso)carbamate (53.3 mg, 1 Eq., 0.200 mmol) and cyclopentylboronic acid (68.4 mg, 3 Eq., 600 μmol) to afford **7** as a colourless oil (36 mg, 0.10 mmol, 52 %). **$R_f$**: 0.5 in 10% EA/Hex.

**$^1$H NMR (500 MHz, $CDCl_3$)** δ 7.02 (d, J = 8.3 Hz, 2H), 6.74 (d, J = 8.2 Hz, 2H), 3.70 (s, 3H), 2.57 – 2.46 (m, 1H), 2.42 – 2.33 (m, 1H), 1.76 (dt, J = 14.3, 7.2 Hz, 2H), 1.71 – 1.58 (m, 3H), 1.58 – 1.46 (m, 2H), 1.46 – 1.35 (m, 2H), 1.20 (s, 12H), 1.11 – 0.95 (m, 2H), 0.86 (q, J = 8.4 Hz, 1H).

**$^{13}$C NMR (126 MHz, $CDCl_3$)** δ 157.7, 135.5, 129.3, 113.8, 83.0, 55.4, 42.0, 35.3, 33.4, 32.6, 32.23, 25.4, 25.2, 25.1, 25.0. (Signal of carbon directly bonded to boron was not detected because of quadrupolar relaxation)

**IR (neat):** 2931 (m), 2860 (w), 1610 (m), 1511 (m), 1243 (s), 1143 (s) $cm^{-1}$.

**HRMS (ESI):** Calculated for $C_{21}H_{33}O_3BNa$ $[M+Na]^+$ 367.2415; found 367.2416.

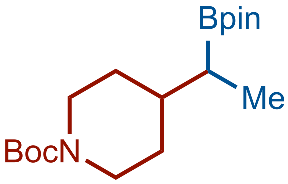

**8**

**tert-butyl 4-(1-(4,4,5,5-tetramethyl-1,3,2-dioxaborolan-2-yl)ethyl)piperidine-1-carboxylate:** This compound was synthesized according to general procedure **2A** using tert-butyl 4-(((ethoxycarbonyl)(nitroso)amino)methyl)piperidine-1-carboxylate (63.1 mg, 1 Eq, 0.200 mmol) and methylboronic acid (35.9 mg, 3 Eq, 600 μmol) to afford **8** as a white solid (41 mg, 0.12 mmol, 62 %) **$R_f$**: 0.5 in 15% EA/Hex. Spectral data matched with the literature report.[6]

**$^1$H NMR (500 MHz, $CDCl_3$)** δ 4.06 (s, 2H), 2.64 (s, 2H), 1.70 – 1.52 (m, 2H), 1.43 (s, 10H), 1.22 (s, 14H), 0.99 – 0.84 (m, 4H).

**$^{13}$C NMR (126 MHz, $CDCl_3$)** δ 155.06, 83.06, 79.16, 44.76, 38.89, 31.54, 30.93, 28.60, 24.90, 24.87, 12.56. (Signal of carbon directly bonded to boron was not detected because of quadrupolar relaxation)

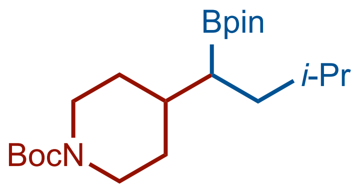


**9**

**tert-butyl 4-(3-methyl-1-(4,4,5,5-tetramethyl-1,3,2-dioxaborolan-2-yl)butyl)piperidine-1-carboxylate:** This compound was synthesized according to general procedure **2A** using tert-butyl 4-(((ethoxycarbonyl)(nitroso)amino)methyl)piperidine-1-carboxylate (63.1 mg, 1 Eq, 0.200 mmol) and isobutylboronic acid (61.2 mg, 3 Eq, 600 μmol) to afford **9** as a clear liquid (51 mg, 0.13 mmol, 67 %). **$R_f$**: 0.6 in 20% EA/Hex.

**$^1$H NMR (500 MHz, CDCl$_3$)** δ 4.01 (s, 2H), 2.56 (s, 2H), 1.66 – 1.48 (m, 2H), 1.37 (s, 11H), 1.35 – 1.27 (m, 1H), 1.17 (s, 12H), 1.13 – 1.00 (m, 2H), 0.91 (dt, J = 11.2, 5.7 Hz, 1H), 0.79 (dd, J = 11.1, 6.5 Hz, 6H).

**$^{13}$C NMR (126 MHz, CDCl$_3$)** δ 155.0, 83.1, 79.2, 44.3, 38.6, 38.2, 32.0, 31.2, 28.6, 27.9, 25.1, 25.0, 23.8, 22.2.

**IR (neat):** 2931 (w), 1695 (s), 1365 (m), 1167 (s) cm$^{-1}$.

**HRMS (ESI):** Calculated for $C_{21}$ $H_{40}$ $O_4$ N B Na; $[M+Na]^+$ 404.2943; found 404.2939.

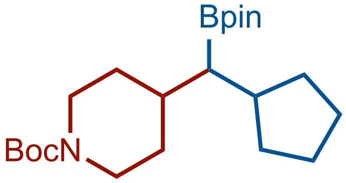


**10**

**tert-butyl 4-(cyclopentyl(4,4,5,5-tetramethyl-1,3,2-dioxaborolan-2-yl)methyl)piperidine-1-carboxylate**: This compound was synthesized according to general procedure **2A** using tert-butyl 4-(((ethoxycarbonyl)(nitroso)amino)methyl)piperidine-1-carboxylate (63.1 mg, 1 Eq, 0.200 mmol) (53.3 mg, 1 Eq, 0.200 mmol) and cyclopentylboronic acid (68.4 mg, 3 Eq, 600 μmol) to afford **10** as a white solid (66 mg, 0.17 mmol, 84 %)**$R_f$**: 0.6 in 20% EA/Hex. Spectral data matched with the literature report. [6]

**$^1$H NMR (500 MHz, CDCl$_3$)** δ 4.01 (br s, 2H), 2.55 (br s, 2H), 1.89 (dt, *J* = 10.0, 7.3 Hz, 1H), 1.79 – 1.65 (m, 2H), 1.65 – 1.39 (m, 7H), 1.37 (s, 9H), 1.17 (s, 14H), 1.00 (dtt, *J* = 12.5, 9.0, 4.9 Hz, 2H), 0.81 (dd, *J* = 9.9, 5.3 Hz, 1H).

**$^{13}$C NMR (126 MHz, $CDCl_3$)** δ 155.1, 83.1, 79.1, 45.2, 38.9, 37.4, 33.0, 32.7, 32.2, 30.3, 28.6, 25.2, 25.1, 25.1, 25.0. (Signal of carbon directly bonded to boron was not detected because of quadrupolar relaxation)

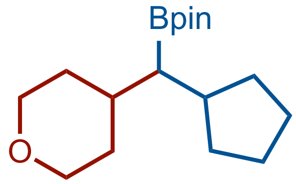


**11**

**2-(cyclopentyl(tetrahydro-2H-pyran-4-yl)methyl)-4,4,5,5-tetramethyl-1,3,2-dioxaborolane:** This compound was synthesized according to general procedure **2A** using ethyl nitroso((tetrahydro-2H-pyran-4-yl)methyl)carbamate (44 mg, 1 Eq., 0.20 mmol) and cyclopentylboronic acid (70 mg, 3 Eq., 0.61 mmol) to afford **11** as a yellowish liquid (27 mg, 92 µmol, 45 %) **$R_f$**: 0.4 in 50% EA/Hex.

**$^1$H NMR (500 MHz, $CDCl_3$)** δ 3.94 (dt, J = 10.5, 4.9 Hz, 2H), 3.34 (qd, J = 11.9, 2.2 Hz, 2H), 1.96 (dtd, J = 17.1, 9.8, 7.2 Hz, 1H), 1.84 – 1.68 (m, 3H), 1.64 – 1.53 (m, 4H), 1.53 – 1.34 (m, 4H), 1.25 (s, 12H), 1.14 – 1.00 (m, 2H), 0.88 (dd, J = 9.8, 5.8 Hz, 1H).

**$^{13}$C NMR (126 MHz, $CDCl_3$)** δ 83.1, 68.8, 68.7, 38.7, 36.4, 34.0, 32.7, 32.2, 31.4, 25.2, 25.2, 25.1, 25.1.

**IR (neat):** 2948 (m), 2838 (w), 1355 (m), 1142 (s) $cm^{-1}$.

**HRMS (ESI):** Calculated for $C_{17}H_{32}O_3B$; $[M+H]^+$ 295.2439; found 295.2440.

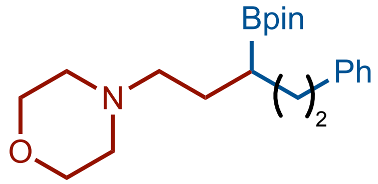


**12**

**4-(5-phenyl-3-(4,4,5,5-tetramethyl-1,3,2-dioxaborolan-2-yl)pentyl)morpholine:** This compound was synthesized according to general procedure **2A** using ethyl (3-morpholinopropyl)(nitroso)carbamate (49.06 mg, 1 Eq., 0.20 mmol) and phenethylboronic acid (89.99 mg, 3 Eq., 600.0 μmol) to afford **12** as a yellow liquid (35 mg, 97 μmol, 49 %) **$R_f$**: 0.4 in 70% EA/Hex.

**$^1$H NMR (500 MHz, $CDCl_3$)** δ 7.26 (t, J = 7.5 Hz, 2H), 7.17 (d, J = 7.7 Hz, 3H), 3.72 (t, J = 4.6 Hz, 4H), 2.60 (qt, J = 13.6, 7.2 Hz, 2H), 2.54 – 2.40 (m, 4H), 2.34 (tp, J = 11.8, 5.6 Hz, 2H), 1.76 (tdd, J = 15.0, 10.3, 5.8 Hz, 1H), 1.71 – 1.57 (m, 3H), 1.26 (s, 12H), 1.05 (dq, J = 9.0, 4.5 Hz, 1H).

**$^{13}$C NMR (126 MHz, $CDCl_3$)** δ 142.9, 128.5, 128.4, 125.8, 83.2, 67.0, 58.8, 53.9, 35.6, 33.5, 27.86, 25.0, 24.7.

**IR (neat):** 2926 (w), 2855 (w), 1454 (m), 1314 (m), 1143 (s) $cm^{-1}$.

**HRMS (ESI):** Calculated for $C_{21}H_{35}O_3NB$; $[M+H]^+$ 360.2705; found 360.2711.

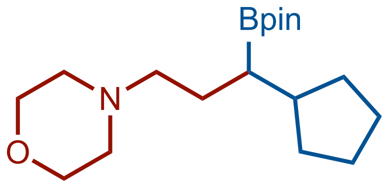


**13**

**4-(3-cyclopentyl-3-(4,4,5,5-tetramethyl-1,3,2-dioxaborolan-2-yl)propyl)morpholine:** This compound was synthesized according to general procedure **2A** using ethyl (3-morpholinopropyl)(nitroso)carbamate (49.06 mg, 1 Eq., 0.20 mmol) and cyclopentylboronic acid (68.37 mg, 3 Eq., 600.0 μmol) to afford **13** as a yellow liquid (60 mg, 0.19 mmol, 93 %) **$R_f$**: 0.4 in 70% EA/Hex.

**$^{1}$H NMR (500 MHz, CDCl$_3$)** δ 3.75 – 3.64 (m, 4H), 2.47 (dt, J = 10.1, 4.6 Hz, 2H), 2.39 (dq, J = 9.6, 4.7 Hz, 2H), 2.36 – 2.30 (m, 1H), 2.30 – 2.22 (m, 1H), 1.81 (dt, J = 12.7, 7.5 Hz, 2H), 1.72 (ddt, J = 12.6, 9.3, 4.2 Hz, 1H), 1.66 – 1.54 (m, 4H), 1.52 – 1.40 (m, 2H), 1.24 (s, 12H), 1.16 – 1.03 (m, 2H), 0.84 (td, J = 9.0, 5.8 Hz, 1H).

**$^{13}$C NMR (126 MHz, CDCl$_3$)** δ 83.1, 67.1, 59.3, 54.0, 42.0, 32.7, 32.2, 27.7, 25.4, 25.2, 25.1, 25.0. (Signal of carbon directly bonded to boron was not detected because of quadrupolar relaxation)

**IR (neat):** 2948 (m), 2856 (w), 1371 (m), 1145 (s) cm$^{-1}$.

**HRMS (ESI):** Calculated for $C_{18}H_{35}O_3NB$; [M+H]$^+$ 324.2705; found 324.2710.

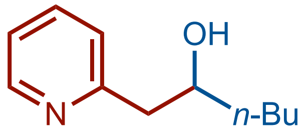


**14**

**1-(pyridin-2-yl)hexan-2-ol:** This compound was synthesized according to general procedure **2B** using ethyl nitroso ethyl nitroso(2-(pyridin-2-yl)ethyl)carbamate (45 mg, 1 Eq, 0.20 mmol) and butylboronic acid (62 mg, 3 Eq, 0.60 mmol) to afford **14** as a colourless liquid (31 mg, 0.17 mmol, 86 %) **R$_f$**: 0.5 in 40% EA/Hex. Spectral data matched with the literature report.[7]

**$^{1}$H NMR (500 MHz, CDCl$_3$)** δ 8.47 (dd, *J* = 5.1, 2.7 Hz, 1H), 7.60 (td, *J* = 7.7, 2.1 Hz, 1H), 7.13 (d, *J* = 7.7 Hz, 2H), 4.02 (qd, *J* = 5.7, 2.4 Hz, 1H), 2.96 – 2.88 (m, 1H), 2.82 (dd, *J* = 14.9, 8.9 Hz, 1H), 1.57 (dq, *J* = 17.8, 8.9 Hz, 1H), 1.53 – 1.42 (m, 2H), 1.42 – 1.27 (m, 3H), 0.96 – 0.86 (m, 3H). (alcoholic proton is not seen).

**$^{13}$C NMR (126 MHz, CDCl$_3$)** δ 160.5, 148.7, 136.8, 123.8, 121.6, 71.1, 43.4, 37.0, 28.0, 22.9, 14.2.

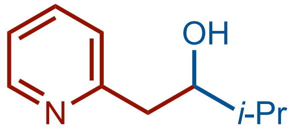

**15**

**3-methyl-1-(pyridin-2-yl)butan-2-ol:** This compound was synthesized according to general procedure **2B** using ethyl nitroso(2-(pyridin-2-yl)ethyl)carbamate (45 mg, 1 Eq, 0.20 mmol) and isopropylboronic acid (53 mg, 3 Eq, 0.60 mmol) to afford **15** as a colourless liquid (30 mg, 0.18 mmol, 90 %) $\mathbf{R_f}$: 0.5 in 40% EA/Hex. Spectral data matched with the literature report.[8]

**$^1$H NMR (500 MHz, CDCl$_3$)** δ 8.47 (d, $J$ = 4.9 Hz, 1H), 7.63 (t, $J$ = 7.7 Hz, 1H), 7.22 – 7.12 (m, 2H), 3.77 (ddd, $J$ = 8.9, 5.7, 2.6 Hz, 1H), 2.97 – 2.80 (m, 2H), 1.81 – 1.70 (m, $J$ = 6.7 Hz, 1H), 1.00 (d, $J$ = 6.8 Hz, 3H), 0.97 (d, $J$ = 6.8 Hz, 3H).(alcoholic proton is not seen).

**$^{13}$C NMR (126 MHz, CDCl$_3$)** δ 160.6, 148.5, 137.1, 124.0, 121.7, 76.3, 40.5, 33.6, 18.7, 18.1.

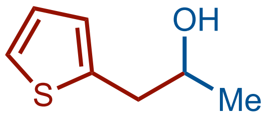

**16**

**1-(thiophen-2-yl)propan-2-ol:** This compound was synthesized according to general procedure **2B** using ethyl nitroso(2-(thiophen-2-yl)ethyl)carbamate (45 mg, 1 Eq., 0.20 mmol) and methylboronic acid (35 mg, 3 Eq., 0.59 mmol) to afford **16** as a yellowish oil (14 mg, 0.1 mmol, 50 %) $\mathbf{R_f}$: 0.3 in 10% EA/Hex. Spectral data matched with the literature report.[9]

**$^1$H NMR (500 MHz, CDCl$_3$)** δ 7.18 (dd, $J$ = 5.2, 1.2 Hz, 1H), 6.97 (dd, $J$ = 5.1, 3.4 Hz, 1H), 6.87 (dd, $J$ = 3.4, 1.1 Hz, 1H), 4.02 (dddq, $J$ = 8.0, 6.1, 3.8, 2.0 Hz, 1H), 3.01 (ddd, $J$ =

14.6, 4.4, 0.9 Hz, 1H), 2.91 (dd, *J* = 14.7, 7.9 Hz, 1H), 1.70 (d, *J* = 4.0 Hz, 1H), 1.27 (d, *J* = 6.3 Hz, 4H).

**$^{13}$C NMR (126 MHz, $CDCl_3$)** δ 140.66, 127.18, 126.17, 124.40, 68.82, 39.81, 22.70.

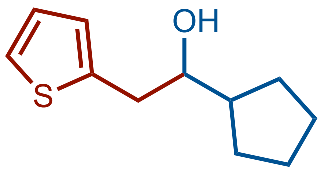


**17**

**1-cyclopentyl-2-(thiophen-2-yl)ethan-1-ol:** This compound was synthesized according to general procedure **2B** using ethyl nitroso(2-(thiophen-2-yl)ethyl)carbamate (45 mg, 1 Eq., 0.20 mmol) and cyclopentylboronic acid (68.37 mg, 3 Eq., 600.0 µmol) to afford **17** as a colourless oil (28 mg, 0.14 mmol, 72 %)**$R_f$**: 0.3 in 10% EA/Hex.

**$^1$H NMR (500 MHz, $CDCl_3$)** δ 7.17 (s, 1H), 6.96 (t, *J* = 4.3 Hz, 1H), 6.88 (d, *J* = 3.3 Hz, 1H), 3.62 (td, *J* = 8.1, 3.3 Hz, 1H), 3.09 (dd, *J* = 14.8, 3.3 Hz, 1H), 2.88 (dd, *J* = 14.8, 8.5 Hz, 1H), 1.94 (h, *J* = 8.2 Hz, 1H), 1.89 – 1.71 (m, 3H), 1.64 (dp, *J* = 11.4, 3.9 Hz, 2H), 1.57 (qd, *J* = 7.2, 4.1 Hz, 2H), 1.43 (dq, *J* = 12.1, 7.8 Hz, 1H), 1.31 (dt, *J* = 12.4, 7.9 Hz, 1H).

**$^{13}$C NMR (126 MHz, $CDCl_3$)** δ 141.0, 127.1, 126.1, 124.3, 76.6, 45.5, 37.0, 29.4, 28.8, 25.9, 25.8.

**IR (neat):** 3350 (br), 2949 (m), 2866 (w), 1248 (w), 1044 (m) cm$^{-1}$.

**GC (EI, m/z):** Calculated for $C_{11}H_{16}OS$ 196.0922; found [M-$C_6H_{11}O$+H] 98.0185.

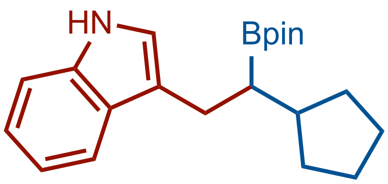


**18**

**3-(2-cyclopentyl-2-(4,4,5,5-tetramethyl-1,3,2-dioxaborolan-2-yl)ethyl)-1H-indole:** This compound was synthesized according to general procedure **2A** using ethyl (2-(1H-indol-3-yl)ethyl)(nitroso)carbamate (50 mg, 1 Eq., 0.19 mmol) and cyclopentylboronic acid (65 mg, 3 Eq., 0.57 mmol) to afford **18** as a yellowish liquid (27 mg, 80 µmol, 42 %) **$R_f$**: 0.5 in 30% EA/Hex. *The compound is unstable.*

**$^1$H NMR (500 MHz, CDCl$_3$)** δ 7.88 (s, 1H), 7.64 (d, *J* = 7.9 Hz, 1H), 7.31 (d, *J* = 8.0 Hz, 1H), 7.15 (t, *J* = 7.5 Hz, 1H), 7.09 (t, *J* = 7.4 Hz, 1H), 7.03 (d, *J* = 2.2 Hz, 1H), 2.95 – 2.81 (m, 2H), 1.97 (p, *J* = 7.9 Hz, 2H), 1.85 – 1.75 (m, 1H), 1.65 (dh, *J* = 8.0, 3.7 Hz, 2H), 1.53 (td, *J* = 7.5, 4.7 Hz, 2H), 1.50 – 1.41 (m, 1H), 1.27 (dp, *J* = 11.4, 3.2 Hz, 2H), 1.06 (d, *J* = 15.2 Hz, 12H).

**$^{13}$C NMR (126 MHz, CDCl$_3$)** δ 136.3, 128.0, 121.8, 121.5, 119.5, 119.0, 117.2, 110.8, 82.9, 42.4, 33.0, 32.0, 26.1, 25.5, 25.3, 24.9, 24.8.

**IR (neat):** 3347 (br), 2976 (w), 2946 (w), 1456 (m), 1372 (m), 1141 (s) cm$^{-1}$.

**HRMS (ESI):** Calculated for $C_{21}H_{30}O_2NBNa$; $[M+Na]^+$ 362.2262; found 362.2262.

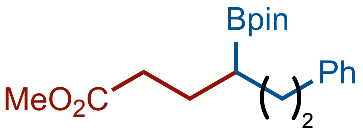


**19**

**methyl 5-phenyl-3-(4,4,5,5-tetramethyl-1,3,2-dioxaborolan-2-yl)pentanoate:** This compound was synthesized according to general procedure **2A** in absence of cesium carbonate using methyl 3-((ethoxycarbonyl)(nitroso)amino)propanoate (40.8 mg, 1 Eq, 0.200 mmol) and phenethyl boronic Acid (180 mg, 6 Eq, 1.20 mmol) to afford **19** as a colourless liquid (40 mg, 0.13 mmol, 63 %). **$R_f$**: 0.5 in 10% diethyl ether/Hex. Spectral data matched with the literature report.[10]

**$^1$H NMR (500 MHz, CDCl$_3$)** δ 7.25 (d, $J$ = 6.7 Hz, 2H), 7.17 (d, $J$ = 7.6 Hz, 3H), 3.65 (s, 3H), 2.64 (dt, $J$ = 9.8, 5.7 Hz, 2H), 2.47 (dd, $J$ = 7.6, 6.0 Hz, 2H), 1.85 – 1.74 (m, 1H), 1.64 (ddt, $J$ = 13.5, 9.9, 6.7 Hz, 1H), 1.43 – 1.36 (m, 1H), 1.26 (d, $J$ = 4.9 Hz, 12H).

**$^{13}$C NMR (126 MHz, CDCl$_3$)** δ 174.4, 142.7, 128.6, 128.4, 125.8, 83.4, 51.6, 35.7, 35.2, 32.8, 25.0, 24.9. (Signal of carbon directly bonded to boron was not detected because of quadrupolar relaxation)

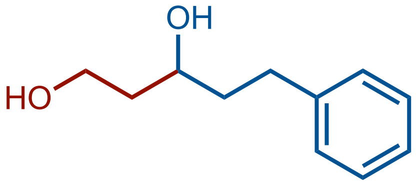


**20**

**5-phenylpentane-1,3-diol:** This compound was synthesized according to general procedure **2B** using ethyl (3-hydroxypropyl)(nitroso)carbamate (35.2 mg, 1 Eq, 0.200 mmol) and phenethylboronic acid (90.0 mg, 3 Eq, 600 μmol) to afford **20** as a colourless oil (20 mg, 0.11 mmol, 55 %). **R$_f$**: 0.3 in 80% EA/Hex. Spectral data matched with the literature report.[11]

**$^1$H NMR (500 MHz, CDCl$_3$)** δ 7.58 (q, $J$ = 7.6 Hz, 2H), 7.50 (dd, $J$ = 11.4, 7.2 Hz, 3H), 4.44 – 4.25 (m, 1H), 4.20 (dq, $J$ = 12.3, 4.6 Hz, 1H), 4.13 (ddd, $J$ = 11.1, 7.1, 4.9 Hz, 1H), 3.09 (ddd, $J$ = 15.0, 11.4, 6.6 Hz, 1H), 3.05 – 2.92 (m, 1H), 2.97 – 2.59 (m, 1H), 2.25 – 1.89 (m, 4H).

**$^{13}$C NMR (126 MHz, CDCl$_3$)** δ 142.0, 128.6, 128.5, 126.0, 71.7, 62.0, 39.5, 38.5, 32.0.

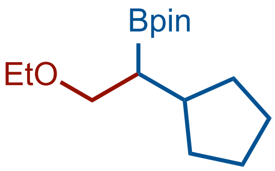

**21**

**2-(1-cyclopentyl-2-ethoxyethyl)-4,4,5,5-tetramethyl-1,3,2-dioxaborolane:** This compound was synthesized according to general procedure **2A** using ethyl (2-ethoxyethyl)(nitroso)carbamate (38 mg, 1 Eq., 0.20 mmol) and cyclopentylboronic acid (68 mg, 3 Eq., 0.60 mmol) to afford **21** as a yellowish liquid (43 mg, 0.16 mmol, 80 %) **$R_f$**: 0.5 in 10% diethyl ether/Hex.

**$^1$H NMR (500 MHz, $CDCl_3$)** δ 3.53 (dd, $J$ = 8.7, 6.7 Hz, 1H), 3.50 – 3.36 (m, 3H), 1.92 – 1.80 (m, 1H), 1.75 (dtd, $J$ = 14.3, 6.9, 3.1 Hz, 2H), 1.47 (pd, $J$ = 7.5, 3.8 Hz, 2H), 1.33 – 1.26 (m, 1H), 1.23 (s, 12H), 1.17 – 1.07 (m, 5H).

**$^{13}$C NMR (126 MHz, $CDCl_3$)** δ 83.0, 72.4, 66.2, 39.1, 32.8, 32.2, 25.2, 25.0, 24.9, 24.8, 15.3.

**IR (neat):** 2947 (w), 2864 (w), 1370 (m), 1140 (s) $cm^{-1}$.

**HRMS (ESI):** Calculated for $C_{15}$ $H_{29}$ $O_3$ B Na; $[M+Na]^+$ 291.2102; found 291.2103.

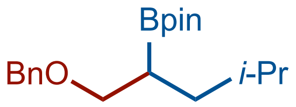


**22**

**2-(1-(benzyloxy)-4-methylpentan-2-yl)-4,4,5,5-tetramethyl-1,3,2-dioxaborolane:** This compound was synthesized according to general procedure **2A** using ethyl (2-(benzyloxy)ethyl)(nitroso)carbamate (50.45 mg, 1 Eq, 0.2000 mmol) and isobutylboronic acid (61.16 mg, 3 Eq, 600.0 µmol) to afford **22** as a colourless liquid (42 mg, 0.13 mmol, 66 %). **$R_f$**: 0.5 in 10% EA/Hex. Spectral data matched with the literature report.[12]

**$^1$H NMR (500 MHz, $CDCl_3$)** δ 7.31 – 7.14 (m, 5H), 4.42 (s, 2H), 3.44 (dd, $J$ = 7.3, 2.2 Hz, 2H), 1.41 (dt, $J$ = 12.8, 6.8 Hz, 2H), 1.30 (ddd, $J$ = 15.4, 9.1, 6.6 Hz, 2H), 1.16 (s, 12H), 0.81 (d, $J$ = 6.6 Hz, 6H).

**$^{13}$C NMR (126 MHz, CDCl$_3$)** δ 139.1, 128.3, 127.6, 127.4, 83.2, 72.9, 72.8, 37.3, 29.9, 27.6, 24.9(d), 23.2, 22.7.

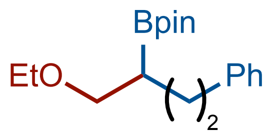


**SI-20**

**2-(1-ethoxy-4-phenylbutan-2-yl)-4,4,5,5-tetramethyl-1,3,2-dioxaborolane:** This compound was synthesized according to general procedure **2A** using ethyl ethyl (2-ethoxyethyl)(nitroso)carbamate (38 mg, 1 Eq., 0.20 mmol) and phenethylboronic acid (90 mg, 3 Eq., 0.60 mmol) to afford **SI-20** as a yellowish liquid (40 mg, 0.13 mmol, 66 %) **$R_f$**: 0.5 in 10% EA/Hex.

**$^1$H NMR (500 MHz, CDCl$_3$)** δ 7.26 (t, J = 7.5 Hz, 2H), 7.23 – 7.13 (m, 3H), 3.55 – 3.42 (m, 4H), 2.74 – 2.54 (m, 2H), 1.77 (ddt, J = 12.3, 10.0, 6.0 Hz, 2H), 1.50 – 1.40 (m, 1H), 1.26 (s, 12H), 1.17 (t, J = 7.0 Hz, 3H).

**$^{13}$C NMR (126 MHz, CDCl$_3$)** δ 143.0, 128.6, 128.3, 125.7, 83.2, 72.1, 66.1, 35.6, 30.2, 24.9, 24.86, 15.3.

**IR (neat):** 2976 (w), 2858 (w), 1454 (w), 1318 (s), 1107 (s) cm$^{-1}$.

**HRMS (ESI):** Calculated for $C_{18}$ $H_{29}$ $O_3$ B Na; $[M+Na]^+$ 327.2102; found 327.2103.

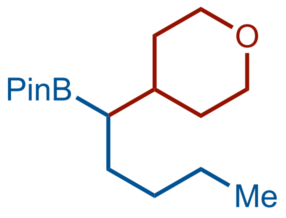


**SI-21**

**4,4,5,5-tetramethyl-2-(1-(tetrahydro-2H-pyran-4-yl)pentyl)-1,3,2-dioxaborolane:** This compound was synthesized according to general procedure **2A** using ethyl

nitroso((tetrahydro-2H-pyran-4-yl)methyl)carbamate (44 mg, 1 Eq., 0.20 mmol) and butylboronic acid (62 mg, 3 Eq., 0.61 mmol) to afford **SI-21** as a colourless liquid (33 mg, 0.12 mmol, 57 %) **R$_f$**: 0.4 in 50% EA/Hex.

**$^1$H NMR (500 MHz, CDCl$_3$)** δ 3.93 (dt, *J* = 10.9, 5.3 Hz, 2H), 3.34 (tt, *J* = 11.6, 2.3 Hz, 2H), 1.65 – 1.51 (m, 3H), 1.40 (h, *J* = 6.0 Hz, 3H), 1.24 (s, 17H), 0.86 (t, *J* = 7.1 Hz, 4H).

**$^{13}$C NMR (126 MHz, CDCl$_3$)** δ 83.0, 68.6, 68.4, 36.9, 32.8, 32.4, 31.7, 28.0, 25.0, 24.9, 23.0, 14.1. (Signal of carbon directly bonded to boron was not detected because of quadrupolar relaxation)

**IR (neat):** 2927 (m), 1372 (m), 1240 (m), 1145 (s) cm$^{-1}$.

**HRMS (ESI):** Calculated for $C_{16}$ $H_{32}$ $O_3$ B; [M+H]$^+$ 283.2439; found 283.2440.

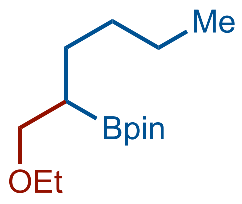


**SI-22**

**2-(1-ethoxyhexan-2-yl)-4,4,5,5-tetramethyl-1,3,2-dioxaborolane:** This compound was synthesized according to general procedure **2A** using ethyl (2-ethoxyethyl)(nitroso)carbamate (38 mg, 1 Eq., 0.20 mmol) and butylboronicacid (61 mg, 3 Eq., 0.60 mmol) to afford **SI-22** as a clear liquid (23 mg, 90 μmol, 45 %) **R$_f$**: 0.5 in 10% diethyl ether/Hex.

**$^1$H NMR (500 MHz, CDCl$_3$)** δ 3.49 – 3.41 (m, 4H), 1.45 – 1.34 (m, 3H), 1.34 – 1.27 (m, 4H), 1.24

(s, 12H), 1.16 (t, J = 7.0 Hz, 3H), 0.88 (t, J = 6.9 Hz, 3H).

**$^{13}$C NMR (126 MHz, CDCl$_3$)** δ 83.1, 72.4, 66.1, 31.6, 27.8, 24.9, 23.1, 15.3, 14.2. Signal of carbon directly bonded to boron was not detected because of quadrupolar relaxation)

**IR (neat):** 2976 (w), 2858 (w), 1371 (s), 1146 (s) cm$^{-1}$.

**HRMS (ESI):** Calculated for $C_{14}$ $H_{29}$ $O_3$ B Na; [M+Na]$^+$ 279.2102; found 279.2103.

## VI. General procedure 3- Cross-coupling with aryl boronic acid.

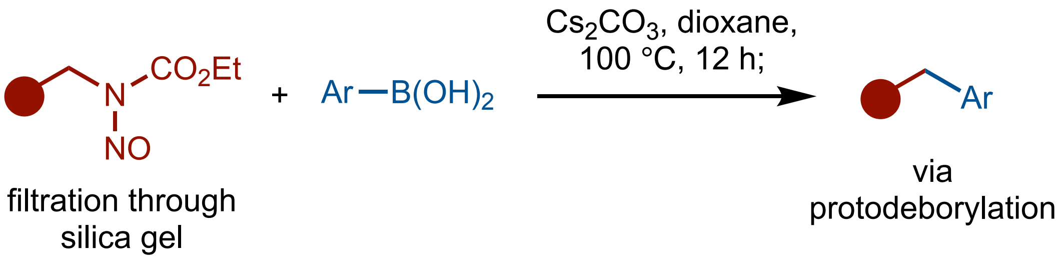


A reaction tube equipped with a stir bar was charged with nitrosocarbamate (1 Eq.), caesium carbonate (3 Eq.), and aryl boronic acid (3 Eq.). The tube was closed with a septum and evacuated/backfilled with nitrogen. Next dioxane (0.1 M) was added to the tube and the septum was replaced with a screw cap and placed in a metal block heated to 100 °C. The reaction mixture was stirred at that temperature for 16 hrs. After that, the reaction was cooled to room temperature, diluted with ethyl acetate, and passed through a pad of celite. The filtrate was evaporated to dryness and purified via flash column chromatography.

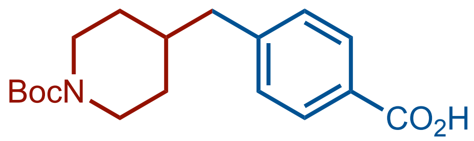


**24**

**4-((1-(tert-butoxycarbonyl)piperidin-4-yl)methyl)benzoic acid:** This compound was synthesized according to general procedure **3** using tert-butyl 4-(((ethoxycarbonyl)(nitroso)amino)methyl)piperidine-1-carboxylate (63.1 mg, 1 Eq., 0.200

mmol) and 4-carboxyphenylboronic acid (99.6 mg, 3 Eq., 600 µmol) to afford **24** as a clear liquid (34 mg, 0.11 mmol, 53 %) **$R_f$**: 0.2 in 90% ethyl acetate/hexane. Spectral data matched with the literature report.[13]

**$^1$H NMR (500 MHz, CDCl$_3$)** δ 8.03 (d, *J* = 7.9 Hz, 2H), 7.24 (d, *J* = 7.9 Hz, 2H), 4.08 (s, 3H), 2.74 – 2.52 (m, 4H), 1.70 (ddt, *J* = 11.4, 7.8, 3.9 Hz, 1H), 1.60 (d, *J* = 13.2 Hz, 2H), 1.45 (s, 9H), 1.16 (qd, *J* = 12.6, 4.2 Hz, 2H).

**$^{13}$C NMR (126 MHz, CDCl$_3$)** δ 171.8, 155.0, 146.9, 130.4, 129.4, 127.4, 79.6, 43.9, 43.3, 38.1, 32.1, 28.6.

**HRMS (ESI):** Calculated for $C_{18}$ $H_{25}$ $O_4$ N Na [M+Na]$^+$ 342.1676; found 342.1678.

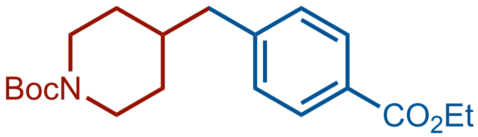


**25**

**tert-butyl 4-(4-(ethoxycarbonyl)benzyl)piperidine-1-carboxylate** : This compound was synthesized according to general procedure **3** using tert-butyl 4-(((ethoxycarbonyl)(nitroso)amino)methyl)piperidine-1-carboxylate (63.1 mg, 1 Eq., 0.200 mmol) and (4-(ethoxycarbonyl)phenyl)boronic acid (116 mg, 3 Eq., 600 µmol) to afford **25** as a clear liquid (34 mg, 98 µmol, 49 %)**$R_f$**: 0.5 in 20% ethyl acetate/hexane.

**$^1$H NMR (500 MHz, CDCl$_3$)** δ 7.95 (d, *J* = 8.2 Hz, 2H), 7.19 (d, *J* = 7.9 Hz, 2H), 4.36 (q, *J* = 7.1 Hz, 2H), 4.06 (s, 2H), 2.71 – 2.53 (m, 4H), 1.69 – 1.64 (m, 1H), 1.64 – 1.55 (m, 2H), 1.44 (s, 9H), 1.38 (t, *J* = 7.1 Hz, 3H), 1.14 (qd, *J* = 12.3, 4.2 Hz, 2H).

**$^{13}$C NMR (126 MHz, CDCl$_3$)** δ 166.7, 155.0, 145.8, 129.7, 129.2, 128.5, 79.4, 61.0, 43.0, 43.2, 38.2, 32.1, 28.6, 14.5.

**IR (neat):** 2977 (w), 2851 (w), 1715 (s), 1689 (s), 1417 (m), 1160 (s) cm$^{-1}$.

**HRMS (ESI):** Calculated for $C_{20}$ $H_{29}$ $O_4$ N Na $[M+Na]^+$ 370.1989; found 370.1991.

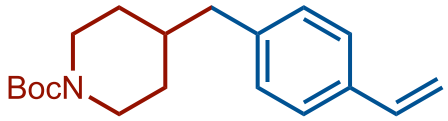

**26**

**tert-butyl 4-(4-vinylbenzyl)piperidine-1-carboxylate :** This compound was synthesized according to general procedure **3** using tert-butyl 4-(((ethoxycarbonyl)(nitroso)amino)methyl)piperidine-1-carboxylate (31.5 mg, 1 Eq., 0.100 mmol) and (4-vinylphenyl)boronic acid (44.4 mg, 3 Eq., 300 μmol) to afford **26** as a clear liquid (13 mg, 43 μmol, 43 %) $\mathbf{R_f}$: 0.5 in 20% ethyl acetate/hexane.

**$^1$H NMR (500 MHz, $CDCl_3$)** δ 7.26 (d, *J* = 7.7 Hz, 2H), 7.02 (d, *J* = 7.7 Hz, 2H), 6.62 (dd, *J* = 17.6, 10.9 Hz, 1H), 5.64 (d, *J* = 17.6 Hz, 1H), 5.13 (d, *J* = 10.9 Hz, 1H), 3.99 (s, 2H), 2.57 (d, *J* = 13.1 Hz, 2H), 2.45 (d, *J* = 6.8 Hz, 2H), 1.54 (d, *J* = 14.6 Hz, 3H), 1.38 (s, 9H), 1.12 – 1.02 (m, 2H).

**$^{13}$C NMR (126 MHz, $CDCl_3$)** δ 155.0, 140.1, 136.7, 135.6, 129.4, 126.3, 113.2, 79.4, 43.8, 43.0, 38.3, 32.1, 28.6.

**IR (neat):** 2922 (w), 2850 (w), 1690 (m), 1421 (m), 1162 (s) $cm^{-1}$.

**HRMS (ESI):** Calculated for $C_{19}$ $H_{27}$ $O_2$ N Na $[M+Na]^+$ 324.1934; found 324.1936.

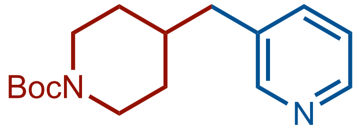

**27**

**tert-butyl 4-(pyridin-3-ylmethyl)piperidine-1-carboxylate:** This compound was synthesized according to general procedure **3** using tert-butyl 4-(((ethoxycarbonyl)(nitroso)amino)methyl)piperidine-1-carboxylate (31.5 mg, 1 Eq., 0.100

mmol) and pyridin-3-ylboronic acid (36.9 mg, 3 Eq., 300 μmol) to afford **27** as a clear liquid (6.5 mg, 24 μmol, 24 %) **R$_f$**: 0.2 in 90% ethyl acetate/hexane. Spectral data matched with literature report.[14]

**$^1$H NMR (500 MHz, CDCl$_3$)** δ 8.46 (s, 1H), 8.42 (s, 1H), 7.46 (d, *J* = 7.7 Hz, 1H), 7.22 (dd, *J* = 7.8, 4.8 Hz, 1H), 4.08 (s, 2H), 2.72 – 2.58 (m, 2H), 2.54 (d, *J* = 7.0 Hz, 2H), 1.72 – 1.57 (m, 3H), 1.45 (s, 9H), 0.98 – 0.78 (m, 2H).

**$^{13}$C NMR (126 MHz, CDCl$_3$)** δ 183.3, 155.0, 150.3, 147.5, 136.9, 123.5, 79.5, 40.3, 38.1, 32.0, 28.6.

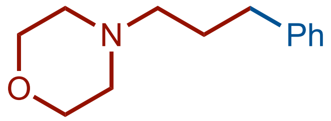


**28**

**4-(3-phenylpropyl)morpholine:** This compound was synthesized according to general procedure **3** using ethyl (3-morpholinopropyl)(nitroso)carbamate (24.53 mg, 1 Eq., 0.1000 mmol) and phenylboronic acid (36.58 mg, 3 Eq., 300.0 μmol) to afford **28** as a clear liquid (17 mg, 83 μmol, 83 %) **R$_f$**: 0.5 in 20% ethyl acetate/hexane. Spectral data matched with literature report.[15]

**$^1$H NMR (500 MHz, CDCl$_3$)** δ 7.24 – 7.15 (m, 2H), 7.15 – 7.06 (m, 3H), 3.65 (t, *J* = 4.7 Hz, 4H), 2.57 (t, *J* = 7.7 Hz, 2H), 2.36 (t, *J* = 4.7 Hz, 4H), 2.33 – 2.25 (m, 2H), 1.75 (p, *J* = 7.6 Hz, 2H).

**$^{13}$C NMR (126 MHz, CDCl$_3$)** δ 142.2, 128.5, 128.5, 125.9, 67.1, 58.5, 53.9, 33.8, 28.4.

## VII. General procedure 4: Cross-coupling with alkyl potassium trifluoroborate salt.

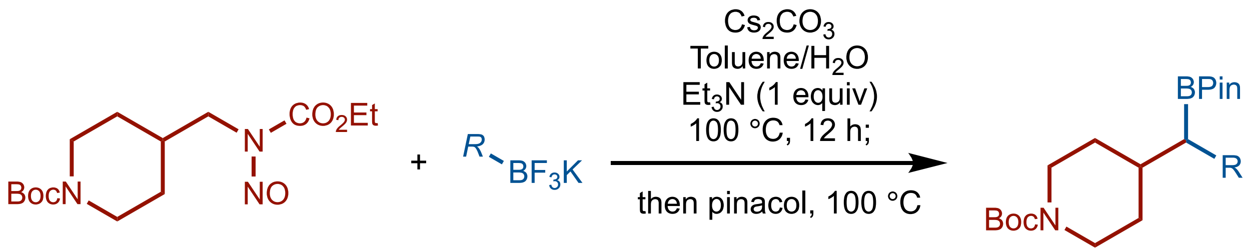


An oven dried reaction tube equipped with a stir bar was charged with nitrosocarbamate (1 Eq.), cesium carbonate (3 Eq.), and boronic acid (3 Eq.). The tube was closed with a septum and evacuated/backfilled with nitrogen. Next 2 drops of $Et_3N$, and 2 drops of water were added to the reaction mixture followed by toluene (0.1 M). The septum was replaced with a screw cap and placed in a metal block heated to 100 °C. The reaction mixture was stirred at that temperature for 16 hrs. After that, the crude reaction was cooled to room temperature, and pinacol (5 Eq.) was added followed by placed at 100 °C for 1 hr. Next, the reaction was cooled to room temperature, diluted with ethyl acetate, and passed through a pad of celite. The filtrate was evaporated to dryness and purified via flash column chromatography.

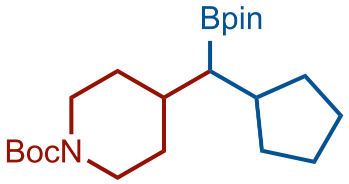


**10**

**tert-butyl 4-(cyclopentyl(4,4,5,5-tetramethyl-1,3,2-dioxaborolan-2-yl)methyl)piperidine-1-carboxylate**: This compound was synthesized according to general procedure **4** using tert-butyl 4-(((ethoxycarbonyl)(nitroso)amino)methyl)piperidine-1-carboxylate (63.1 mg, 1 Eq., 0.200

mmol) and potassium cyclopentyltrifluoroborate (106 mg, 3 Eq., 600 µmol), (prepared from treatment Cyclopentylboronic acid and 5 Eq. of Potassium bifluoride in methanol ) to afford **10** as a white solid (61 mg, 0.16 mmol, 78 %). **R$_f$**: 0.6 In 20% EA/Hex. Spectral data matched with the literature report.[6]

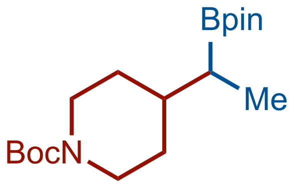


**8**

**tert-butyl 4-(1-(4,4,5,5-tetramethyl-1,3,2-dioxaborolan-2-yl)ethyl)piperidine-1-carboxylate:** This compound was synthesized according to general procedure **4** using tert-butyl 4-(((ethoxycarbonyl)(nitroso)amino)methyl)piperidine-1-carboxylate (63.1 mg, 1 Eq., 0.200 mmol) and potassium trifluoro(methyl)borate (73.2 mg, 3 Eq., 600 µmol) to afford **8** as a white solid (51 mg, 0.15 mmol, 75 %). **R$_f$**: 0.5 in 15% EA/Hex. Spectral data matched with the literature report.[6]

## VIII. General procedure 5: Cross coupling with aryl boronic acid-mitigation of protodeborylation.

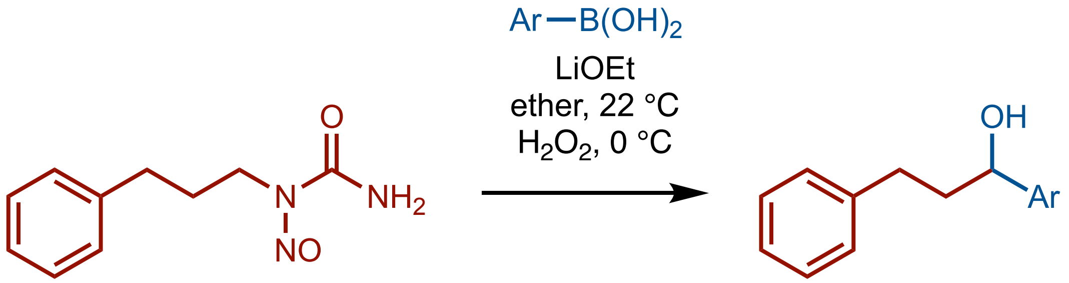


A reaction tube equipped with a stir bar was charged with nitrosourea (1 Eq.), LiOEt (6 Eq.), and aryl boronic acid (3 Eq.). The tube was closed with a septum and evacuated/backfilled with nitrogen. Next diethyl ether (0.1 M) was added to the tube and

the septum was replaced with a screw cap and stirred at room temperature for 48 hrs. The crude reaction was cooled to 0 °C, and $H_2O_2$ (0.5 ml) was added followed by stirring at the same temperature. After 30 mins, the reaction was warm to room temperature, and the aqueous layer was extracted with ethyl acetate (3 times). The combined organic layers were dried over $Na_2SO_4$ and filtered. The filtrate was evaporated to dryness and purified via flash column chromatography.

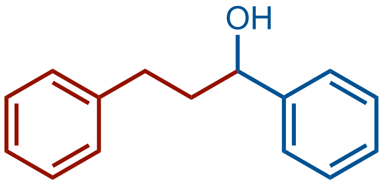

**32**

**1,3-diphenylpropan-1-ol:** This compound was synthesized according to general procedure **5** using 1-nitroso-1-(3-phenylpropyl)urea (41.4 mg, 1 Eq., 0.200 mmol) and phenylboronic acid (146 mg, 6.00 Eq., 1.20 mmol) to afford **32** as an oil (26 mg, 0.12 mmol, 61 %) **$R_f$**: 0.5 in 20% EA/Hex. Spectral data matched with the literature report.[16]

**$^1$H NMR (500 MHz, $CDCl_3$)** δ 7.38 (d, *J* = 4.3 Hz, 4H), 7.35 – 7.26 (m, 3H), 7.22 (dd, *J* = 7.8, 2.0 Hz, 3H), 4.72 (ddd, *J* = 8.5, 5.3, 3.5 Hz, 1H), 2.23 – 2.12 (m, 1H), 2.12 – 2.01 (m, 1H), 1.84 (d, *J* = 3.5 Hz, 1H).

**$^{13}$C NMR (126 MHz, $CDCl_3$)** δ 144.7, 141.9, 128.7, 128.6, 128.6, 127.8, 126.1, 126.0, 74.1, 40.6, 32.2.

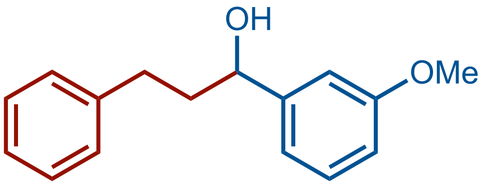

**33**

**1-(3-methoxyphenyl)-3-phenylpropan-1-ol:** This compound was synthesized according to general procedure **5** using 1-nitroso-1-(3-phenylpropyl)urea (41.4 mg, 1 Eq., 0.200 mmol) and (3-methoxyphenyl)boronic acid (182 mg, 6 Eq., 1.20 mmol) to afford **33** as an oil (31 mg, 0.13 mmol, 64 %) **$R_f$**: 0.6 in 20% EA/Hex. Spectral data matched with the literature report.[17]

**$^1$H NMR (600 MHz, $CDCl_3$)** δ 7.27 – 7.22 (m, 3H), 7.19 – 7.15 (m, 3H), 6.94 – 6.88 (m, 2H), 6.81 – 6.78 (m, 1H), 4.64 (dd, *J* = 7.9, 5.3 Hz, 1H), 3.79 (s, 3H), 2.73 (ddt, *J* = 13.5, 9.4, 4.7 Hz, 1H), 2.65 (ddd, *J* = 14.0, 9.5, 6.4 Hz, 1H), 2.10 (dddd, *J* = 13.7, 9.6, 7.9, 5.7 Hz, 1H), 2.01 (dddd, *J* = 13.8, 9.8, 6.5, 5.3 Hz, 1H).

**$^{13}$C NMR (126 MHz, $CDCl_3$)** δ 160.0, 146.5, 141.9, 129.7, 128.6, 128.5, 126.0, 118.4, 113.2, 111.6, 74.0, 55.4, 40.6, 32.2.

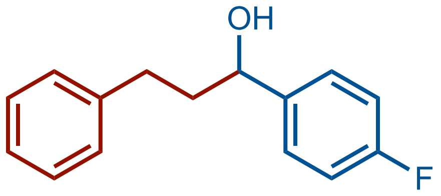


**34**

**1-(4-fluorophenyl)-3-phenylpropan-1-ol:** This compound was synthesized according to general procedure **5** using 1-nitroso-1-(3-phenylpropyl)urea (41.4 mg, 1 Eq, 0.200 mmol) and (4-fluorophenyl)boronic acid (168 mg, 6 Eq, 1.20 mmol) to afford **34** (23 mg, 0.10 mmol, 50 %) as an oil. **$R_f$**: 0.6 in 20% EA/Hex. Spectral data matched with the literature report.[18]

**$^1$H NMR (600 MHz, $CDCl_3$)** δ 7.32 (dd, *J* = 8.4, 5.5 Hz, 2H), 7.28 (t, *J* = 7.6 Hz, 2H), 7.19 (t, *J* = 6.9 Hz, 3H), 7.04 (t, *J* = 8.7 Hz, 2H), 4.68 (ddd, *J* = 8.4, 5.0, 3.3 Hz, 1H), 2.74 (ddd,

*J* = 15.1, 9.7, 5.8 Hz, 1H), 2.66 (ddd, *J* = 14.0, 9.5, 6.5 Hz, 1H), 2.16 – 2.07 (m, 1H), 2.07 – 1.97 (m, 1H), 1.82 (d, *J* = 3.4 Hz, 1H).

**$^{13}$C NMR (126 MHz, CDCl$_3$)** δ 163.34 (d, *J* = 246 Hz), 141.71, 140.45 (d, *J* = 3 Hz), 128.59 (d, *J* = 4 Hz), 127.74 (d, *J* = 9 Hz), 126.09, 115.56, 115.39, 73.37, 40.72, 32.15.

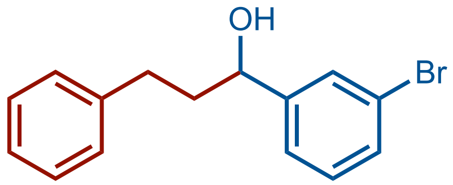


**35**

**1-(3-bromophenyl)-3-phenylpropan-1-ol:** This compound was synthesized according to general procedure **5** using 1-nitroso-1-(3-phenylpropyl)urea (41.4 mg, 1 Eq, 0.200 mmol) and (3-bromophenyl)boronic acid (241 mg, 6 Eq, 1.20 mmol) to afford **35** (12 mg, 41 µmol, 21 %) as an oil.

**$R_f$**: 0.5 in 20% EA/Hex. Spectral data matched with the literature report.[19]

**$^{1}$H NMR (500 MHz, CDCl$_3$)** δ 7.44 (d, *J* = 2.0 Hz, 1H), 7.41 – 7.27 (m, 1H), 7.20 (dd, *J* = 15.3, 7.9 Hz, 3H), 7.17 – 7.09 (m, 4H), 4.58 (dt, *J* = 8.3, 4.1 Hz, 1H), 2.74 – 2.55 (m, 2H), 2.01 (ddd, *J* = 14.3, 9.9, 5.7 Hz, 1H), 1.98 – 1.89 (m, 1H), 1.80 (d, *J* = 3.5 Hz, 1H).

**$^{13}$C NMR (126 MHz, CDCl$_3$)** δ 147.10, 141.57, 130.80, 130.24, 129.18, 128.61, 128.57, 126.14, 124.65, 122.80, 73.30, 40.65, 32.06.

*Substrate limitation*

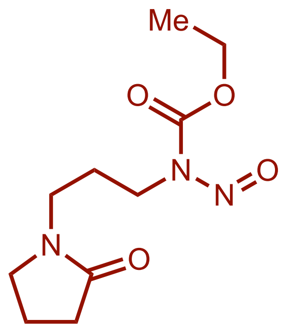


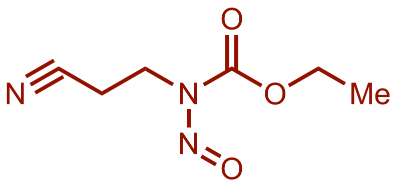


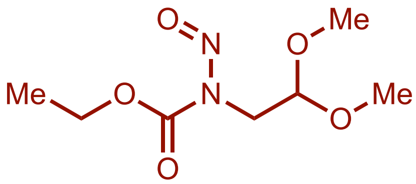


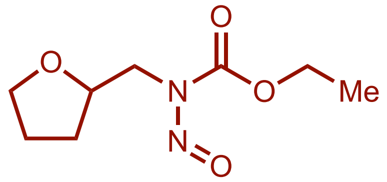


## IX. General procedure 6: Lysin side chain modification.

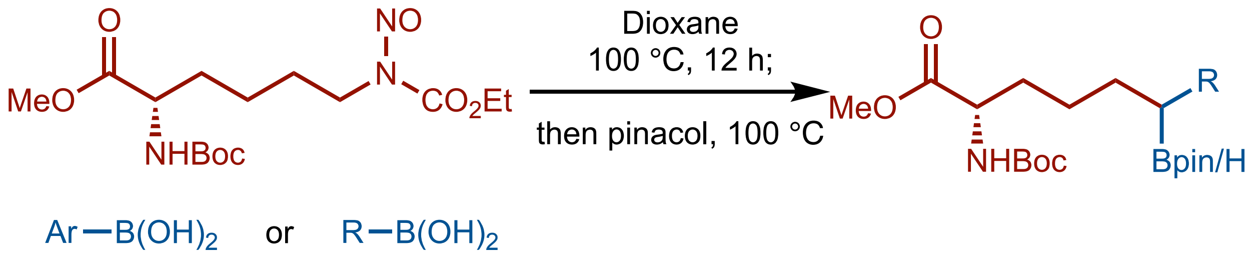


A reaction tube equipped with a stir bar was charged with nitrosocarbamate (1 Eq.) and aryl/alkyl boronic acid (3 Eq.). The tube was closed with a septum and evacuated/backfilled with nitrogen. Next dioxane (0.1 M) was added to the tube and the septum was replaced with a screw cap and placed in a metal block heated to 100 °C. The reaction mixture was stirred at that temperature for 16 hrs. Next, the crude reaction was cooled to room temperature, and pinacol (5 Eq.) was added followed by placed in 100 °C for 1 hr. After that, the reaction was cooled to room temperature, diluted with ethyl acetate,

and passed through a pad of celite. The filtrate was evaporated to dryness and purified via flash column chromatography.

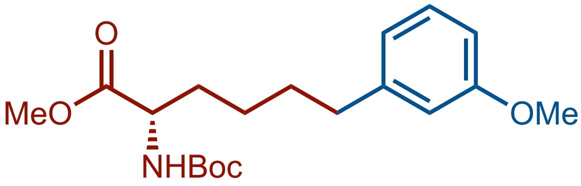


**38**

**methyl (S)-2-((tert-butoxycarbonyl)amino)-6-(3-methoxyphenyl)hexanoate:** This compound was synthesized according to general procedure **6** using methyl N2-(tert-butoxycarbonyl)-N6-(ethoxycarbonyl)-N6-nitroso-L-lysinate (35.00 mg, 1 Eq, 96.85 μmol) and (3-methoxyphenyl)boronic acid (46 mg, 3 Eq., 300.0 μmol) to afford **38** as an oil methyl (S)-2-((tert-butoxycarbonyl)amino)-6-(3-methoxyphenyl)hexanoate (18 mg, 51 μmol, 53 %) $\mathbf{R_f}$: 0.6 in 20% EA/Hex. Spectral data matched with the literature report.[20]

**NMR (500 MHz, $CDCl_3$)** δ 7.18 (t, $J$ = 7.8 Hz, 1H), 6.78 – 6.68 (m, 3H), 4.98 (d, $J$ = 8.5 Hz, 1H), 4.30 (q, $J$ = 7.3 Hz, 1H), 3.79 (s, 3H), 3.72 (s, 3H), 2.58 (t, $J$ = 7.7 Hz, 2H), 1.81 (td, $J$ = 11.4, 6.2 Hz, 1H), 1.64 (dt, $J$ = 15.0, 7.5 Hz, 3H), 1.44 (s, 9H), 1.41 – 1.30 (m, 2H).

**$^{13}$C NMR (126 MHz, $CDCl_3$)** δ 173.6, 159.8, 155.5, 144.0, 129.4, 121.0, 114.3, 111.2, 80.0, 55.26, 53.5, 52.4, 35.8, 32.8, 31.0, 28.5, 25.0.

**HPLC:** >99:1 er. The enantiopurity was determined by HPLC analysis using Lux Cellulose-2 column, 1:99 Hexane: isopropanol, 254 nm. t = 29.332 min. HPLC chromatogram is attached below.

(The material was also prepared using 3 eq. $Cs_2CO_3$, which afforded **38** with 45% ee. A racemic trace is also provided in the HPLC section.)

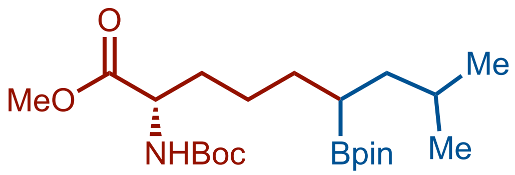


**39**

**methyl (2S)-2-((tert-butoxycarbonyl)amino)-8-methyl-6-(4,4,5,5-tetramethyl-1,3,2-dioxaborolan-2-yl)nonanoate**: This compound was synthesized according to general procedure **6** using methyl methyl N2-(tert-butoxycarbonyl)-N6-(ethoxycarbonyl)-N6-nitroso-L-lysinate (36.14 mg, 1 Eq, 0.1000 mmol) and isobutylboronic acid (30.58 mg, 3 Eq, 300.0 μmol) to afford **39** as an oil (33 mg, 77 μmol, 77 %).

**$R_f$**: 0.6 in 20% EA/Hex

**$^1$H NMR (500 MHz, $CDCl_3$)** δ 5.03 – 4.92 (m, 1H), 4.32 – 4.21 (m, 1H), 3.71 (d, *J* = 2.0 Hz, 3H), 1.76 (q, *J* = 9.2 Hz, 1H), 1.61 (d, *J* = 16.5 Hz, 1H), 1.59 – 1.49 (m, 1H), 1.43 (s, 10H), 1.39 – 1.27 (m, 5H), 1.22 (s, 12H), 1.13 (dddd, *J* = 13.5, 8.3, 6.5, 2.0 Hz, 1H), 1.07 – 0.98 (m, 1H), 0.85 (dd, *J* = 6.6, 2.2 Hz, 6H).

**$^{13}$C NMR (126 MHz, $CDCl_3$)** δ 173.7, 155.5, 83.0, 79.9, 53.7 (d), 52.3 (d), 40.8 (d), 33.0 (d), 31.3 (d), 28.5, 27.4, 25.1, 25.0 (d), 23.2(d), 22.7.

**IR (neat):** 3359 (br), 2977 (m), 1745 (m), 1716 (s), 1460 (w), 1389 (m), 1163 (s).

**HRMS (ESI):** Calculated for $C_{22}H_{42}O_6NBNa$ $[M+Na]^+$ 450.2997; found 450.3004.

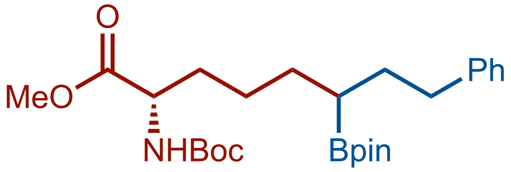


**40**

**Methyl (2S)-2-((tert-butoxycarbonyl)amino)-8-phenyl-6-(4,4,5,5-tetramethyl-1,3,2-dioxaborolan-2-yl)octanoate:** This compound was synthesized according to general

procedure **F** using methyl N2-(tert-butoxycarbonyl)-N6-(ethoxycarbonyl)-N6-nitroso-D-lysinate (36.14 mg, 1 Eq., 0.1000 mmol) and phenethylboronic acid (89.99 mg, 6 Eq., 600.0 μmol) to afford **40** as an oil (26 mg, 55 μmol, 55 %) **$R_f$**: 0.6 in 30% EA/Hex.

**$^1$H NMR (600 MHz, $CDCl_3$)** δ 7.28 – 7.25 (m, 2H), 7.17 (d, *J* = 7.6 Hz, 4H), 4.98 (d, *J* = 8.4 Hz, 1H), 4.32 – 4.23 (m, 1H), 3.72 (d, *J* = 2.1 Hz, 3H), 2.65 – 2.53 (m, 2H), 1.74 (dtt, *J* = 14.1, 9.5, 4.8 Hz, 2H), 1.66 – 1.59 (m, 2H), 1.49 (d, *J* = 6.9 Hz, 1H), 1.44 (s, 9H), 1.41 – 1.29 (m, 3H), 1.26 (s, 12H), 1.03 (ddd, *J* = 9.1, 6.2, 2.7 Hz, 1H).

**$^{13}$C NMR (126 MHz, $CDCl_3$)** δ 173.6, 155.5, 143.1, 128.5, 128.4, 125.7, 83.2, 79.9, 53.6, 52.3, 35.7, 33.5, 33.0, 30.9, 28.5, 25.0, 24.9. (Signal of carbon directly bonded to boron was not detected because of quadrupolar relaxation)

**IR (neat):** 3384 (br), 2978 (w), 2931 (w), 1698 (s), 1497 (m), 1454 (m), 1367 (m), 1161 (s) $cm^{-1}$.

**HRMS (ESI):** Calculated for $C_{26}H_{42}O_6NBNa$ $[M+Na]^+$ 498.2997; found 498.3005.

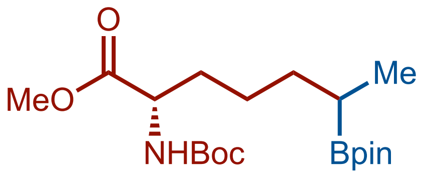


**41**

**methyl (2S)-2-((tert-butoxycarbonyl)amino)-6-(4,4,5,5-tetramethyl-1,3,2-dioxaborolan-2-yl)heptanoate:** This compound was synthesized according to general procedure **6** using methyl methyl N2-(tert-butoxycarbonyl)-N6-(ethoxycarbonyl)-N6-nitroso-L-lysinate (36.14 mg, 1 Eq, 0.1000 mmol) and methyl boronic acid (17.96 mg, 3 Eq, 0.3 mmol) to afford **41** as an oil (18 mg, 47 μmol, 47 %)

**$R_f$**: 0.6 in 30% EA/Hex

**$^{1}$H NMR (500 MHz, CDCl$_3$)** δ 4.98 (d, *J* = 8.4 Hz, 1H), 4.27 (s, 1H), 3.72 (d, *J* = 1.0 Hz, 3H), 1.77 (q, *J* = 7.5 Hz, 1H), 1.61 (q, *J* = 7.9 Hz, 2H), 1.43 (s, 9H), 1.39 – 1.25 (m, 4H), 1.23 (s, 12H), 1.04 – 0.91 (m, 4H).

**$^{13}$C NMR (126 MHz, CDCl$_3$)** δ 173.7, 155.5, 83.0, 79.9, 53.7 (d), 52.3, 32.9 (d), 28.5, 24.9 (d), 24.9 (d), 24.7 (d), 15.6 (d). (Signal of carbon directly bonded to boron was not detected because of quadrupolar relaxation)

**IR (neat):** 3363 (br), 2978 (m), 1745 (m), 1715 (s), 1501 (w), 1366 (m), 1163 (s).

**HRMS (ESI):** Calculated for $C_{19}H_{36}O_6NBNa$ $[M+Na]^+$ 408.2528; found 408.2535.

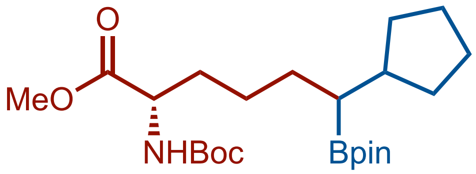


**42**

**Methyl (2S)-2-((tert-butoxycarbonyl)amino)-6-cyclopentyl-6-(4,4,5,5-tetramethyl-1,3,2-dioxaborolan-2-yl)hexanoate:** This compound was synthesized according to general procedure **F** using methyl methyl N2-(tert-butoxycarbonyl)-N6-(ethoxycarbonyl)-N6-nitroso-D-lysinate (36.14 mg, 1 Eq., 0.1000 mmol) and cyclopentylboronic acid (68.37 mg, 6 Eq., 0.6 mmol) to afford **42** as an oil (31 mg, 71 µmol, 71 %) **R$_f$**: 0.6 in 30% EA/Hex.

**$^{1}$H NMR (500 MHz, CDCl$_3$)** δ 4.96 (t, *J* = 6.5 Hz, 1H), 4.25 (dd, *J* = 12.5, 6.3 Hz, 1H), 3.70 (d, *J* = 2.5 Hz, 3H), 1.86 – 1.65 (m, 5H), 1.63 – 1.52 (m, 3H), 1.42 (s, 14H), 1.23 (s, 12H), 1.15 – 1.00 (m, 2H), 0.84 (q, *J* = 8.6 Hz, 1H).

**$^{13}$C NMR (126 MHz, CDCl$_3$)** δ 173.7, 155.5, 83.0, 79.8, 53.7, 52.3, 42.0, 33.0, 32.7, 32.2, 30.5, 28.5, 25.4, 25.2, 25.0, 24.9. (Signal of carbon directly bonded to boron was not detected because of quadrupolar relaxation)

**IR (neat):** 3363 (br), 2948 (w), 2865 (w), 1745 (s), 1716 (s), 1501 (m), 1367 (m), 1163 (s) $cm^{-1}$.

**HRMS (ESI):** Calculated for $C_{23} H_{42} O_6$ N B Na $[M+Na]^+$ 462.2997; found 462.3006.

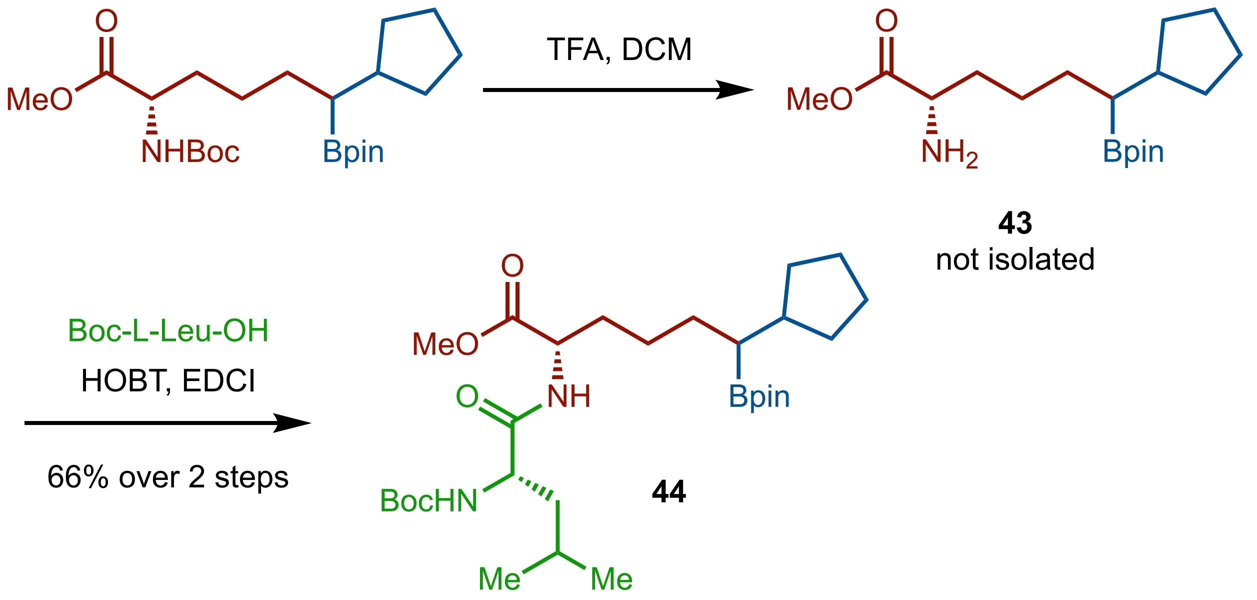


**methyl (2*S*)-2-((*S*)-2-((*tert*-butoxycarbonyl)amino)-4-methylpentanamido)-6-cyclopentyl-6-(4,4,5,5-tetramethyl-1,3,2-dioxaborolan-2-yl hexanoate):** To a solution of BOC-L-Leucine-OH (15.0 mg, 1.2 Eq, 60.0 µmol), 1-Hydroxybenzotriazole - hydrate (14.4 mg, 9.98 µL, 80% Wt, 1.5 Eq, 75.0 µmol) and 1-(3-Dimethylaminopropyl)-3-ethylcarbodiimideHydrochloride(EDCI) (14.4 mg, 1.5 Eq, 75.0 µmol) in 0.1 M DMF, 0.5 ml was added diisopropylethylamine (19.4 mg, 25.8 µL, 3 Eq, 150 µmol) dropwise and stirred at 0 °C for 30 mins. To this was added methyl (2S)-2-amino-6-cyclopentyl-6-(4,4,5,5-tetramethyl-1,3,2-dioxaborolan-2-yl)hexanoate (17.0 mg, 1 Eq, 0.0500 mmol) dissolved in 0.5 ml (0.1 M) DMF and the reaction mixture was stirred overnight. The next day, the reaction mixture was diluted with water, extracted with ethyl acetate (2 * 3 ml), dried over $Na_2SO_4$ and evaporated under reduced pressure. The crude mixture was

purified under purified via flash column chromatography to afford **44** as a colourless oil (18.3 mg, 33.1 μmol, 66%). **$R_f$**: 0.5 in 50% EA/Hex.

**$^1$H NMR (500 MHz, $CDCl_3$)** δ 6.47 (dd, *J* = 13.2, 7.9 Hz, 1H), 4.87 (s, 1H), 4.53 (dtd, *J* = 13.0, 7.3, 5.6 Hz, 1H), 4.10 (d, *J* = 7.4 Hz, 1H), 3.71 (s, 3H), 1.87 – 1.74 (m, 3H), 1.74 – 1.61 (m, 5H), 1.61 – 1.52 (m, 2H), 1.44 (s, 13H), 1.41 – 1.35 (m, 2H), 1.23 (s, 13H), 1.08 (dtd, *J* = 16.1, 7.9, 4.4 Hz, 2H), 0.93 (dd, *J* = 6.3, 3.4 Hz, H), 0.83 (qd, *J* = 7.0, 2.7 Hz, 1H).

**$^{13}$C NMR (126 MHz, $CDCl_3$)** δ 172.9, 172.3, 155.7, 83.0, 80.2, 53.2, 52.4, 52.3, 42.0, 41.5, 32.9, 32.7, 32.2, 30.6, 28.4, 25.4, 25.2, 25.0, 24.9, 23.1, 22.2. (Signal of carbon directly bonded to boron was not detected because of quadrupolar relaxation)

**IR (neat):** 3301 (br), 2951 (w), 2857 (w), 1746 (s), 1657 (m), 1523 (m), 1367 (m), 1164 (s) $cm^{-1}$.

**HRMS (ESI):** Calculated for $C_{29}H_{53}O_7N_2BNa$ $[M+Na]^+$ 575.3838; found 575.3844.

# X. Spectra

3

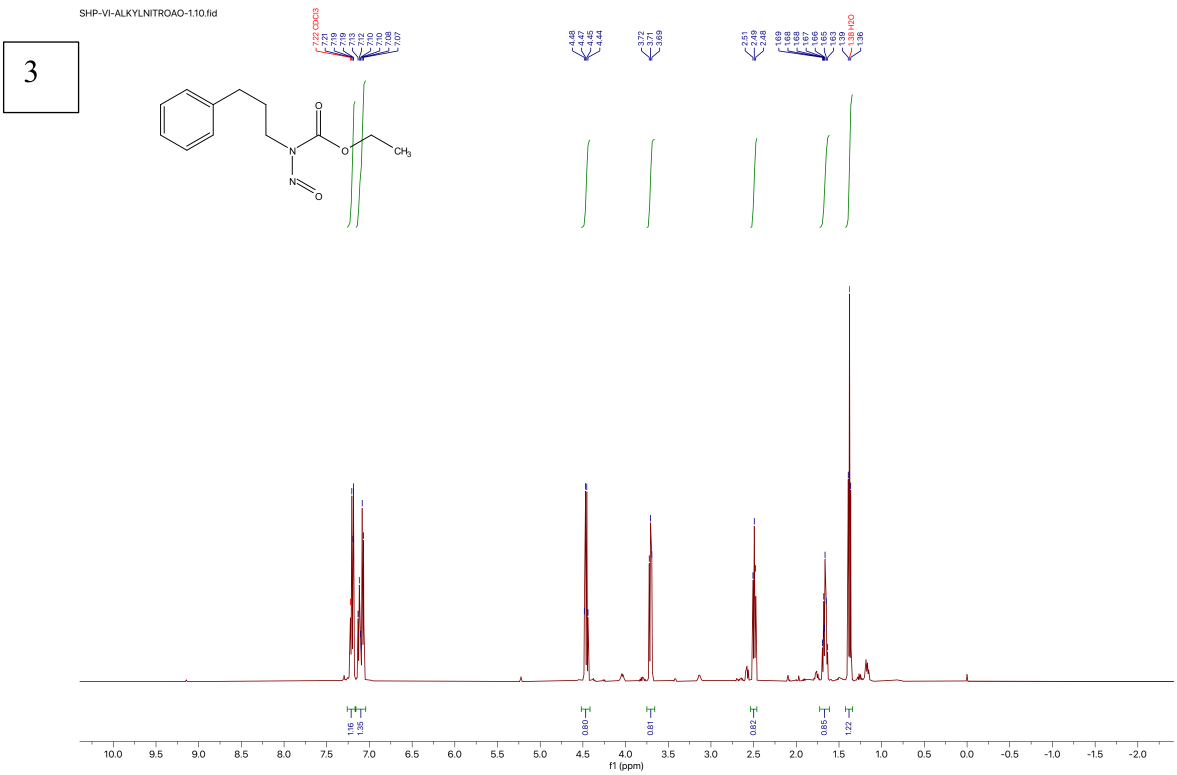

SHP-VI-ALKYLNITROAO-1.10.fid
7.22 CDCl3
7.21
7.19
7.19
7.13
7.12
7.10
7.10
7.08
7.07
4.48
4.47
4.45
4.44
3.72
3.71
3.69
2.51
2.49
2.48
1.69
1.68
1.68
1.67
1.66
1.65
1.63
1.39
1.38 H2O
1.36
CH3
O
N
N
O
1.16
1.35
0.80
0.81
0.82
0.85
1.22
f1 (ppm)


3

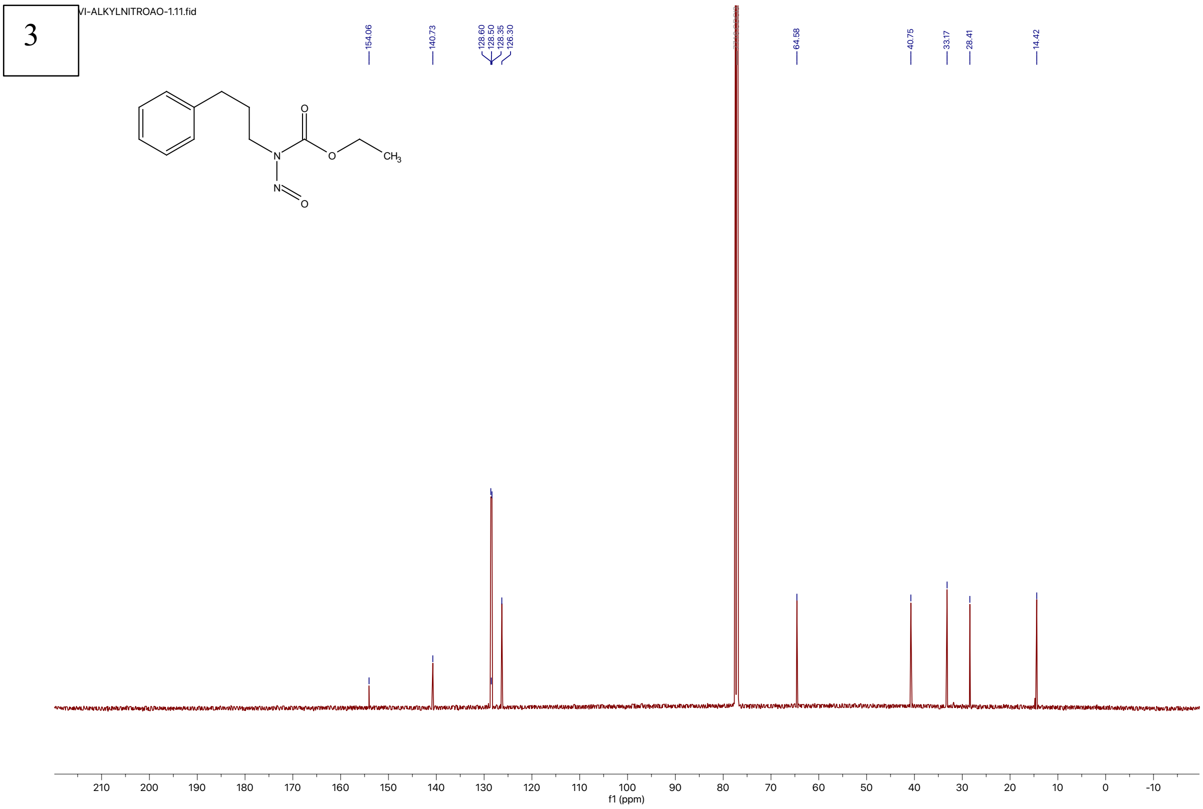

VI-ALKYLNITROAO-1.11.fid
154.06
140.73
128.60
128.50
128.35
126.30
64.58
40.75
33.17
28.41
14.42
CH3
O
N
N
O
f1 (ppm)

SI-7

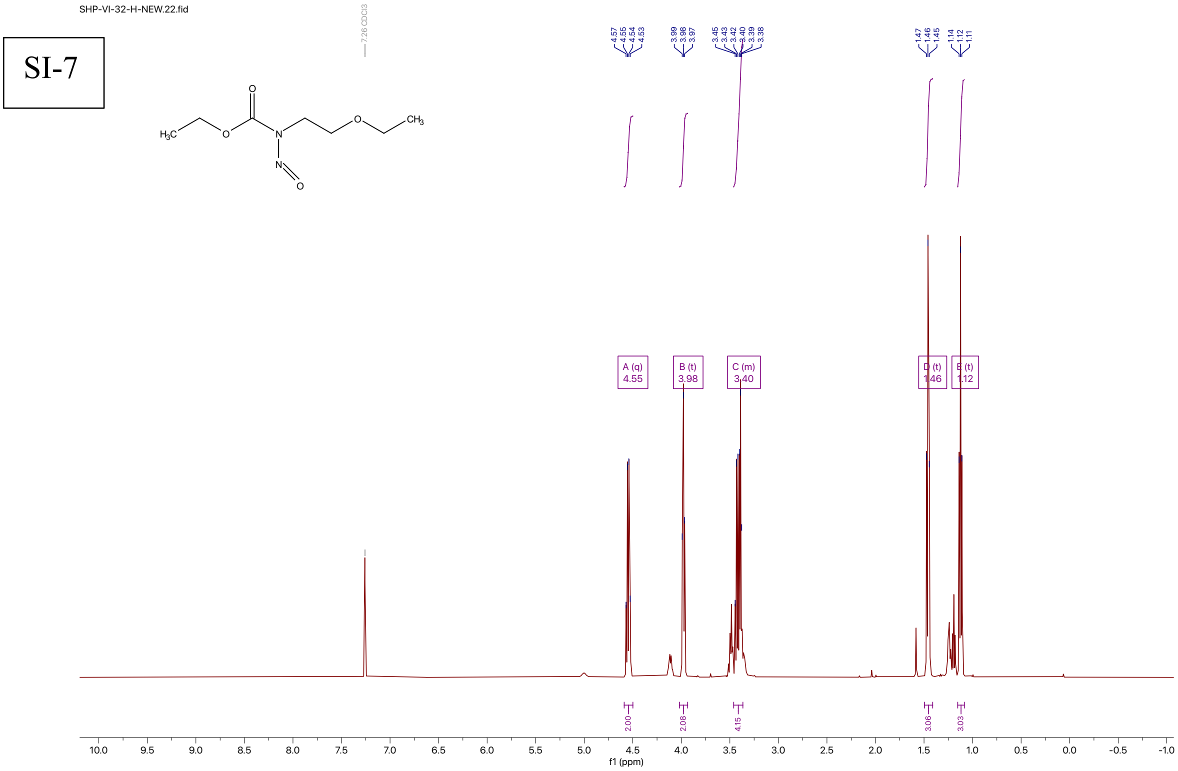
SHP-VI-32-H-NEW.22.fid
7.26 CDCl3
4.57
4.55
4.54
4.53
3.99
3.98
3.97
3.45
3.43
3.42
3.40
3.39
3.38
1.47
1.46
1.45
1.14
1.12
1.11
A (q)
4.55
B (t)
3.98
C (m)
3.40
D (t)
1.46
E (t)
1.12
2.00
2.08
4.15
3.06
3.03
f1 (ppm)

SI-7

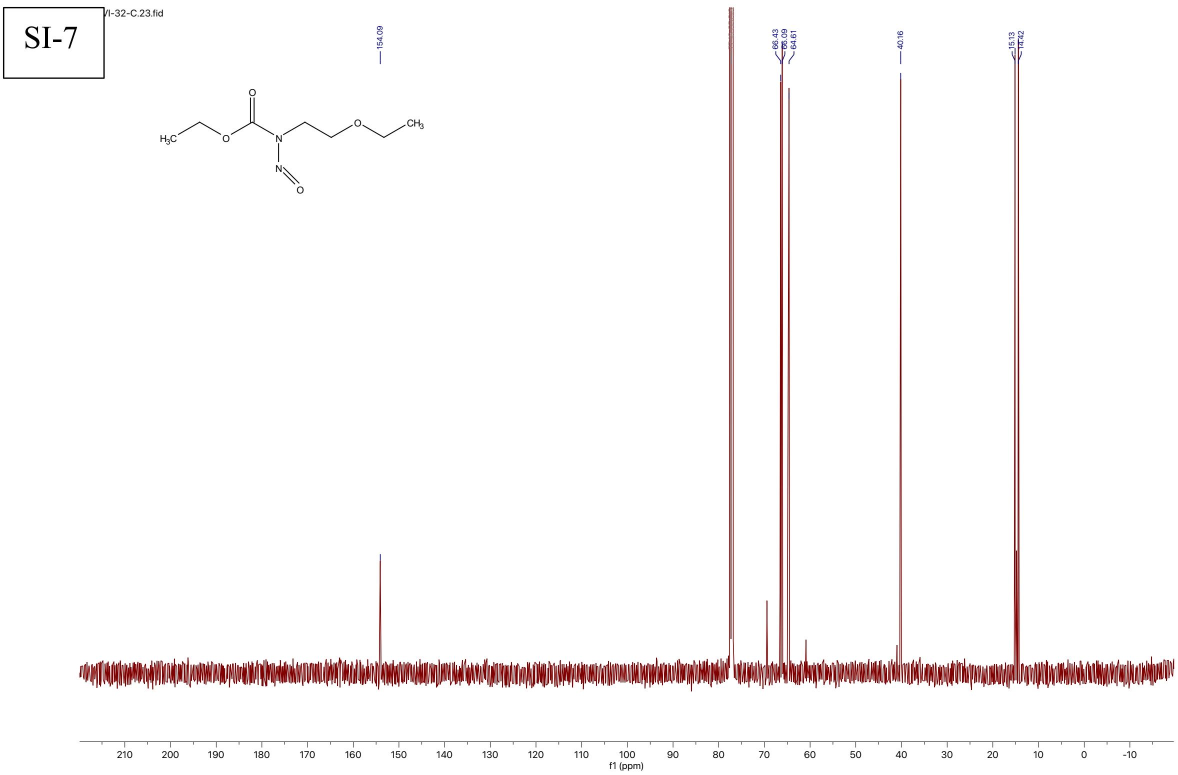
VI-32-C.23.fid
154.09
66.43
65.09
64.61
40.16
15.13
14.42
f1 (ppm)

SI-8

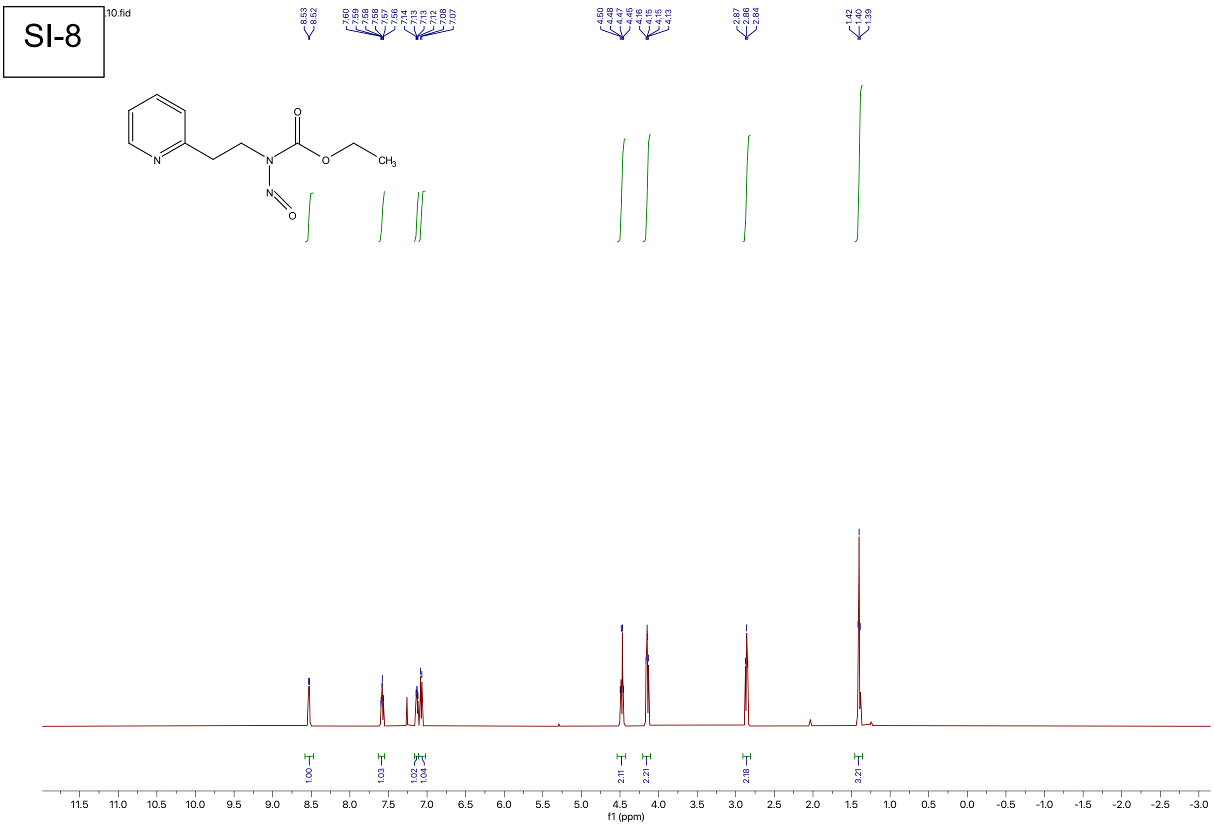

SI-8

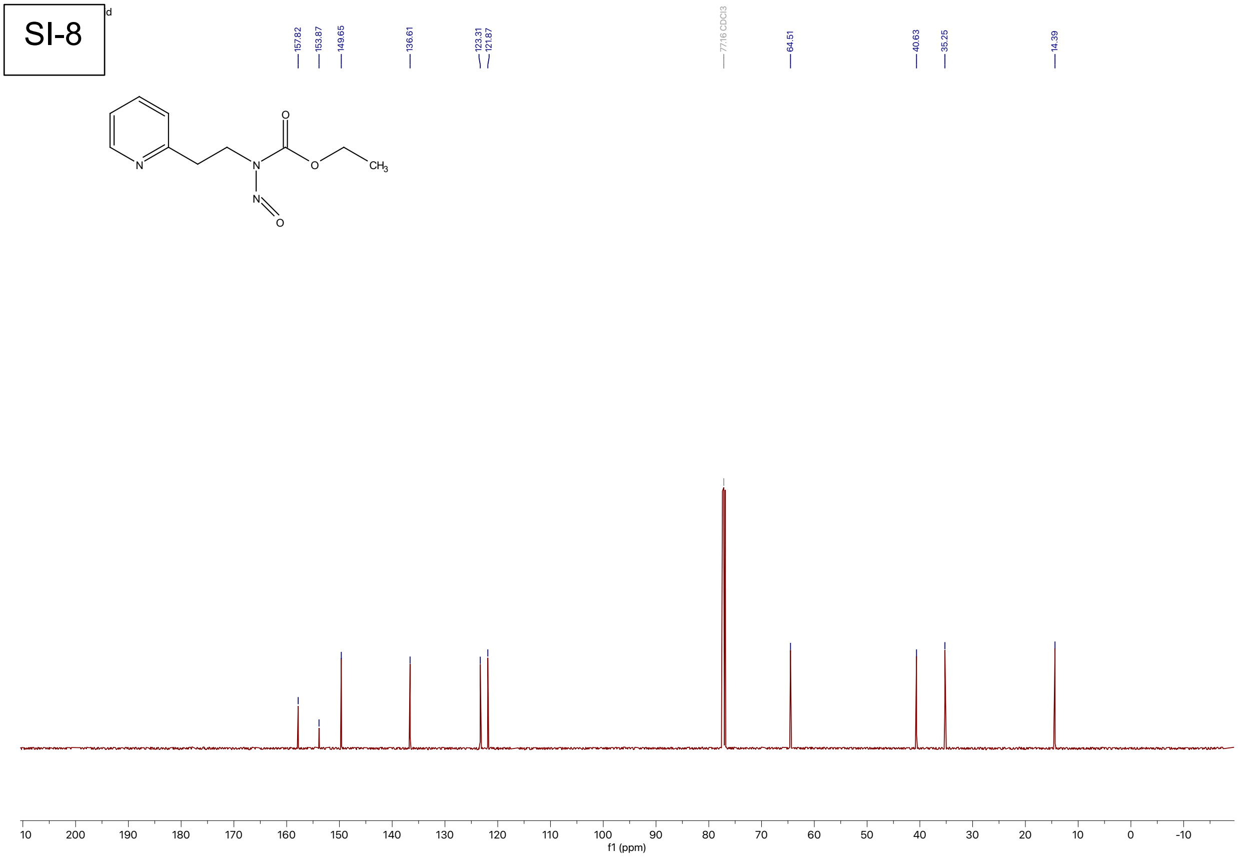

SI-9

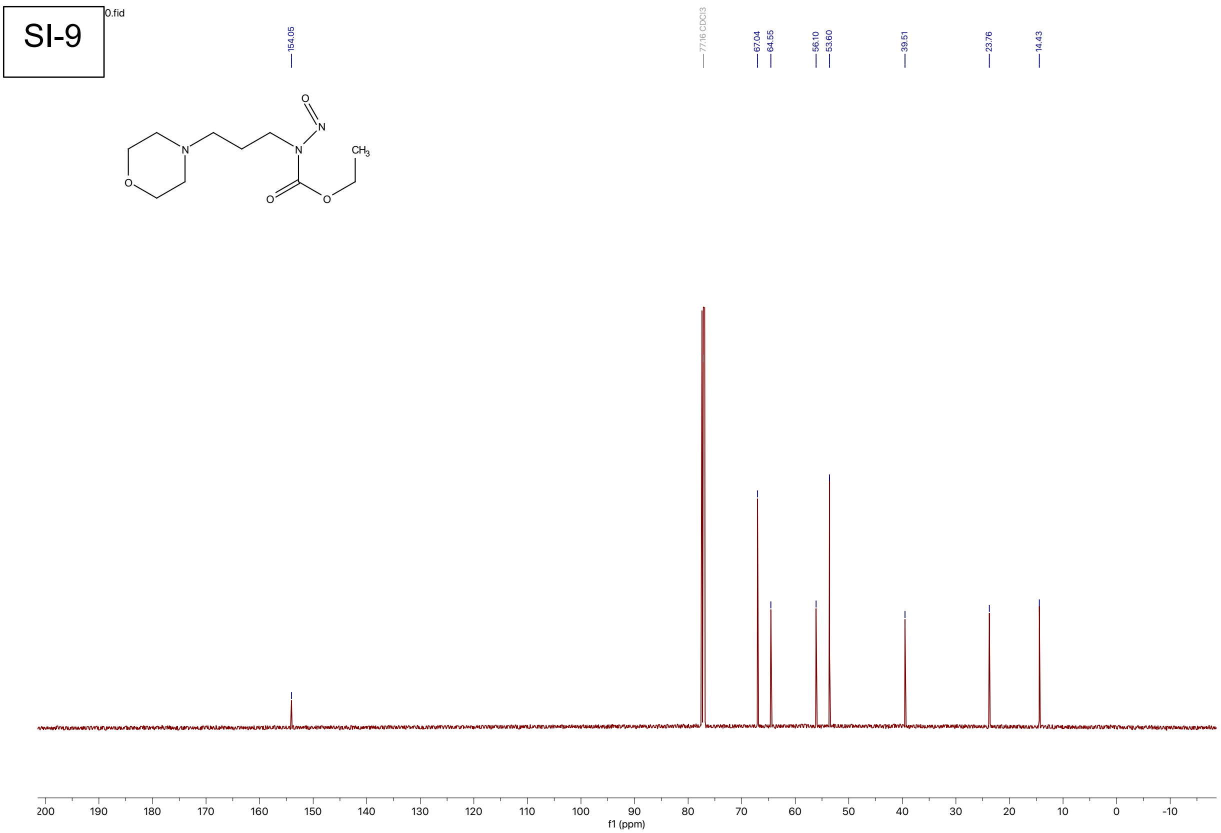

SI-9

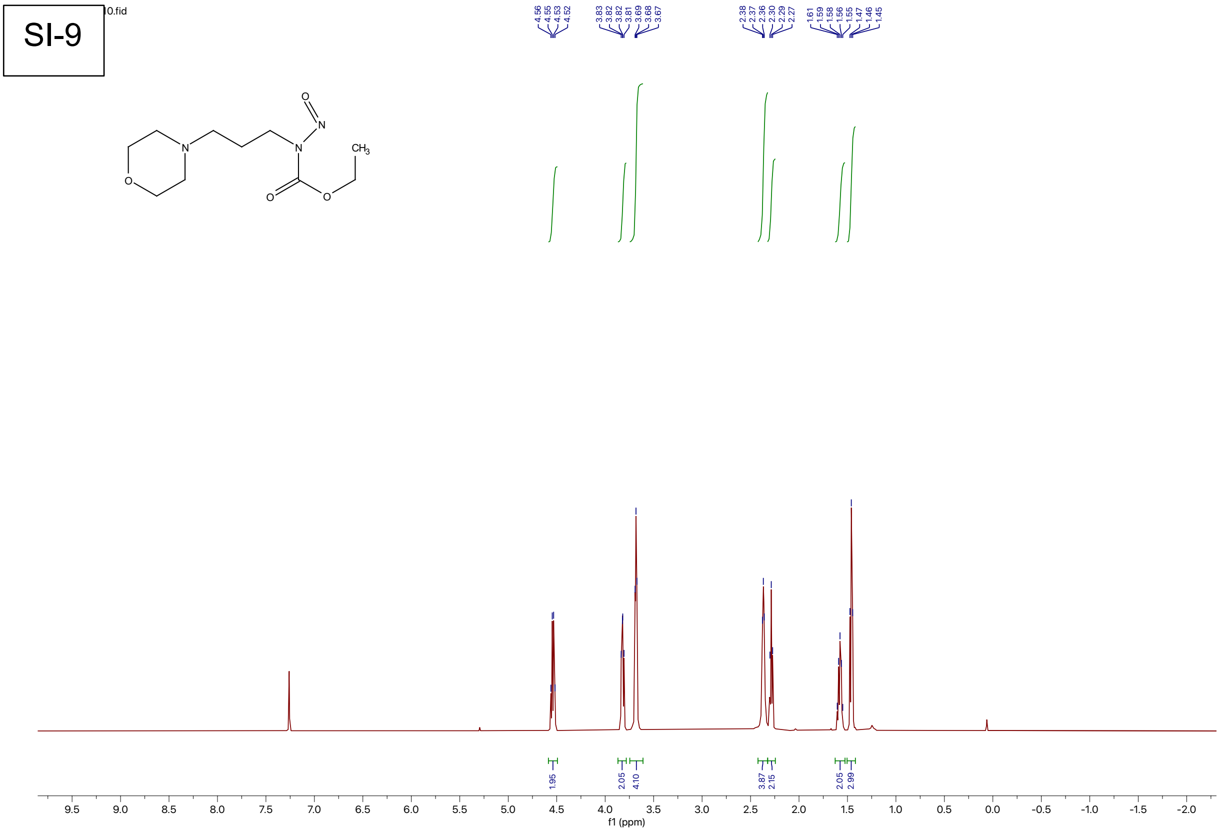

SI-10

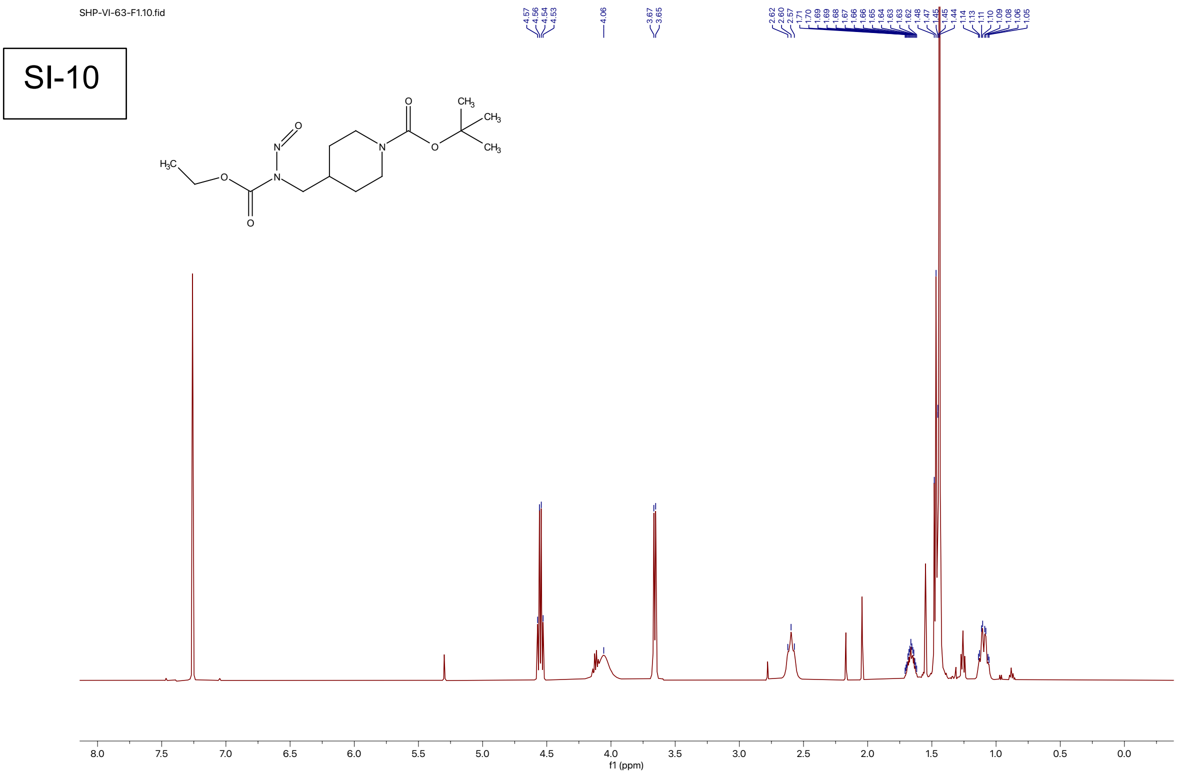
SHP-VI-63-F1.10.fid
f1 (ppm)

SI-10

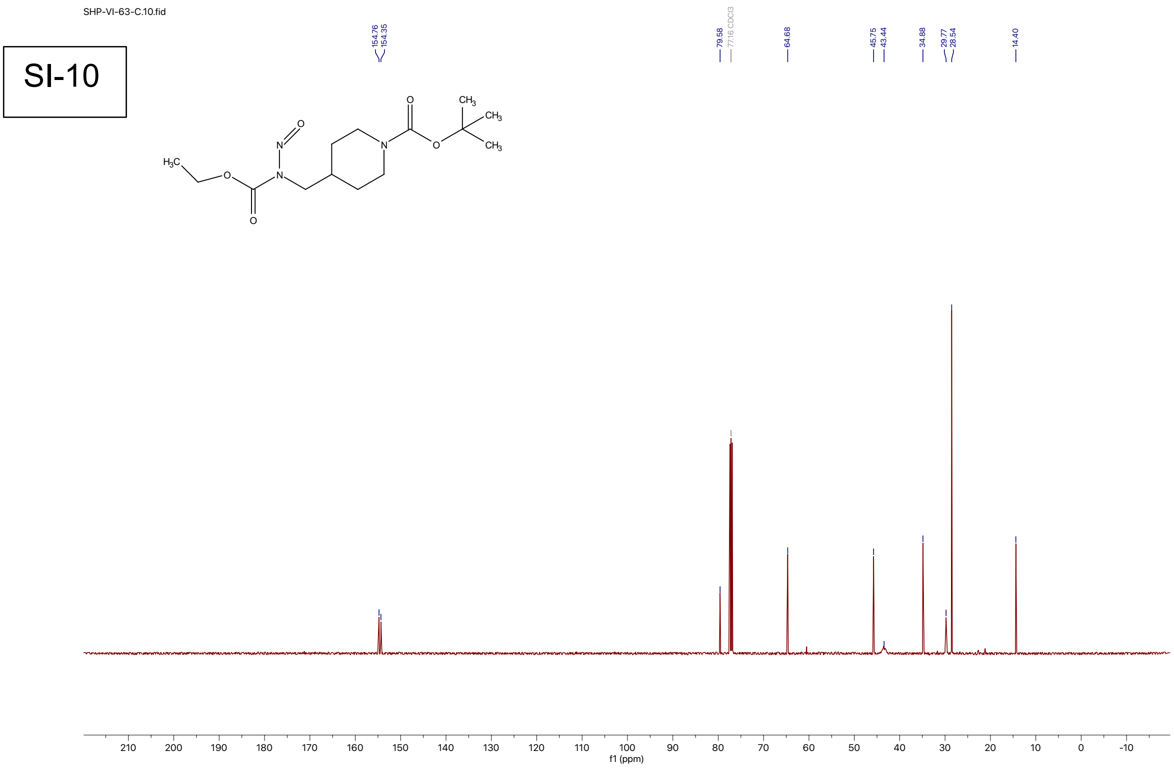
SHP-VI-63-C.10.fid
154.76
154.35
79.58
77.16 CDCl3
64.68
45.75
43.44
34.88
29.77
28.54
14.40
f1 (ppm)

SHP-VI-68-F1.10.fid

SI-11

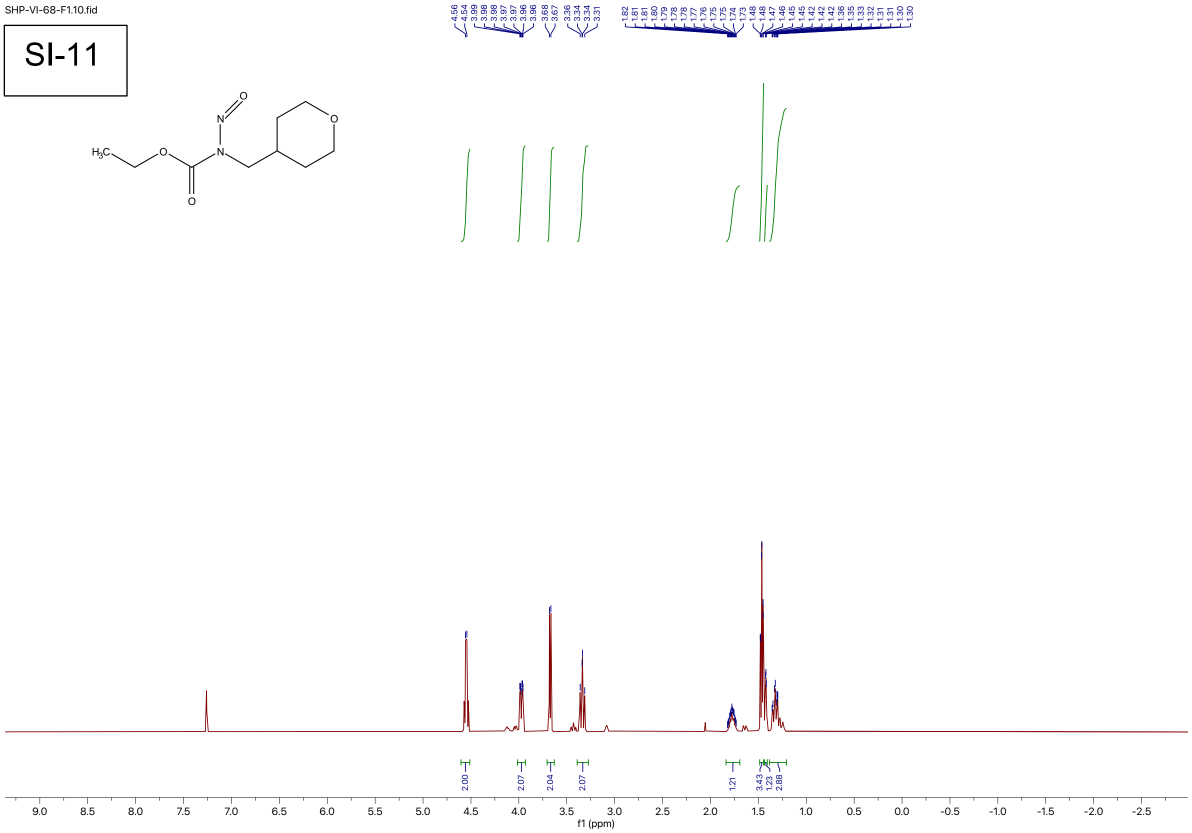


SHP-VI-68-F1-C.12.fid

SI-11

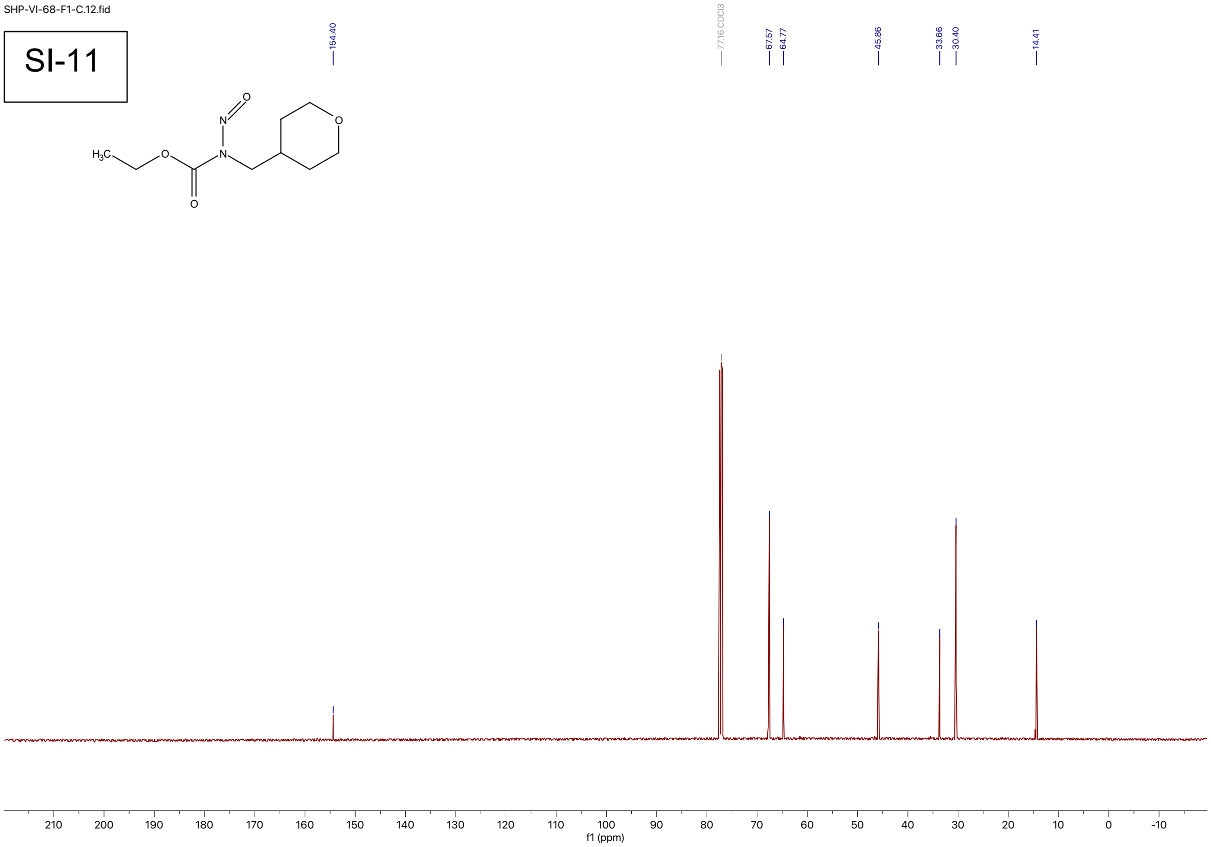

SI-12

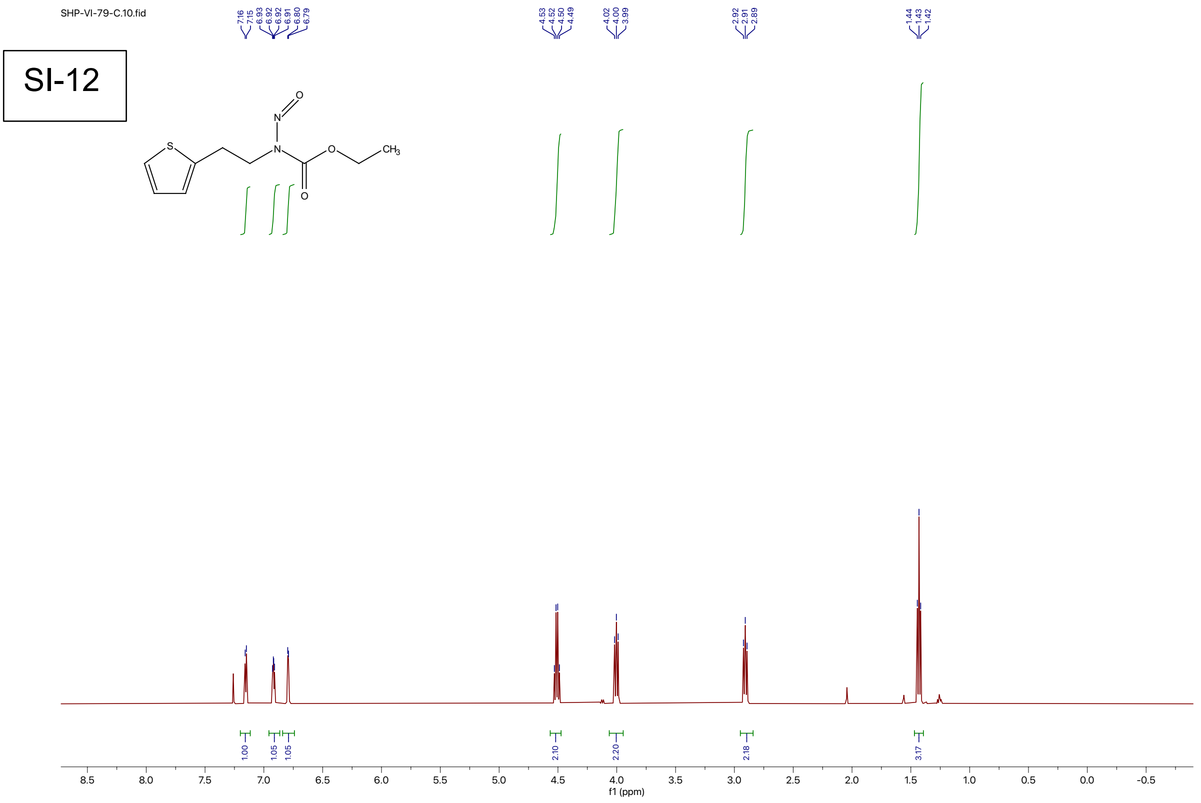
SHP-VI-79-C.10.fid
7.16
7.15
6.93
6.92
6.92
6.91
6.80
6.79
4.53
4.52
4.50
4.49
4.02
4.00
3.99
2.92
2.91
2.89
1.44
1.43
1.42
S
N
O
N
O
CH3
O
1.00
1.05
1.05
2.10
2.20
2.18
3.17
f1 (ppm)

SI-12

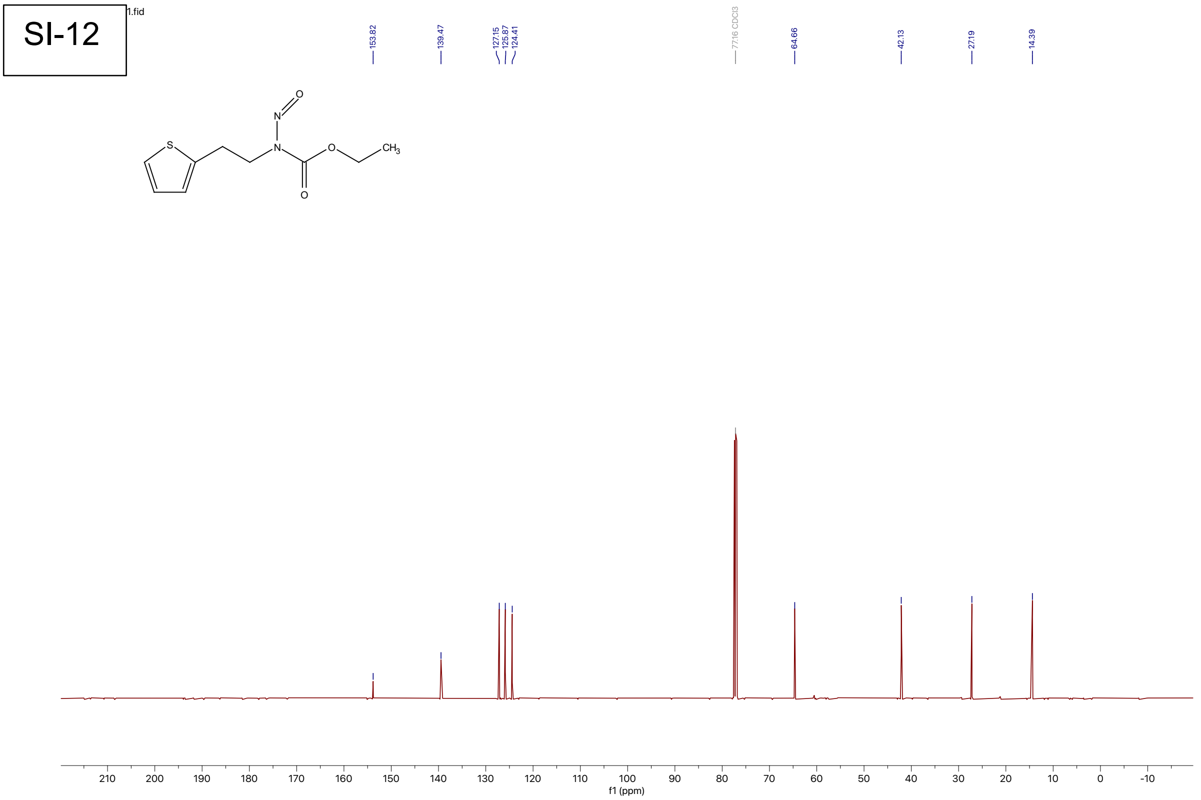
1.fid
153.82
139.47
127.15
125.87
124.41
77.16 CDCl3
64.66
42.13
27.19
14.39
S
N
O
N
O
CH3
O
f1 (ppm)

SI-14

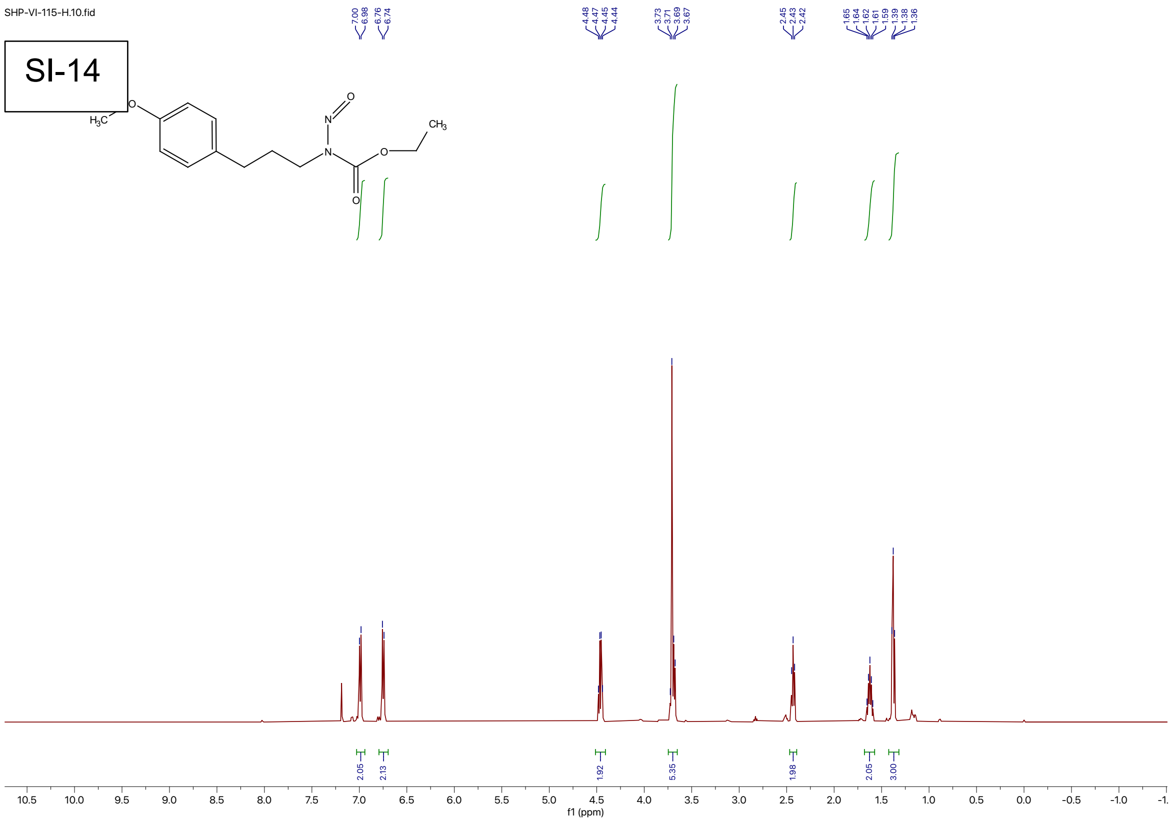
SHP-VI-115-H.10.fid
7.00
6.98
6.76
6.74
4.48
4.47
4.45
4.44
3.73
3.71
3.69
3.67
2.45
2.43
2.42
1.65
1.64
1.62
1.61
1.59
1.39
1.38
1.36
2.05
2.13
1.92
5.35
1.98
2.05
3.00
f1 (ppm)

SI-14

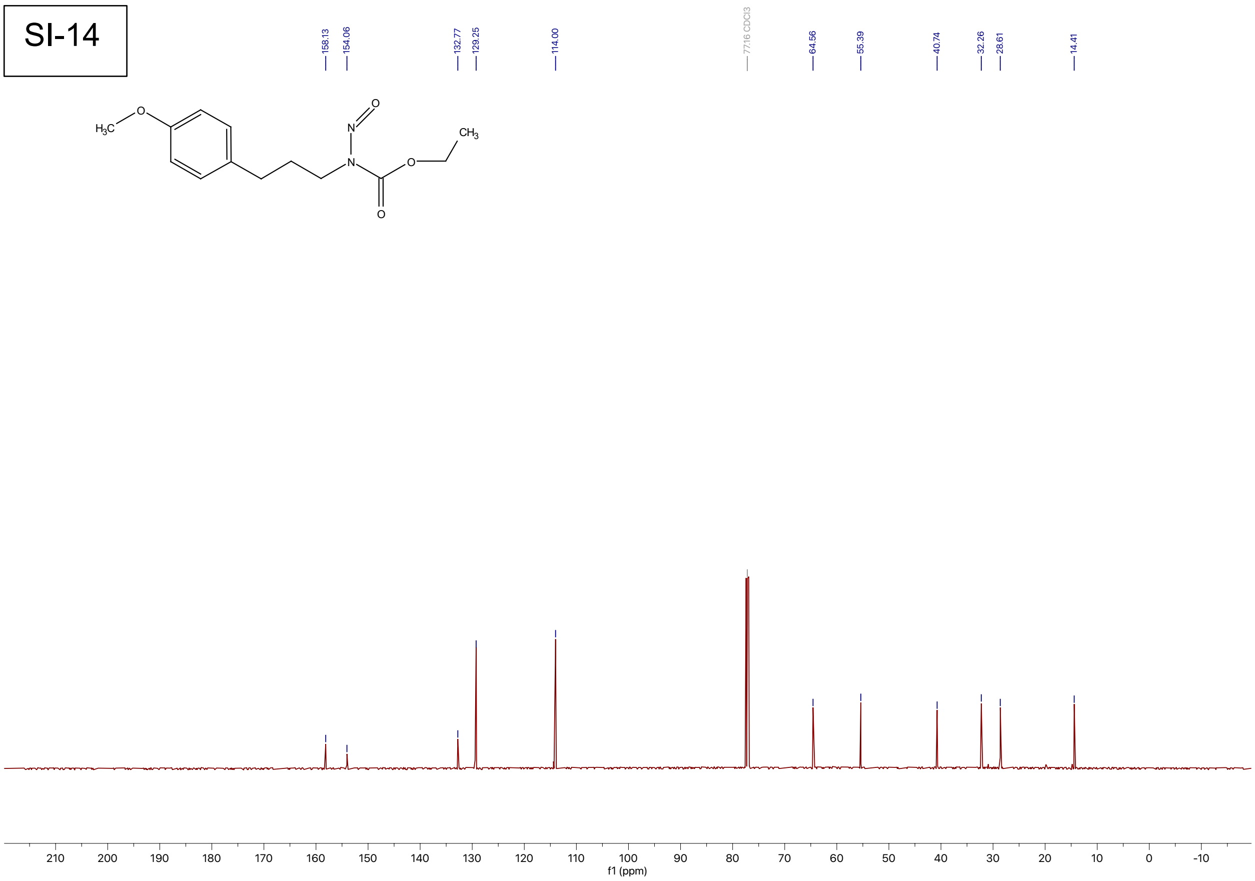
158.13
154.06
132.77
129.25
114.00
77.16 CDCl3
64.56
55.39
40.74
32.26
28.61
14.41
f1 (ppm)

SI-19

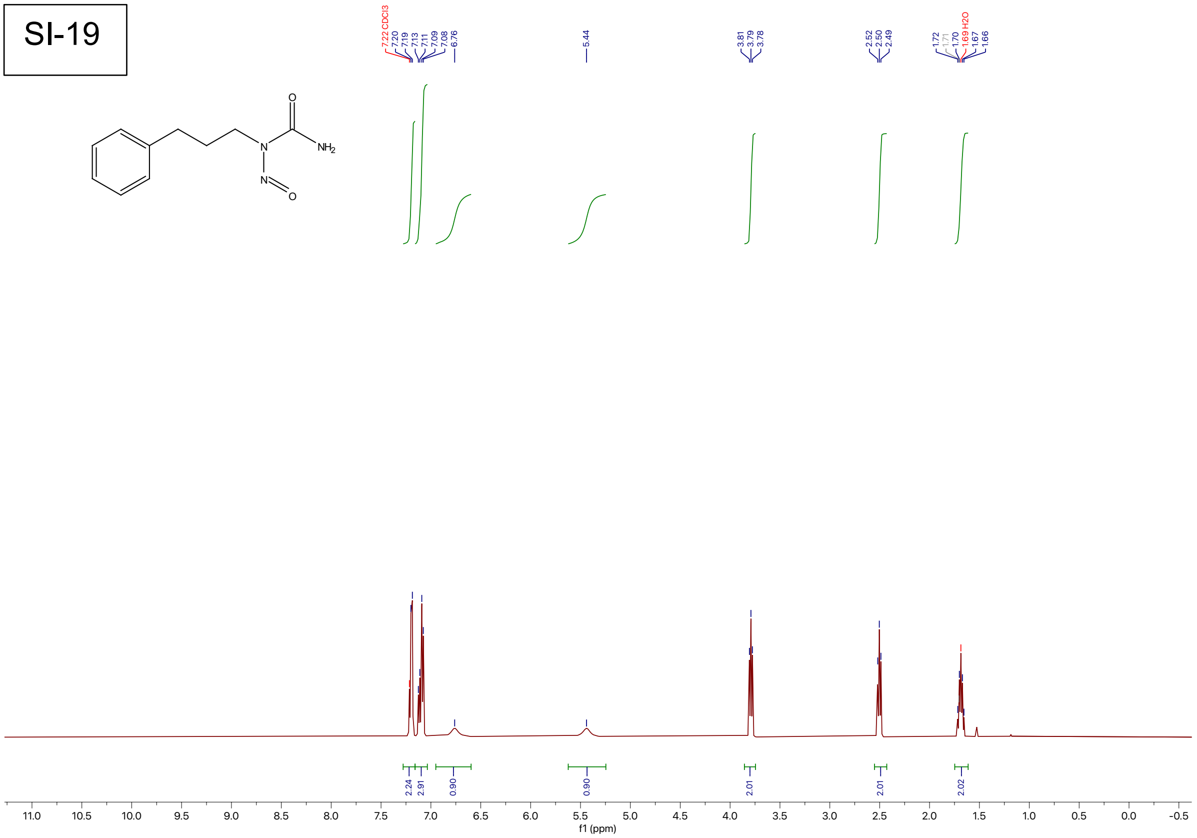


SI-19

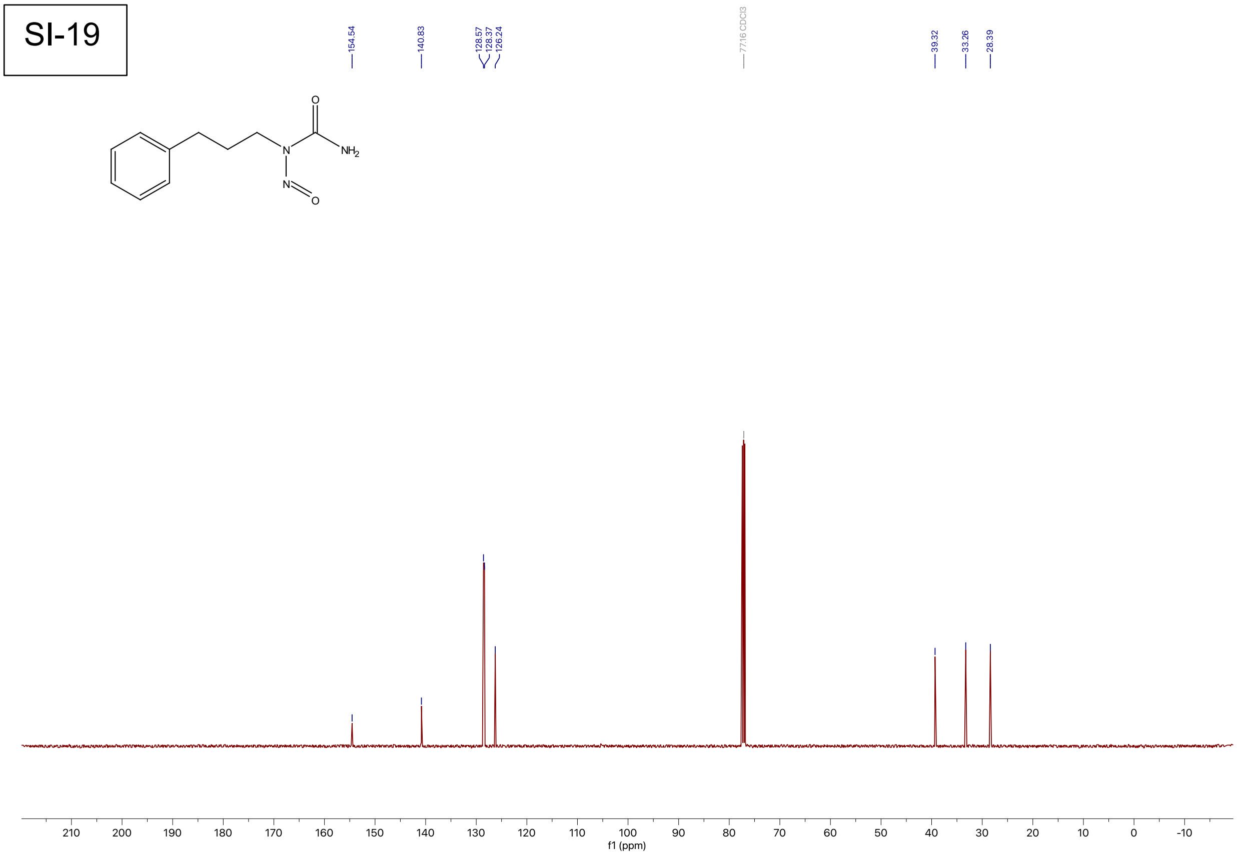

SI-13

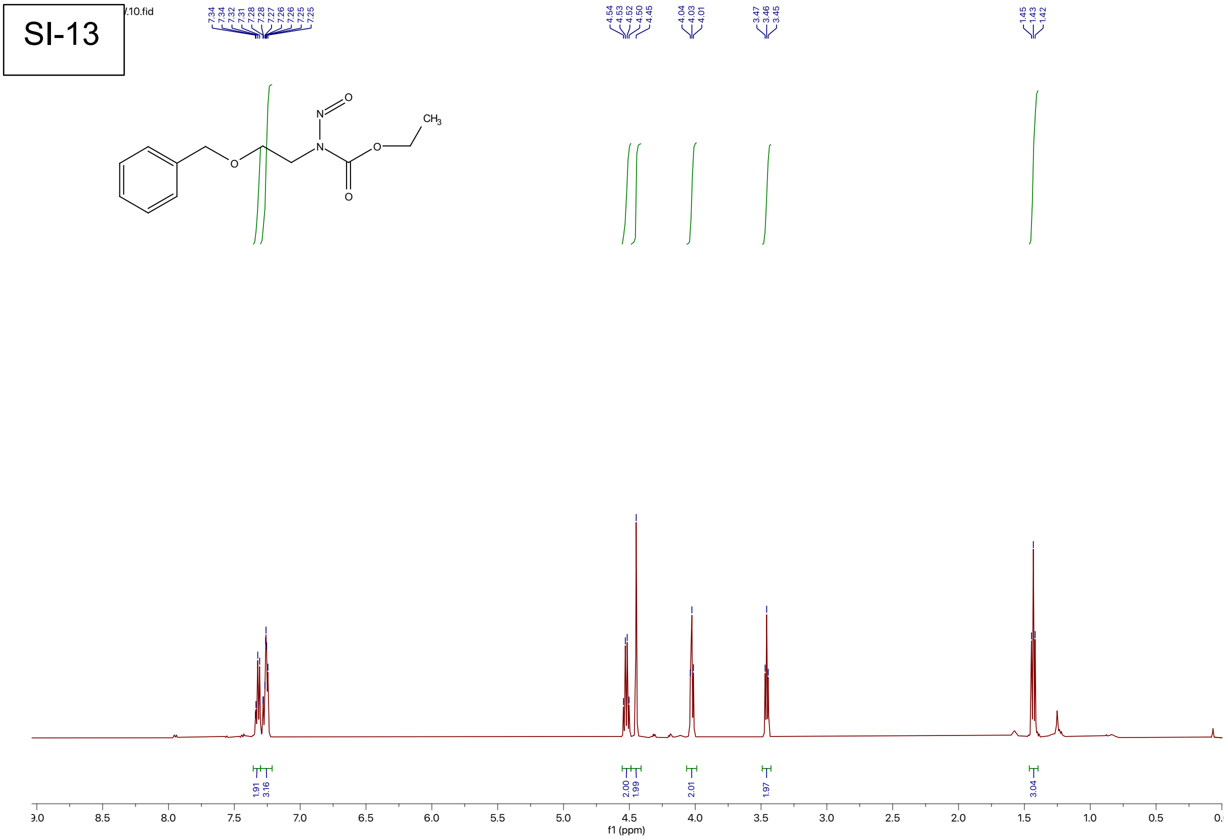


SHP-VI-106-C-NEW.12.fid

SI-13

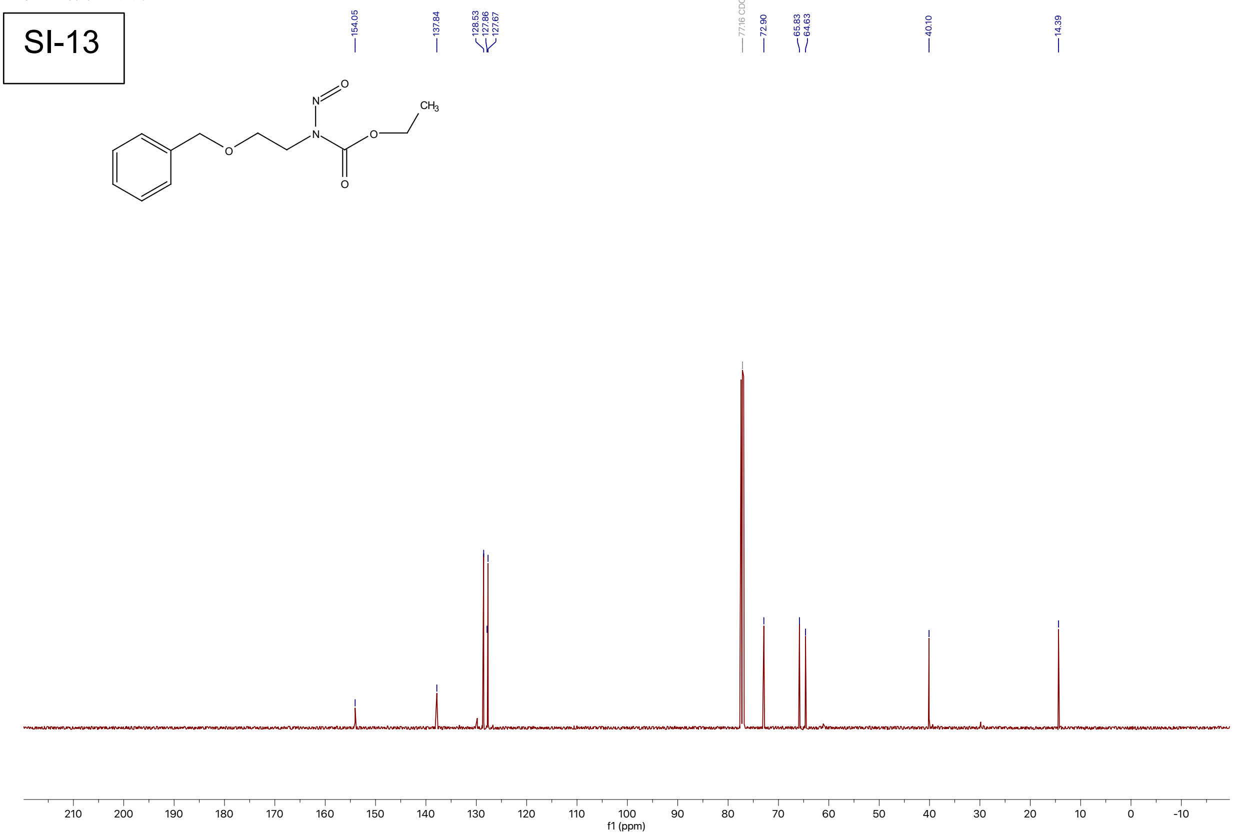

SI-15

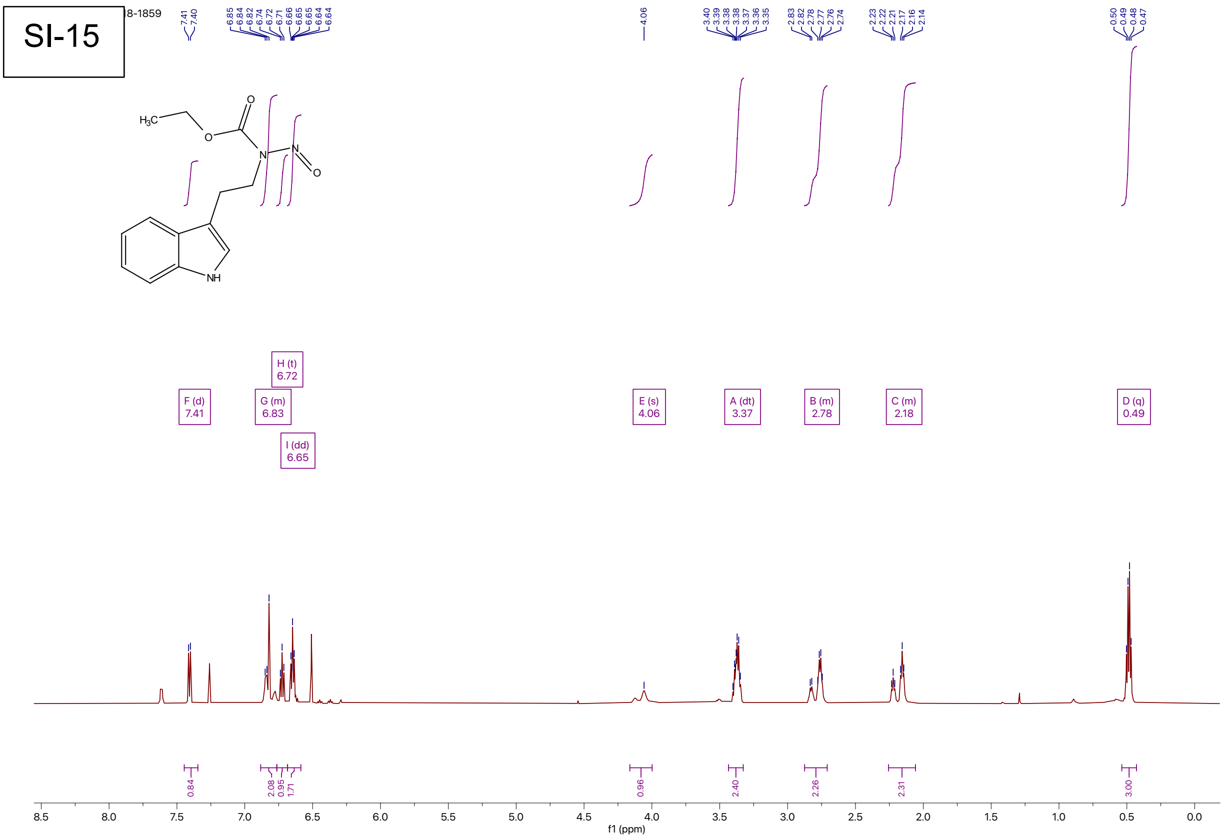

8-1859
H3C
O
O
N
N
O
NH
H (t)
6.72
F (d)
7.41
G (m)
6.83
I (dd)
6.65
E (s)
4.06
A (dt)
3.37
B (m)
2.78
C (m)
2.18
D (q)
0.49
f1 (ppm)


SI-15

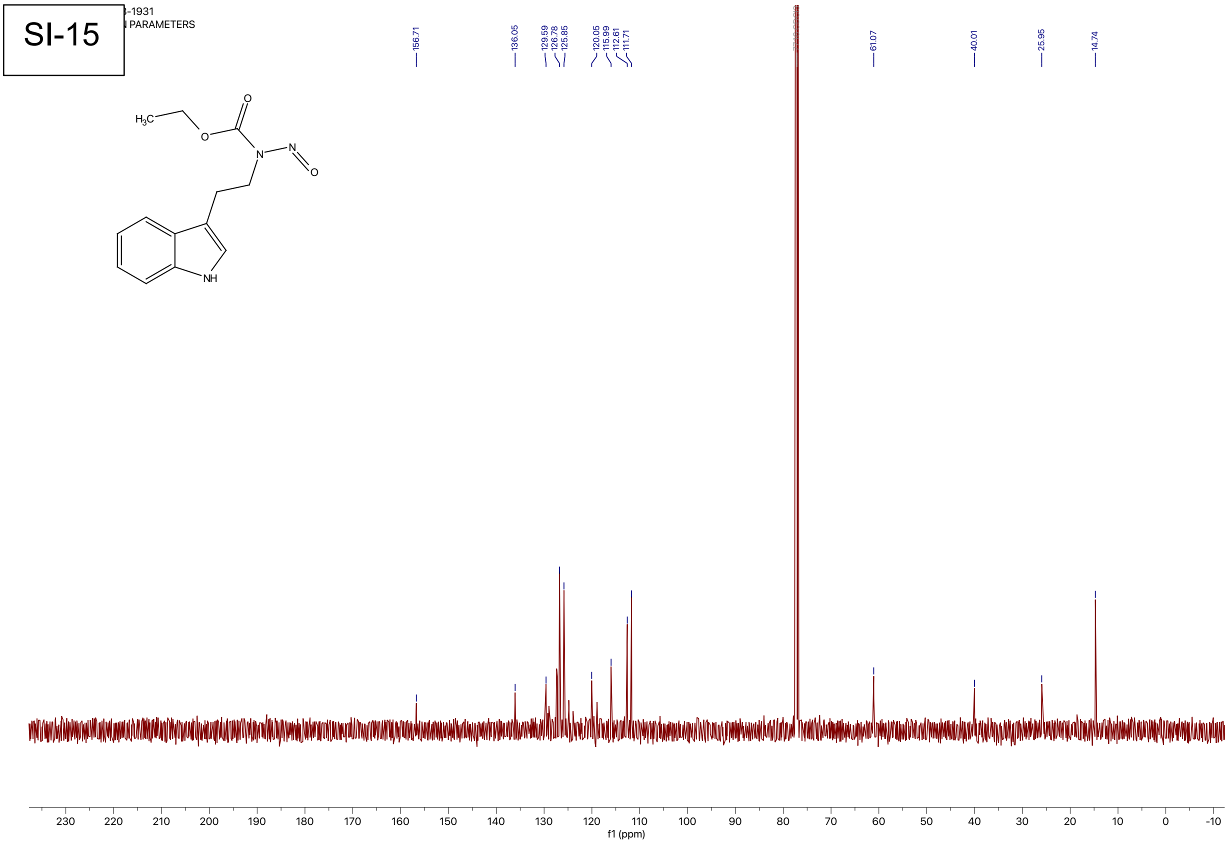

-1931
PARAMETERS
156.71
136.05
129.59
126.78
125.85
120.05
115.99
112.61
111.71
61.07
40.01
25.95
14.74
H3C
O
O
N
N
O
NH
f1 (ppm)

SI-16

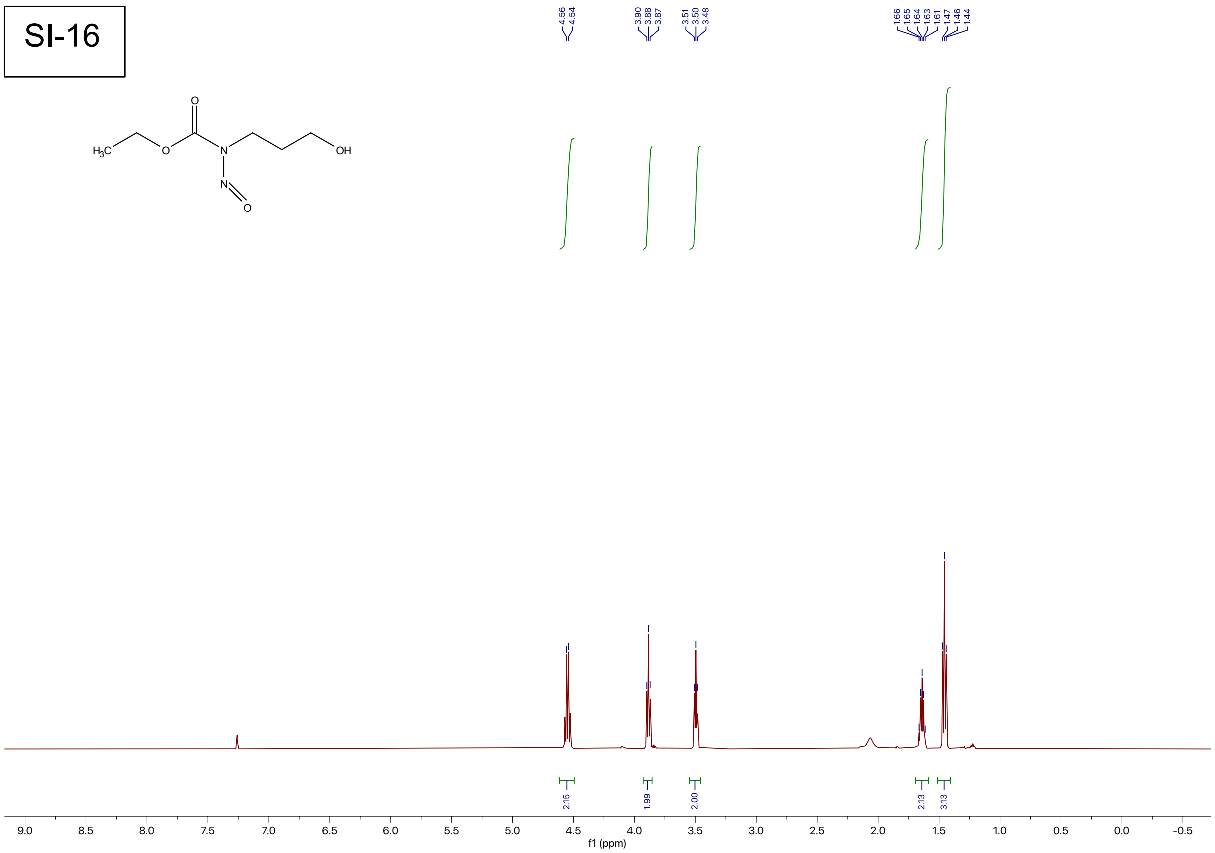

SI-16

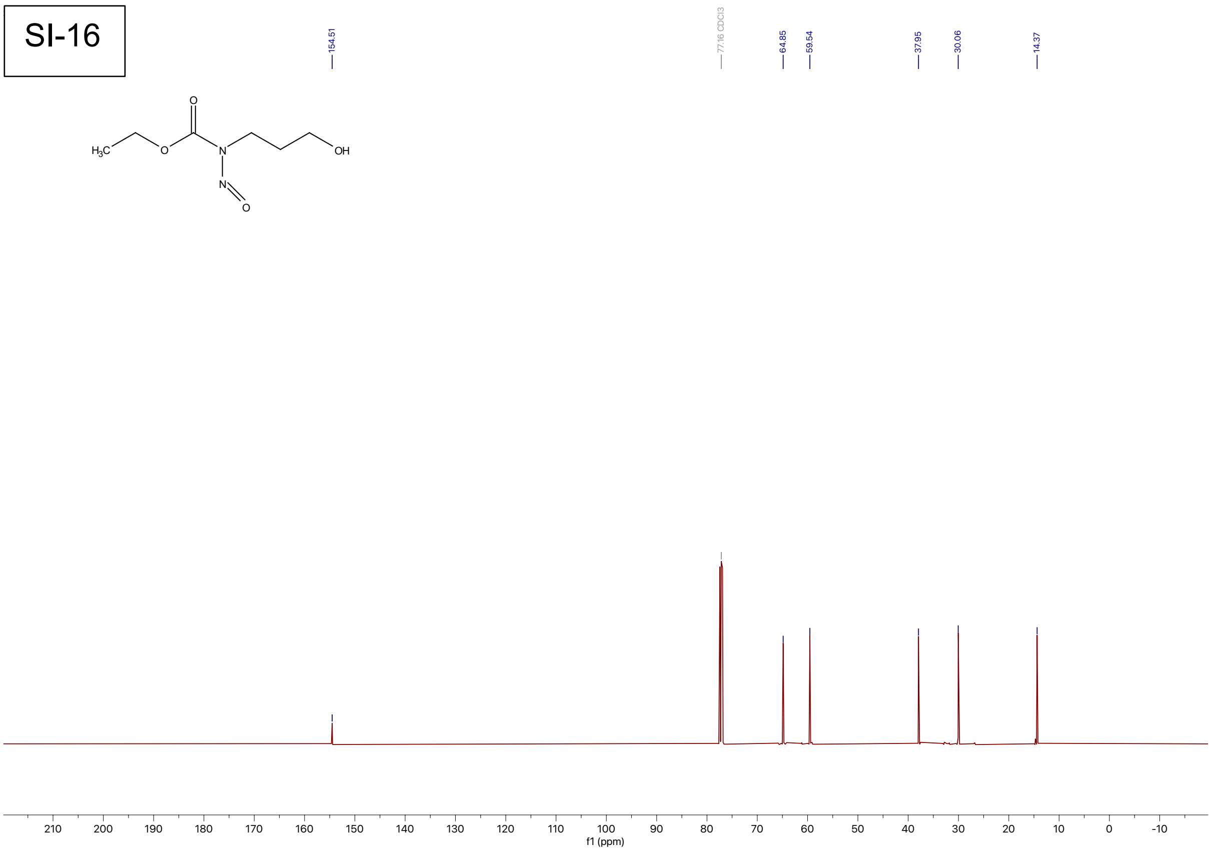

SI-17

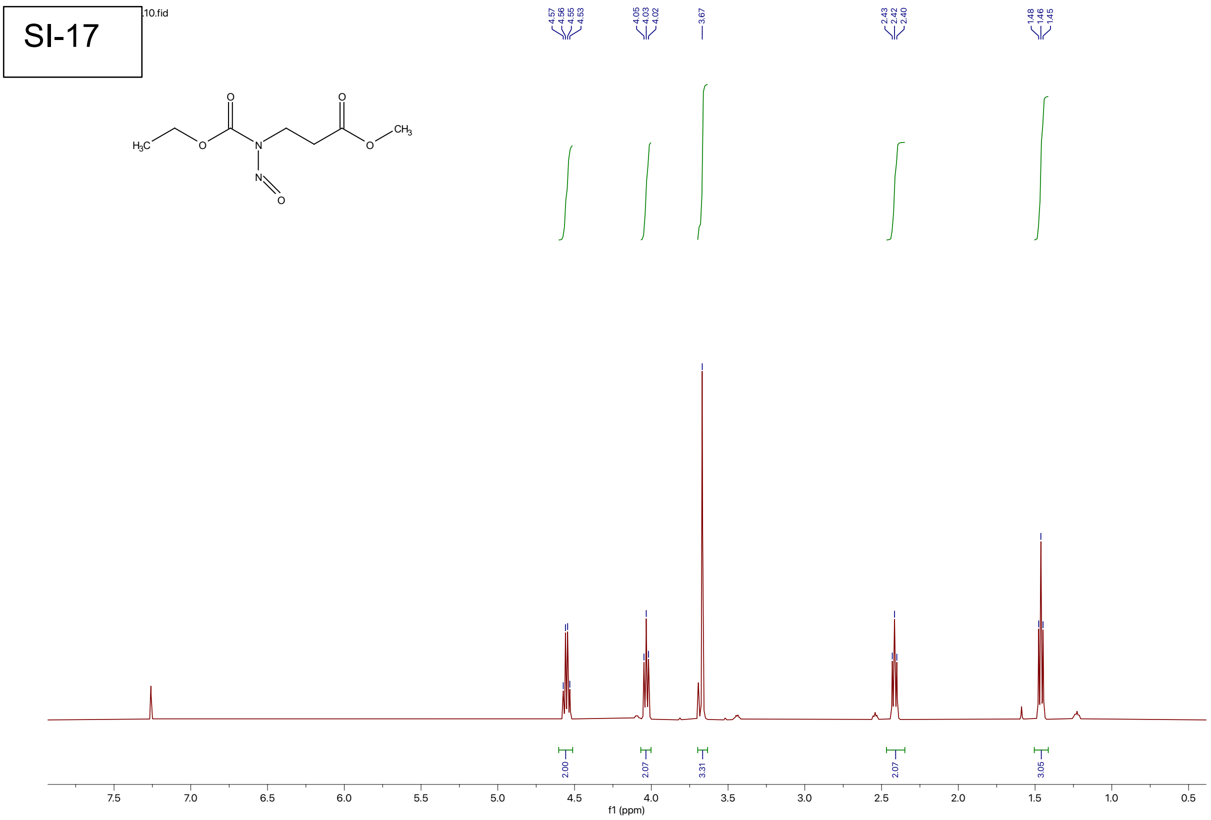

.10.fid
H3C
O
N
N
O
CH3
4.57
4.56
4.55
4.53
4.05
4.03
4.02
3.67
2.43
2.42
2.40
1.48
1.46
1.45
2.00
2.07
3.31
2.07
3.05
f1 (ppm)


SI-17

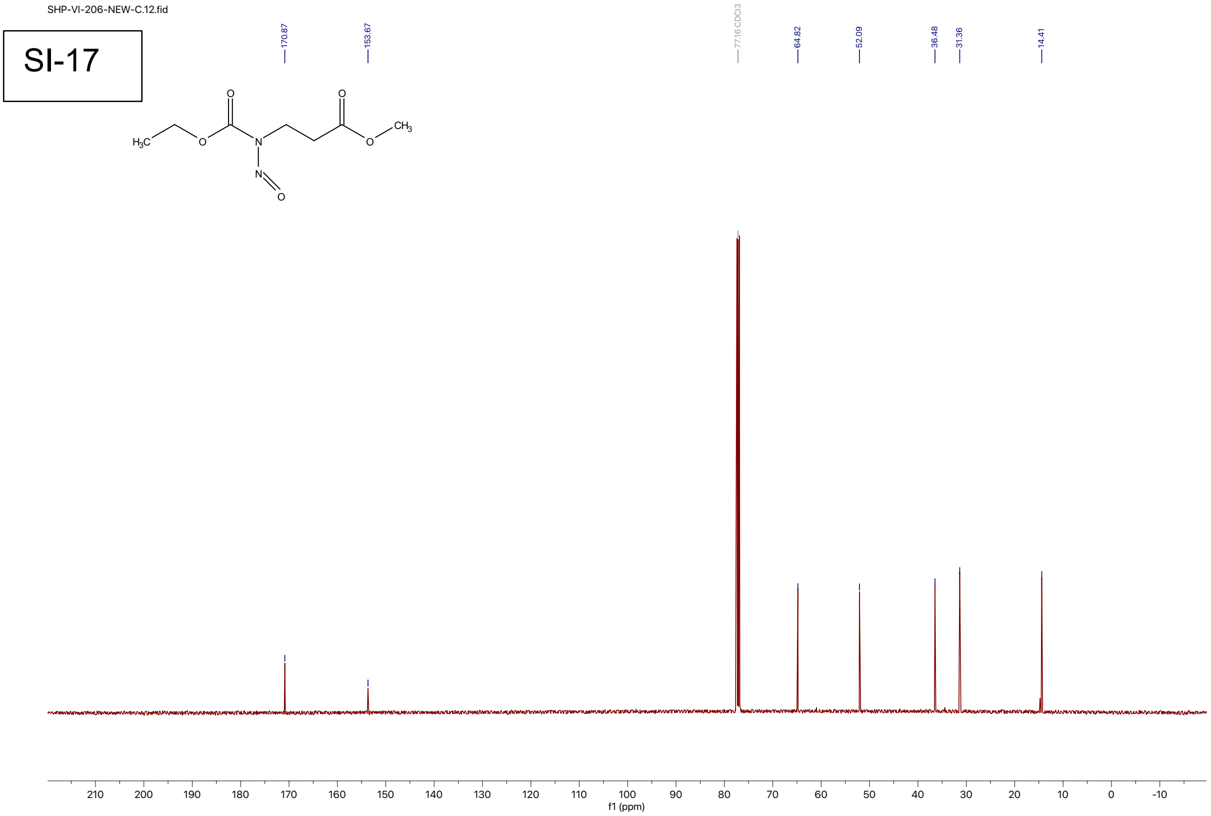

SHP-VI-206-NEW-C.12.fid
170.87
153.67
77.16 CDCl3
64.82
52.09
36.48
31.36
14.41
f1 (ppm)

36

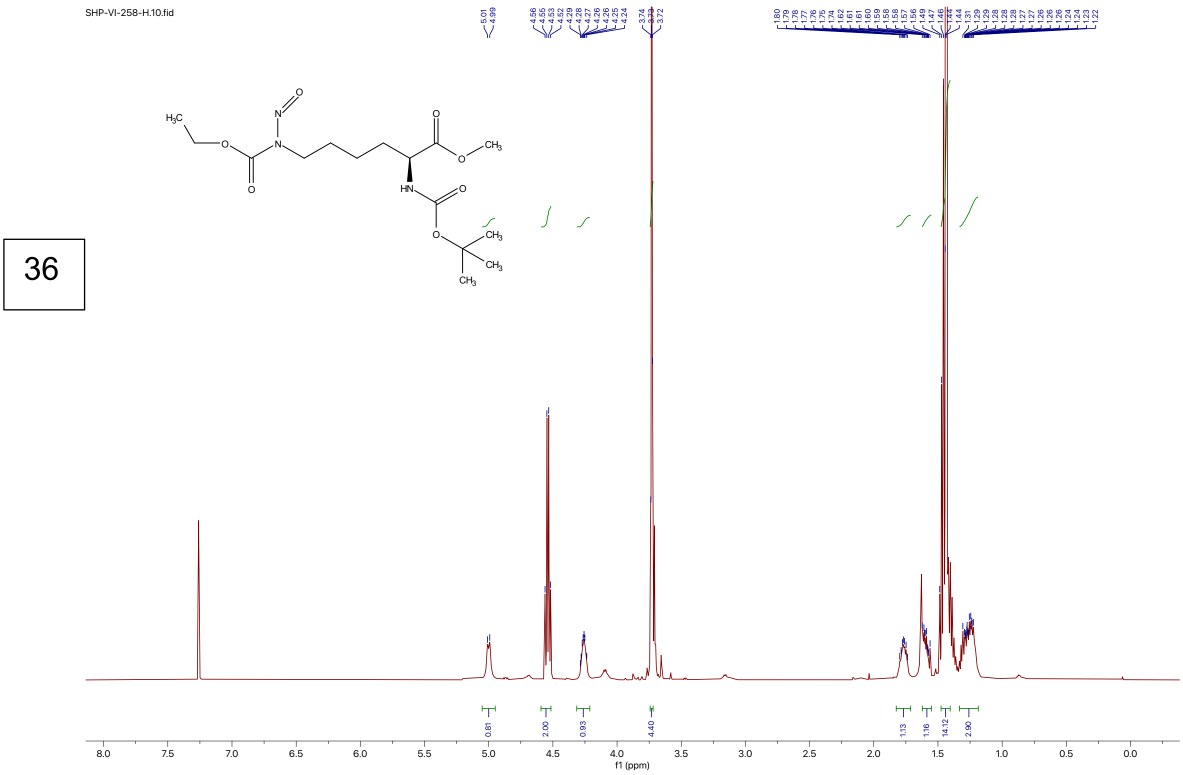

SHP-VI-258-H.10.fid
0.81
2.00
0.93
4.40
1.13
1.16
14.12
2.90
f1 (ppm)


36

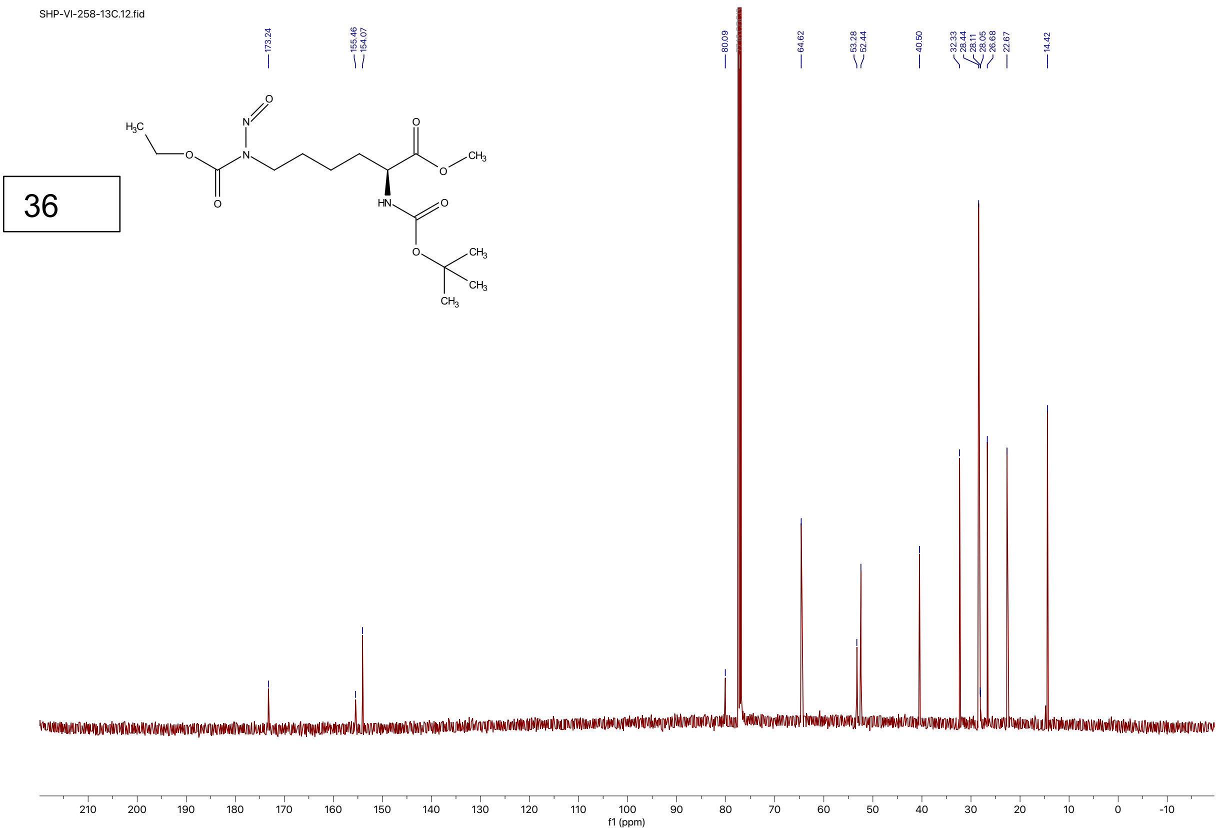

SHP-VI-258-13C.12.fid
173.24
155.46
154.07
80.09
64.62
53.28
52.44
40.50
32.33
28.44
28.11
28.05
26.68
22.67
14.42
f1 (ppm)

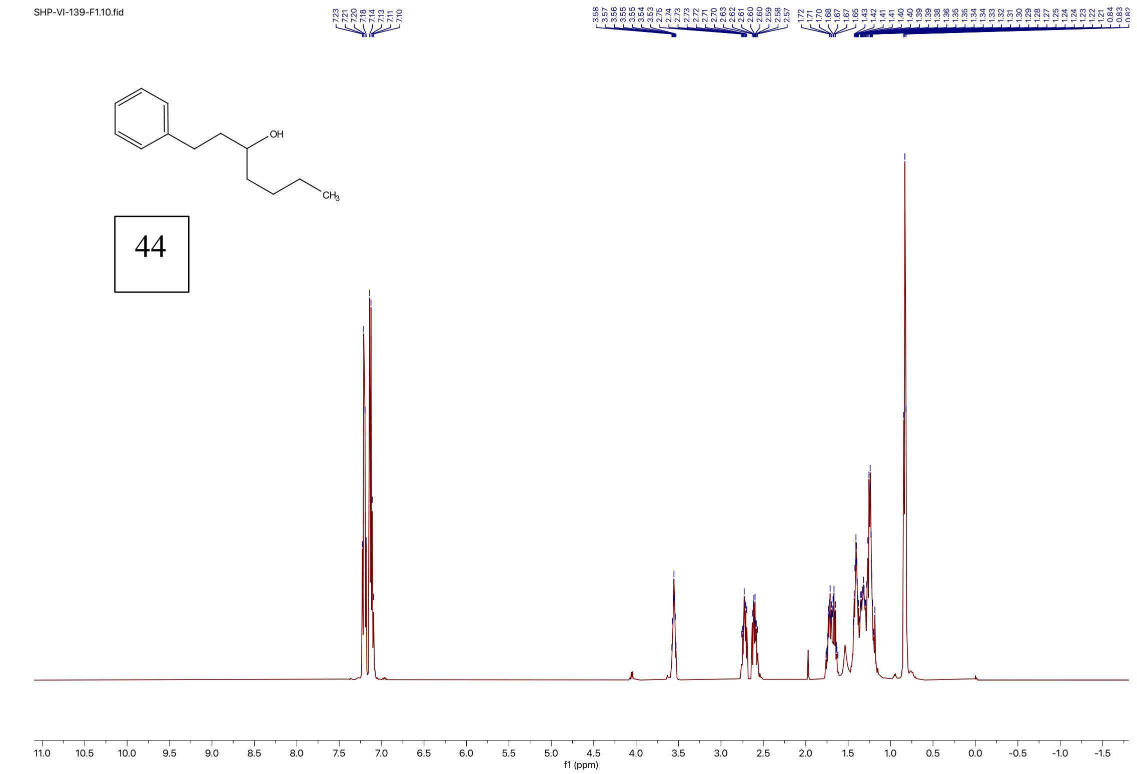
SHP-VI-139-F1.10.fid
OH
CH3
44
f1 (ppm)

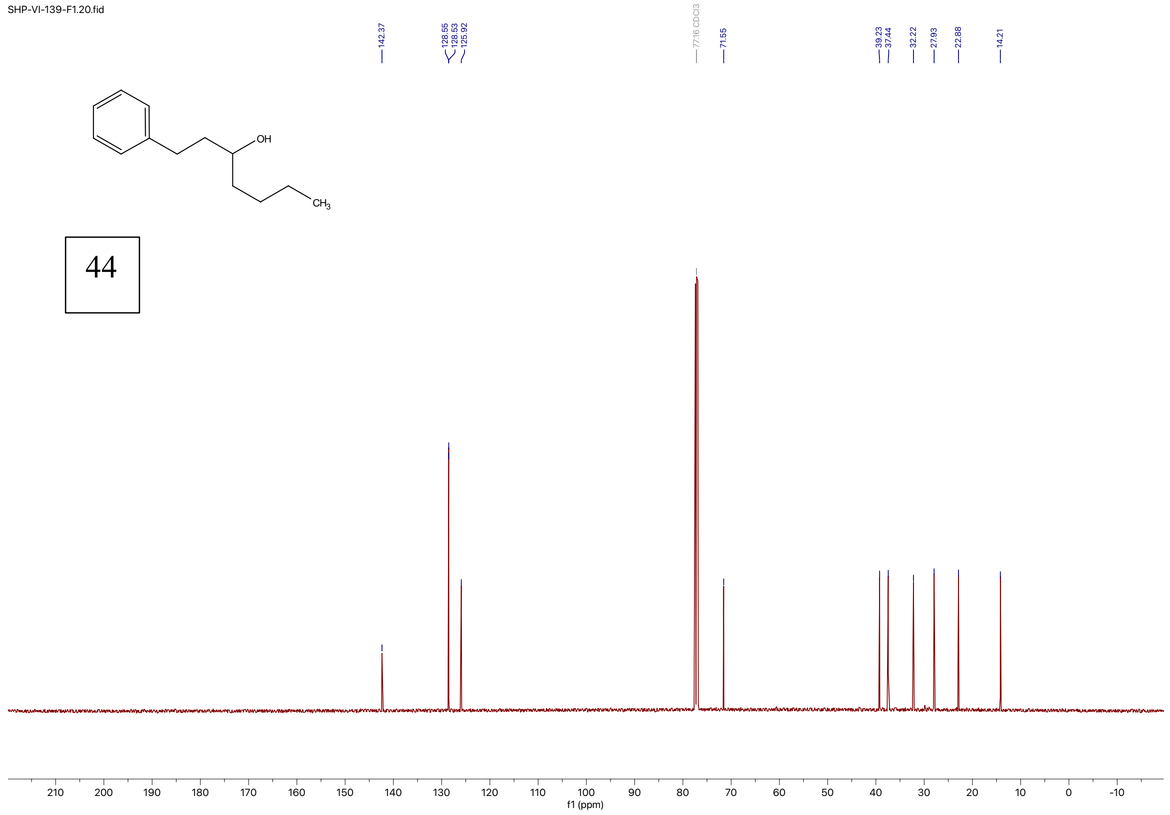
SHP-VI-139-F1.20.fid
142.37
128.55
128.53
125.92
77.16 CDCl3
71.55
39.23
37.44
32.22
27.93
22.88
14.21
OH
CH3
44
f1 (ppm)

6

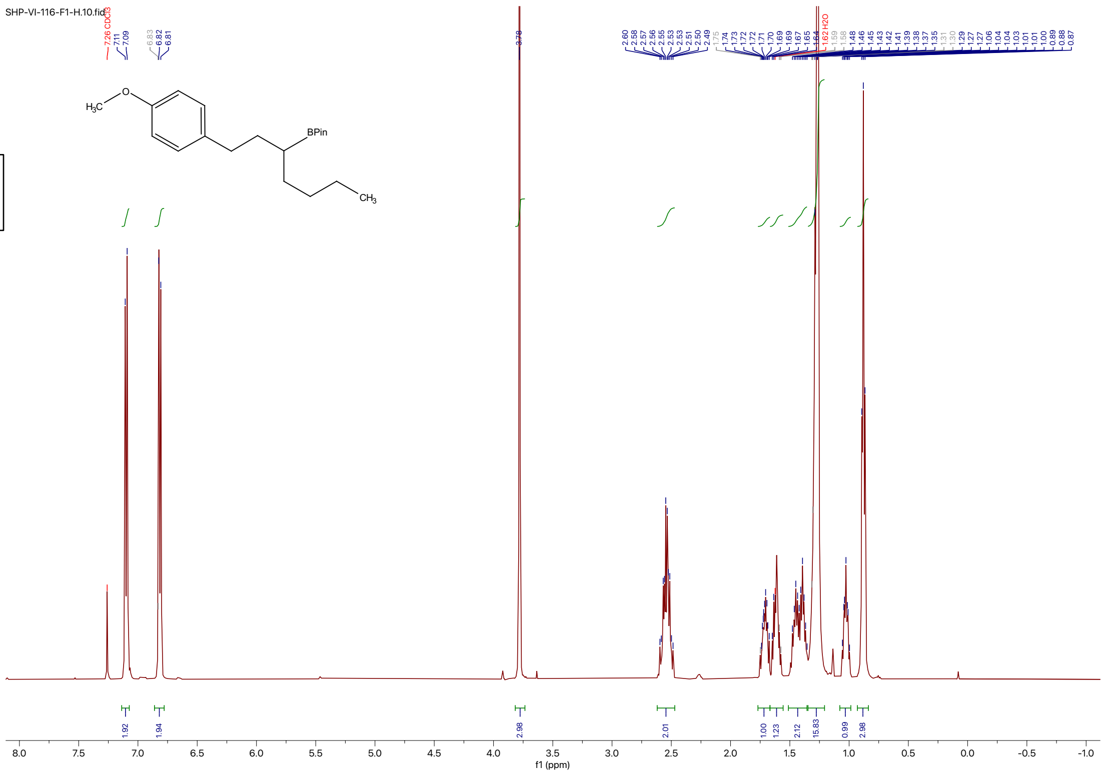
SHP-VI-116-F1-H.10.fid
7.26 CDCl3
7.11
7.09
6.83
6.82
6.81
3.79
1.62 H2O
H3C
O
BPin
CH3
1.92
1.94
2.98
2.01
1.00
1.23
2.12
15.83
0.99
2.98
f1 (ppm)

6

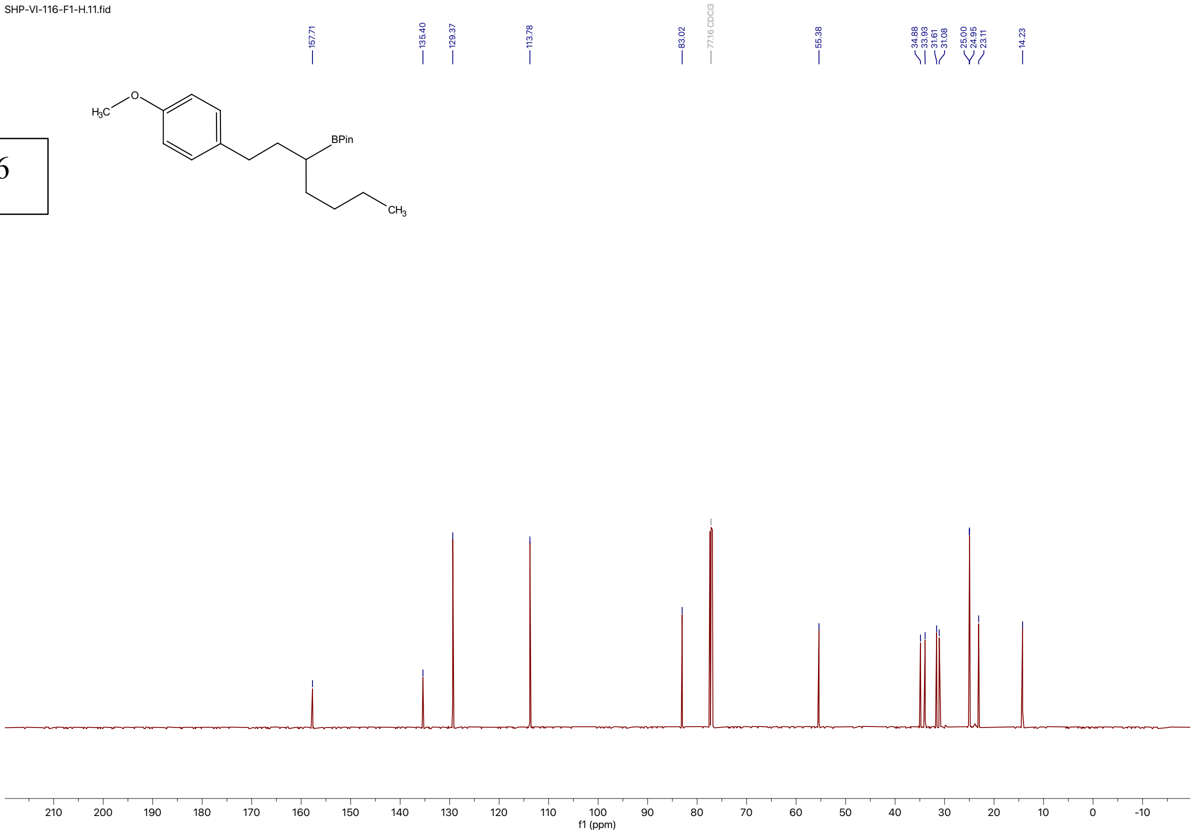
SHP-VI-116-F1-H.11.fid
157.71
135.40
129.37
113.78
83.02
77.16 CDCl3
55.38
34.88
33.93
31.61
31.08
25.00
24.95
23.11
14.23
H3C
O
BPin
CH3
f1 (ppm)

7

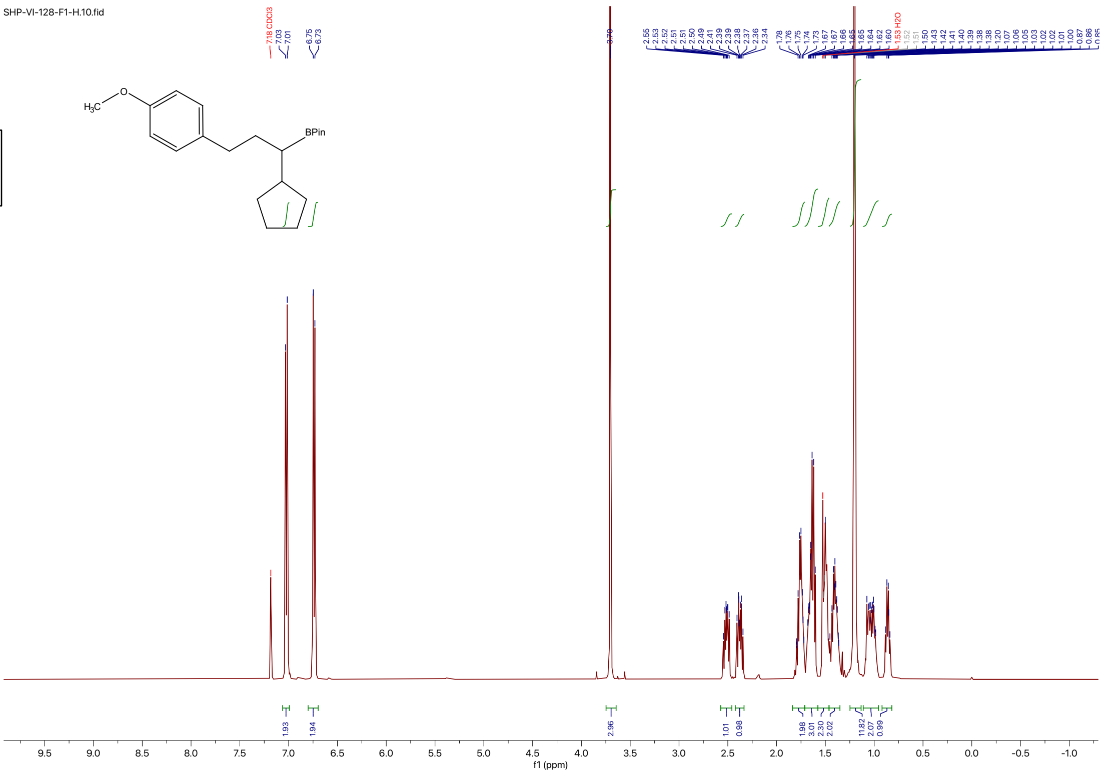
SHP-VI-128-F1-H.10.fid
H3C
O
BPin
f1 (ppm)

7

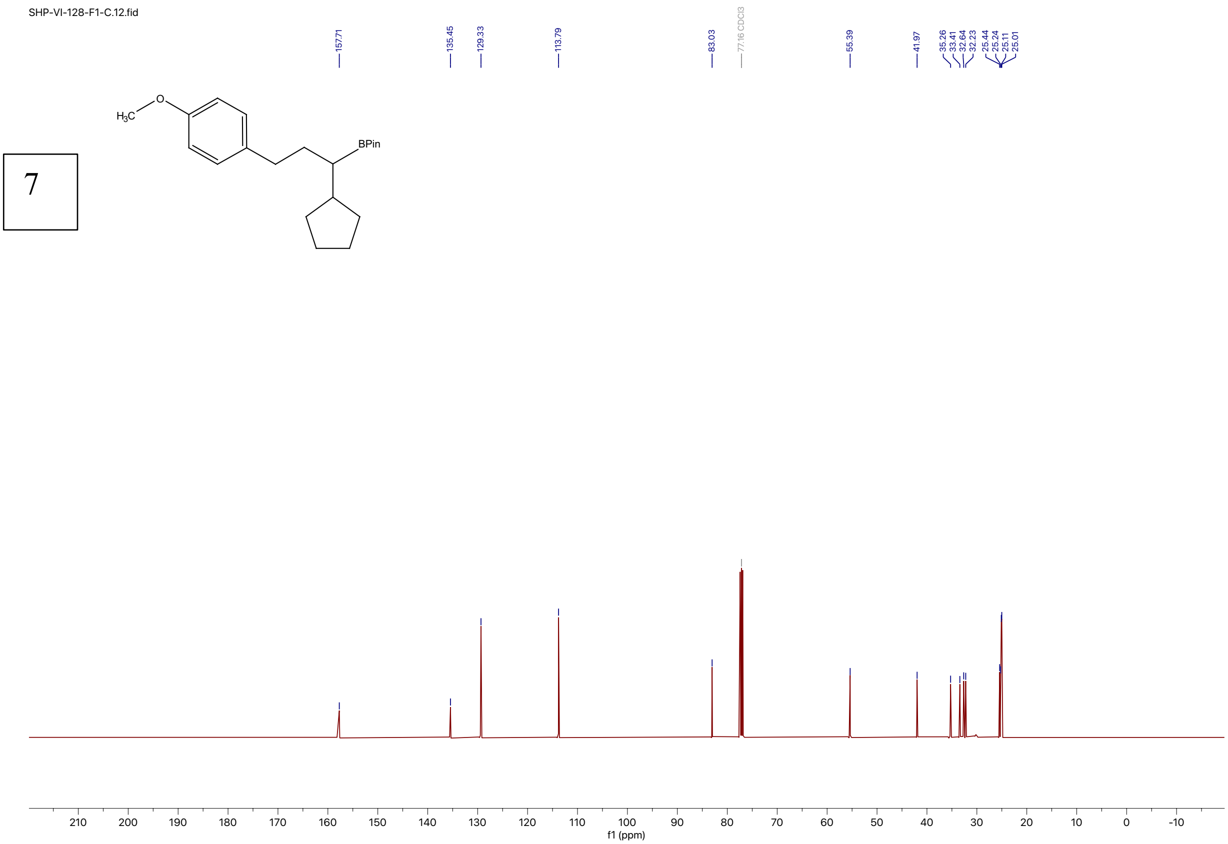
SHP-VI-128-F1-C.12.fid
157.71
135.45
129.33
113.79
83.03
77.16 CDCl3
55.39
41.97
H3C
O
BPin
f1 (ppm)

8

SHP-VI-73-H.10.fid

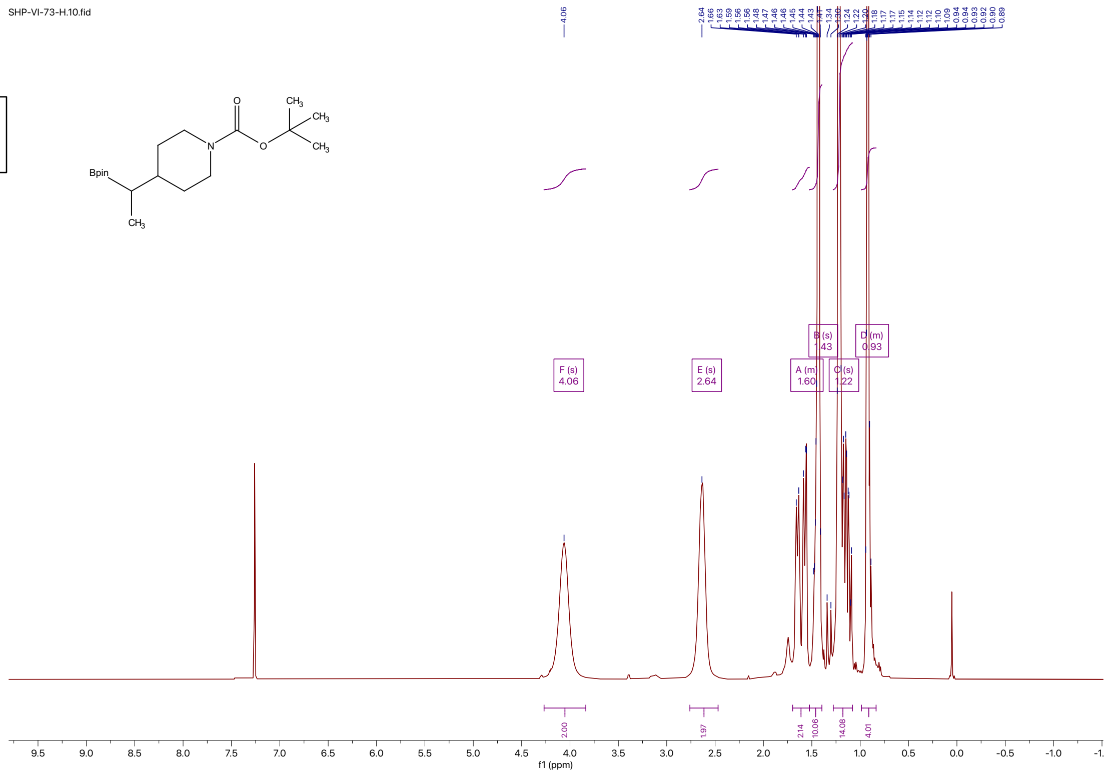


8

SHP-VI-73-C.10.fid

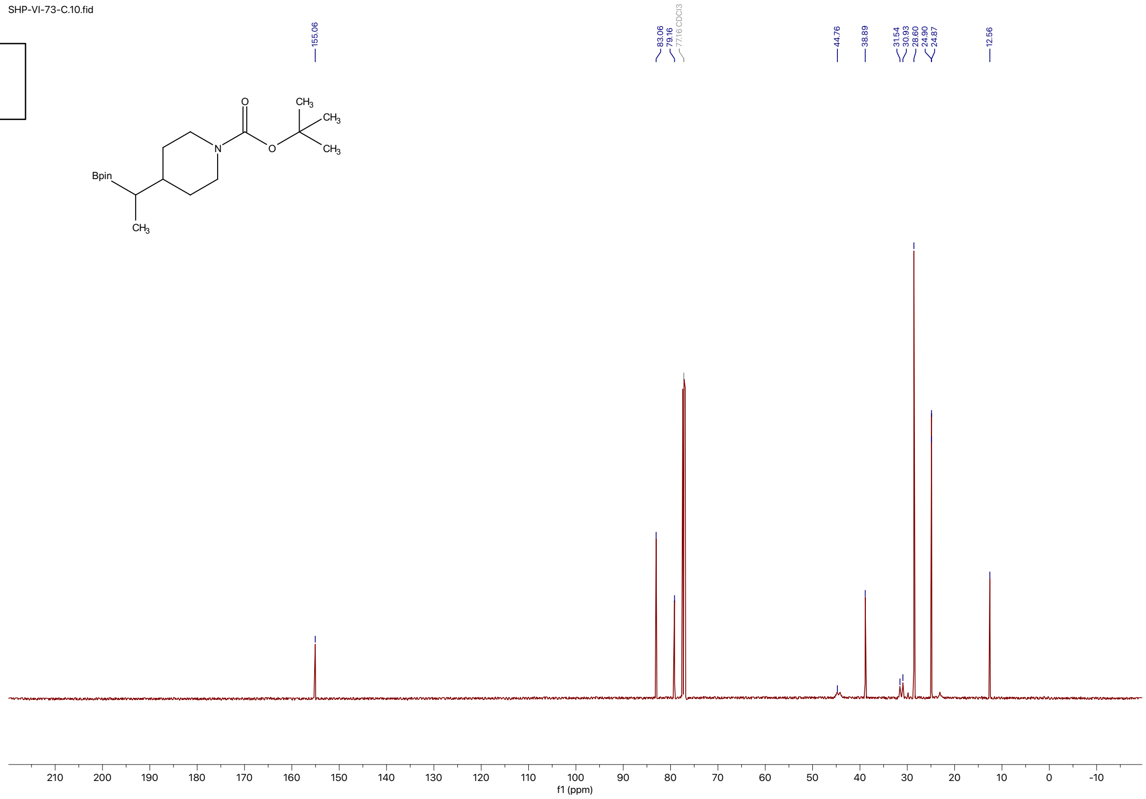

9

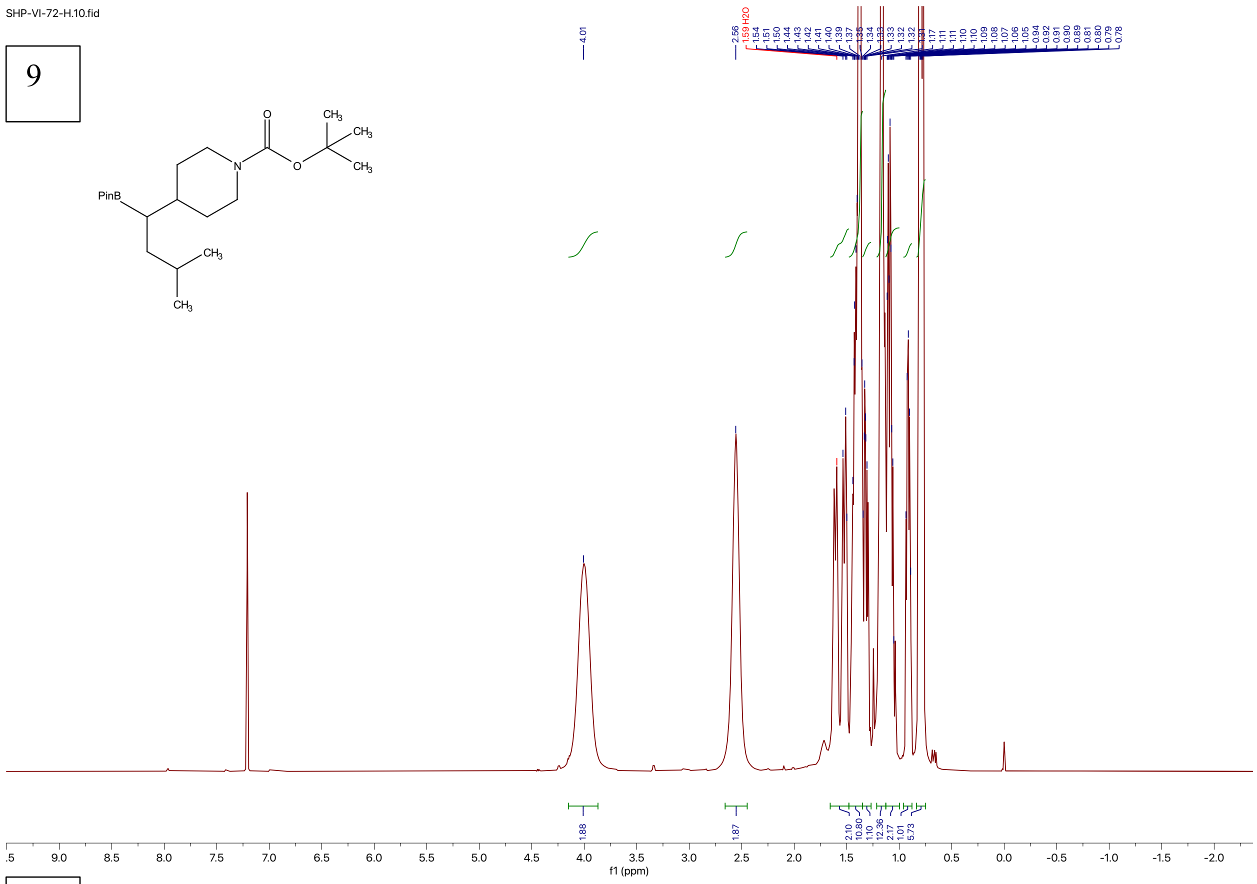
SHP-VI-72-H.10.fid
PinB
O
N
O
CH3
CH3
CH3
CH3
CH3
4.01
2.56
1.59 H2O
1.54
1.51
1.50
1.44
1.43
1.42
1.41
1.40
1.39
1.37
1.35
1.34
1.33
1.33
1.32
1.32
1.17
1.11
1.11
1.10
1.10
1.09
1.08
1.07
1.06
1.05
0.94
0.92
0.91
0.90
0.89
0.81
0.80
0.79
0.78
1.88
1.87
2.10
10.80
1.10
12.36
2.17
1.01
5.73
f1 (ppm)

9

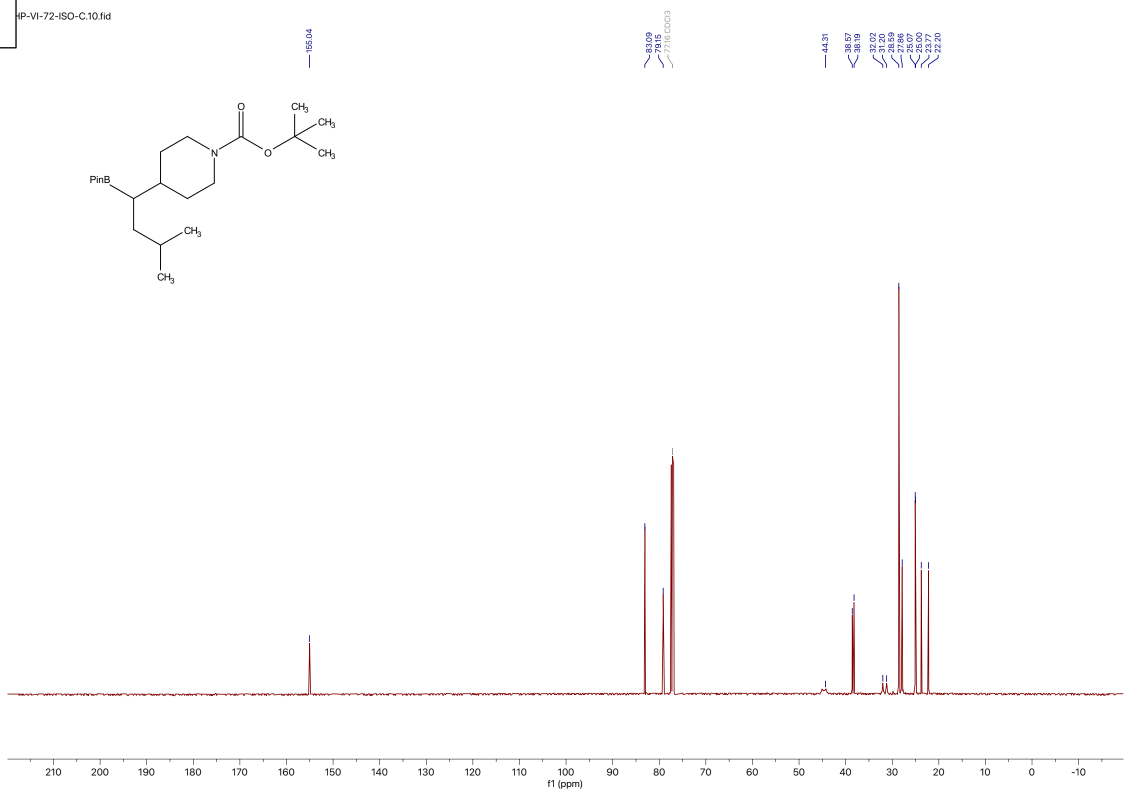
HP-VI-72-ISO-C.10.fid
PinB
O
N
O
CH3
CH3
CH3
CH3
CH3
155.04
83.09
79.15
77.16 CDCl3
44.31
38.57
38.19
32.02
31.20
28.59
27.86
25.07
25.00
23.77
22.20
f1 (ppm)

SHP-VI-71-F1.10.fid

10

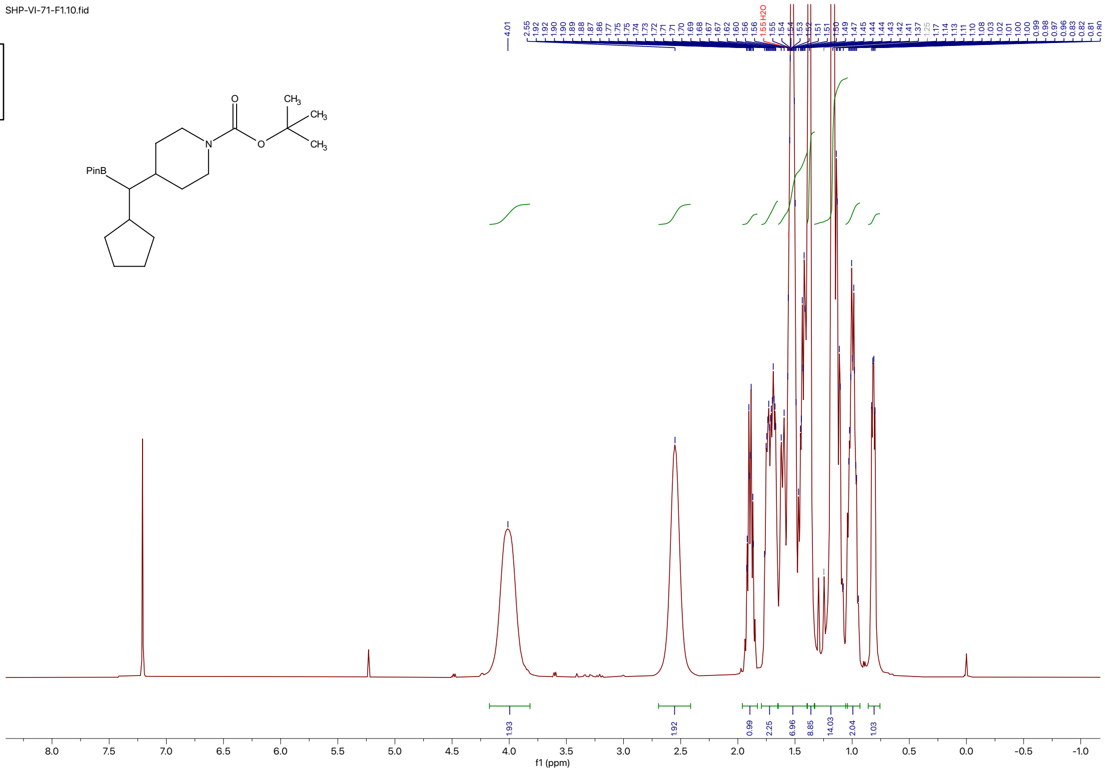


SHP-VI-71-C.10.fid

10

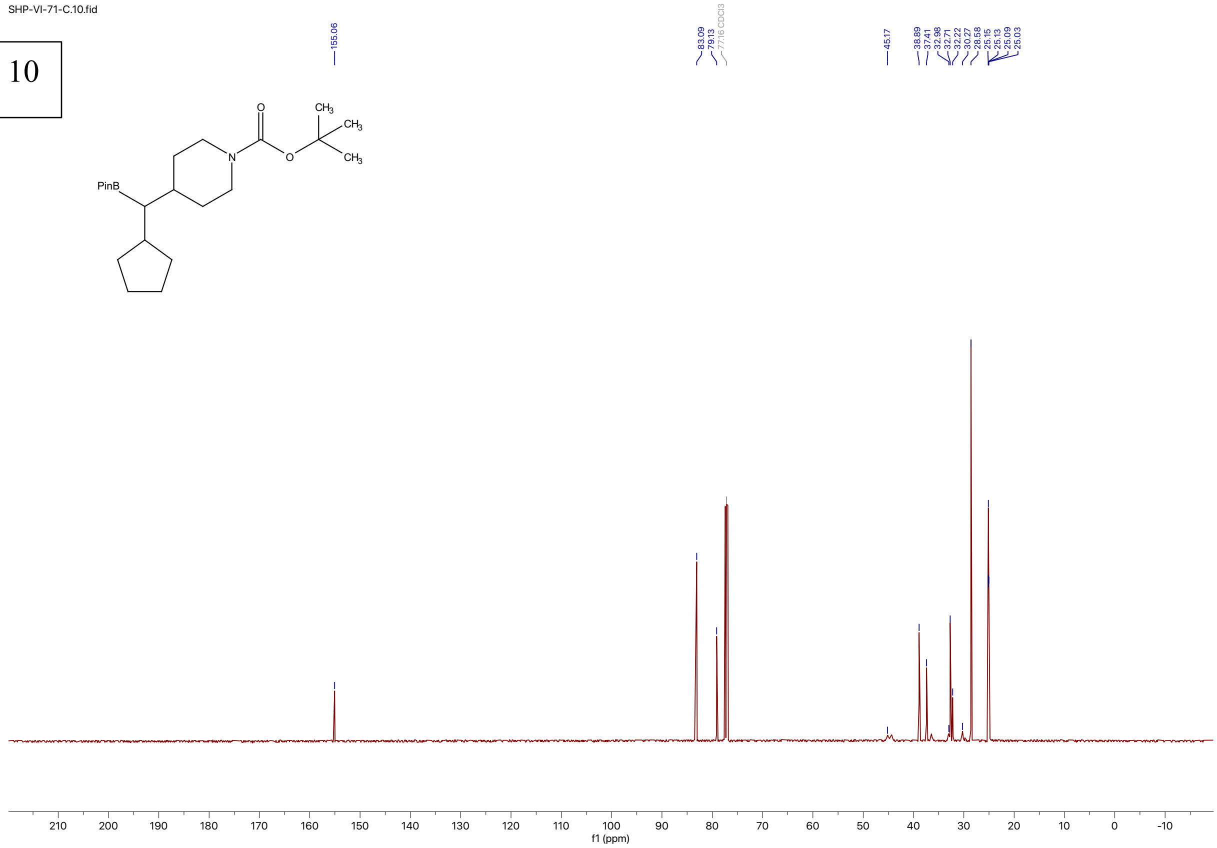

11

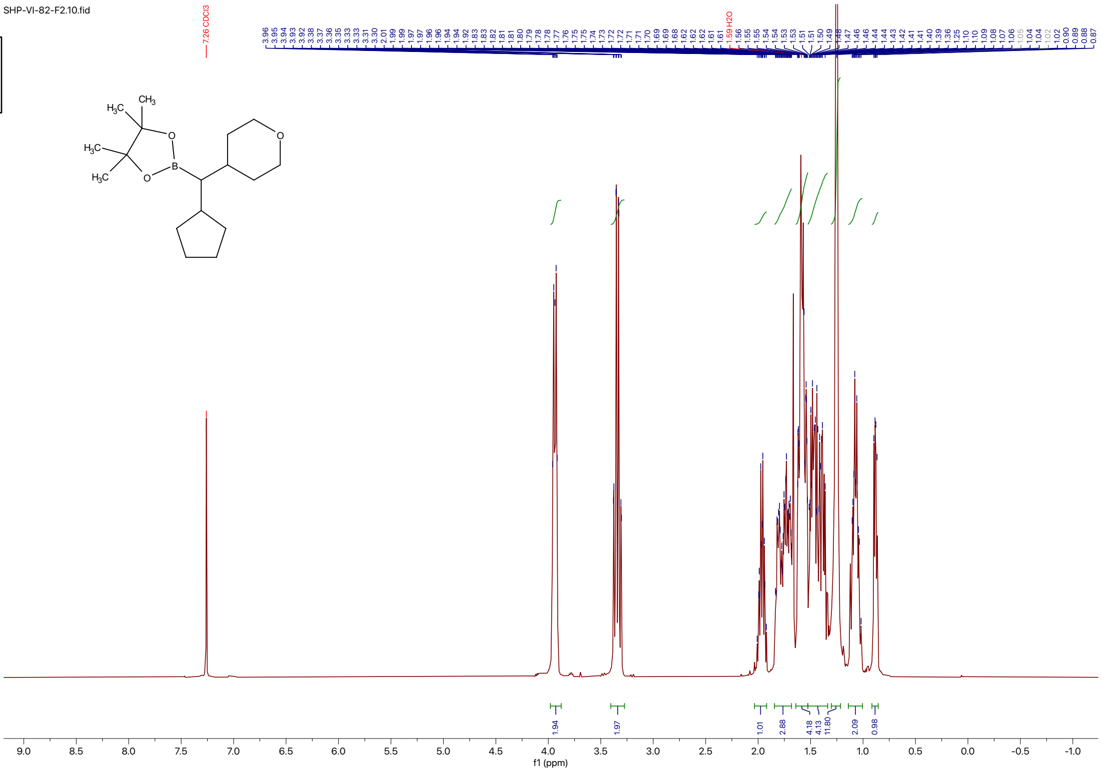
SHP-VI-82-F2.10.fid
7.26 CDCl3
1.59 H2O
1.94
1.97
1.01
2.88
4.18
4.13
11.80
2.09
0.98
f1 (ppm)

11

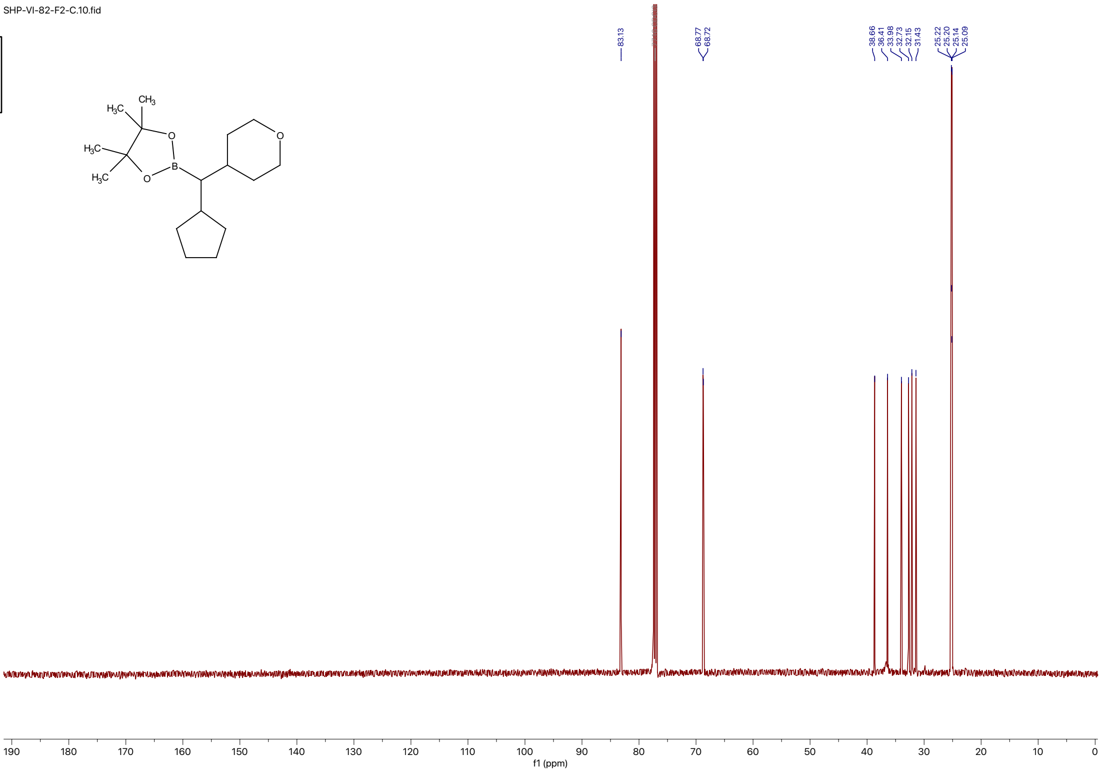
SHP-VI-82-F2-C.10.fid
83.13
68.77
68.72
38.66
36.41
33.98
32.73
32.15
31.43
25.22
25.20
25.14
25.09
f1 (ppm)

12

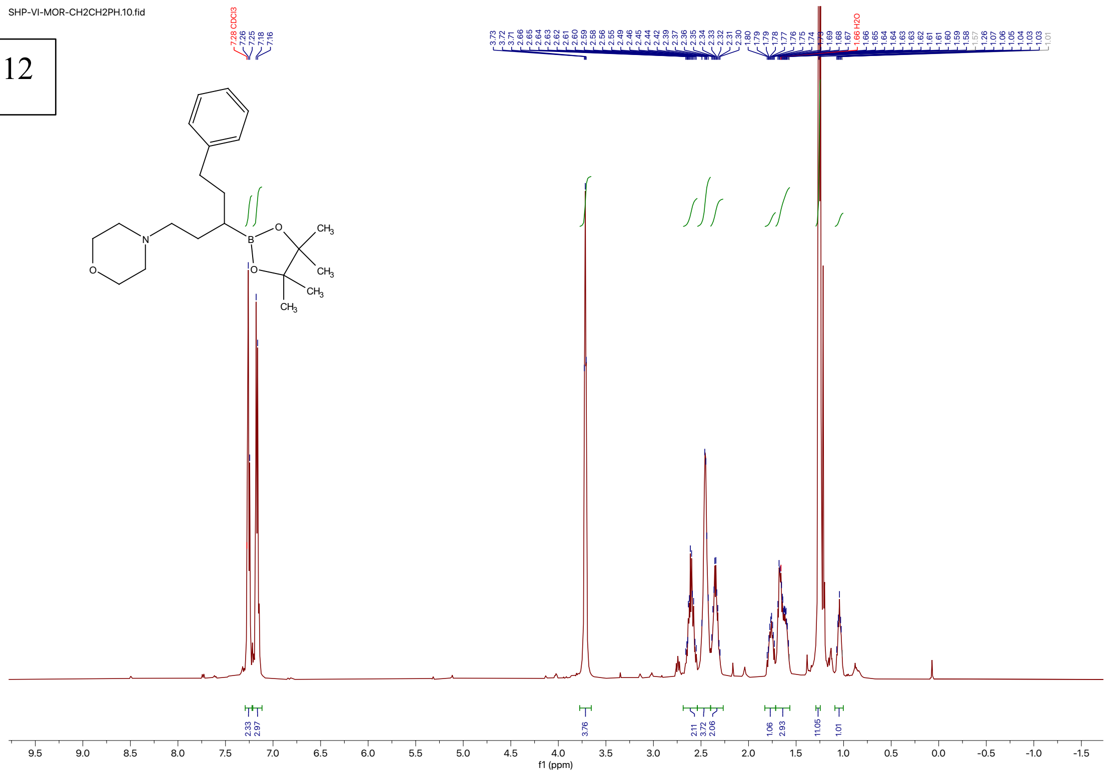
SHP-VI-MOR-CH2CH2PH.10.fid
2.33
2.97
3.76
2.11
3.72
2.06
1.06
2.93
11.05
1.01
f1 (ppm)

12

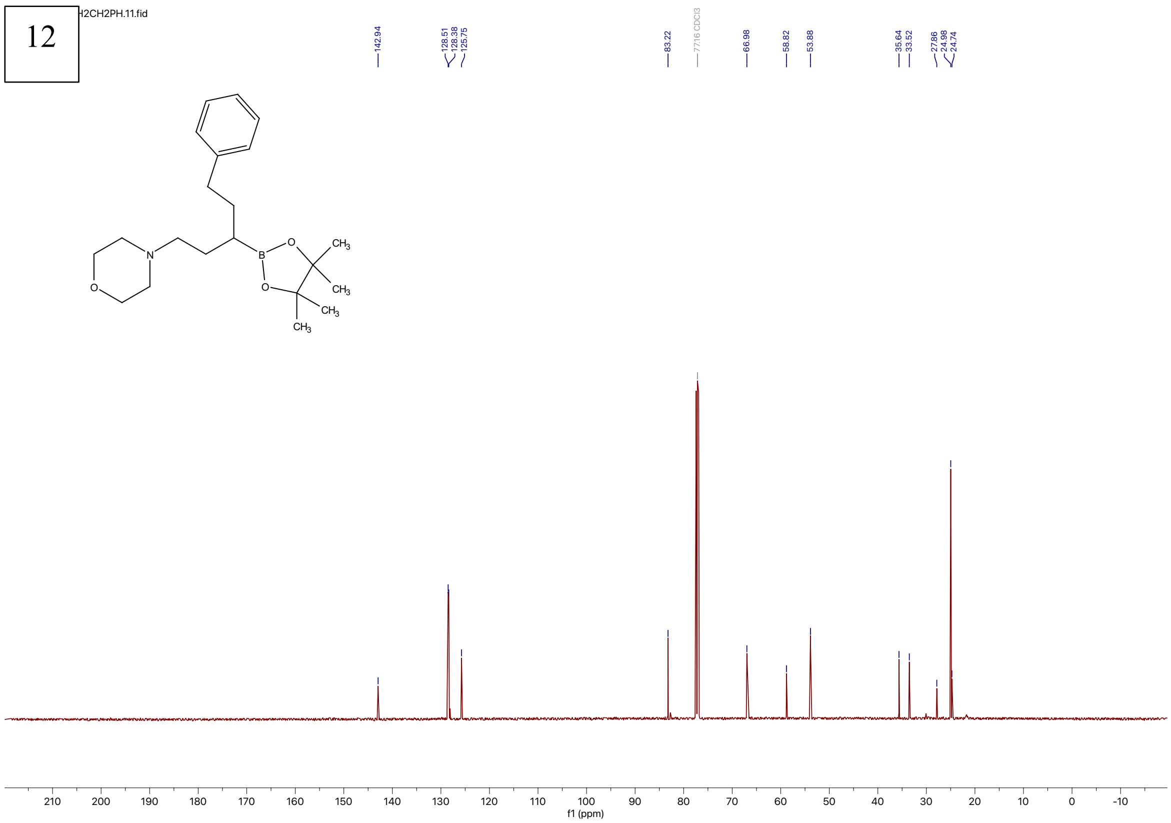
H2CH2PH.11.fid
142.94
128.51
128.38
125.75
83.22
77.16 CDCl3
66.98
58.82
53.88
35.64
33.52
27.86
24.98
24.74
f1 (ppm)

13

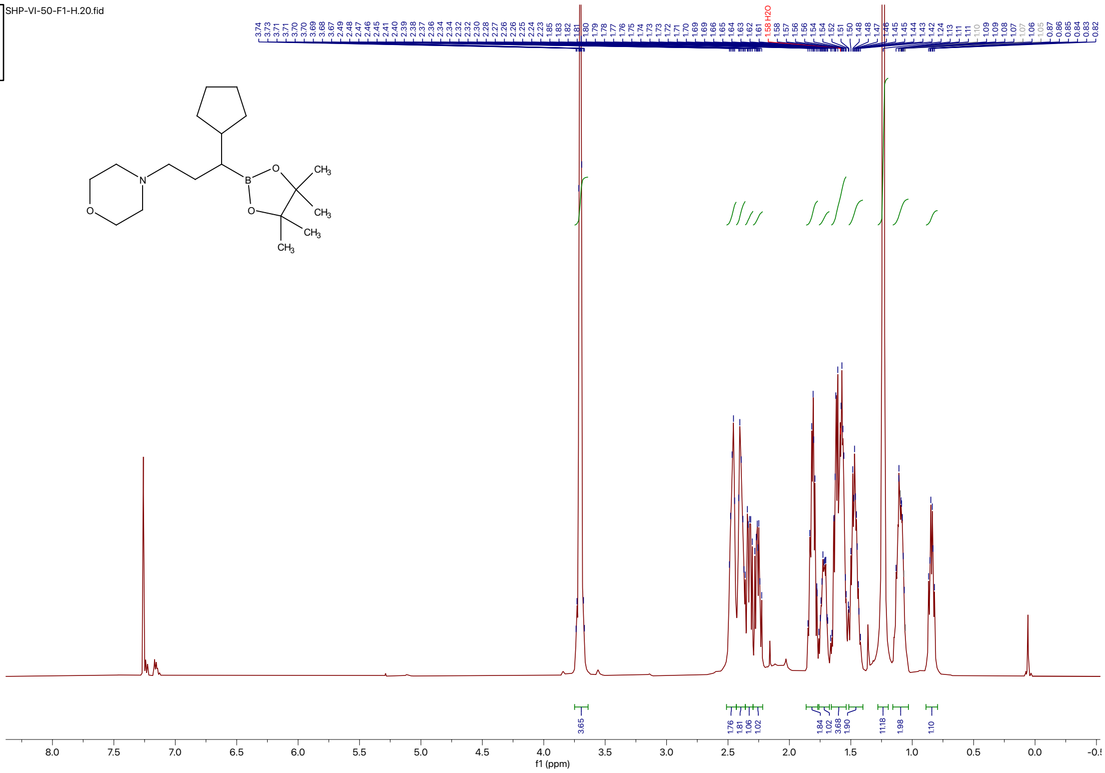
SHP-VI-50-F1-H.20.fid
f1 (ppm)

13

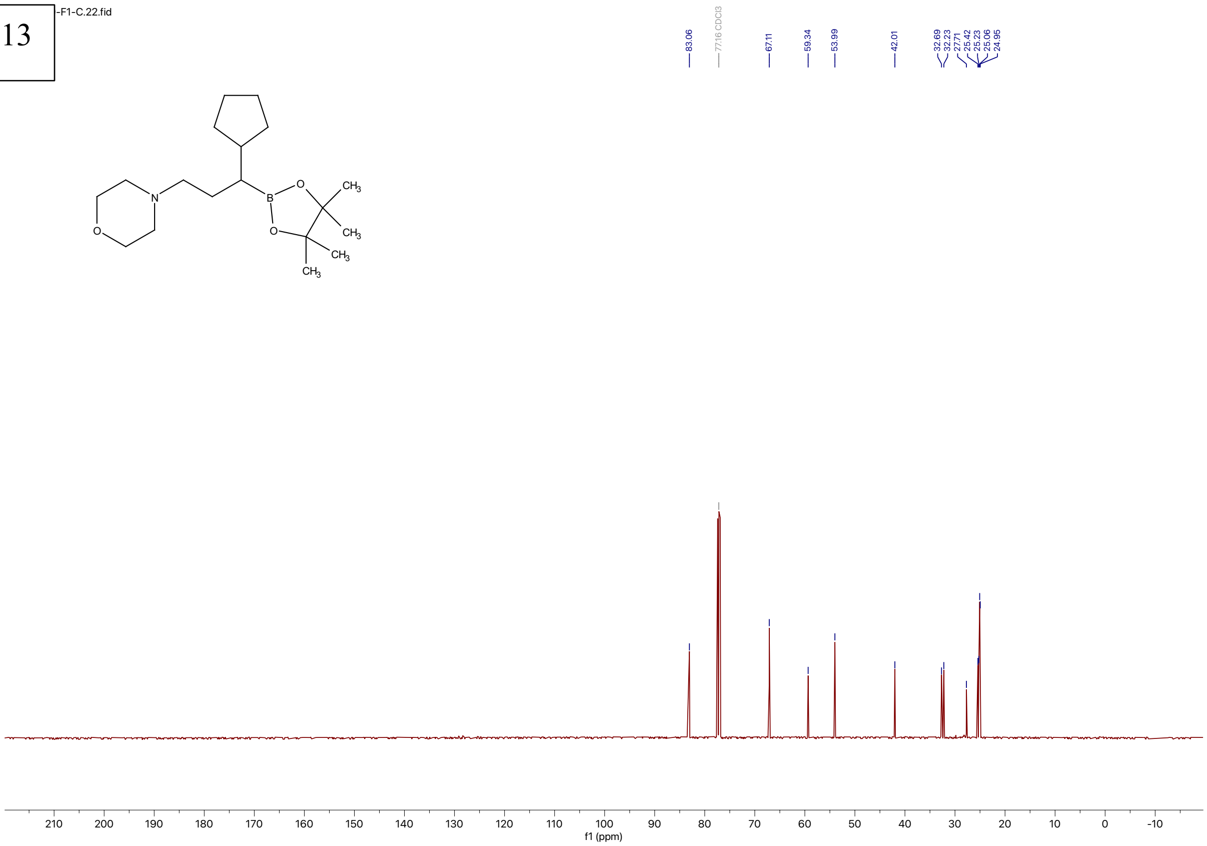
-F1-C.22.fid
83.06
77.16 CDCl3
67.11
59.34
53.99
42.01
32.69
32.23
27.71
25.42
25.23
25.06
24.95
f1 (ppm)

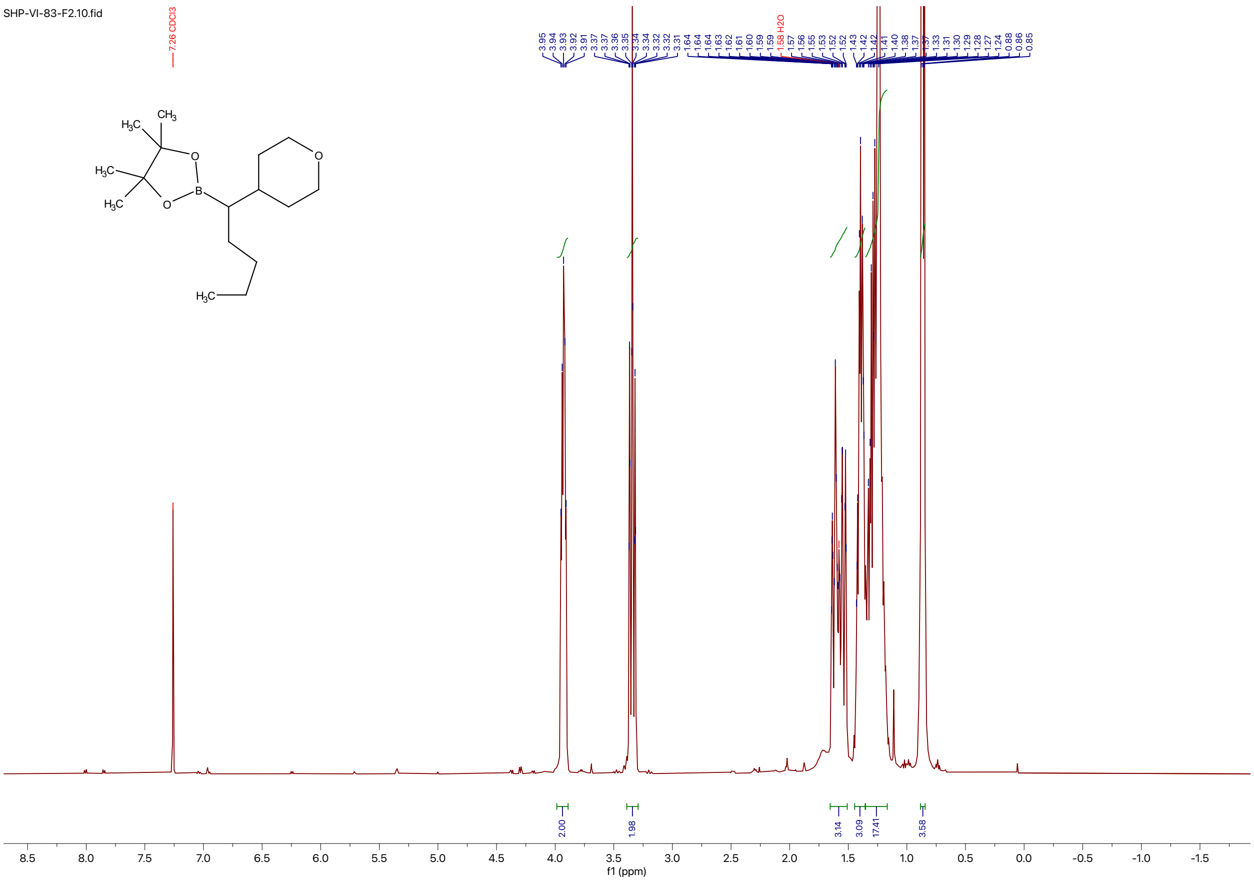
SHP-VI-83-F2.10.fid
7.26 CDCl3
3.95 3.94 3.93 3.92 3.91 3.37 3.37 3.36 3.35 3.34 3.34 3.32 3.32 3.31 1.64 1.64 1.63 1.62 1.61 1.60 1.59 1.59 1.58 H2O 1.57 1.56 1.55 1.53 1.52 1.52 1.43 1.42 1.42 1.41 1.40 1.38 1.37 1.37 1.33 1.31 1.30 1.29 1.28 1.27 1.24 0.88 0.86 0.85
2.00 1.98 3.14 3.09 17.41 3.58
f1 (ppm)

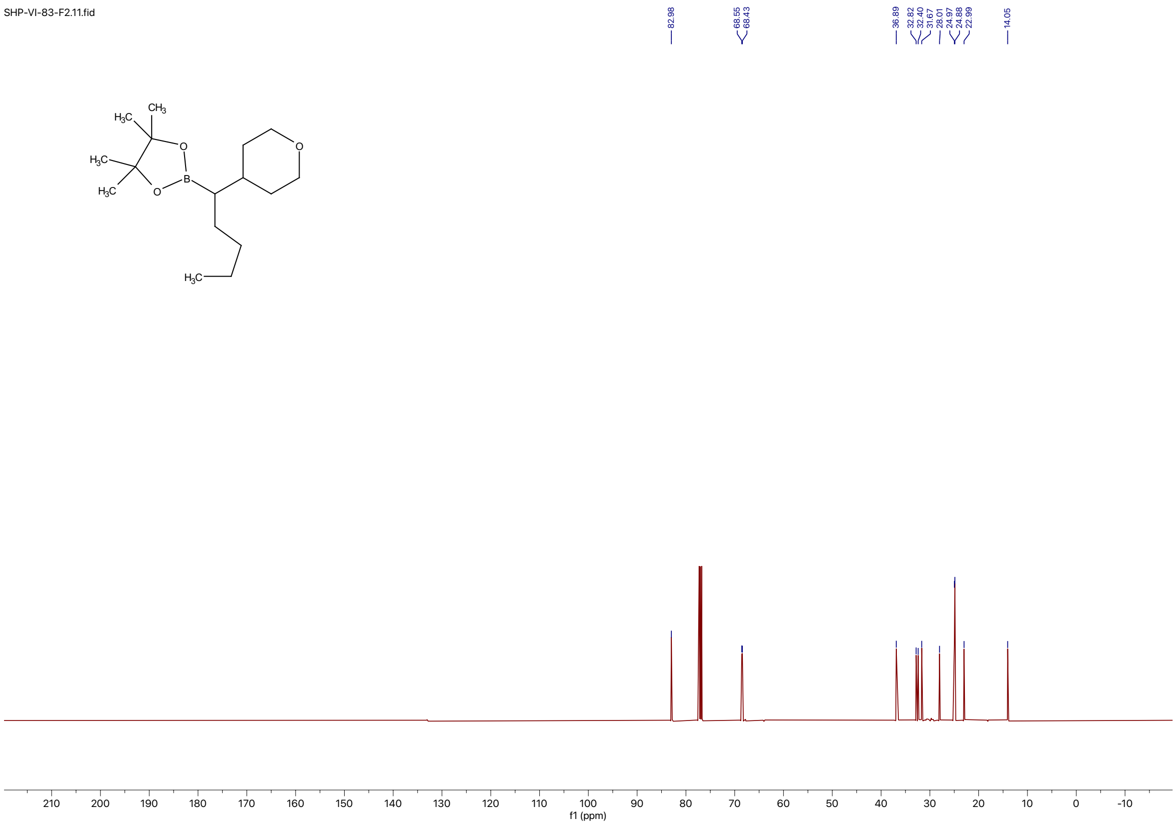
SHP-VI-83-F2.11.fid
82.98
68.55 68.43
36.89 32.82 32.40 31.67 28.01 24.97 24.88 22.99
14.05
f1 (ppm)

14

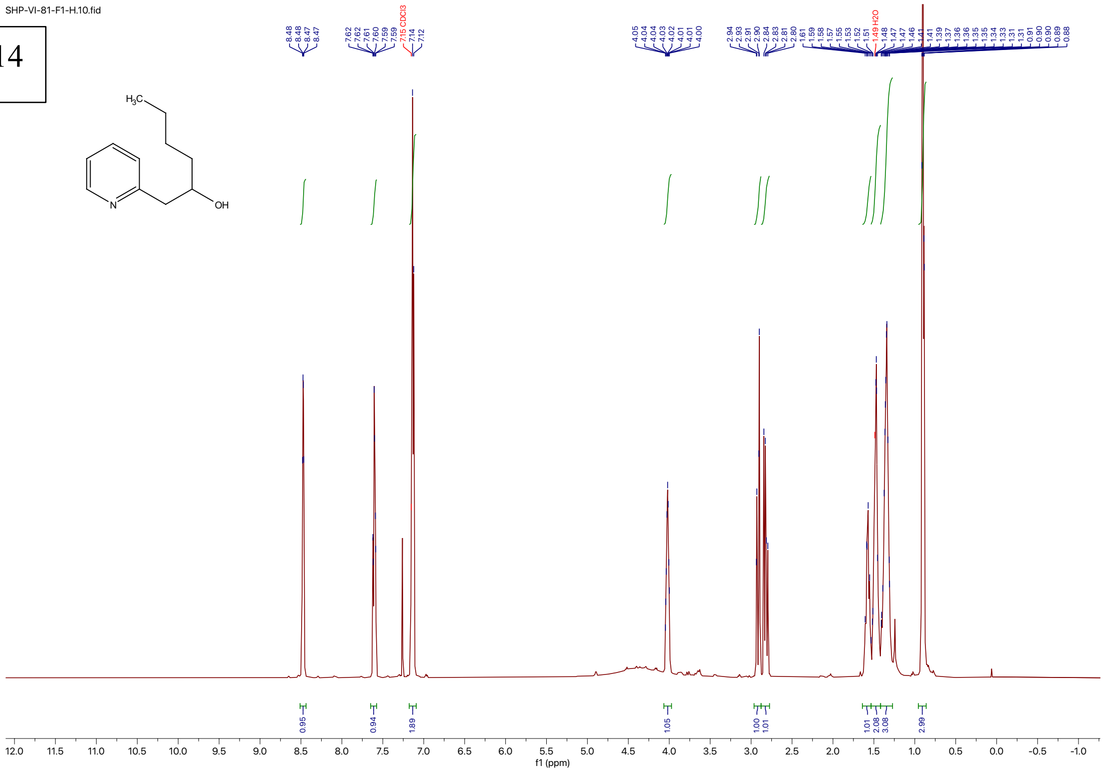
SHP-VI-81-F1-H.10.fid
8.48
8.48
8.47
8.47
7.62
7.62
7.61
7.60
7.59
7.59
7.15 CDCl3
7.14
7.12
4.05
4.04
4.04
4.03
4.02
4.01
4.01
4.00
2.94
2.93
2.91
2.90
2.84
2.83
2.81
2.80
1.61
1.59
1.58
1.57
1.55
1.53
1.52
1.51
1.49 H2O
1.48
1.47
1.46
1.41
1.41
1.39
1.37
1.36
1.36
1.35
1.35
1.34
1.33
1.31
0.91
0.90
0.90
0.89
0.88
H3C
N
OH
0.95
0.94
1.89
1.05
1.00
1.01
1.01
2.08
3.08
2.99
f1 (ppm)

14

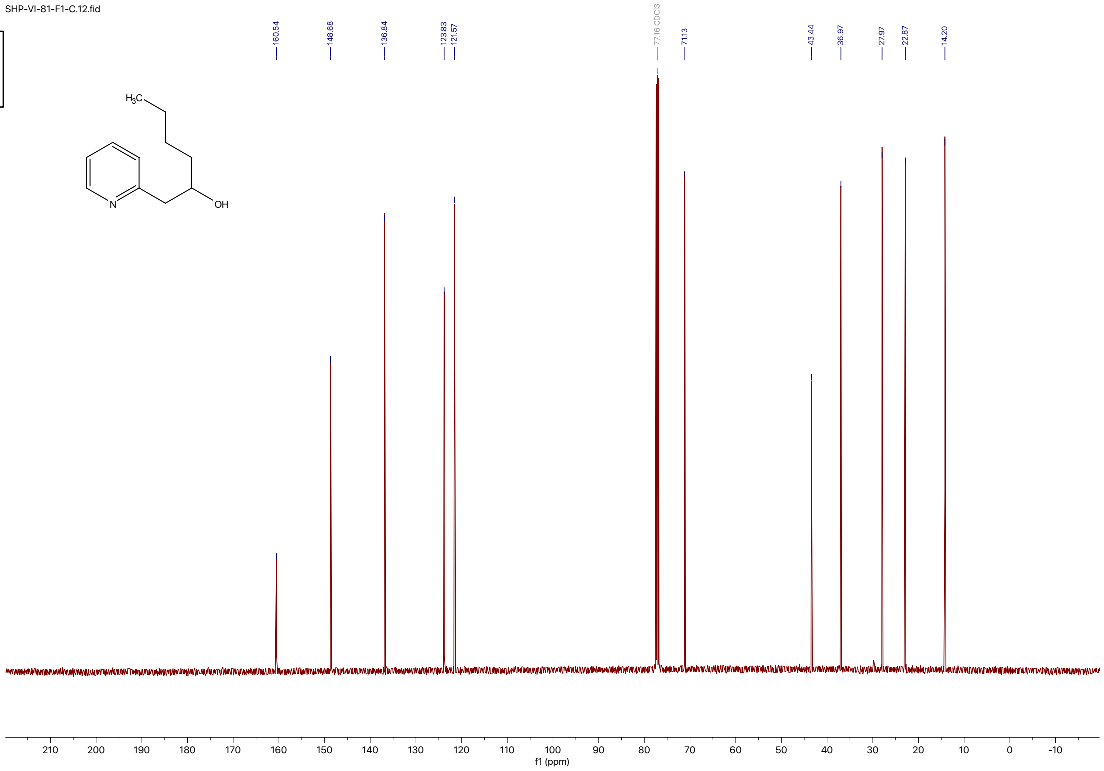
SHP-VI-81-F1-C.12.fid
160.54
148.68
136.84
123.83
121.57
77.16 CDCl3
71.13
43.44
36.97
27.97
22.87
14.20
H3C
N
OH
f1 (ppm)

15

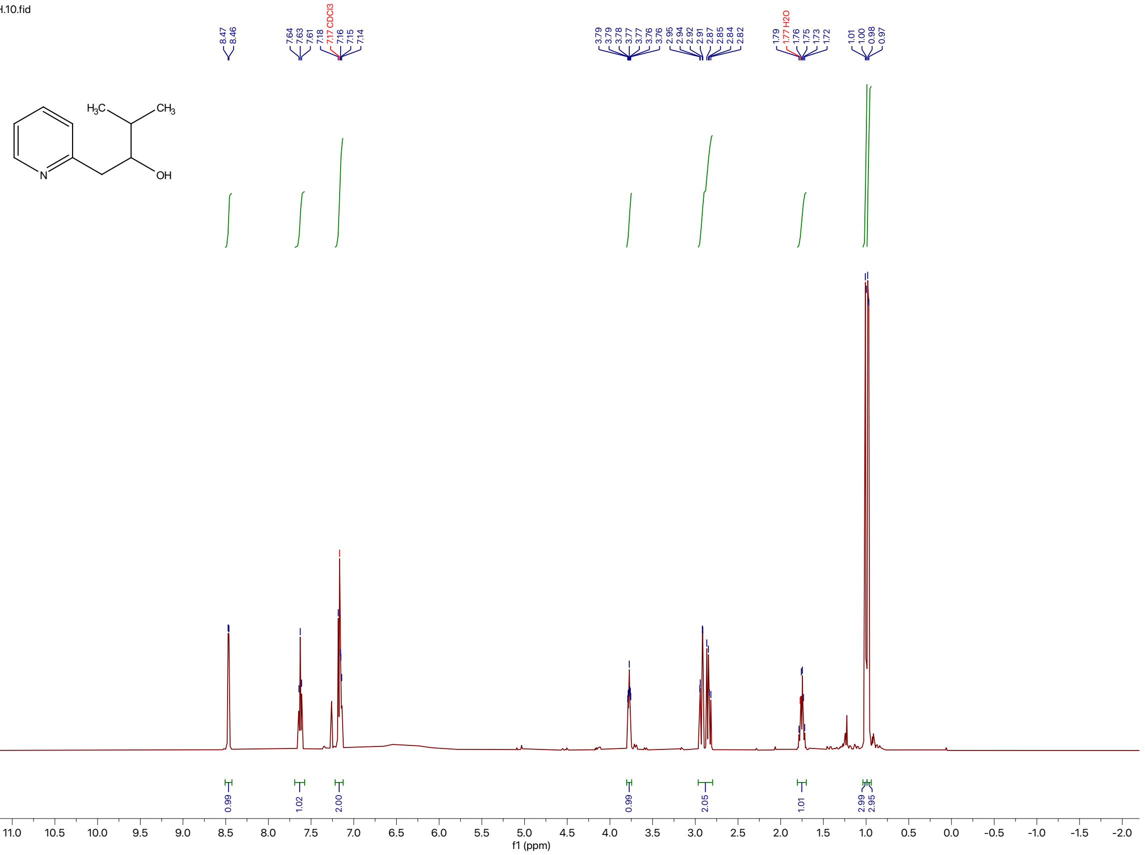
SHP-VI-75-F1-H.10.fid
H3C
CH3
N
OH
8.47
8.46
7.64
7.63
7.61
7.18
7.17 CDCl3
7.16
7.15
7.14
3.79
3.79
3.78
3.77
3.77
3.76
3.76
2.95
2.94
2.92
2.91
2.87
2.85
2.84
2.82
1.79
1.77 H2O
1.76
1.75
1.73
1.72
1.01
1.00
0.98
0.97
0.99
1.02
2.00
0.99
2.05
1.01
2.99
2.95
f1 (ppm)

15

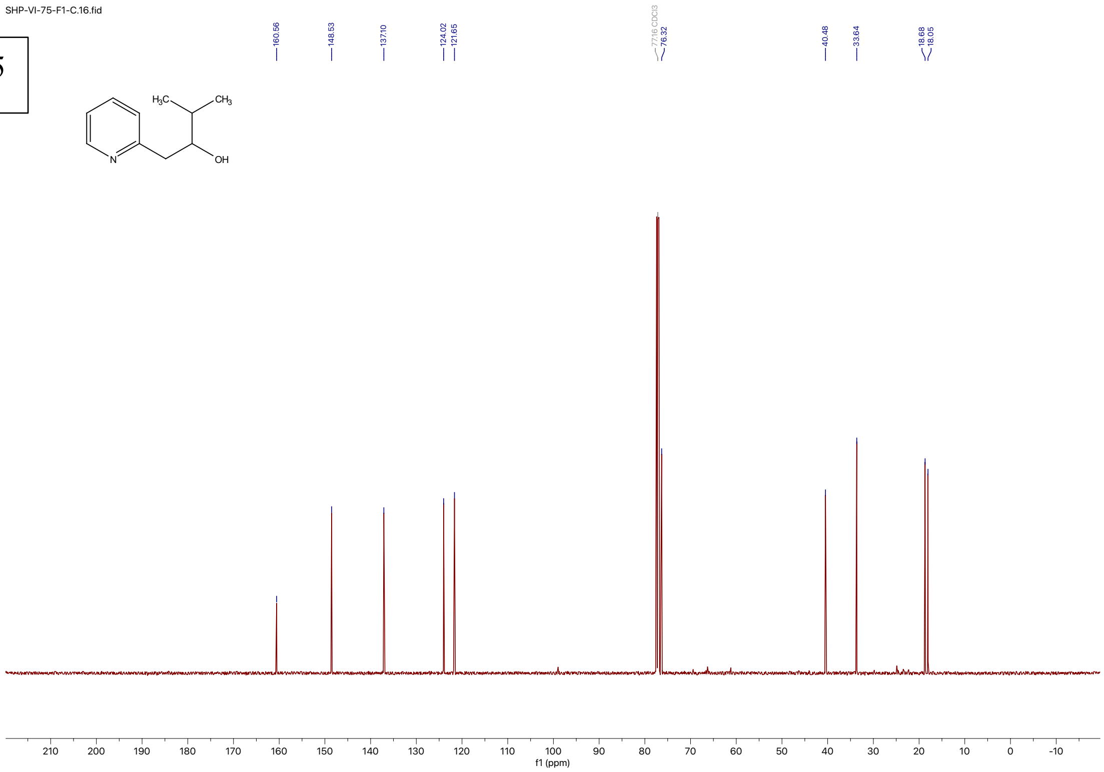
SHP-VI-75-F1-C.16.fid
H3C
CH3
N
OH
160.56
148.53
137.10
124.02
121.65
77.16 CDCl3
76.32
40.48
33.64
18.68
18.05
f1 (ppm)

16

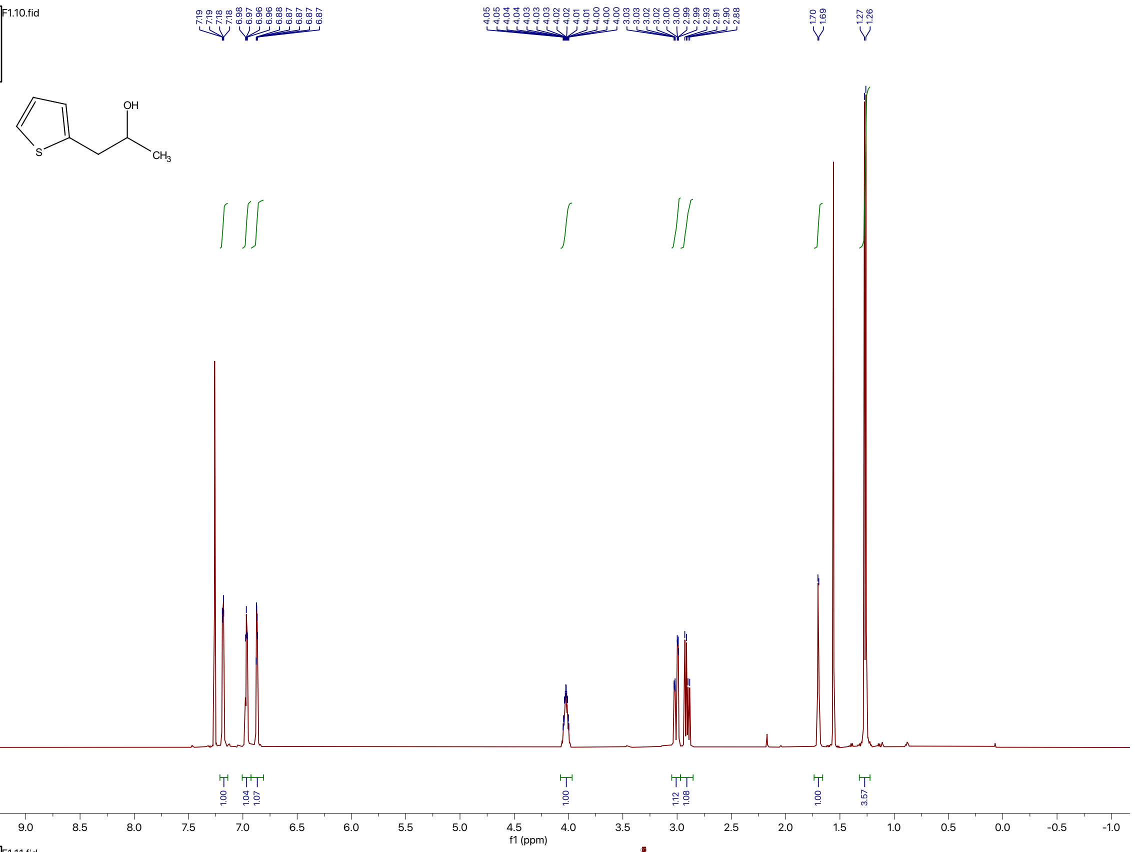
F1.10.fid
OH
S
CH3
7.19
7.19
7.18
7.18
6.98
6.97
6.96
6.96
6.88
6.87
6.87
6.87
4.05
4.05
4.04
4.03
4.03
4.02
4.02
4.01
4.01
4.00
4.00
4.00
1.70
1.69
1.27
1.26
1.00
1.04
1.07
1.00
1.12
1.08
1.00
3.57
f1 (ppm)

16

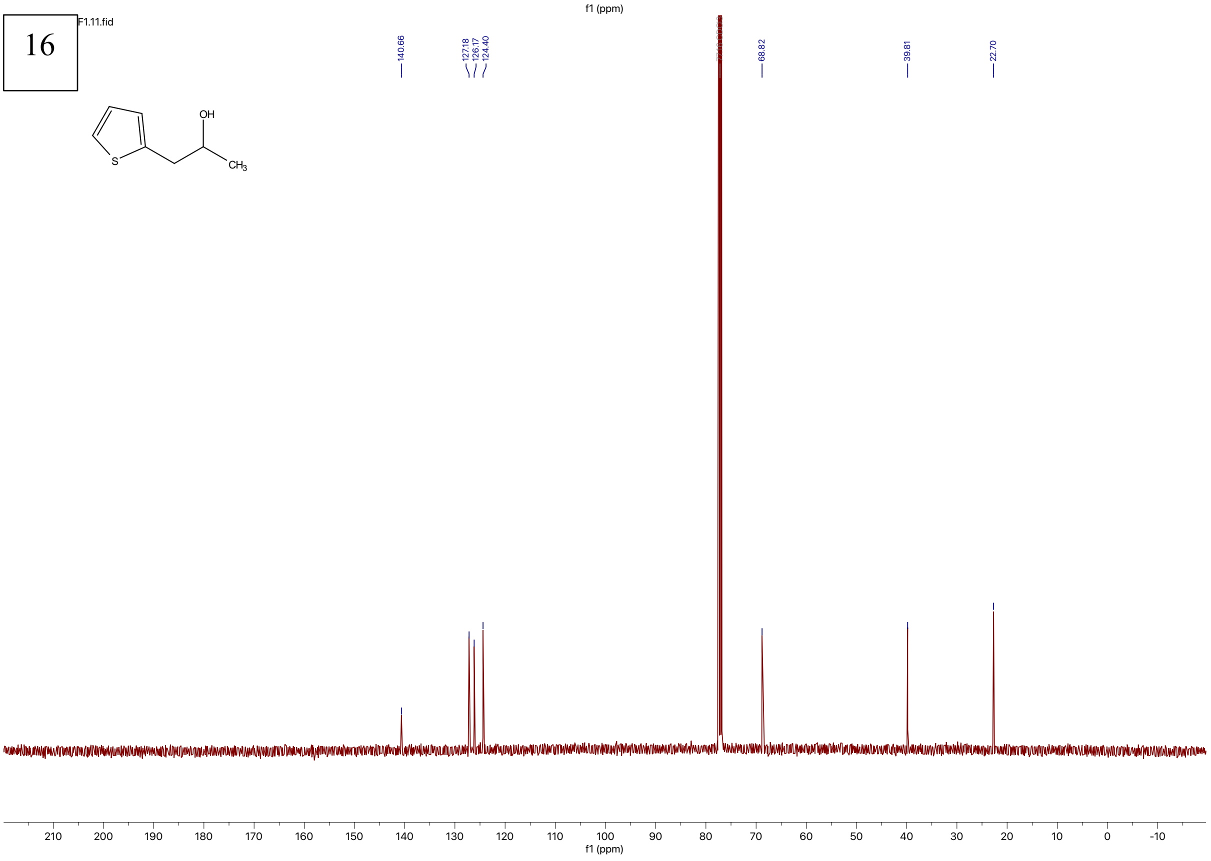
F1.11.fid
OH
S
CH3
140.66
127.18
126.17
124.40
68.82
39.81
22.70
f1 (ppm)

17

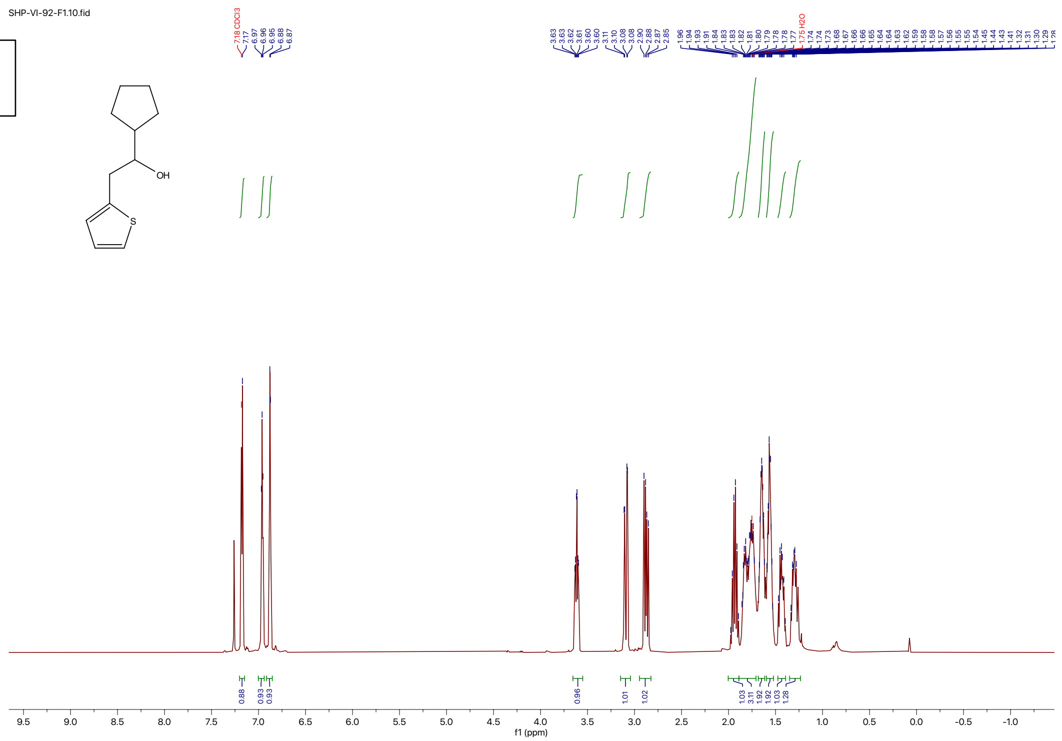
SHP-VI-92-F1.10.fid
OH
S
7.18 CDCl3
7.17
6.97
6.96
6.95
6.88
6.87
3.63
3.63
3.62
3.61
3.60
3.60
3.11
3.10
3.08
3.08
2.90
2.88
2.87
2.85
1.75 H2O
0.88
0.93
0.93
0.96
1.01
1.02
1.03
3.11
1.92
1.92
1.03
1.28
f1 (ppm)

17

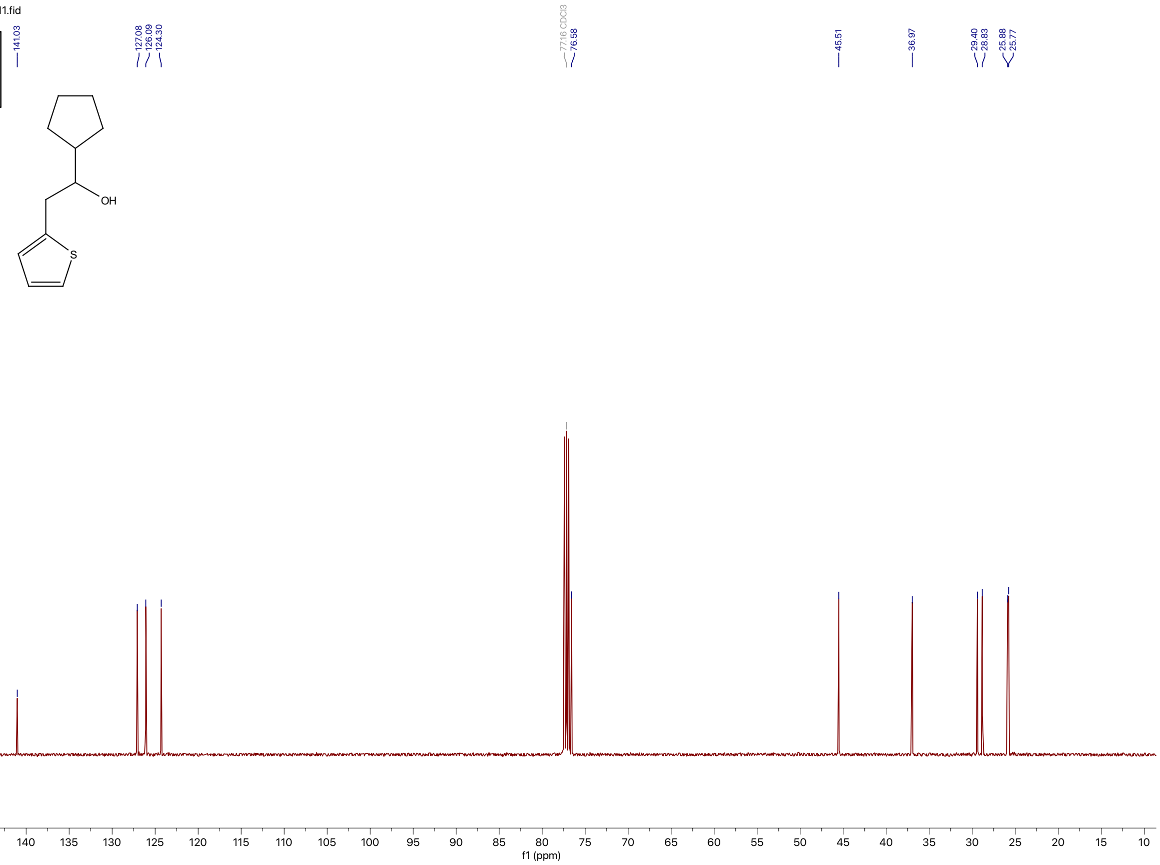
SHP-VI-92-F1.11.fid
OH
S
141.03
127.08
126.09
124.30
77.16 CDCl3
76.58
45.51
36.97
29.40
28.83
25.88
25.77
f1 (ppm)

SHP-VI-TRIP-BPIN.10.fid

18

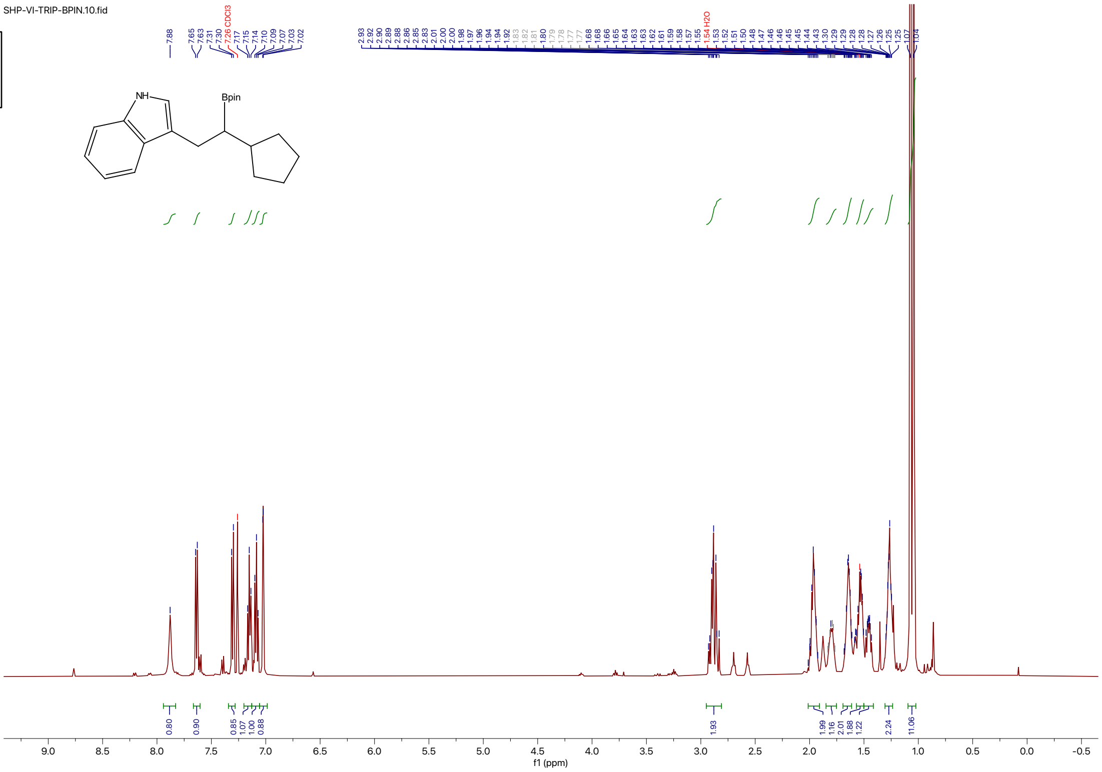


SHP-VI-TRIP-BPIN-C.10.fid

18

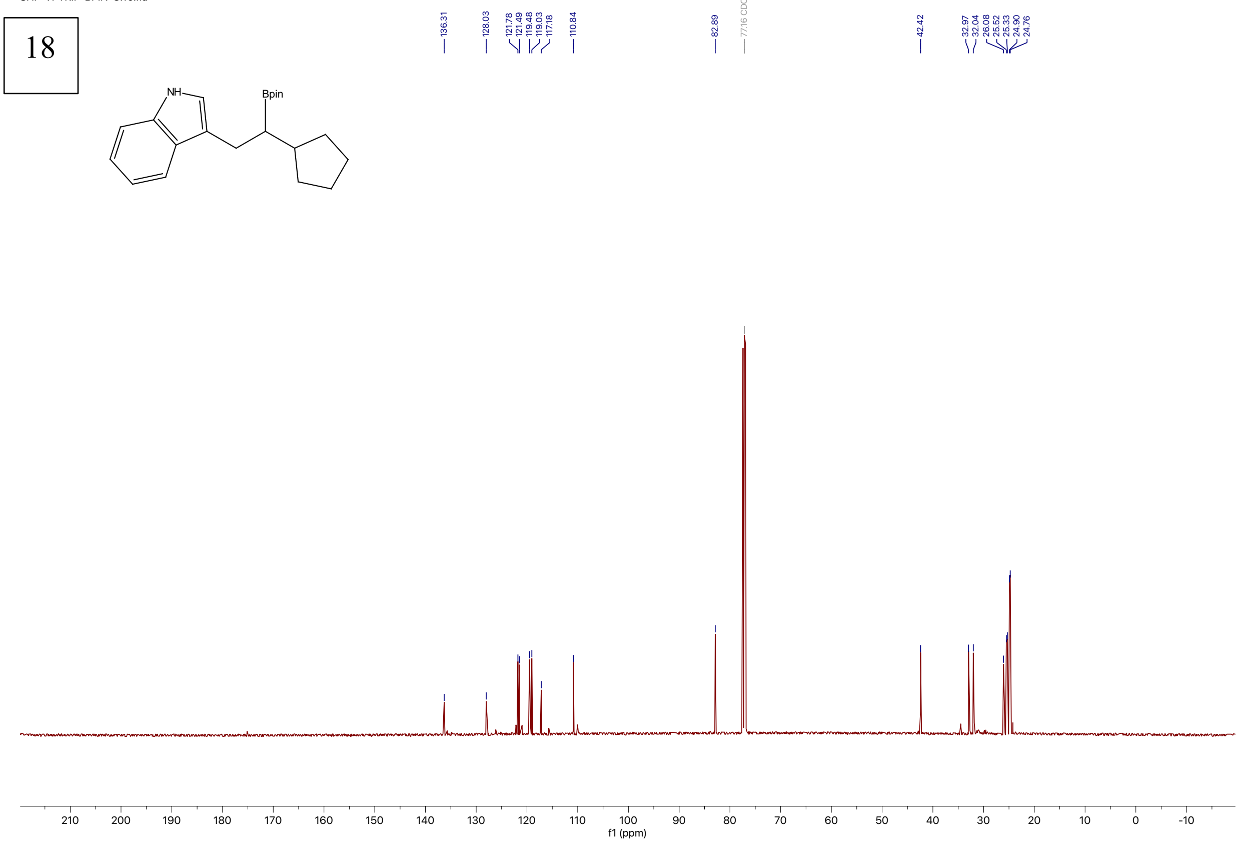

19

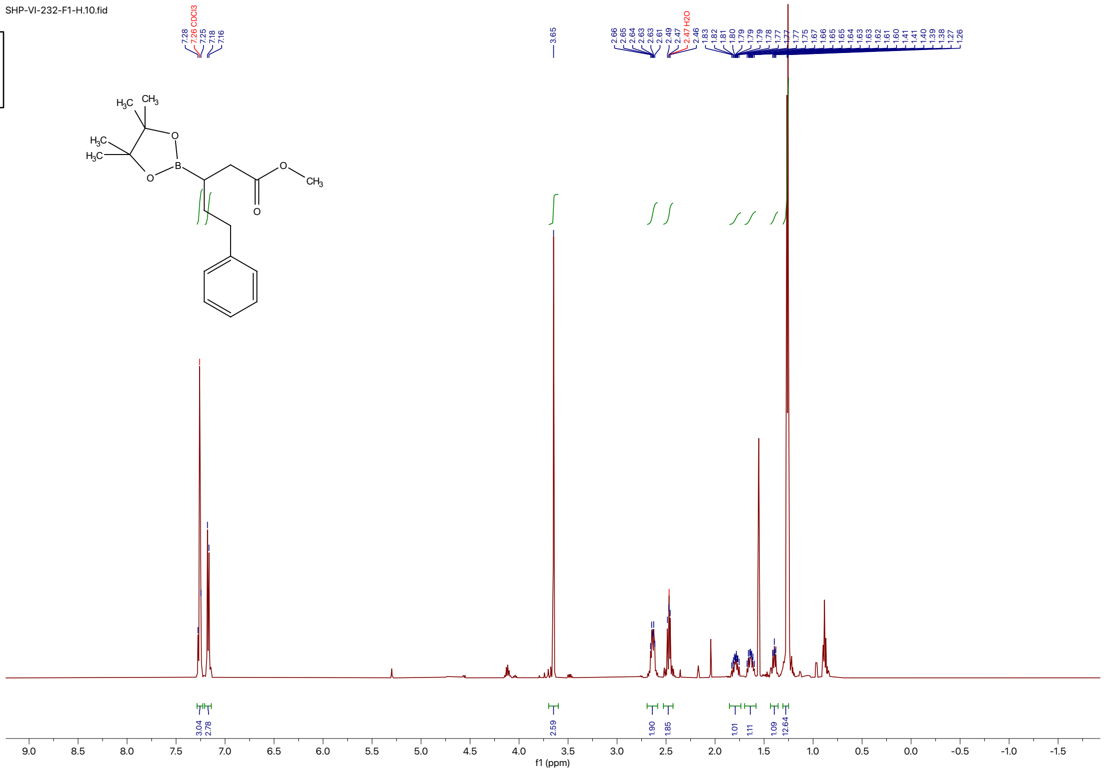
SHP-VI-232-F1-H.10.fid
3.65
3.04
2.78
2.59
1.90
1.85
1.01
1.11
1.09
12.64
f1 (ppm)

19

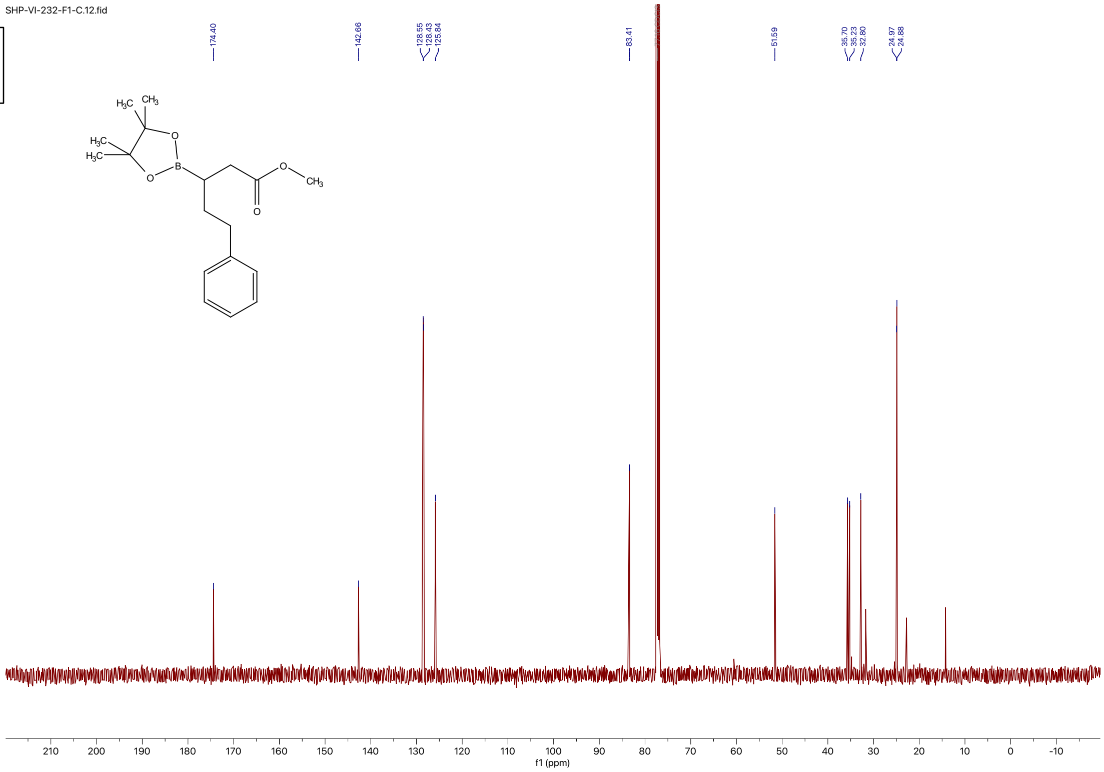
SHP-VI-232-F1-C.12.fid
174.40
142.66
128.55
128.43
125.84
83.41
51.59
35.70
35.23
32.80
24.97
24.88
f1 (ppm)

20

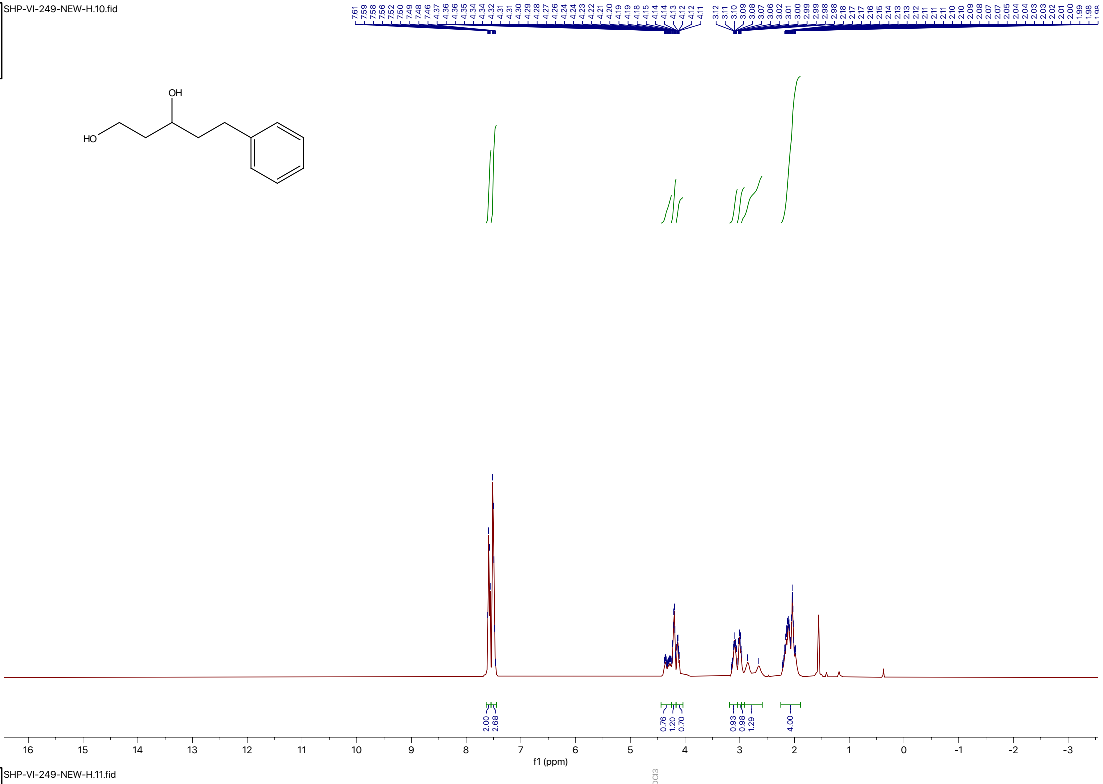

SHP-VI-249-NEW-H.10.fid
OH
HO
2.00
2.68
0.76
1.20
0.70
0.93
0.98
1.29
4.00
f1 (ppm)


20

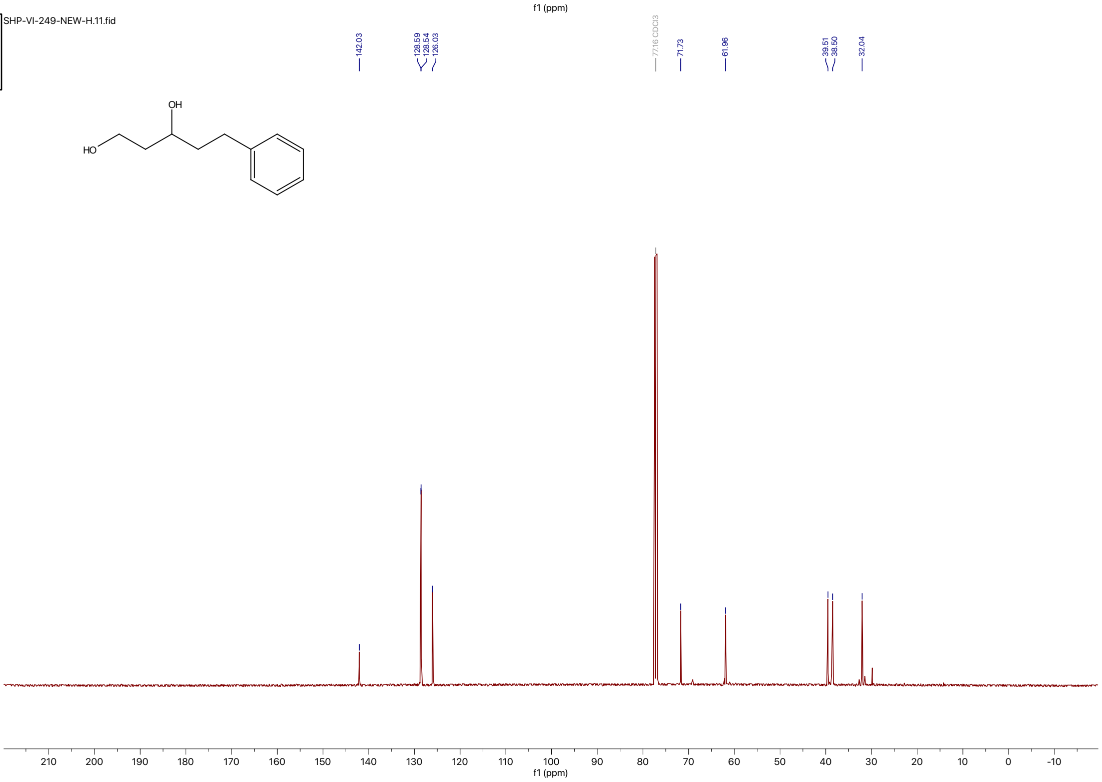

SHP-VI-249-NEW-H.11.fid
OH
HO
142.03
128.59
128.54
126.03
77.16 CDCl3
71.73
61.96
39.51
38.50
32.04
f1 (ppm)

21

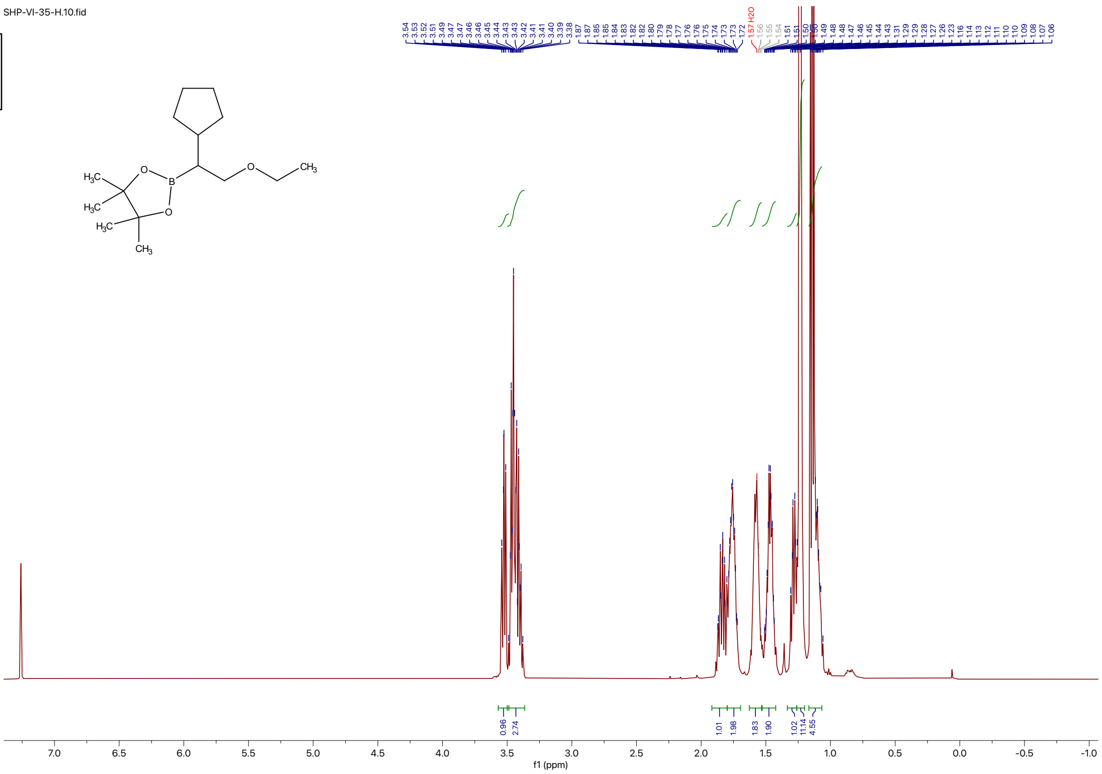

SHP-VI-35-H.10.fid
1.57 H2O
0.96
2.74
1.01
1.98
1.83
1.90
1.02
1.14
4.55
f1 (ppm)


21

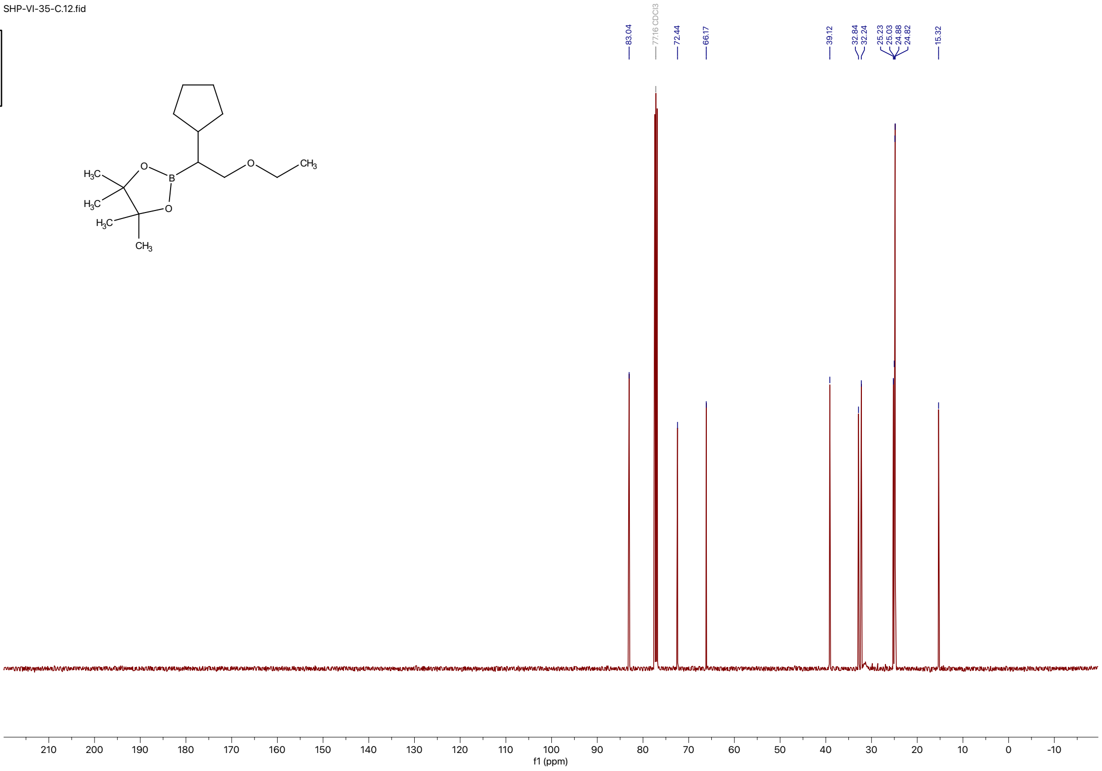

SHP-VI-35-C.12.fid
83.04
77.16 CDCl3
72.44
66.17
39.12
32.84
32.24
25.23
25.03
24.88
24.82
15.32
f1 (ppm)

22

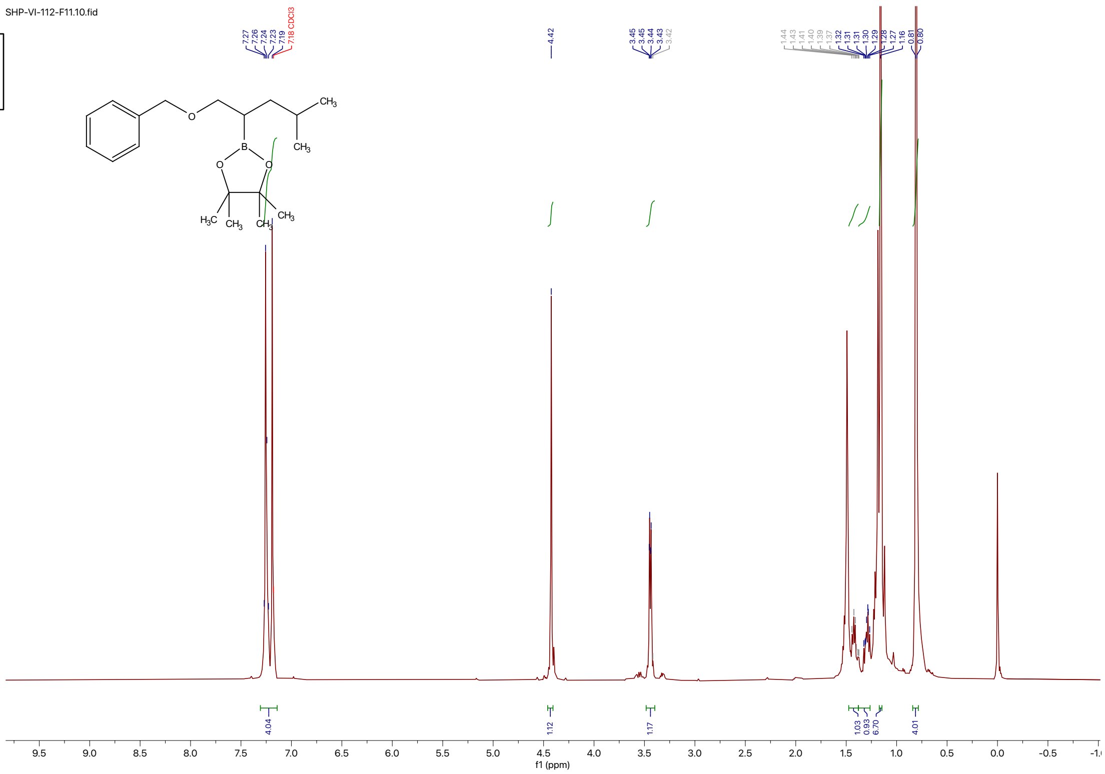
SHP-VI-112-F11.10.fid
7.27
7.26
7.24
7.23
7.19
7.18 CDCl3
4.42
3.45
3.45
3.44
3.43
3.42
4.04
1.12
1.17
1.03
0.93
6.70
4.01
f1 (ppm)

22

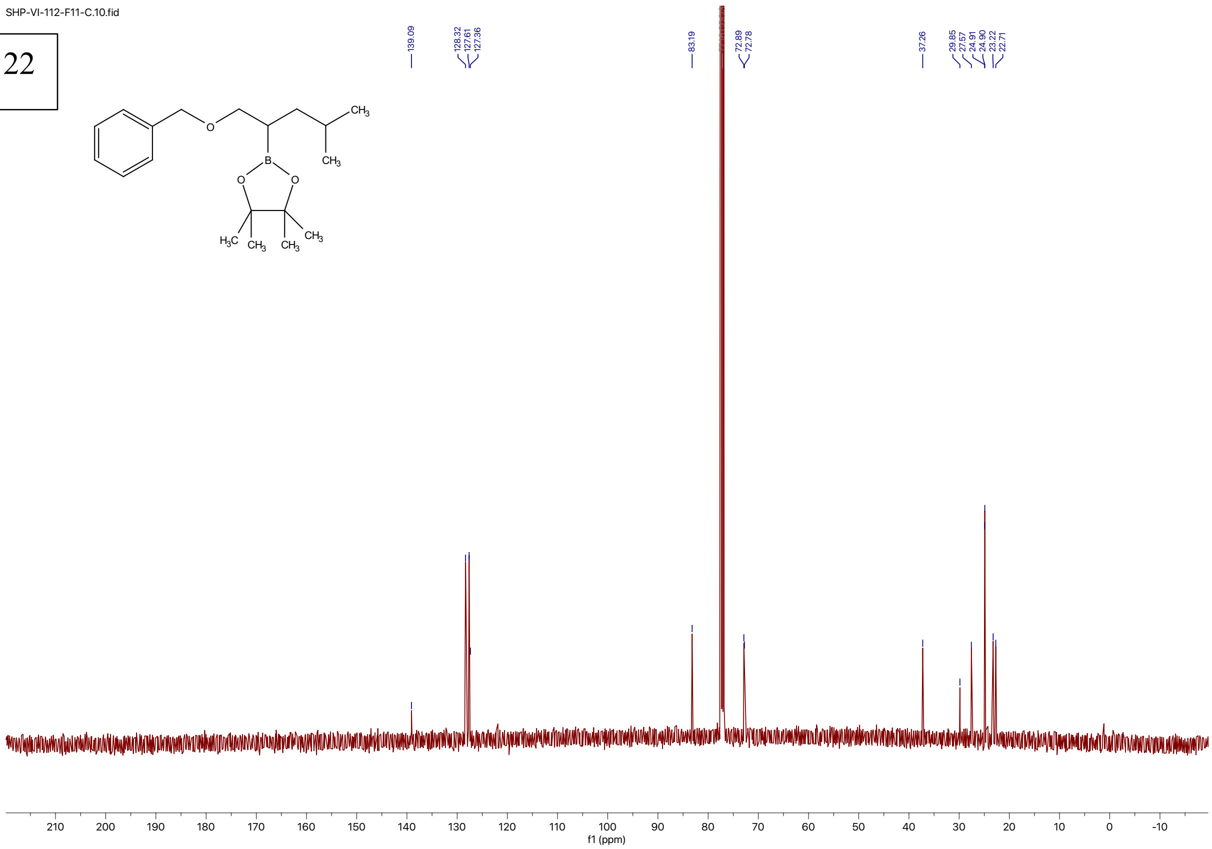
SHP-VI-112-F11-C.10.fid
139.09
128.32
127.61
127.36
83.19
72.89
72.78
37.26
29.85
27.57
24.91
24.90
23.22
22.71
f1 (ppm)

SHP-VI-33-F2.10.fid

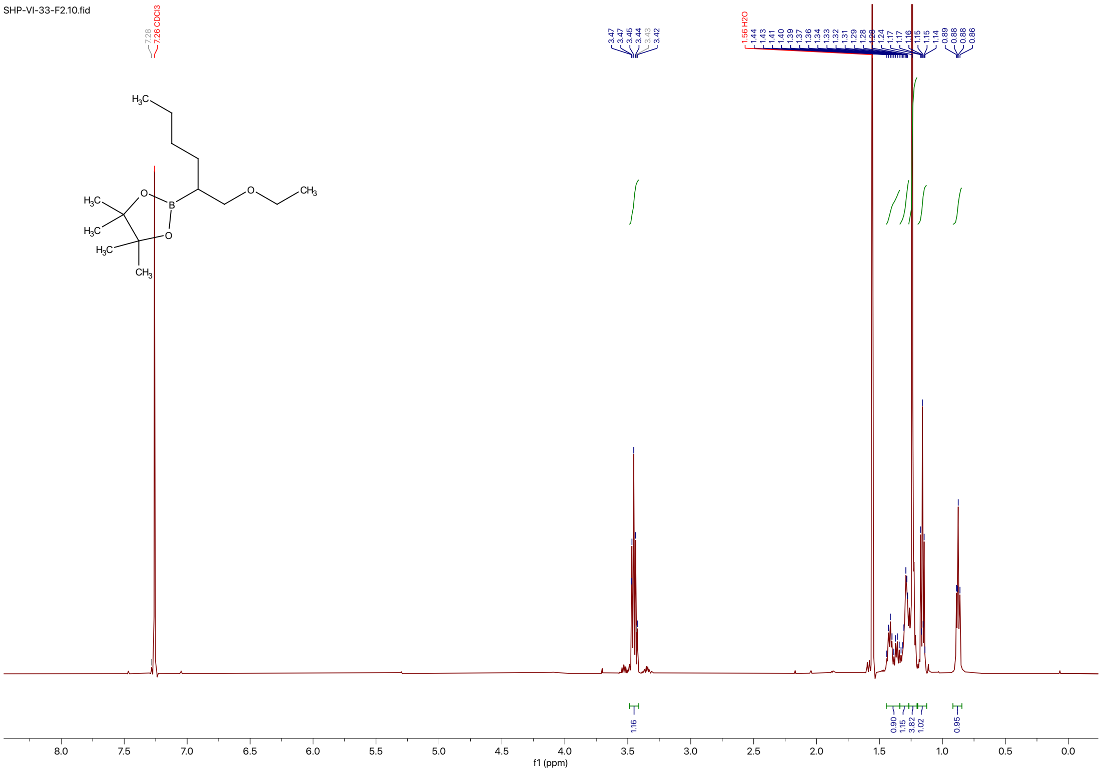

SHP-VI-33-F2-C.10.fid

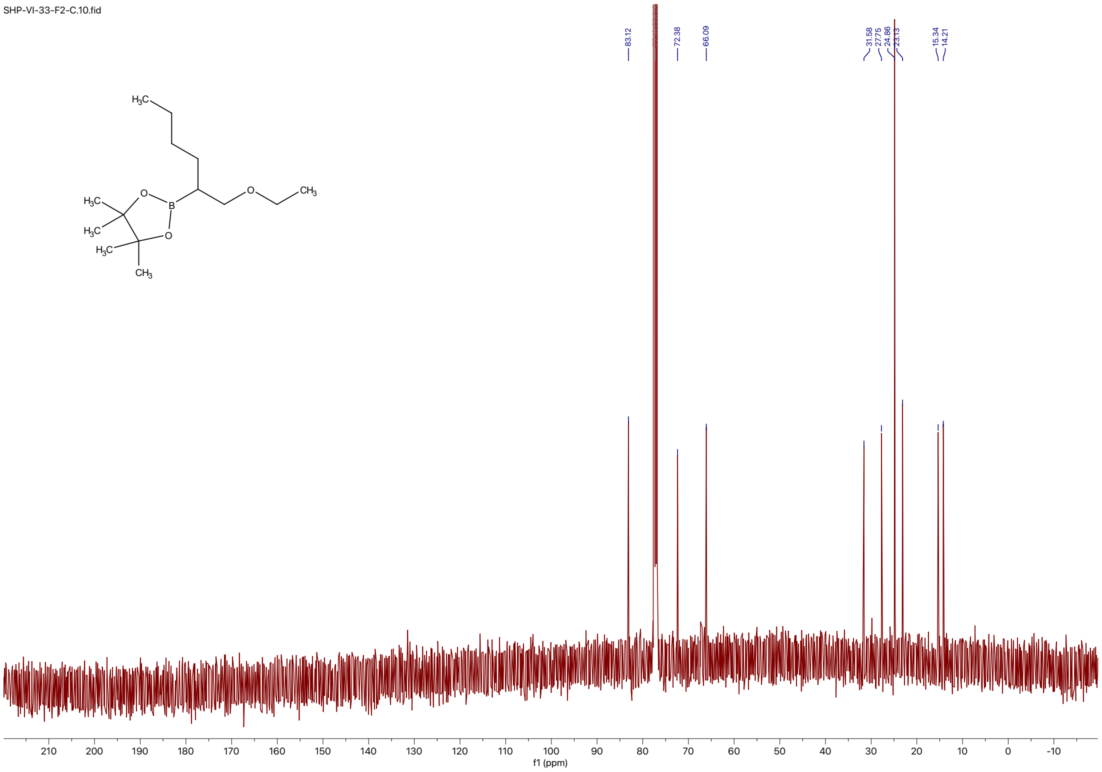

SHP-VI-36-H.10.fid

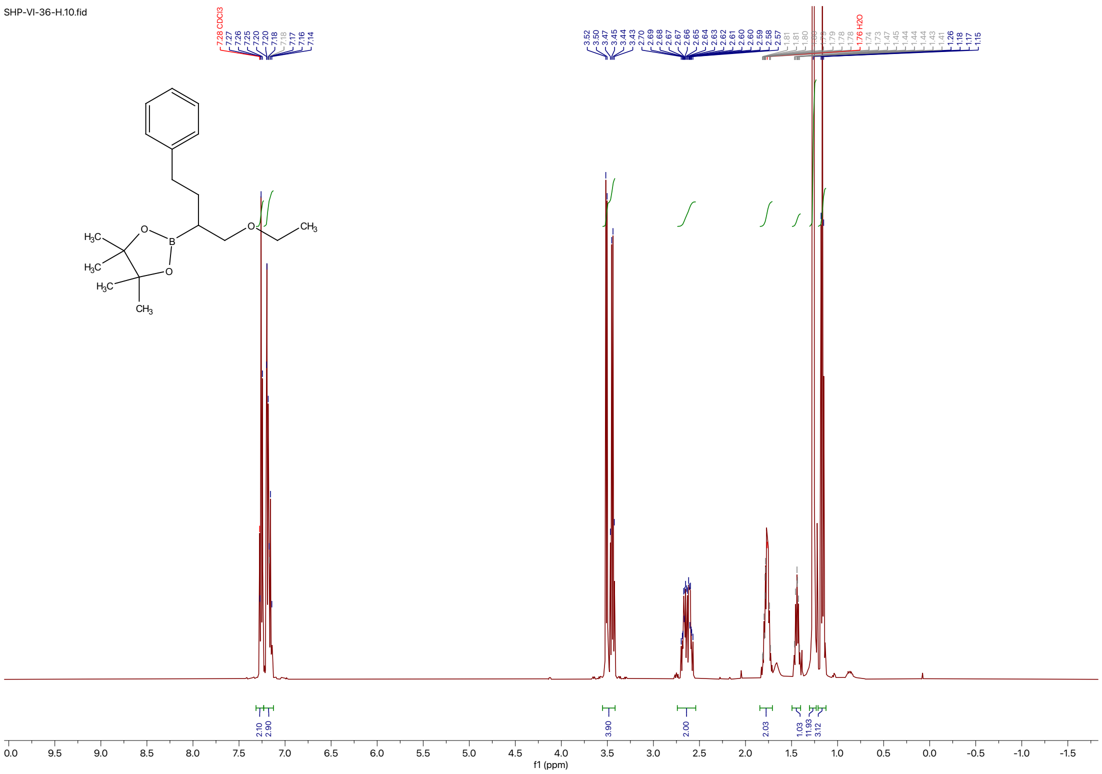


SHP-VI-36-C.12.fid

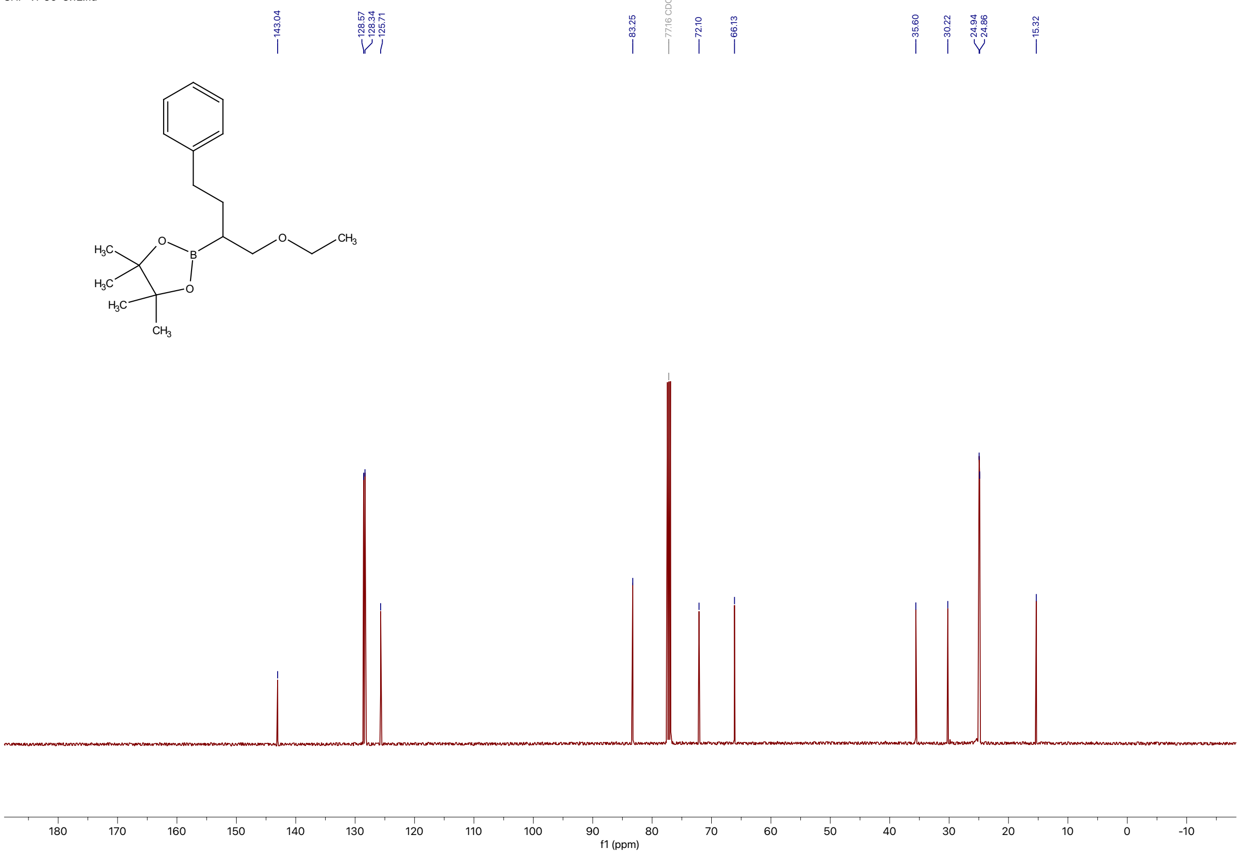

24

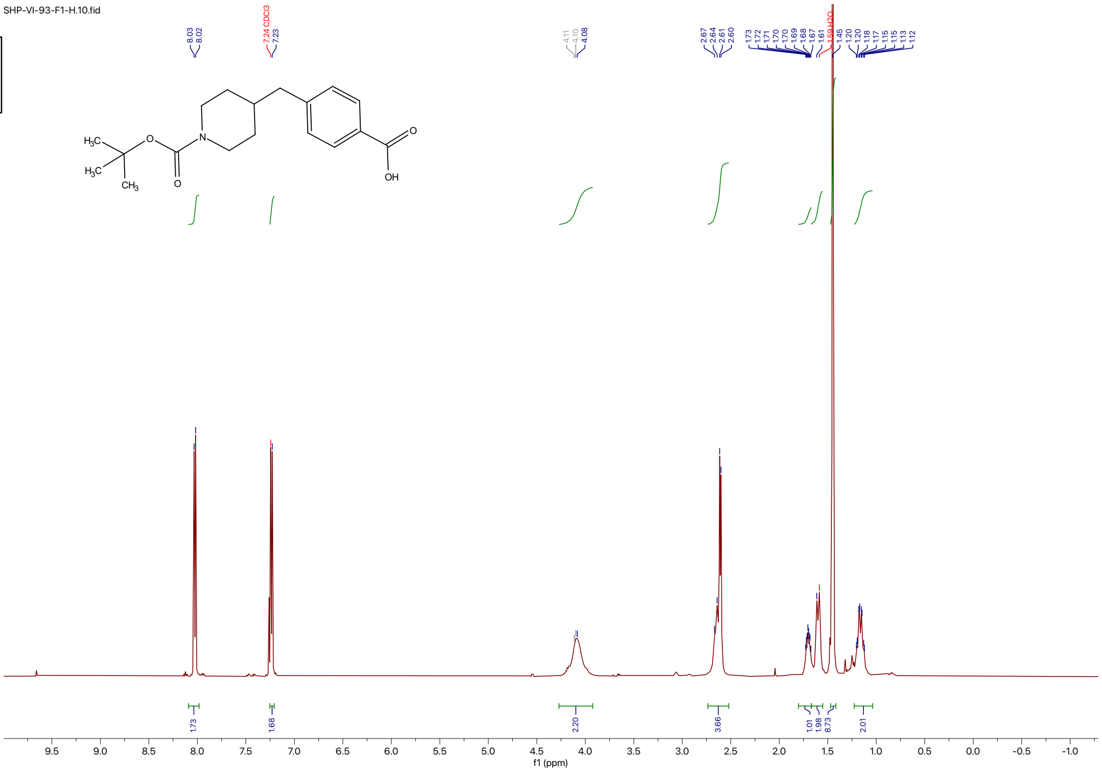

SHP-VI-93-F1-H.10.fid
8.03
8.02
7.24 CDCl3
7.23
4.11
4.10
4.08
2.67
2.64
2.61
2.60
1.73
1.72
1.71
1.70
1.70
1.69
1.68
1.67
1.61
1.45
1.20
1.20
1.18
1.17
1.15
1.15
1.13
1.12
1.73
1.68
2.20
3.66
1.01
1.98
8.73
2.01
f1 (ppm)


24

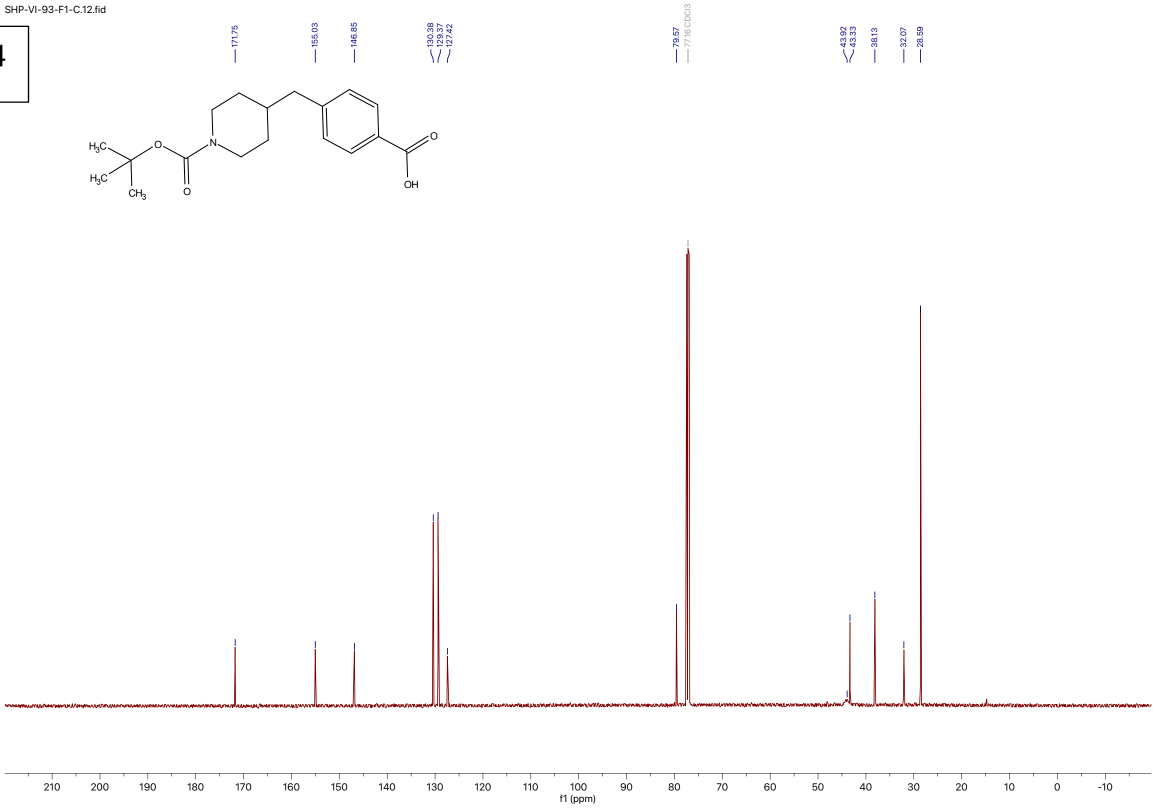

SHP-VI-93-F1-C.12.fid
171.75
155.03
146.85
130.38
129.37
127.42
79.57
77.16 CDCl3
43.92
43.33
38.13
32.07
28.59
f1 (ppm)

25

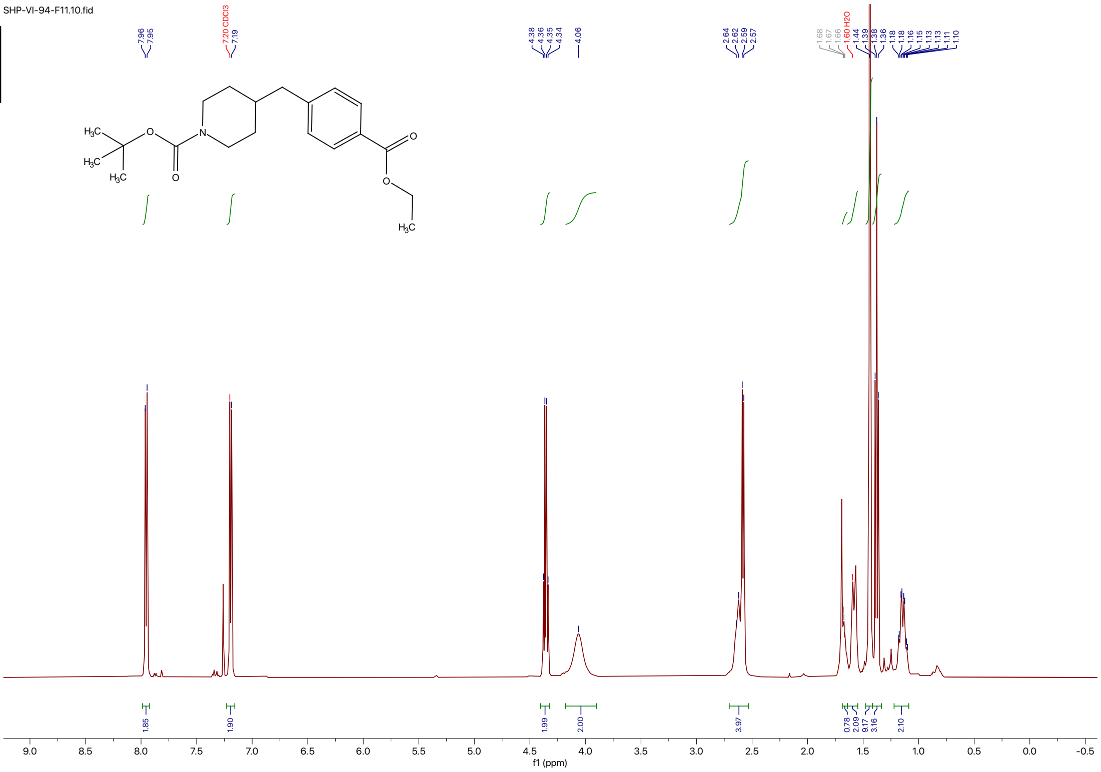
SHP-VI-94-F11.10.fid
7.96
7.95
7.20 CDCl3
7.19
4.38
4.36
4.35
4.34
4.06
2.64
2.62
2.59
2.57
1.60 H2O
1.85
1.90
1.99
2.00
3.97
0.78
2.09
9.17
3.16
2.10
f1 (ppm)

25

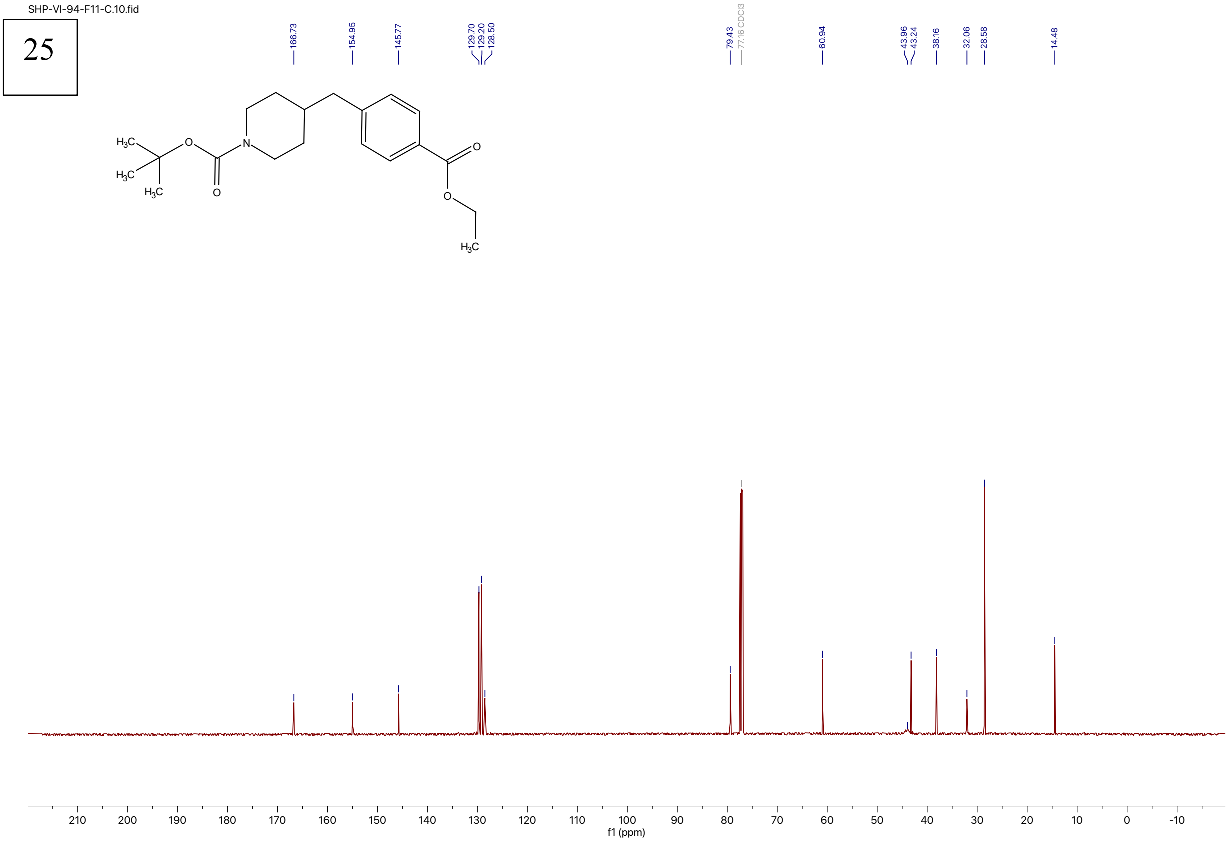
SHP-VI-94-F11-C.10.fid
166.73
154.95
145.77
129.70
129.20
128.50
79.43
77.16 CDCl3
60.94
43.96
43.24
38.16
32.06
28.58
14.48
f1 (ppm)

SHP-VI-108-F1.10.fid

26

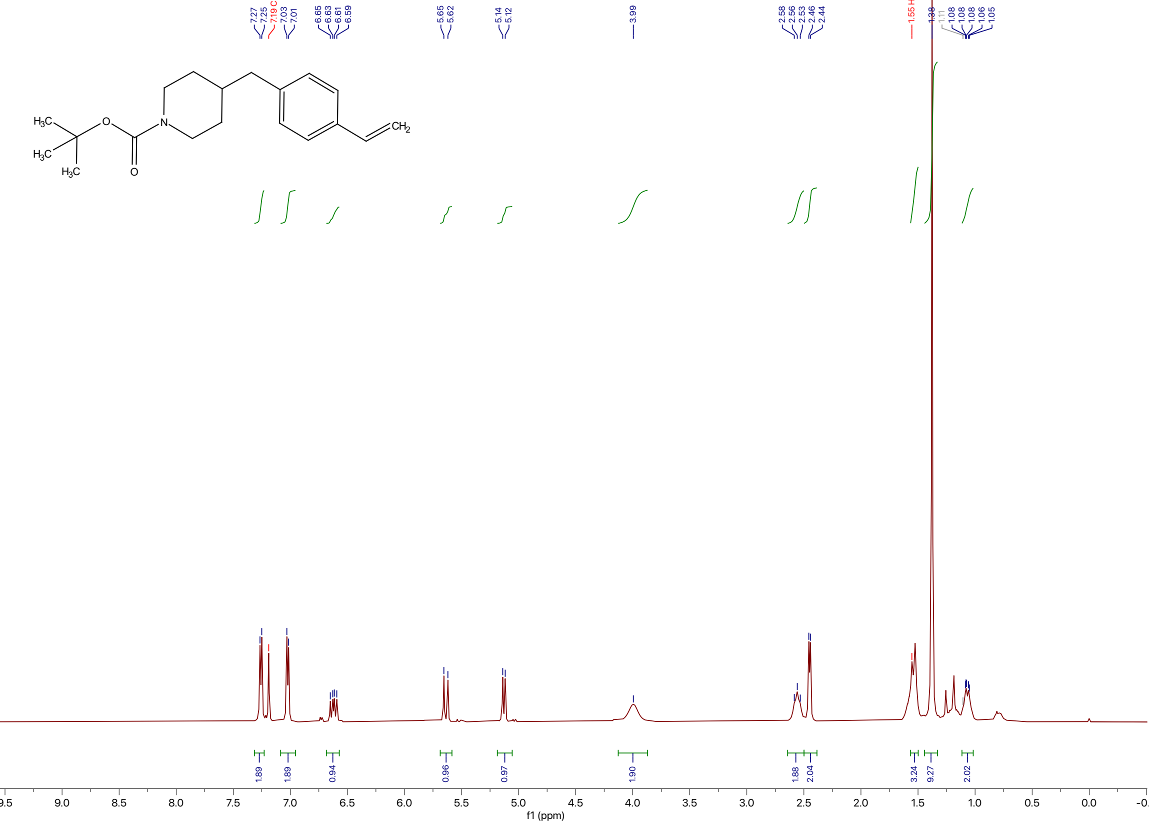


26

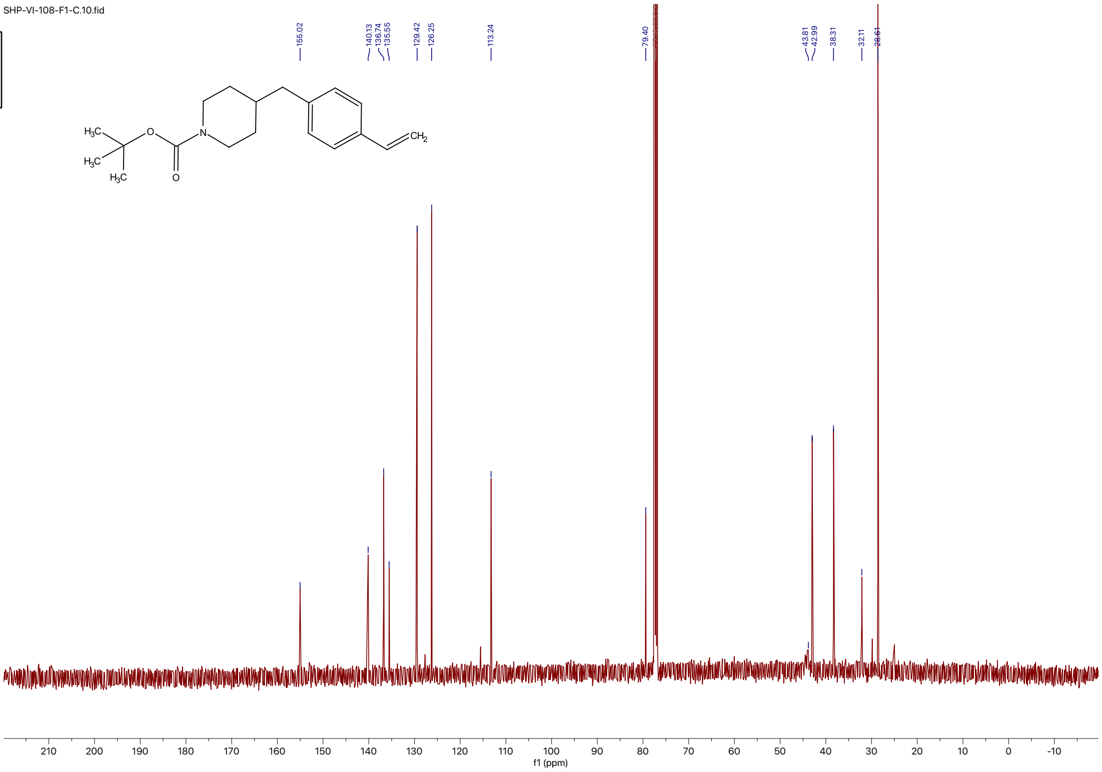

27

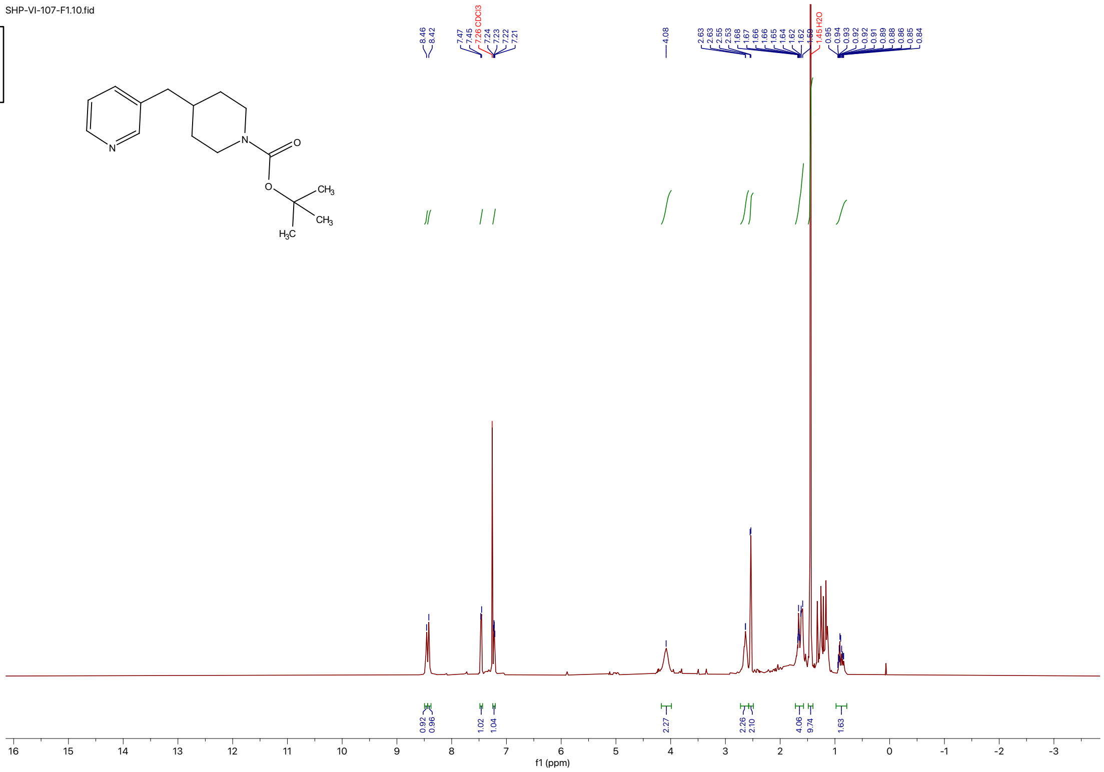
SHP-VI-107-F1.10.fid
8.46
8.42
7.47
7.45
7.26 CDCl3
7.24
7.23
7.22
7.21
4.08
2.63
2.63
2.55
2.53
1.68
1.67
1.66
1.66
1.65
1.64
1.62
1.62
1.59
1.45 H2O
0.95
0.94
0.93
0.92
0.92
0.91
0.89
0.88
0.86
0.85
0.84
N
O
O
CH3
CH3
H3C
0.92
0.96
1.02
1.04
2.27
2.26
2.10
4.06
9.74
1.63
f1 (ppm)

27

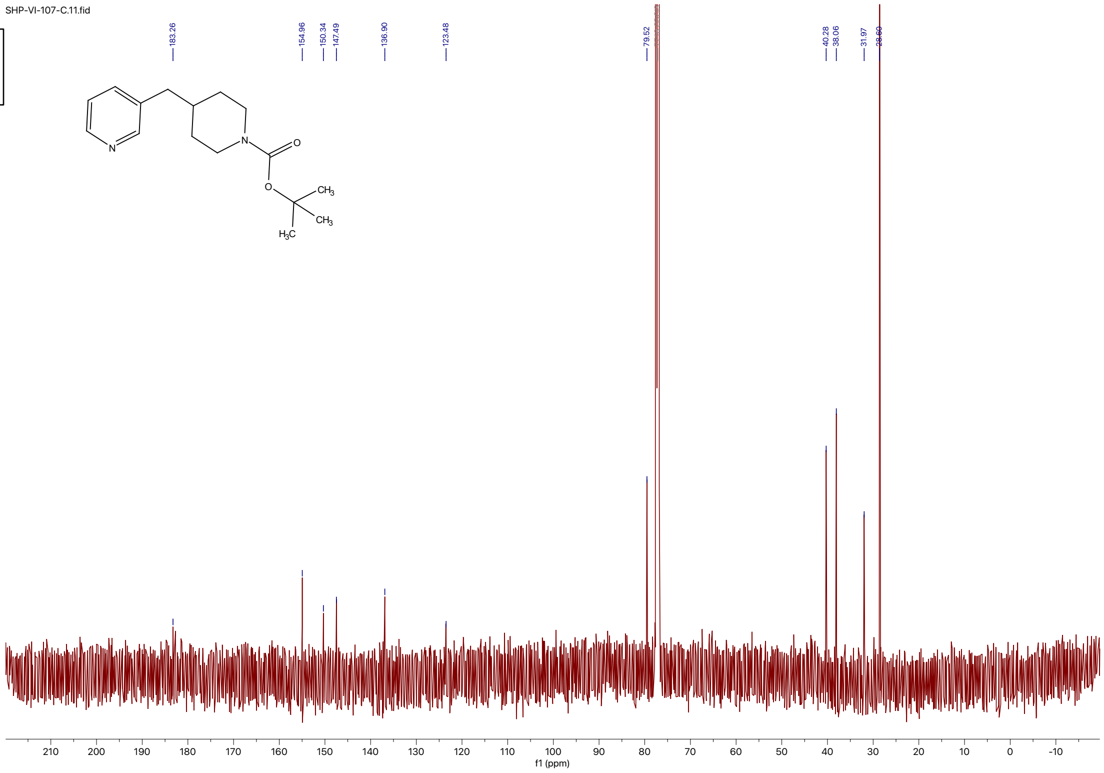
SHP-VI-107-C.11.fid
183.26
154.96
150.34
147.49
136.90
123.48
79.52
40.28
38.06
31.97
28.50
N
O
O
CH3
CH3
H3C
f1 (ppm)

28

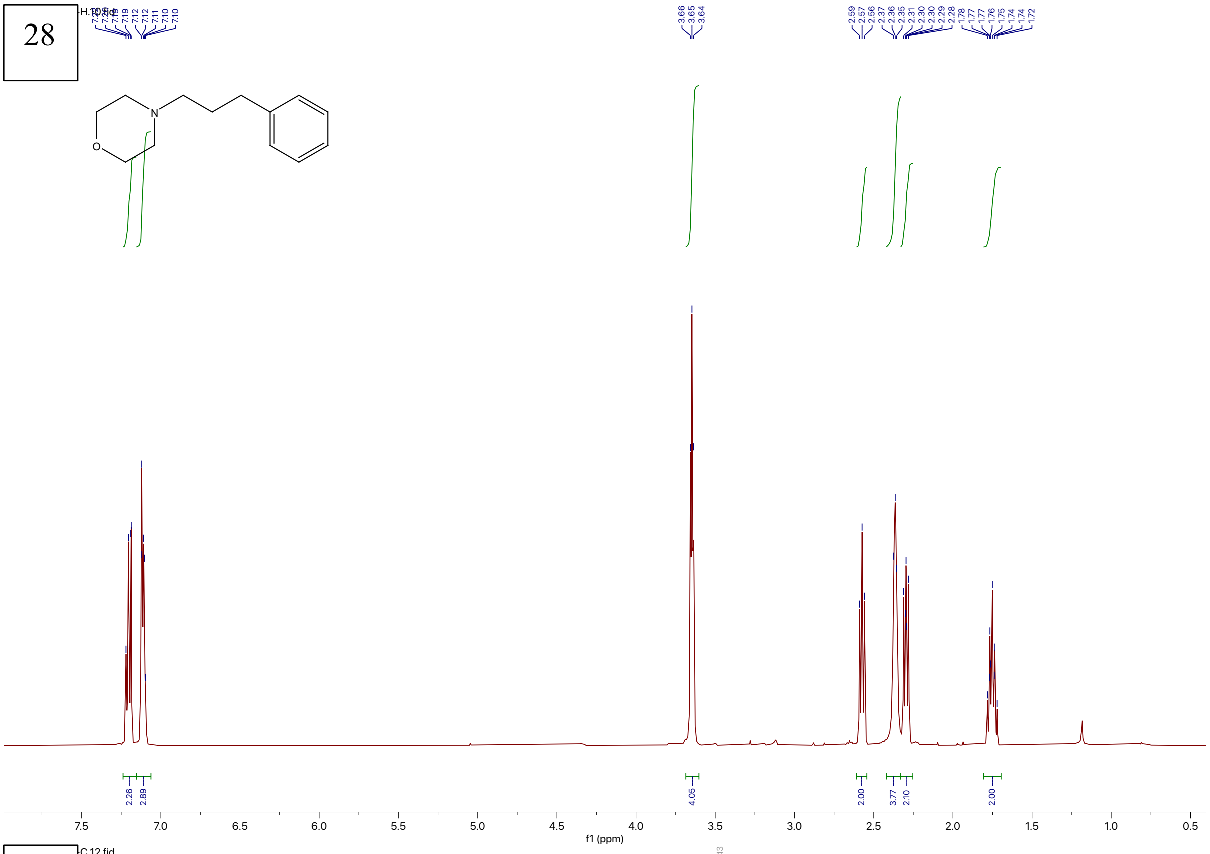
H.10.fid
7.19 7.12 7.12 7.11 7.10 7.10
3.66 3.65 3.64
2.59 2.57 2.56 2.37 2.36 2.35 2.31 2.30 2.30 2.29 2.28 1.78 1.77 1.77 1.76 1.75 1.74 1.74 1.72
O
N
2.26 2.89 4.05 2.00 3.77 2.10 2.00
f1 (ppm)

28

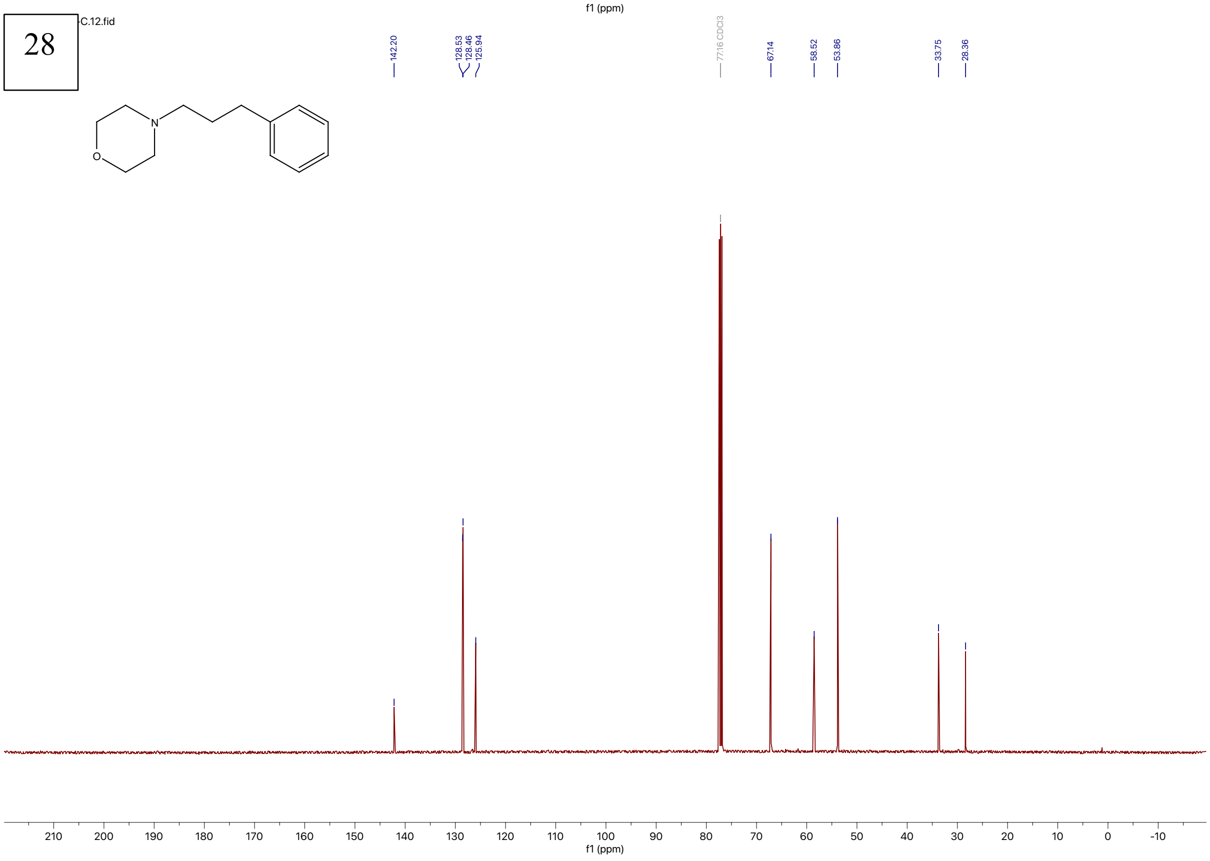
C.12.fid
142.20
128.53 128.46 125.94
77.16 CDCl3
67.14
58.52
53.86
33.75
28.36
O
N
f1 (ppm)

32

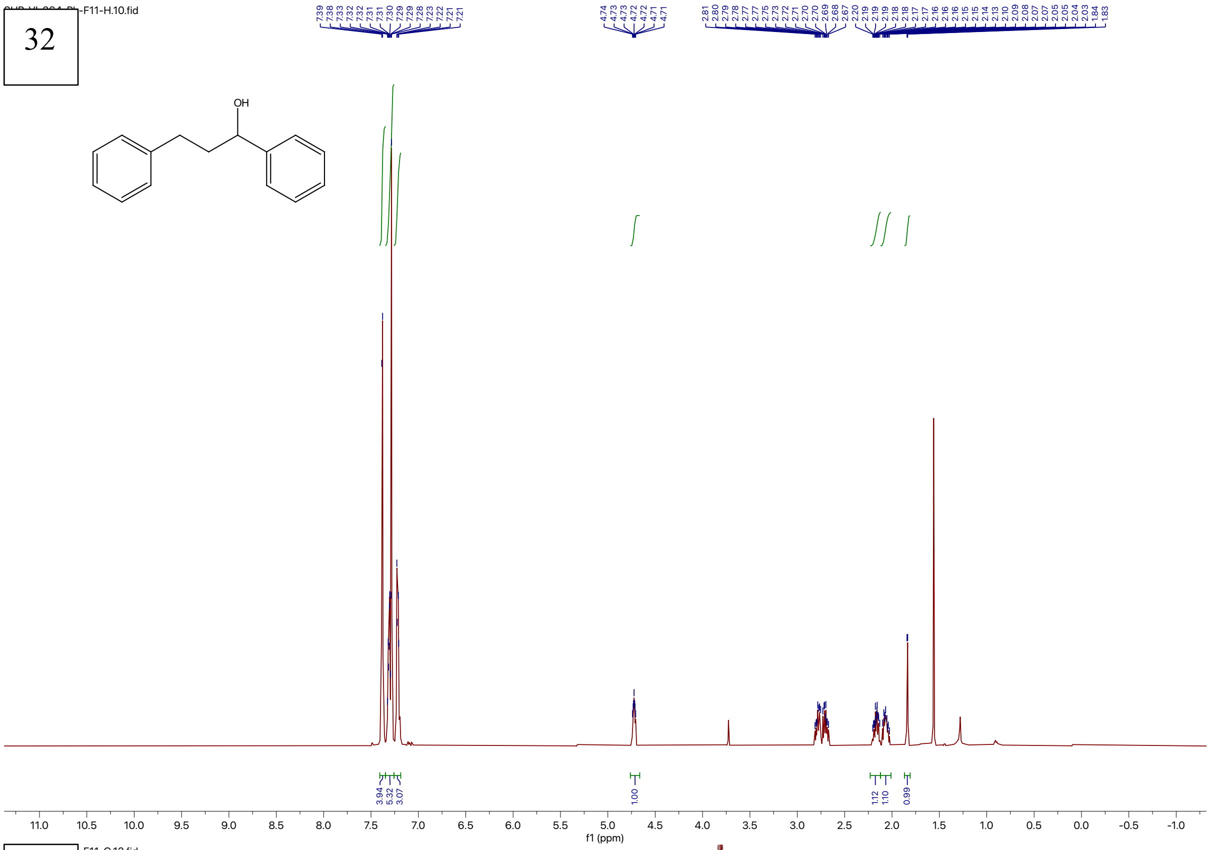

32

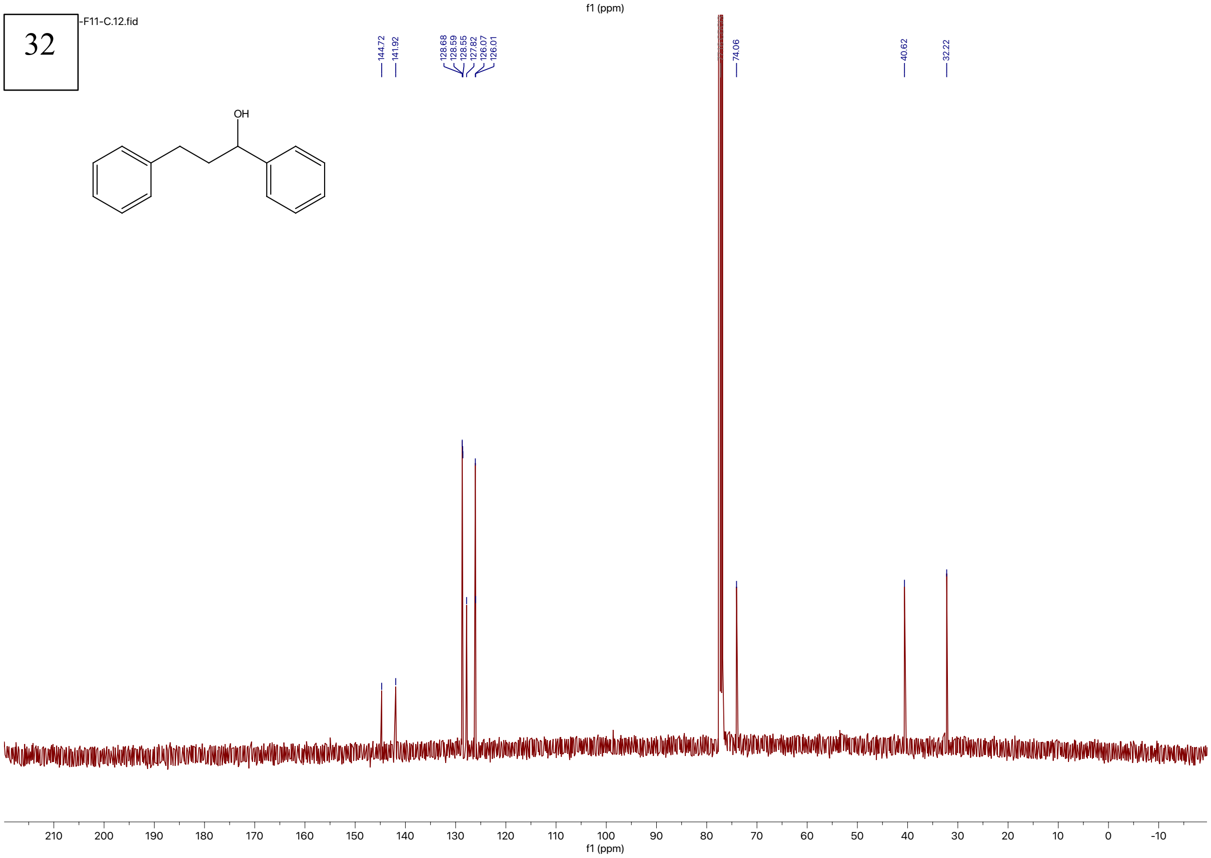

33

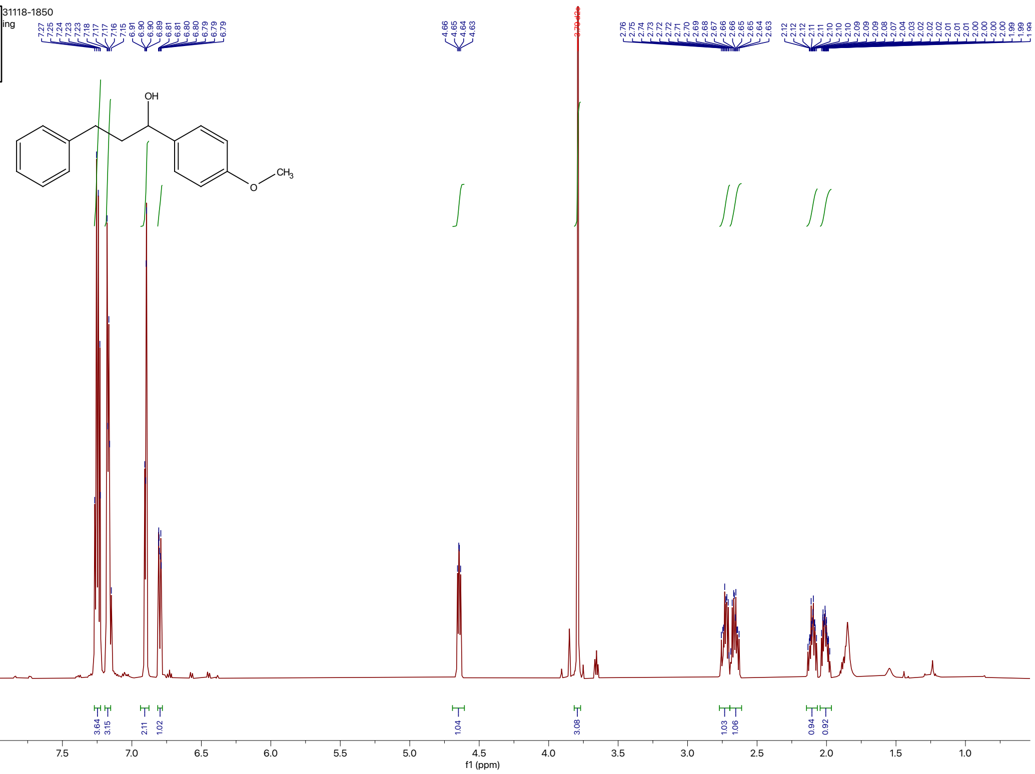

31118-1850
OH
O
CH3
f1 (ppm)


33

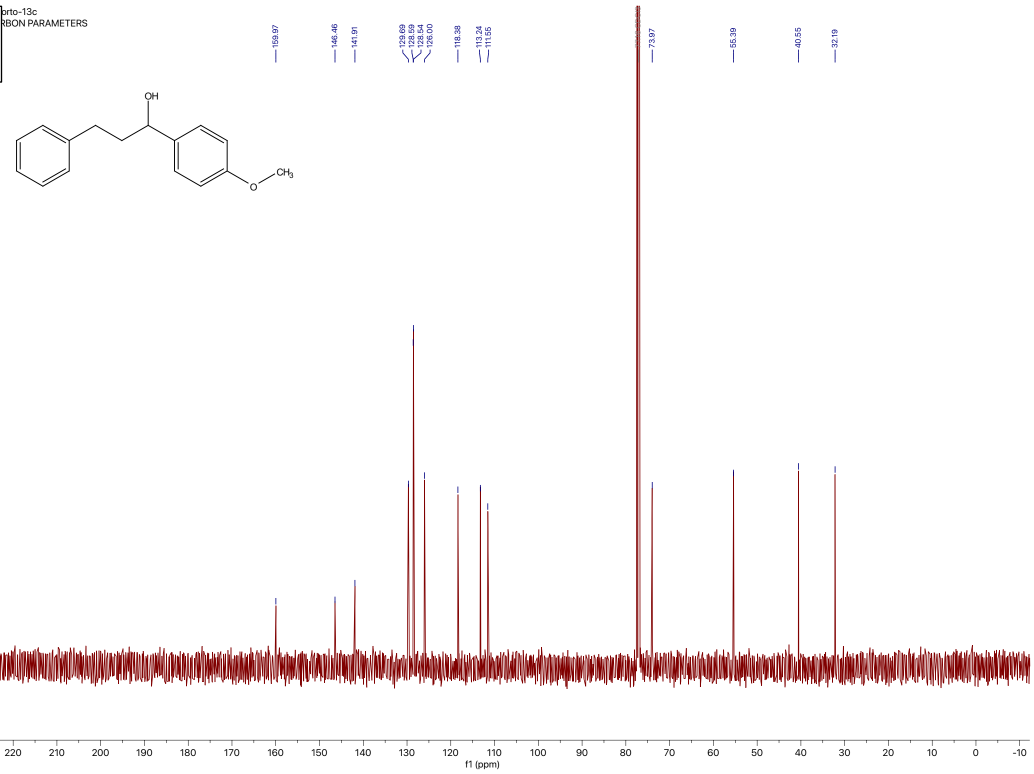

rto-13c
RBON PARAMETERS
OH
O
CH3
159.97
146.46
141.91
129.69
128.59
128.54
126.00
118.38
113.24
111.55
73.97
55.39
40.55
32.19
f1 (ppm)

34

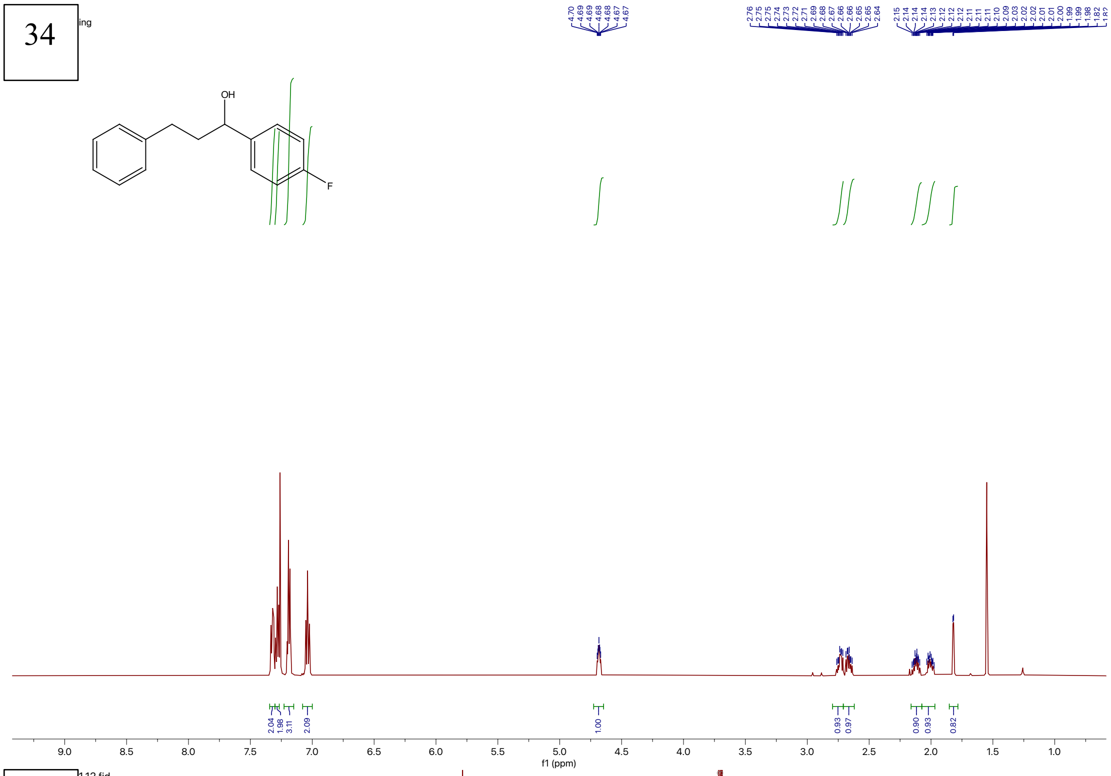
ing
OH
F
2.04
1.98
3.11
2.09
1.00
0.93
0.97
0.90
0.93
0.82
f1 (ppm)

34

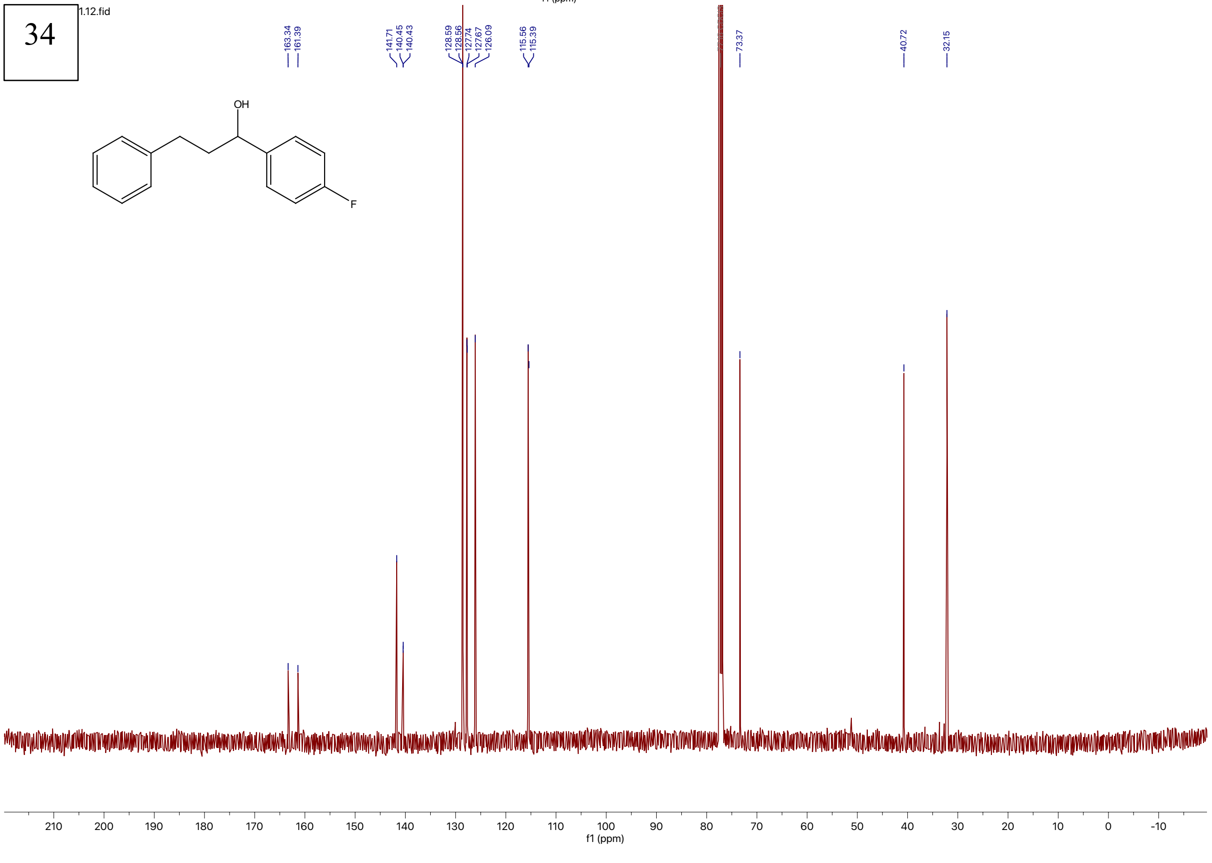
1.12.fid
163.34
161.39
141.71
140.45
140.43
128.59
128.56
127.74
127.67
126.09
115.56
115.39
73.37
40.72
32.15
OH
F
f1 (ppm)

35

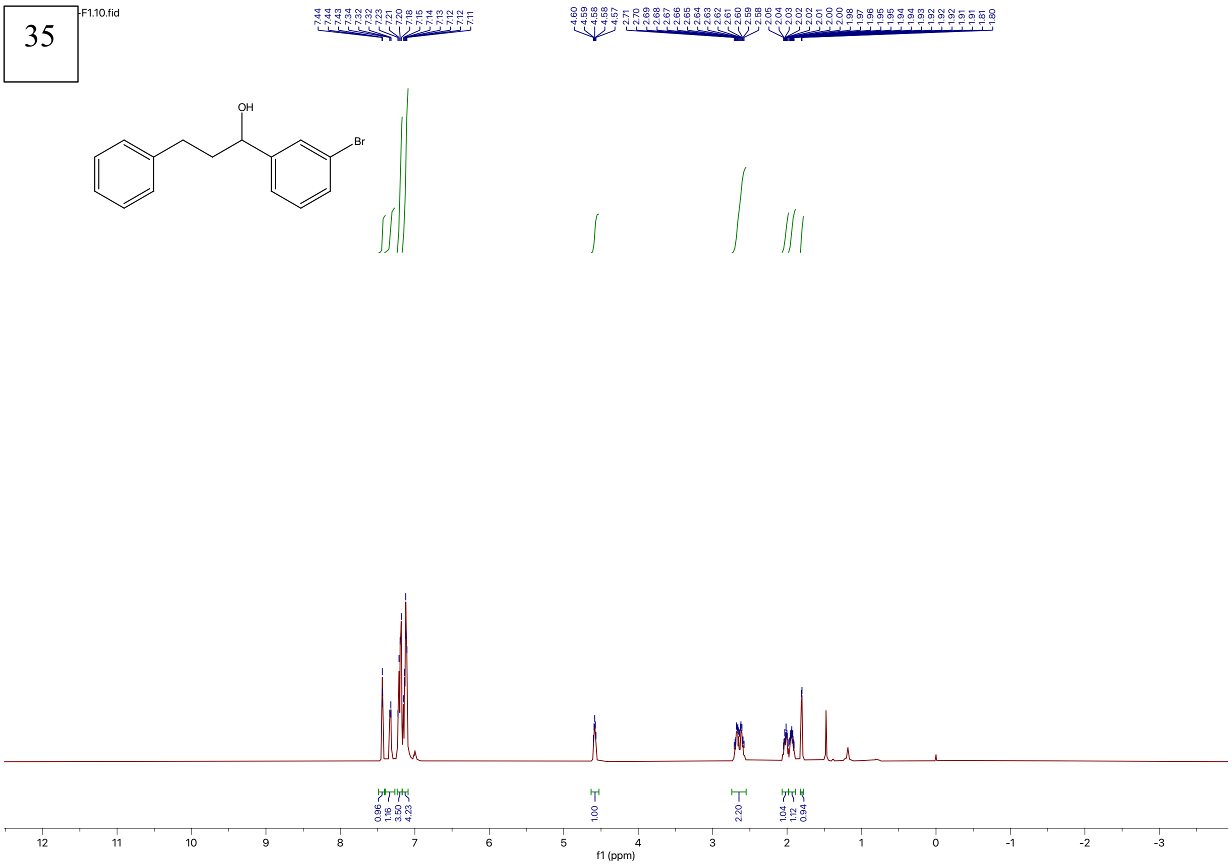
F1.10.fid
OH
Br
f1 (ppm)

35

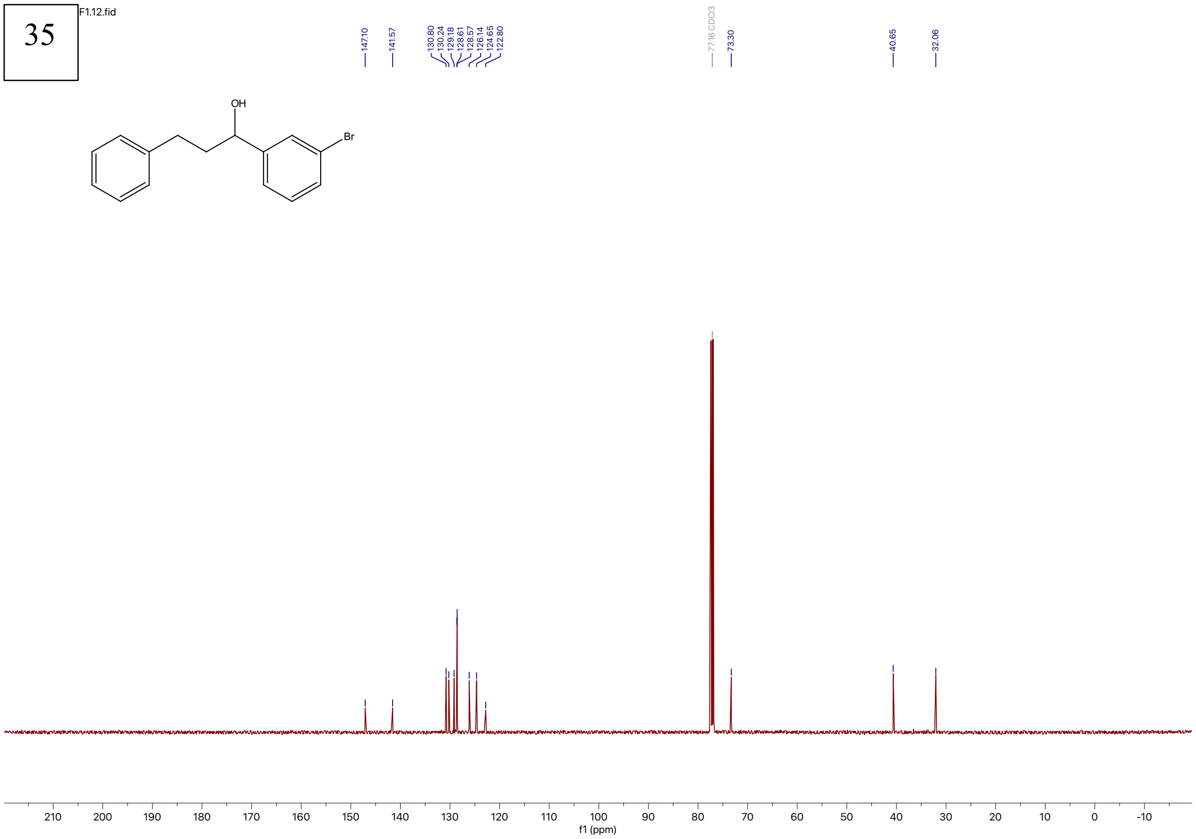
F1.12.fid
147.10
141.57
130.80
130.24
129.18
128.61
128.57
126.14
124.65
122.80
77.16 CDCl3
73.30
40.65
32.06
OH
Br
f1 (ppm)

38

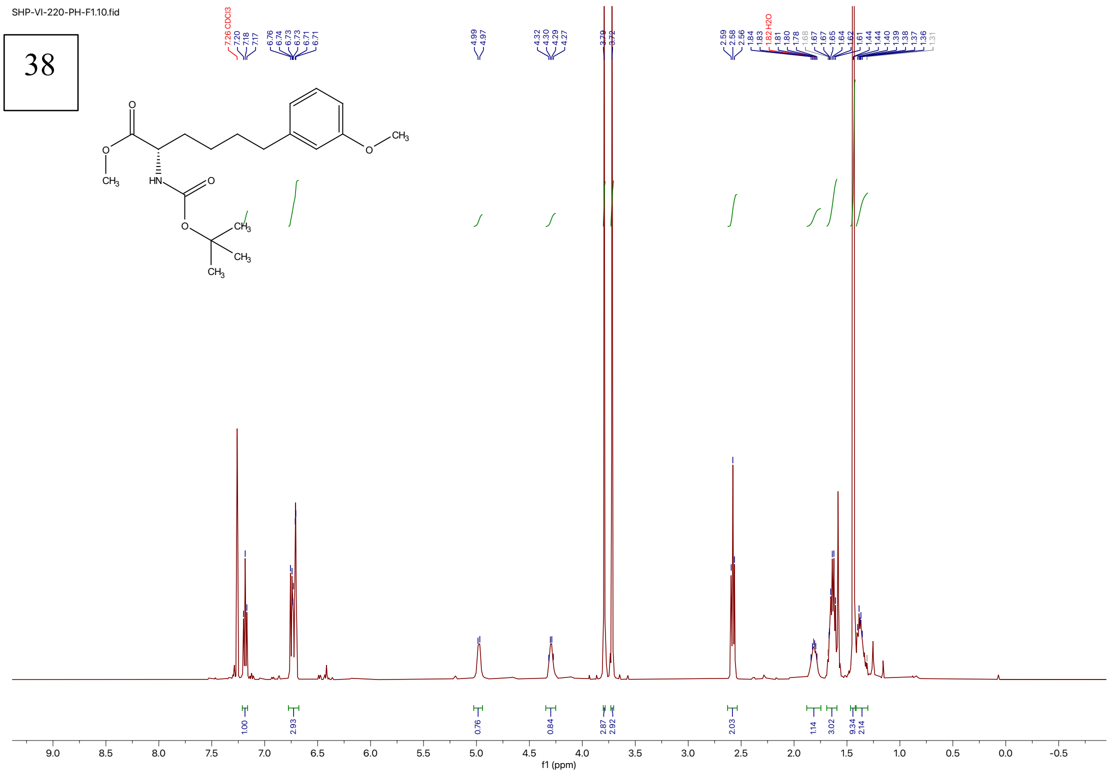
SHP-VI-220-PH-F1.10.fid
7.26 CDCl3
7.20
7.18
7.17
6.76
6.74
6.73
6.73
6.71
6.71
4.99
4.97
4.32
4.30
4.29
4.27
3.79
3.72
2.59
2.58
2.56
1.84
1.83
1.82 H2O
1.81
1.80
1.78
1.68
1.67
1.67
1.65
1.64
1.62
1.61
1.44
1.44
1.40
1.39
1.38
1.37
1.36
1.31
CH3
HN
O
1.00
2.93
0.76
0.84
2.87
2.92
2.03
1.14
3.02
9.34
2.14
f1 (ppm)

38

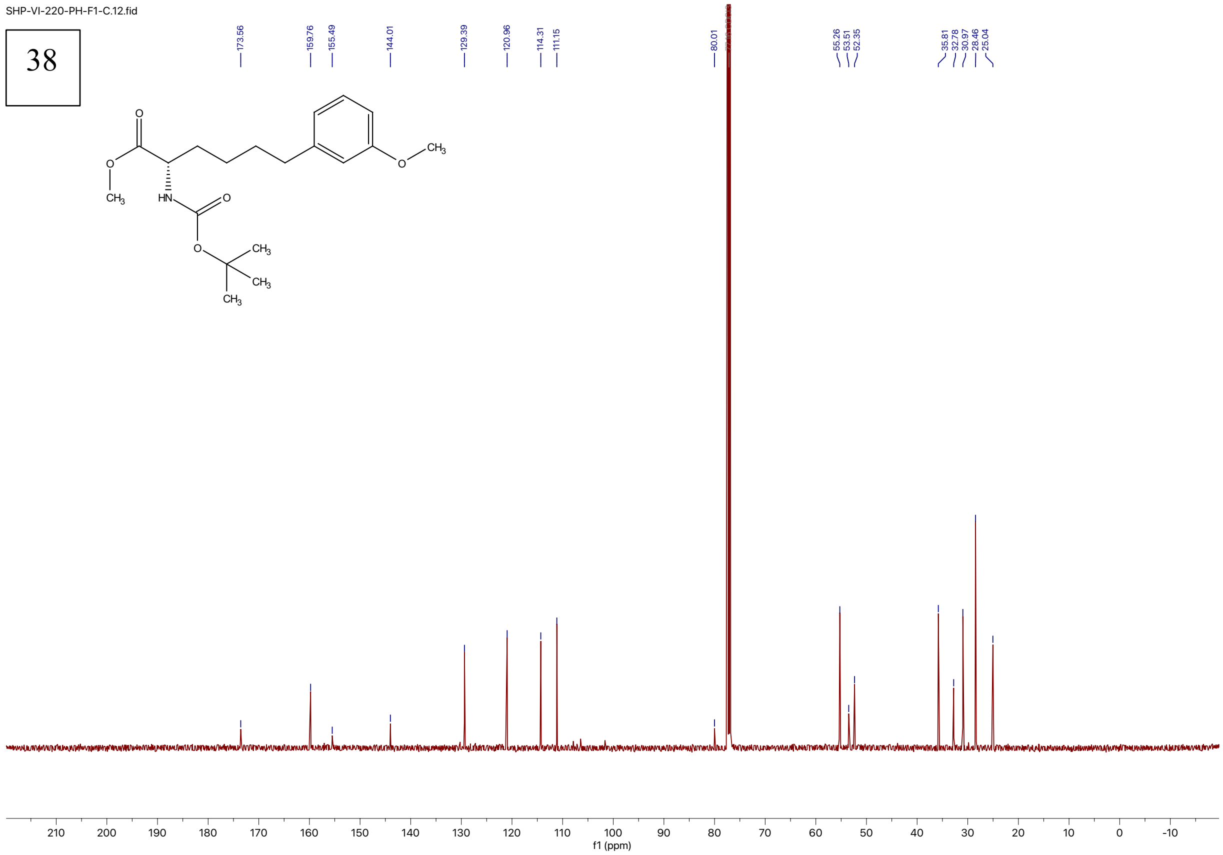
SHP-VI-220-PH-F1-C.12.fid
173.56
159.76
155.49
144.01
129.39
120.96
114.31
111.15
80.01
55.26
53.51
52.35
35.81
32.78
30.97
28.46
25.04
CH3
HN
O
f1 (ppm)

39

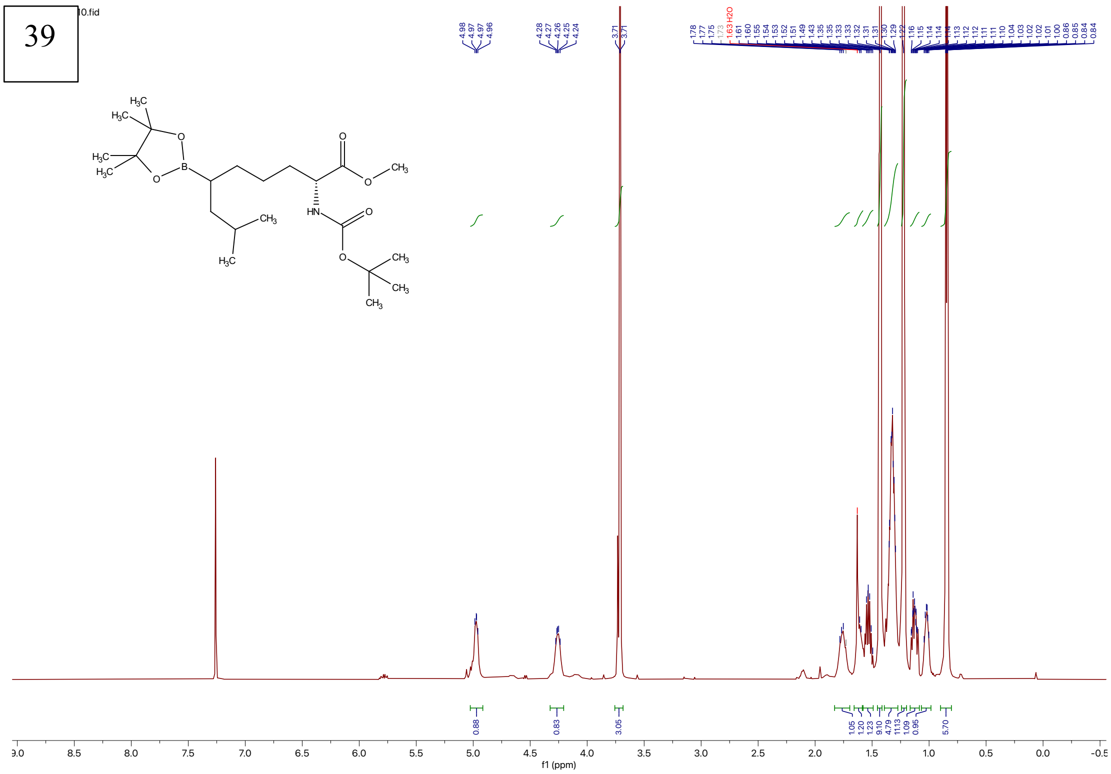


39

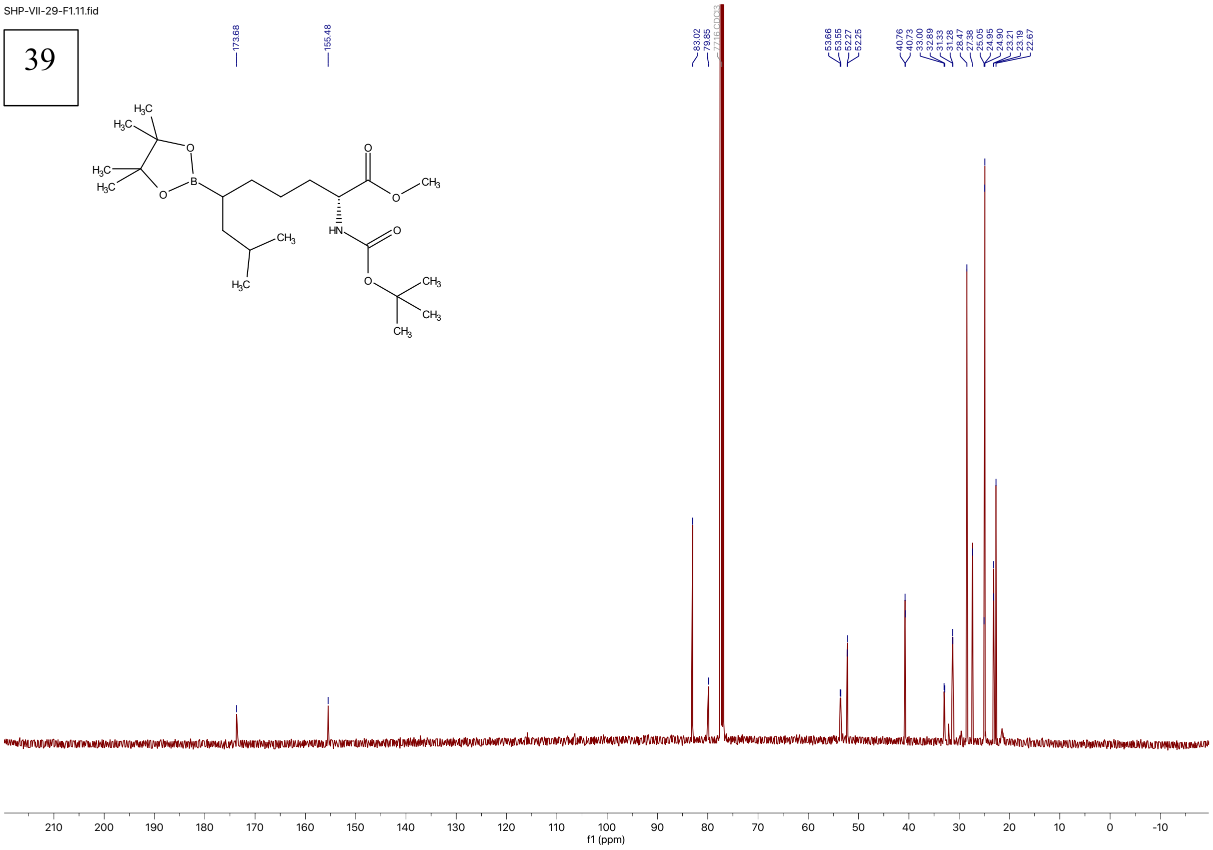

40

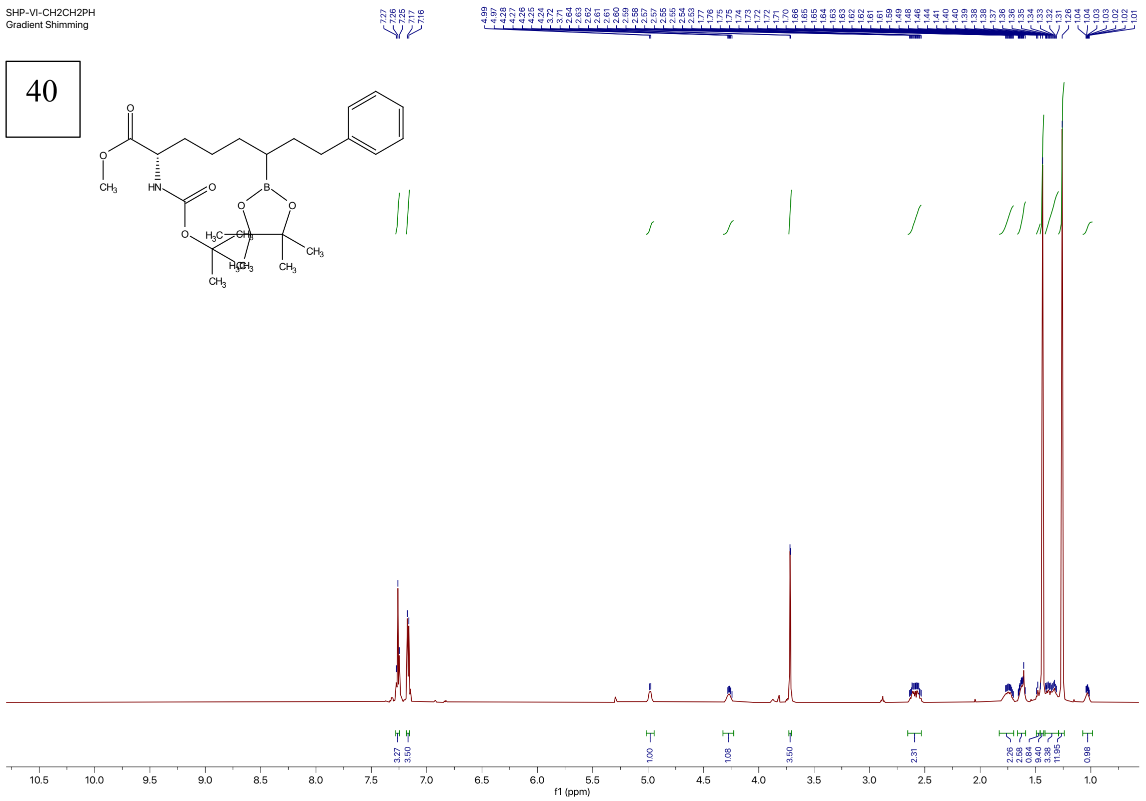
SHP-VI-CH2CH2PH
Gradient Shimming
f1 (ppm)

40

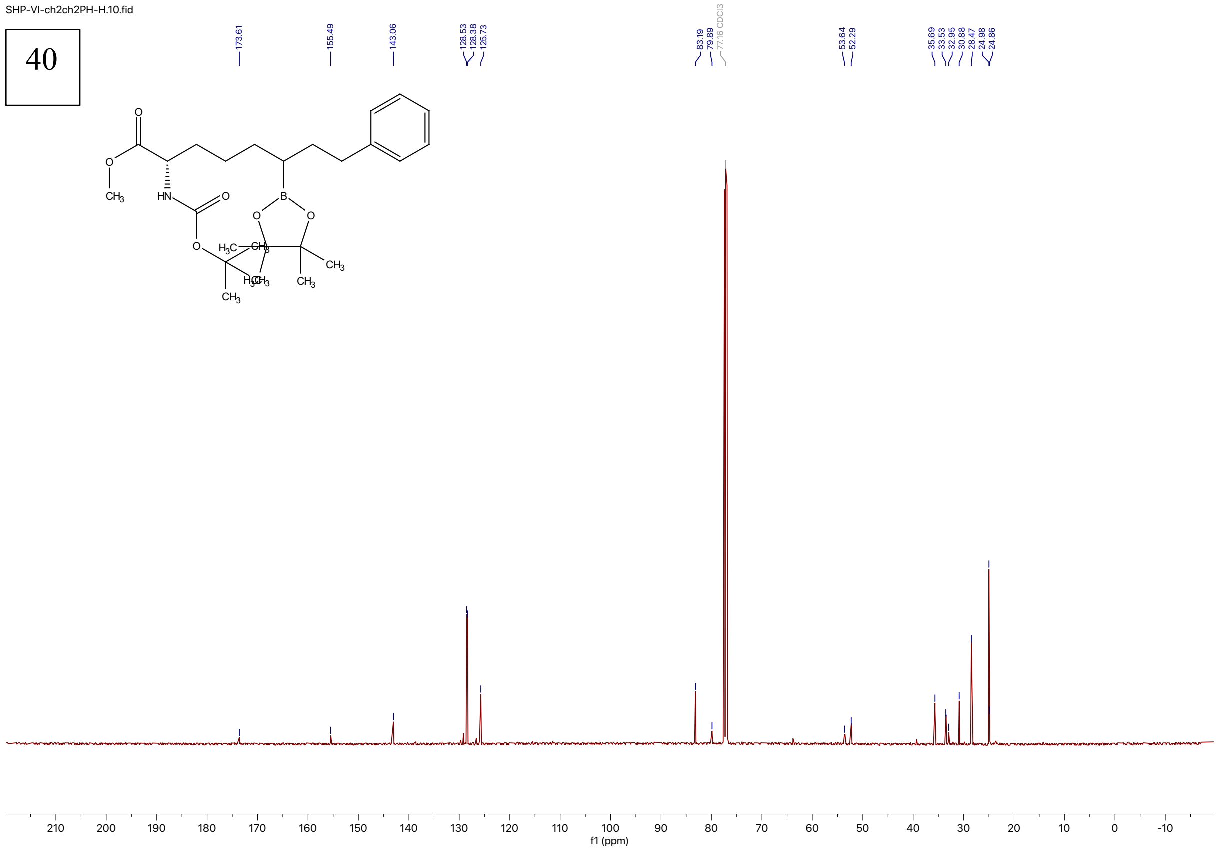
SHP-VI-ch2ch2PH-H.10.fid
173.61
155.49
143.06
128.53
128.38
125.73
83.19
79.89
77.16 CDCl3
53.64
52.29
35.69
33.53
32.95
30.88
28.47
24.98
24.86
f1 (ppm)

41

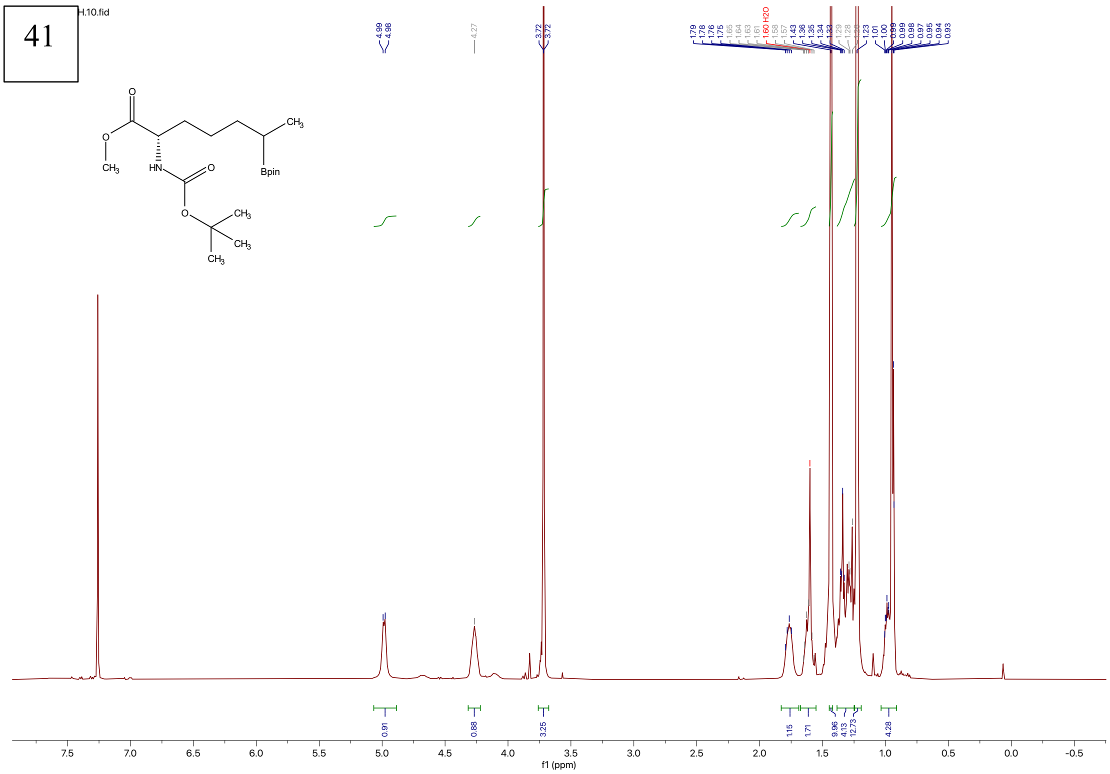

41

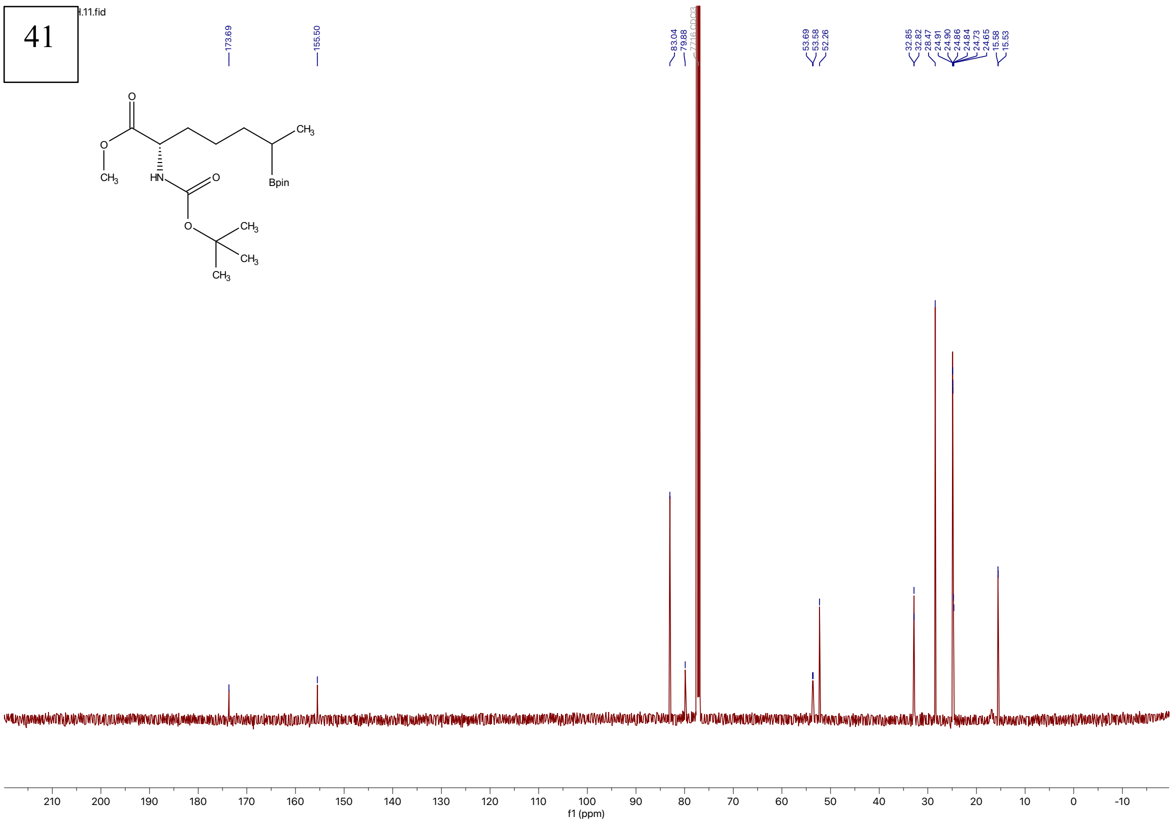

42

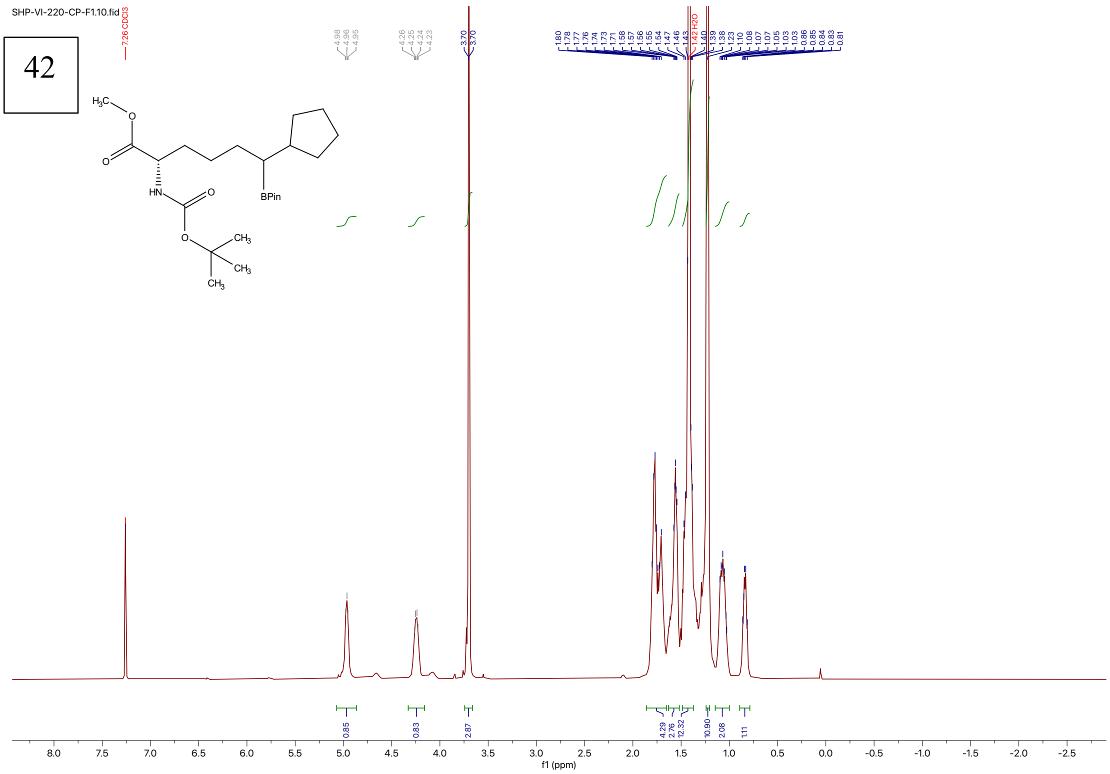
SHP-VI-220-CP-F1.10.fid
7.26 CDCl3
H3C
O
O
HN
O
O
CH3
CH3
CH3
BPin
0.85
0.83
2.87
4.29
2.76
12.32
10.90
2.08
1.11
f1 (ppm)

42

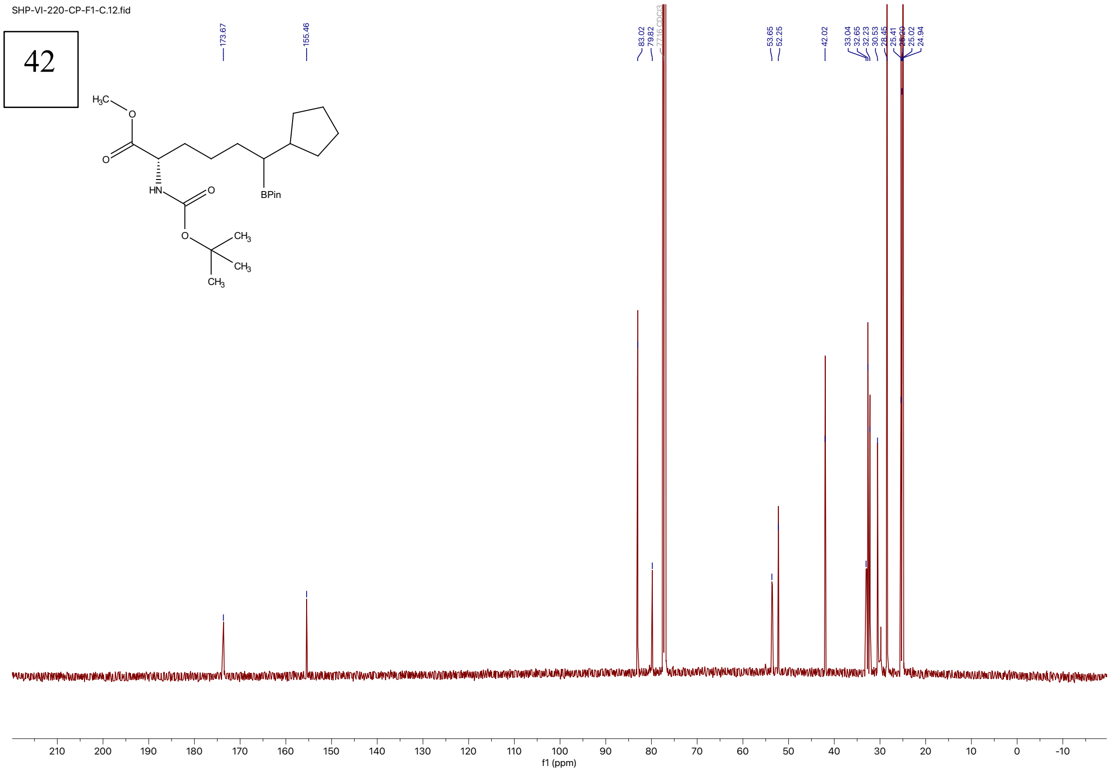
SHP-VI-220-CP-F1-C.12.fid
173.67
155.46
83.02
79.82
53.65
52.25
42.02
H3C
O
O
HN
O
O
CH3
CH3
CH3
BPin
f1 (ppm)

43

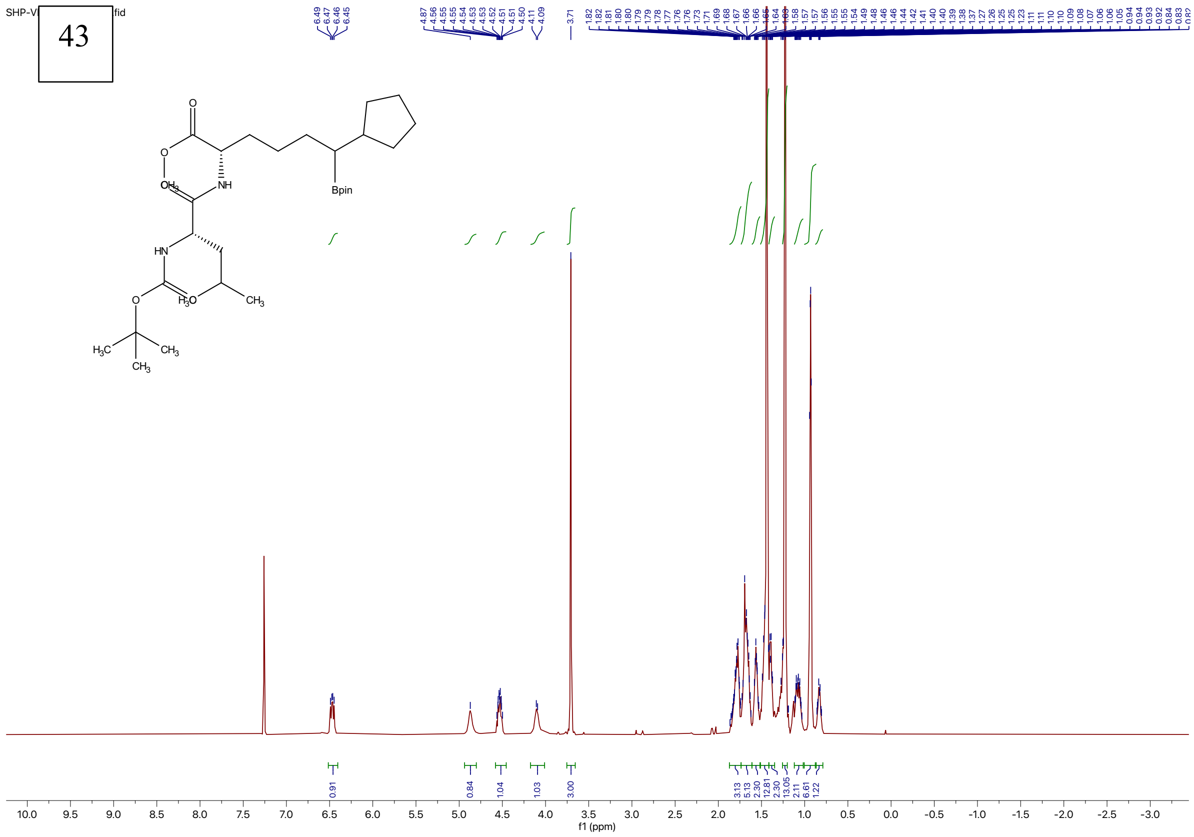

Bpin
f1 (ppm)


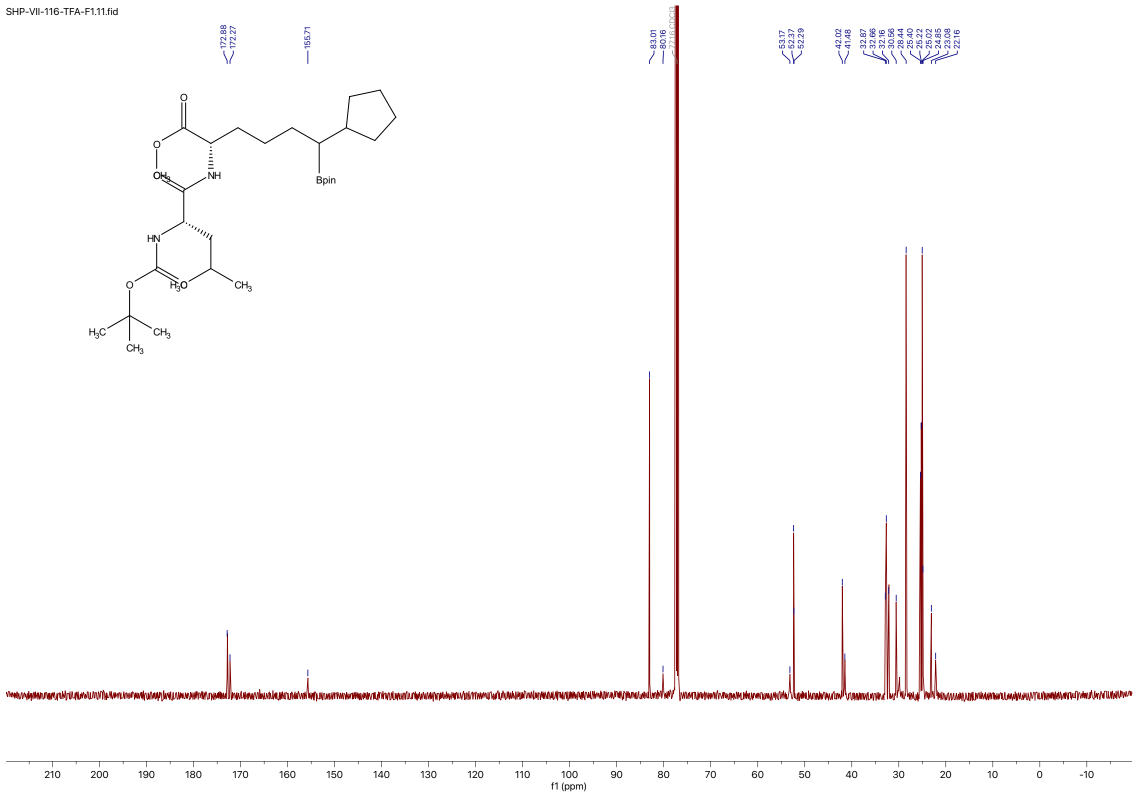

SHP-VII-116-TFA-F1.11.fid
172.88
172.27
155.71
83.01
80.16
53.17
52.37
52.29
42.02
41.48
32.87
32.66
32.16
30.56
28.44
25.40
25.22
25.02
24.85
23.08
22.16
Bpin
f1 (ppm)

# XI. HPLC

```
Data File C:\CHEM32\2\DATA\SHP\SHP-IV-273-1207.D
Sample Name: SHP-VI-286-D/L-NEW

    =====================================================================
    Acq. Operator   : SYSTEM                        Seq. Line :    4
    Acq. Instrument : 1220 HPLC                      Location : Vial 31
    Injection Date  : 1/12/2024 3:51:26 PM                Inj :    1
                                                   Inj Volume : 20.000 µl
    Acq. Method     : C:\CHEM32\2\METHODS\MLC_VARIABLE.M
    Last changed    : 1/12/2024 12:23:18 PM by SYSTEM
    Analysis Method : C:\CHEM32\2\METHODS\DEF_LC.M
    Last changed    : 1/12/2024 12:23:00 PM by SYSTEM
    Sample Info     : 1:99 IPA:HEX LUX CELL2 254 nm 1ML/MIN

    Additional Info : Peak(s) manually integrated
```

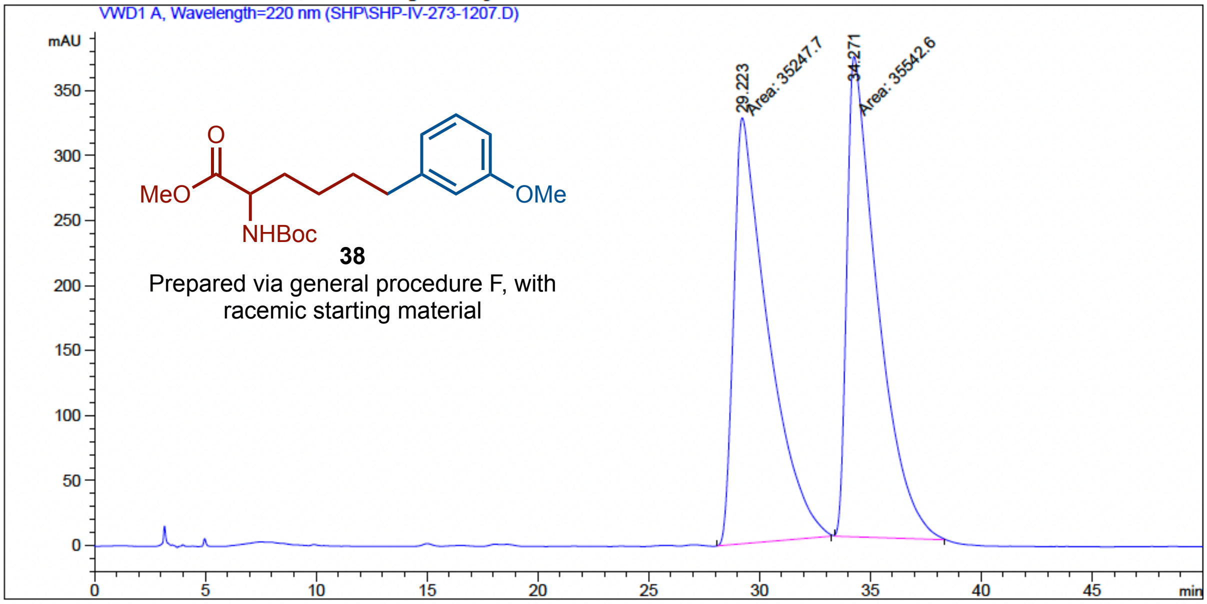


```
    =====================================================================
                         Area Percent Report
    =====================================================================

    Sorted By             :      Signal
    Multiplier:                    :      1.0000
    Dilution:                      :      1.0000
    Use Multiplier & Dilution Factor with ISTDs


    Signal 1: VWD1 A, Wavelength=220 nm

    Peak RetTime Type  Width     Area      Height     Area
      #   [min]        [min]   [mAU*s]     [mAU]       %
    ----|-------|----|-------|----------|----------|--------|
       1  29.223 PM    1.7935 3.52477e4  327.55478  49.7917
       2  34.271 MM    1.6030 3.55426e4  369.54474  50.2083

    Totals :                  7.07903e4  697.09952

    =====================================================================
                              *** End of Report ***

1220 HPLC 1/13/2024 2:44:39 PM SYSTEM                              Page    1 of 1
```

```
Data File C:\CHEM32\2\DATA\SHP\SHP-IV-273-1211.D
Sample Name: SHP-VI-273-BASE

=====================================================================
Acq. Operator   : SYSTEM                          Seq. Line :   8
Acq. Instrument : 1220 HPLC                        Location : Vial 11
Injection Date  : 1/12/2024 7:15:39 PM                  Inj :   1
                                                 Inj Volume : 20.000 µl
Acq. Method     : C:\CHEM32\2\METHODS\MLC_VARIABLE.M
Last changed    : 1/12/2024 12:23:18 PM by SYSTEM
Analysis Method : C:\CHEM32\2\METHODS\DEF_LC.M
Last changed    : 1/12/2024 12:23:00 PM by SYSTEM
Sample Info     : 1:99 IPA:HEX LUX CELL2 254 nm 1ML/MIN

Additional Info : Peak(s) manually integrated
```

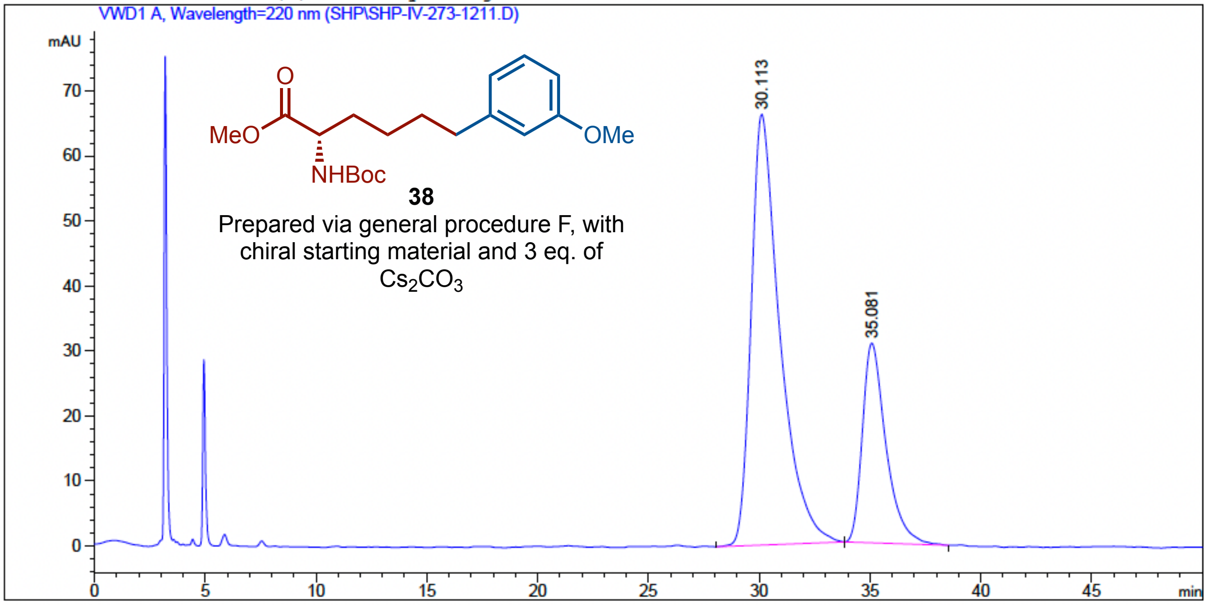


```
=====================================================================
                          Area Percent Report
=====================================================================

Sorted By             :      Signal
Multiplier:                  :       1.0000
Dilution:                    :       1.0000
Use Multiplier & Dilution Factor with ISTDs


Signal 1: VWD1 A, Wavelength=220 nm
```

| Peak # | RetTime [min] | Type | Width [min] | Area [mAU*s] | Height [mAU] | Area % |
|---|---|---|---|---|---|---|
| 1 | 30.113 | BB | 1.3193 | 5988.23535 | 66.32629 | 72.4611 |
| 2 | 35.081 | BB | 1.1032 | 2275.83447 | 30.72376 | 27.5389 |

Totals : 8264.06982 97.05005

```
=====================================================================
                          *** End of Report ***
```

Data File C:\CHEM32\2\DATA\SHP\SHP-IV-273-1210.D
Sample Name: SHP-VI-273-NO-BASE

=====================================================================

Acq. Operator : SYSTEM Seq. Line : 7
Acq. Instrument : 1220 HPLC Location : Vial 1
Injection Date : 1/12/2024 6:24:37 PM Inj : 1
Inj Volume : 20.000 µl
Acq. Method : C:\CHEM32\2\METHODS\MLC_VARIABLE.M
Last changed : 1/12/2024 12:23:18 PM by SYSTEM
Analysis Method : C:\CHEM32\2\METHODS\DEF_LC.M
Last changed : 1/12/2024 12:23:00 PM by SYSTEM
Sample Info : 1:99 IPA:HEX LUX CELL2 254 nm 1ML/MIN

Additional Info : Peak(s) manually integrated

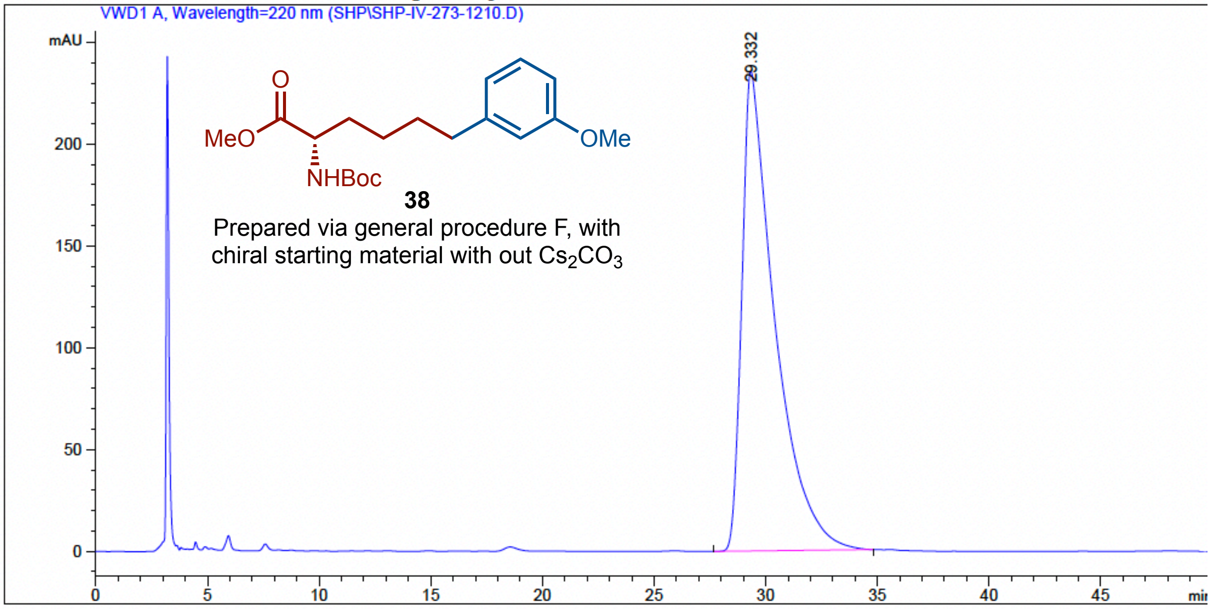


=====================================================================

Area Percent Report

=====================================================================

Sorted By : Signal
Multiplier: : 1.0000
Dilution: : 1.0000
Use Multiplier & Dilution Factor with ISTDs

Signal 1: VWD1 A, Wavelength=220 nm

| Peak # | RetTime [min] | Type | Width [min] | Area [mAU*s] | Height [mAU] | Area % |
|---|---|---|---|---|---|---|
| 1 | 29.332 | BB | 1.4096 | 2.41775e4 | 235.72646 | 100.0000 |

Totals : 2.41775e4 235.72646

=====================================================================

*** End of Report ***

## XII. References


[1]S. M. Hecht, J. W. Kozarich, *J. Org. Chem*. **1973**, *38*, 1821-1824.
[2] I. M. Heyns, R. Pfukwa, B. Klumperman, *Biomacromolecules* **2016**, *17*, 1795-1800.
[3] Z. Ghiasifar, H. Salehabadi, N. Adibpour, E. Alipour, F. Kobarfard, M. R. Shoushizadeh, *BKCS* **2021**, *42*, 48-59.
[4]H. Esaki, R. Ohtaki, T. Maegawa, Y. Monguchi, H. Sajiki, *J. Org. Chem.* **2007**, *72*, 2143-2150.
[5]G. Casoni, M. Kucukdisli, J. M. Fordham, M. Burns, E. L. Myers, V. K. Aggarwal, *J. Am. Chem. Soc.* **2017**, *139*, 11877-11886.
[6]Y. Yang, J. Tsien, A. Ben David, J. M. E. Hughes, R. R. Merchant, T. Qin, *J. Am. Chem. Soc*. **2021**, *143*, 471-480.
[7]M. Angiolini, L. Belvisi, D. Poma, A. Salimbeni, N. Sciammetta, C. Scolastico, *Bioorg Med. Chem.* **1998**, *6*, 2013-2027.
[8] S. Rendler, O. Plefka, B. Karatas, G. Auer, R. Fröhlich, C. Mück-Lichtenfeld, S. Grimme, M. Oestreich, *Eur. J. Chem.* **2008**, *14*, 11512-11528.
[9] T. Kumamoto, K. Hosoya, S. Kanzaki, K. Masuko, M. Watanabe, K. Shirai, *BCSJ.* **2006**, *59*, 3097-3101.
[10]Q.-D. Wang, J.-M. Yang, D. Fang, J. Ren, B. Dong, B. Zhou, B.-B. Zeng, *Tetrahedron Lett.* **2016**, *57*, 2587 2590.
[11] H.-J. Liu, W. Luo, *Can. J. Chem*.**1992**, *70*, 128-134.
[12]F. Sandfort, F. Strieth-Kalthoff, F. J. R. Klauck, M. J. James, F. Glorius, *Eur. J. Chem*. **2018**, *24*, 17210-17214.
[13]S. Imamura, T. Ichikawa, Y. Nishikawa, N. Kanzaki, K. Takashima, S. Niwa, Y. Iizawa, M. Baba, Y. Sugihara, *J. Med. Chem.* **2006**, *49*, 2784-2793.
[14]A. J. Boyington, M.-L. Y. Riu, N. T. Jui, *J. Am. Chem. Soc*. **2017**, *139*, 6582-6585.
[15]S. Li, K. Huang, J. Zhang, W. Wu, X. Zhang, *Org. Lett.* **2013**, *15*, 3078-3081.
[16]P. Satyanarayana, G. M. Reddy, H. Maheswaran, M. L. Kantam, *Adv. Synth. Catal.* **2013**, *355*, 1859-1867.
[17]T. J. Sherbow, C. R. Carr, T. Saisu, J. C. Fettinger, L. A. Berben, *Organometallics* **2016**, *35*, 9-14.
[18]T. W. Ng, G. Liao, K. K. Lau, H.-J. Pan, Y. Zhao, *Angew. Chem. Int. Ed.* **2020**, *59*, 11384-11389.
[19]T. Liu, L. Wang, K. Wu, Z. Yu, *ACS Catal.* **2018**, *8*, 7201-7207.
[20] J. C. Twitty, Y. Hong, B. Garcia, S. Tsang, J. Liao, D. M. Schultz, J. Hanisak, S. L. Zultanski, A. Dion, D. Kalyani, M. P. Watson, *J. Am. Chem. Soc*. **2023**, *145*, 5684-5695.
